# Flagellin adjuvanted F1/V subunit plague vaccine induces T cell and functional antibody responses with unique gene signatures

**DOI:** 10.1038/s41541-020-0156-y

**Published:** 2020-01-23

**Authors:** Fahreta Hamzabegovic, Johannes B. Goll, William F. Hooper, Sharon Frey, Casey E. Gelber, Getahun Abate

**Affiliations:** 1 Division of Infectious Diseases, Allergy and Immunology, Saint Louis University, Saint Louis, MO USA; 2 EMMES, Rockville, MD USA

**Keywords:** Peptide vaccines, Adjuvants

## Abstract

*Yersinia pestis*, the cause of plague, could be weaponized. Unfortunately, development of new vaccines is limited by lack of correlates of protection. We used pre- and post-vaccination sera and peripheral blood mononuclear cells from a flagellin adjuvanted F1/V vaccine trial to evaluate for protective markers. Here, we report for the first time in humans that inverse caspase-3 levels, which are measures of protective antibody, significantly increased by 29% and 75% on days 14 and 28 post-second vaccination, respectively. In addition, there were significant increases in T-cell responses on day 28 post-second vaccination. The strongest positive and negative correlations between protective antibody levels and gene expression signatures were identified for *IFNG* and ENSG00000225107 genes, respectively. Flagellin/F1/V subunit vaccine induced macrophage-protective antibody and significant CD4^+^ T-cell responses. Several genes associated with these responses were identified that could serve as potential correlates of protection.

## Introduction

*Yersinia pestis* (Yp), the causative agent of plague, is a category A potential bioterrorism pathogen that poses a high risk to both national security and public health. Plague has three major clinical forms, bubonic, pneumonic, and septicemic, but the highest concern is for the pneumonic form which allows for direct person-to-person transmission via infectious respiratory droplets and therefore remains a serious public health threat. Thus, due attention should be given to public health preparedness including prevention and early detection. Vaccines are effective in preventing and controlling infectious diseases. However, there is no approved plague vaccine and vaccine development efforts are hampered by a lack of reliable markers of protection.

Plague is currently a rare disease worldwide and is associated with high mortality; therefore, measuring vaccine efficacy based on protection from natural infection is impractical. This means, as with other category A pathogens, plague vaccine development efforts need to rely on inferred correlates of protection, which requires a good understanding of immunity against Yp.

Animal studies have demonstrated that both antibody and cell-mediated immunity (CMI) are essential for protection against challenge with Yp.^1–10^ Different forms of plague vaccines including killed Yp, live attenuated Yp, and subunit vaccines have been studied. Subunit vaccines containing F1 capsular and virulence (V) antigens show the most promising results. A vaccine that had F1 and V antigens mixed with alhydrogel adjuvant was shown to elicit antibody responses in humans, but without measurable CMI.^11^ Interestingly, the post-vaccination sera from this clinical trial protected mice from lethal Yp challenge. Similarly, a recent dose titration clinical trial with a new F1/V subunit vaccine containing flagellin as an adjuvant conducted by the Vaccine and Treatment Evaluation Unit (VTEU) network showed good antibody responses at 6 and 10 μg, again in the absence of significant CMI.^12^ This vaccine was shown to induce excellent antibody responses in mice and non-human primates (NHP), and protect mice against respiratory challenge with Yp.^13^ The protective capacity of antibody responses induced by flagellin-adjuvanted F1/V plague vaccine in humans remains to be studied. The lack of CMI from both clinical trials with subunit vaccines was unexpected because these same subunit vaccines have been shown to elicit protective CMI in animal models.^4,5,10^ One possible explanation for the lack of measurable vaccine-specific CMI found in subunit plague vaccine trials is the limitation of the in vitro assays used (e.g., antigen concentration and duration of in vitro restimulation of T cells). In the first trial, the T-cell activation markers and gross changes in T-cell counts were measured ex vivo without antigenic restimulation.^11^ In the recently completed VTEU clinical trial,^12^ only 24 h stimulation with F1/V antigens was used before collection of culture supernatants for cytokine quantification. Vaccine-specific T cells are generally of low frequency and can be measured reliably only after optimal in vitro stimulation.^14^ This study was carried out with the objectives of evaluating the protective function of antibodies elicited by flagellin adjuvanted F1/V vaccine, reevaluating vaccine-induced T-cell responses using optimal in vitro restimulation conditions, and identifying gene expression markers of good vaccine-induced immune responses.

## Results

### Antibody responses induced by F1/V vaccine prevent macrophage lytic effects of a recombinant Yptb

**Fig. 1:**
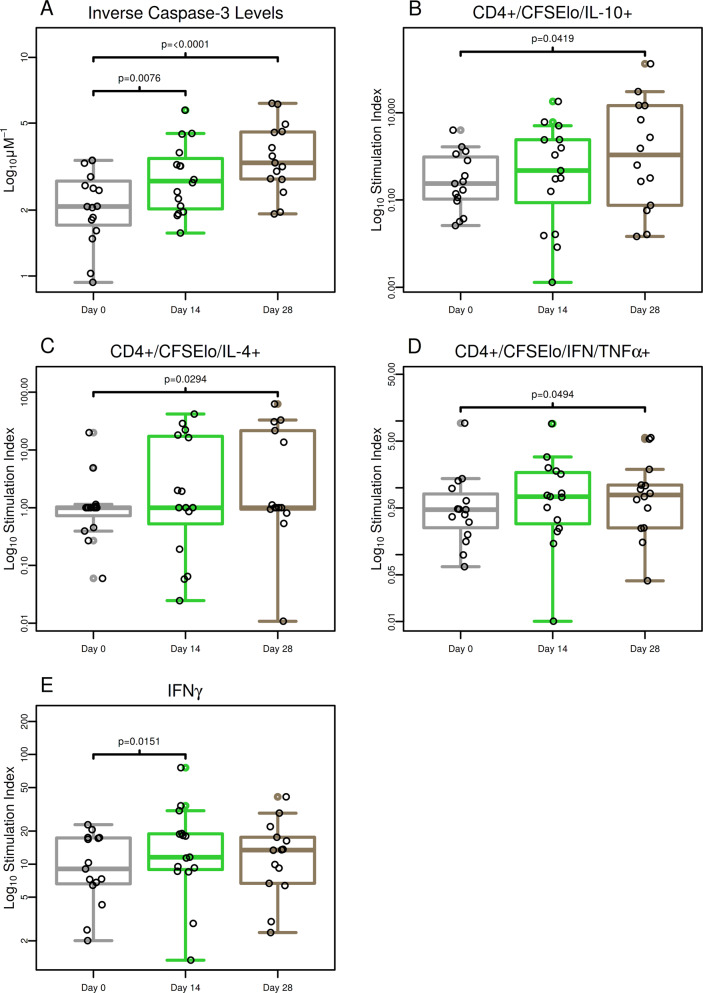
F1/V vaccine increased protective levels of antibody, CD4+ T-cell, and IFN-γ responses. **a** Macrophage-protective levels of antibodies were statistically significantly increased from pre-vaccination on days 14 and 28 with highest response on day 28 post-second vaccination (Wilcoxon signed-rank test *P*-values are shown). **b**–**d** CD4+ that expressed IL-10, IL-4, or co-expressed TNF-α and IFN-γ were statistically increased from pre-vaccination on day 28 but not day 14. **e** IFN-γ cytokine concentrations measured in culture supernatants were statistically significantly increased from pre-vaccination on day 14 but not day 28. Statistical significance is based on Wilcoxon signed-rank test.

We used the caspase-3 assay to determine the ability of vaccine-induced antibodies to protect macrophages from lytic effect of recombinant *Yersinia pseudotuberculosis* (Yptb) expressing V antigen. Caspase 3 release is a hallmark of apoptosis.^15^ Figure 1 shows the inverse anti-V caspase-3 levels by study visit day and treatment group. Tabular results for per-visit and fold change results are provided in Supplementary Table 3. Combined results for samples from volunteers vaccinated with 6 and 10 μg of F1/V vaccine showed that median inverse caspase-3 levels increased by 29% on day 14 (median fold change of 1.29 and *P* = 0.0076) and 75% on day 28 (median fold change of 1.75 and *P* < 0.0001) post-second vaccination (Fig. 1a, Supplementary Table 3).

### Macrophage protection from lysis correlated with anti-V ELISA antibody titer

We assessed Spearman correlation between log_2_ fold change in protective levels of antibody based on inverse normalized caspase-3 levels and change in IgG ELISA titer as well as IgG ELISA concentration against the F1 and V antigens obtained as part of the associated clinical trial^12^ (Supplementary Table 5). Corresponding scatterplots with trend lines are shown in Supplementary Figs 2 and 3. On day 28 post-second vaccination, the positive correlation between change in ELISA IgG titer against the V antigen and change in macrophage-protective antibody levels was statistically significant (*P* = 0.049, Spearman *r* = 0.53). Corresponding correlation results for ELISA IgG concentration were positive as well but not statistically significant (*P* = 0.056, Spearman *r* = 0.52). ELISA results against the F1 antigen were not significantly correlated with macrophage protection.

### F1/V subunit vaccine induced significant CD4 T-cell and IFN-γ cytokine responses

**Fig. 2:**
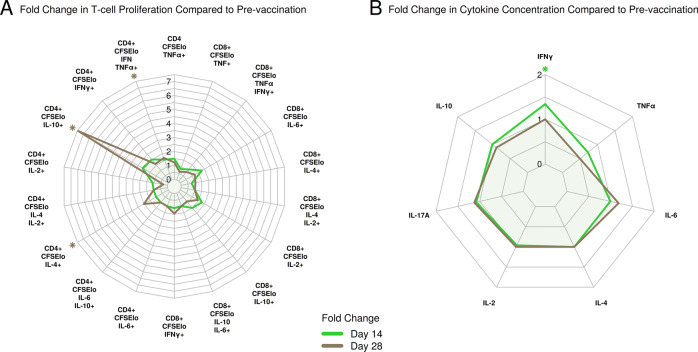
Overview of median CD4+ and CD8+ proliferating T-cell and serum cytokine fold changes. **a** Fold change in T-cell proliferation. **b** Change in serum cytokine concentration. Statistical significance (*P* < 0.05) based on Wilcoxon signed-rank test is highlighted by asterisks.

To assess the proliferation of cytokine-producing T cells following vaccination with F1/V subunit, we stimulated peripheral blood mononuclear cells (PBMCs) with 10 μg/ml of F1/V antigen in vitro and assessed the degree of proliferation compared to medium rest (stimulation index) pre- and post-vaccination (Fig. 2, Supplementary Table 4). Compared to pre-vaccination, day 28 post-second vaccination samples had 7.43 times more CD4^+^/CFSE^lo^/IL-10^+^ T cells (*P* = 0.042) (Fig. 1b). A less strong increase of 2.02-fold was seen in CD4^+^/CFSE^lo^/IL-4^+^ T cells on day 28 post-second vaccination (*P* = 0.029) (Fig. 1c). Interestingly, polyfunctional T cells which proliferated and expressed multiple cytokines also increased significantly after vaccination. There was a 68% increase in CD4^+^/CFSE^lo^/IFN-γ^+^/TNF-α^+^ T cells on day 28 post-second vaccination (*P* = 0.049) (Fig. 1d). Among cytokines measured in culture supernatants, the stimulation index significantly increased by 34% for IFN-γ on day 14 post-second vaccination (*P* = 0.015) (Fig. 1e, Supplementary Table 4). No statistically significant changes were observed for CD8+ cells.

When assessing correlations for CD4^+^/CFSE^lo^/IL-6^+^ and CD4^+^/CFSE^lo^/IFN-γ^+^ T cells on days 14 and 28 post-second vaccination, a positive correlation between protective antibody responses (inverse normalized caspase-3 levels) was observed for changes in CD4^+^/CFSE^lo^/IFN-γ^+^ T cells on day 14 post-second vaccination and macrophage-protective antibody levels on day 28 post-second vaccination (Supplementary Table 6). However, this correlation was not statistically significant (*P* = 0.4, Spearman *r* = 0.244). The strongest negative correlation was observed between changes in CD4^+^/CFSE^lo^/IL-6^+^ T cells and macrophage protection on day 14 post-second vaccination (*P* = 0.026, Spearman *r* = −0.57)

### Vaccination with subunit F1/V vaccine elicited unique gene expression signatures that were primarily downregulated from pre-vaccination

**Fig. 3:**
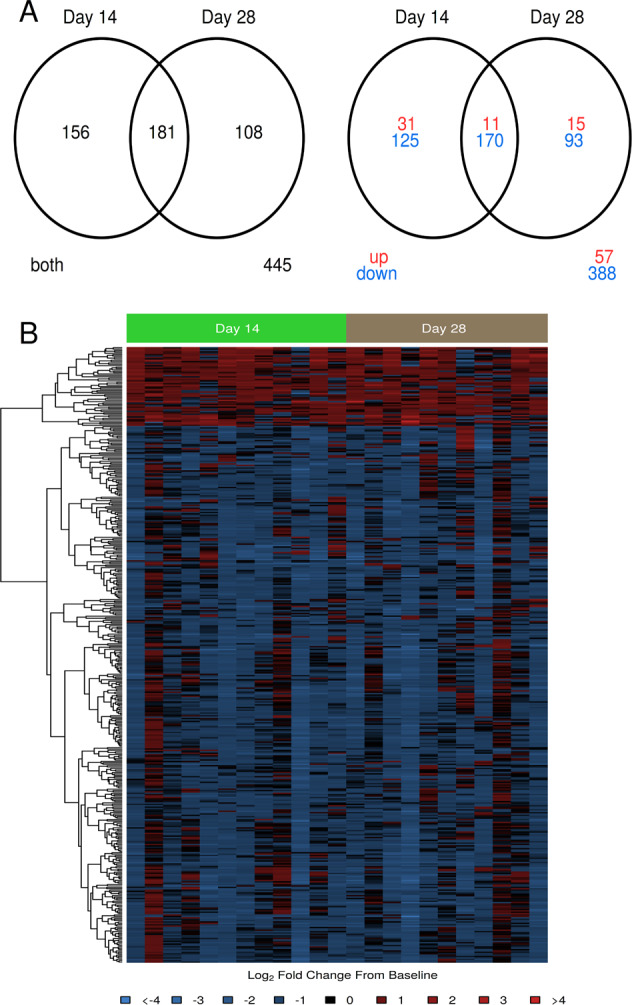
Gene expression signatures following F1/V vaccine that showed statistically differential responses compared to pre-vaccination were primarily downregulated. **a** Venn digrams summarizing overlap in DE genes between days 14 and 28 post-second vaccination. The first shows total differentially expressed genes and the second part shows the number of upregulated and downregulated genes separately. **b** Heatmap of log_2_ fold change from pre-vaccination. Rows represent DE genes, columns represent samples. In red: upregulated compared to pre-vaccination; in blue: downregulated compared to pre-vaccination. Dendrograms were obtained using complete linkage clustering of uncentered pairwise Pearson correlation distances between log_2_ fold changes.

To see how the subunit F1/V vaccine modulated genome-wide gene expression responses in PBMCs, we stimulated PBMCs in vitro with F1/V vaccine antigens for 24 h and measured pre vs. post-vaccination signatures for 12 subjects using RNA-Seq. A total of 337 genes showed differentially-expressed (DE) responses on day 14 post-second vaccination compared to pre-vaccination with 295 (88%) being downregulated (Fig. 3a, Supplementary Table 12). On day 28 post-second vaccination, 289 genes were DE compared to pre-vaccination, with 263 (91%) being downregulated (Supplementary Table 13). The overlap between both post-vaccination days was 181 genes of which 11 (6%) were upregulated and 170 (94%) were downregulated. Figure 3b shows heat maps that summarize gene response for the union of 445 DE genes.

### Differential gene expression signatures were enriched in regulatory, signaling, and bacterial infection-related immune system processes

To understand the functional composition of DE genes, we carried out pathway enrichment analysis (Supplementary Tables 14–25). Results for Gene Ontology Biological Processes showed that ~80 DE genes were enriched in ‘immune system process’ and in ‘positive regulation of response to stimulus’ at each assessed post-vaccination day. The highest enrichment was observed for the ‘cytokine–cytokine receptor interaction’ KEGG pathway. Bacterial-specific infectious disease/immune response pathways included ‘*Staphylococcus aureus* infection’ and ‘Tuberculosis’ which were both enriched in DE genes for both post-vaccination days. Several innate immune signaling pathways were enriched in DE genes including the complement and coagulation cascades, Jak-STAT signaling pathway, and IL-17 signaling pathway.

**Fig. 4:**
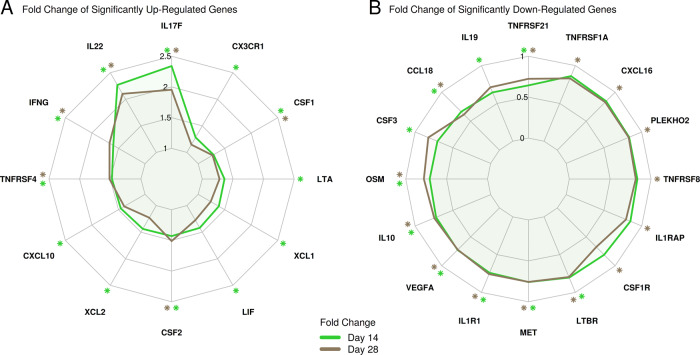
Summary of fold change responses of cytokine–cytokine interaction pathway DE genes. **a** Fold change of significantly upregulated genes. **b** Fold change of significantly downregulated genes. Significantly up- and downregulated genes are highlighted by asterisks (FDR-adjusted *P*-value < 0.05 and fold change ≥1.5).

To further assess the enrichment profile of the ‘cytokine–cytokine receptor interaction’ pathway, we visualized gene fold change responses on top of the pathway map (Supplementary Figs. 19 and 20), and contrasted DE gene responses observed for this pathway using radar plots (Fig. 4). Results showed that, on day 14 post-second vaccination, 12 genes including several cytokine-encoding genes were significantly upregulated including *IL17F* (interleukin 17F), *IL-22* (interleukin-22), *IFNG* (IFN-γ), and *CXCL10* (IP-10) (Fig. 4a), while 10 genes were significantly downregulated including *CCL18* (chemokine (C–C motif) ligand 18), *IL10* (interleukin-10), and *IL19* (interleukin-19) (Fig. 4b). On both days 14 and 28 post-vaccination, *IL-17F*, *IL-22*, *IFNG*, *TNFRSF4*, *CSF1*, and *CSF2* were significantly upregulated whereas *TNFRS21*, *CCL18*, *OSM*, *IL-10*, *VEGFA*, *ILR1*, *MET*, and *LTBR* were significantly downregulated.

**Fig. 5:**
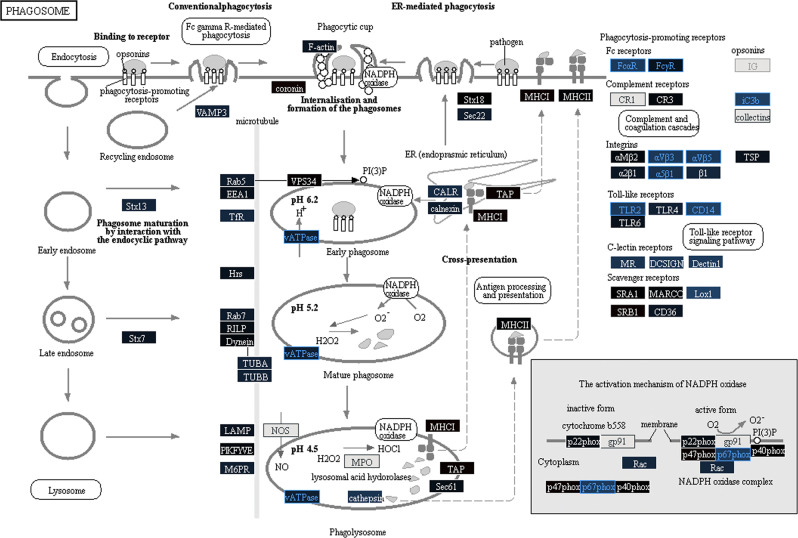
F1/V vaccine resulted in a perturbation of the phagosome pathway with many genes being downregulated compared to pre-vaccation on day 28 post-second vaccination. Node color gradient encodes fold change from pre-vaccination (for multi-gene pathway nodes the median fold change is used). In red: upregulated compared to pre-vaccination, in blue: downregulated compared to pre-vaccination. In black: fold change close to 1, in dark gray: genes filtered out due to low overall expression, light gray: gene missing database mapping, white: non-human gene. DE genes are highlighted using red (significantly upregulated) and blue (significantly downregulated) node label and border colors (FDR-adjusted *P*-value < 0.05 and fold change ≥1.5).

The phagosome pathway map (Fig. 5) showed that many of the pathway members were unaffected or downregulated from pre-vaccination. vATPase complex which is important to maintain acidification of phagosome, p67phox associated with production of reactive oxygen species in the phagosome, Fc receptors such as FcαR and FcγR, integrins such as αVβ3, αVβ5, and α5β1, C-lectin receptors such as MR, DCSIGN, and Dectin 1, TLR2, CD14, and Lox1 were downregulated, indicating reduced phagocytic activity on day 28 post-second vaccination.

### Regulatory gene expression signatures were correlated with vaccine-induced macrophage-protective antibody responses

To identify gene expression signatures that correlated with maximum change in macrophage-protective antibody responses (day 14 or 28 post-second vaccination), we carried out regularized linear regression analysis. For day-14 post-second vaccination, the best predictive model identified 16 genes, of which 56% were known to play a role in regulation of cellular processes (source: UniProt GO) (Supplementary Table 27). Scatterplots that summarize correlations between log_2_ fold changes in gene expression and maximum protective antibody responses for each gene are provided in Supplementary Fig. 37. In the day-14 post-second vaccination model, the strongest positive coefficient was observed for the *IFNG* (IFN-γ) gene and the strongest negative coefficient was observed for ENSG00000225107 encoding for one of the long noncoding RNAs (LincRNA), which may play important roles in the regulation of gene expression and nuclear organization^16^ (Supplementary Table 27).

### Transcriptomics results were correlated with vaccine-induced T-cell and cytokine responses

**Fig. 6:**
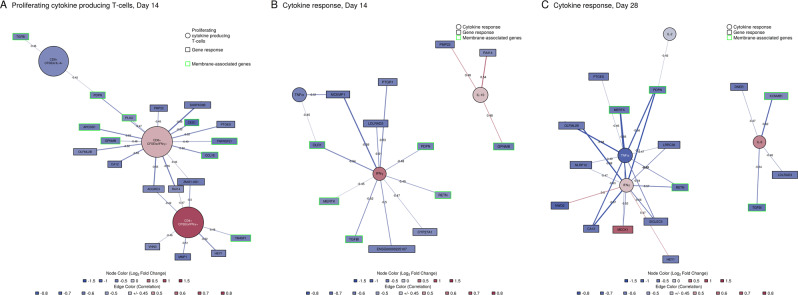
Combination of genes that were correlated with changes in T-cell proliferation or serum cytokine concentration. Node color gradient encodes fold change from pre-vaccination. In red: upregulated compared to pre-vaccination (in blue: downregulated compared to pre-vaccination). Edges represent correlation between genes and T-cell or cytokine responses based on the first canonical variate pairs are shown (in blue: negative, in red: postive correlation, only correlations >0.45 were retained). Edge thickness is scaled by increasing correlation. In green border color: genes encoding for plasma membrane or extracellular space proteins.

Next, we assessed changes in gene expression signatures that were predictive of T-cell or cytokine responses using regularized canonical correlation analysis (Supplementary Table 28). The strongest first canonical correlation was observed between gene responses and proliferating CD8^+^/CFSE^lo^/IFN-γ^+^, CD4^+^/CFSE^lo^/IFN-γ^+^, and CD8^+^/CFSE^lo^/IL-4^+^ T cells on day 14 post-second vaccination (Fig. 6a), followed by changes in day 14 post-second vaccination IFN-γ, TNF-α, and IL-10 serum cytokine concentrations (Fig. 6b), and day 28 post-second vaccination IFN-γ, TNF-α, IL-2, and IL-6 serum cytokine changes (Fig. 6c). Associations with proliferating T cells on day 28 post-second vaccination did not reach a sufficient cross-validation score (score < 0.5). Associated genes were downregulated with the exception of *MEOX1*, a transcription factor regulating vascular cell proliferation, whose expression increased with increasing IFN-γ levels in serum on day 14 (Fig. 6b). The most frequent pathway annotation for associated genes was tissue development for T cells and biological adhesion for cytokines (source: GO Biological Processes). In addition, multiple associated genes encoded for proteins that are part of the plasma membrane or extracellular space (source: GO Biological Component) (Fig. 6, highlighted in green). On day 14 post-second vaccination, the increase in CD8^+^/CFSE^lo^/IFN-γ^+^ was associated with the largest number of these genes including *CCL18* (a lymphocyte-attracting chemotactic factor) and *TNFRSF21* (T-cell regulator).

## Discussion

Studies in animals and humans show that subunit vaccines containing virulent antigens of Yp induce good antibody responses.^11,12,17^ Although the direct mechanism of how antibodies protect against Yp is not completely understood, there is evidence that suggests that antibodies to V antigen of Yp opsonize Yp,^18,19^ suppress Yop translocation^20^ and protect macrophages from apoptosis.^17,21,22^ Sera from subjects immunized with a subunit vaccine containing the F1 capsular and the V antigens in 20% alhydrogel protected mice from the lethal challenge of Yp.^11^ Animal challenge studies require containment labs and will not be practical for all future plague vaccine trials. Therefore, a new assay that measures the ability of vaccine-induced antibodies to protect lysis of macrophages by a recombinant Yersinia expressing V antigen has been developed.^17,22^ Yptb appears to be better than Yp for this assay because Yptb activates apoptosis much more effectively than Yp in vitro.^23^ We tested this assay for the first time on human sera using pre- and post-vaccination samples from a recent clinical trial where we used Flagellin/F1/V subunit vaccine.^12^ Flagellin/F1/V is composed of a single protein incorporating the amino and carboxyl regions of flagellin from *Salmonella enteritidis* and the two antigenic peptides from Yp.^13^ Flagellin is a potent inducer of innate immunity, mainly because of its direct interaction with toll-like receptor 5.^24,25^ This new flagellin adjuvanted F1/V vaccine was shown to induce good antibody response in a recent clinical trial. Now, we demonstrate that post vaccination sera from individuals vaccinated with Flagellin/F1/V vaccine protect macrophages from apoptosis caused by infection of Yptb expressing V antigen (median increase of 75% in protective levels on day 42). It has been shown that the extent to which caspase 3 levels decrease was significantly associated with survival of NHP after aerosol challenge with Yp.^17,22^ Using the survival models as described by Welkos et al.^22^ for NHP for day 70 post-challenge, the NHP survival rate would be estimated at ~73% assuming protective levels as observed on day 56 (28 days post-second vaccination) in our study. Our results indicated that there was a significant correlation between caspase-3 values and the anti-V ELISA IgG titer but not against anti-F1 ELISA. This is because the recombinant Yptb that we used in our assay to measure macrophage-protective antibody responses expresses V but not F antigen; therefore, macrophage protection is likely rendered by antibodies against V antigen.

The roles of T-cell immunity in protection against Yp are increasingly recognized. Treatment of mice with exogenous IFN-γ and TNF-α inhibits multiplication of Yersinia and protects against lethal challenge.^26^ Similarly, neutralization of endogenous IFN-γ and TNF-α make mice more vulnerable to pneumonic plague.^6^ In addition, passive transfer of cells from *Yersinia enterocolitica* convalescent mice partially protects naïve mice against Yp challenge.^27,28^ Further studies identified that CD4 and CD8 T cells are the main cell types conferring Yp immunity in mice, with CD4 T cells improving the cytotoxic effect of CD8 T cells, but not having a significant effect by themselves.^29^ Induction of T-cell immunity may depend on the adjuvants or vaccine types.^30–32^ SA-4-1BBL adjuvant improved Th1 immune response induced by alum-adjuvanted F1/V subunit vaccine.^30^ In a recent murine study, flagellin adjuvanted subunit vaccine has been shown to induce both humoral and cell-mediated immunity.^33^ Multiple antigen peptide constructs of V antigen increases lymphoproliferative responses in mice, with highest levels of Th1 and Th17 cytokines in culture supernatants.^34^ IL-17 appears to mediate enhanced mucosal protection, and neutralization of vaccine-induced IL-17 decreases the survival of mice following challenge with Yp.^32^ Our results similarly showed that Flagellin/F1/V vaccine significantly increased the number of proliferating and IL-10 or IL-4 producing CD4 T cells. In addition, we showed that this flagellin-adjuvanted F1/V vaccination induced polyfunctional CD4 T cells, which proliferate and produce both IFN-γ and TNF-α. It has previously been shown that neutralization of IFN-γ and TNF-α interferes with the capacity of anti-V antibodies to decrease bacterial burden or increase survival in murine model.^9^ While we found no significant correlation between IFN-γ-producing CD4 T cell and the antibody response, we did find a significant negative correlation for IL-6-producing CD4 T cell in our study. IL-6 is a proinflammatory cytokine produced mainly by monocytes^35^ to promote Th2 and Th17 differentiation as well as enhance antibody production.^36,37^ In our study, the IL-6 levels in supernatants of cultures containing post-vaccination PBMC were not different from the pre-vaccination levels. Activated lymphocytes also produce IL-6 which has autocrine function, mainly inducing Th2 responses.^38^ In our study, the strongest negative correlation was between changes in IL-6-producing CD4^+^ T cells and macrophage protection on day 14 post-second vaccination (*P* = 0.026, Spearman *r* = −0.57) suggesting that IL-6 from CD4 T cells may not be a major factor to induce macrophage protective antibody responses. However, because we did not measure IL-6 produced by other major specific cell types such as monocytes, our result may not show the overall effects of IL-6 on macrophage protective responses.

Unlike infection with attenuated strains of Yp, infection of mice and brown Norway rats with wild-type Yp suppresses several genes including genes for antigen processing and presentation, T-cell receptor (TCR)-signaling, NF-kB signaling, and natural killer (NK) cell function.^39^ The length of time after infection with Yp or stage of disease affects gene expression, with genes for Th1 immune response induced only at the initial or the recovery stage.^39,40^ To our knowledge, there are no host transcriptomics studies following vaccination with F1/V subunit vaccine. In this study using RNA-seq of stimulated PBMC, we showed that F1/V vaccination led to changes in the expression of several chemokine and cytokine genes. Genes encoding for IFN-γ, IL-22, IL-17F, CSF1, CSF2, and TNFRSF4 were significantly upregulated whereas genes encoding for IL-10, TNFRSF21, CCL19, OSM, VEGFA, ILR1, MET, and LTBR were downregulated on both days 14 and 28 post-vaccination. IFN-γ activates macrophages and increases their ability to kill intracellular bacteria, including Yp.^26^ All the upregulated genes are associated with macrophage or T-cell function, which may be relevant for the control of Yp. Upregulation of IL-17F and IL-22 genes was previously seen in mice vaccinated with live attenuated strain of Yp.^32^ IL-17-producing T cells often produce IL-22 which acts synergistically with IL-17 to enhance the expression of anti-microbial peptides and promote inflammation.^41–43^ CSF1 and CSF2 are important for monocyte maturation and dendritic cell homeostasis.^44,45^ TNFRSF4 is a member of TNF receptor superfamily and provides costimulatory signals to activate T cells following TCR ligation.^46^ Most of the downregulated genes except LTBR, IL1R1, and CCL19 encode for ligands or receptors associated with inhibitory signals and their inhibition likely enhances the functions of antigen-presenting cells, Th1, or Th17 immunity. IL-10 is a known immunomodulator that inhibits Th1 immunity.^47^ NFRSF21 is an orphan TNF receptor superfamily member which belongs to a subgroup called death receptors and its knockout or downregulation enhances antibody response.^48^ OSM suppresses activation of IL-17 regulation in CD4 T cells.^49^ VEGFA is a proangiogenic factor but inhibits the differentiation and function of T cells.^50^ MET modulates dendritic cells to play an immunosuppressive role.^51^ The reasons why LTBR which is important for homeostasis of dendritic cells,^52^ IL1R1 which is important for IL-17 generation, and IL-1 function^53^ and CCL19 which enhances Th1 immunity^54^ are down regulated are not clear.

Furthermore, genes associated with phagocytic activity were downregulated after F1/V vaccination. In murine studies with Yp infection, F1 and V antigen help Yp avoid phagocytosis. F1 and V antigens acts in concert with plasmid encoded type III secretory system to make Yp highly resistant to phagocytosis.^55,56^ F1, unlike the plasmid encoded type III secretary proteins, only affects the phagocytosis of Yp without inhibiting the general phagocytic ability.^55^ F1 forms a capsule around the bacterium to increase its resistance to phagocytosis, presumably by preventing interactions with yet unknown phagocytic cell receptors.^55,56^ It is not clear why phagocytic genes are downregulated following vaccination with F1/V vaccine and this needs to be investigated further using studies that measure the function of phagocytic cells at different times post F1/V vaccination. This study has the following limitations: (1) the study did not measure the long-term immunity induced by F1/V vaccine beyond 28 days post second-vaccination, (2) gene expression markers associated with immunity induced by the flagellin adjuvanted F1/V vaccine have not been validate by qPCR because of inadequate clinical samples for additional assay, (3) the caspase 3 assay was based on a murine macrophage cell line rather than a human cell line model, and (4) recombinant Yptb used as part of the caspase-3 assay only expressed the V antigen and not the F1 antigen.

To our knowledge, this is the first study that used RNA-Seq to comprehensively assess transcriptome-wide gene expression changes following vaccination, and more particularly, gene expression markers associated with antibody and T-cell responses induced by F1/V plague vaccine to date. Increased expression of the IFNG gene and decreased expression of a lincRNA (ENSG00000225107), was most strongly associated with day 14 macrophage-protective antibody responses. The importance of lincRNA in antibody-mediated response is not known but the IFNG gene which encodes for the IFN-γ cytokine is essential for optimal antibody response.^57,58^ Cytokine and T-cell responses were correlated with genes encoding for tissue development, biological adhesion, and plasma membrane or extracellular space-associated proteins indicating that cell interaction/attachment was modulated on the expression level.

In conclusion, our study indicated that Flagellin/F1/V vaccine induced protective antibody responses and marked T-cell immunity that were correlated with unique gene expression signatures. These signatures serve as potential biomarker candidates for predicting protective immune responses in future plague vaccine trials.

## Methods

### Study design and samples

Stored samples from a recently completed clinical trial (DMID 08-0066) were used.^12^ DMID 08-0066 was a phase I, randomized, double blind, placebo controlled, dose-escalation trial to evaluate flagellin/F1/V, a plague vaccine, in healthy adults. In the DMID 08-0066 clinical trial, volunteers were vaccinated with two doses of 1, 3, 6, or 10 µg of flagellin-adjuvanted F1/V subunit plague vaccine 28 days apart. Sera and PBMCs collected at three time points (pre-vaccination, and days 14 and 28 post-second dose) from volunteers vaccinated with 6 μg and 10 μg of flagellin-adjuvanted F1/V vaccine were used in this study. These two post vaccination time points from the high-dose groups were selected because of high antibody responses.^12^ Clinical samples from these two timepoints, sera stored at −80 °C and PBMC stored in liquid nitrogen, were used in this study. Supplementary Fig. 1 summarizes the number of subjects by treatment group and assay type used in this study. As part of this study, DMID 15-0104, additional blood samples were collected from seven volunteers who received 6 μg or 10 μg of Flagellin/F1/V vaccine in the 08-0066 trial. Sera and PBMCs from these additional blood samples were used to optimize immunological assays for this study.

### Identify macrophage-protective function of antibody responses

Antibodies to V antigen were higher than antibodies to F1 antigens in the stored samples we have used.^12^ A caspase-3 assay which has previously been shown to measure protective capacity of antibody responses to V antigen in mice and NHP was used in this study.^17,22^ The assay had never been tested on human samples and therefore, we optimized methods used in mice and primates.^17,22^ Briefly, suspensions containing recombinant Yptb-expressing V antigen were pretreated with heat-inactivated sera or a positive control monoclonal antibody obtained from BEI resources (NIAID, NIH, NR-31022) for 5 min at 38 °C in an orbital shaker at a speed of 225 rpm. The pretreated bacteria were added into 96-well plates containing J774 (ATCC TIB-67) macrophage cell line (1 × 10^3^ cells per well) and incubated at 37 °C. Then, the levels of caspase-3 in the culture supernatants were quantified 1.5 h later using a caspase-3 assay kit (EnzChek, Life Technologies). Caspase-3 levels were assessed as the mean of triplicate measurements. Normalization was done by dividing the mean caspase-3 values of clinical samples by caspase-3 values obtained in serum-free infected controls. Higher normalized caspase-3 values indicated lower macrophage protection and vice versa. For a more intuitive interpretation of macrophage-protective function of antibodies, the inverse of the normalized caspase-3 level variable was analyzed.

### Assay to measure vaccine-induced T-cell responses

In the initial experiments, we optimized conditions for stimulation including antigen type, antigen concentration, and duration of stimulation. An optimized carboxyfluorescein succinimidyl ester (CFSE)-based flow cytometric assay was used to measure T-cell responses in pre-vaccination and post-vaccination PBMC from volunteers who received 6 or 10 μg of flagellin-adjuvanted F1/V vaccine. The CFSE assay was performed as described previously.^59^ Briefly, PBMCs were stimulated with optimal antigen concentrations. On day 7, cells were stained for surface markers such as CD3, CD4, and CD8 as well as intracellular cytokines such as IFN-γ, IL-2, TNF-α, IL-4, IL-6, IL-10. Flow-cytometric acquisition was performed by using a multi-color BD FACSCanto II instrument, and analyses were done using the CellQuest and FlowJo (Tree Star) software. A minimum of 10,000 events were acquired. Cells with decreased CFSE fluorescence were considered as proliferating cells. The absolute numbers of effector CD4^+^ T and CD8^+^ T cells were calculated by multiplying the total number of viable cells recovered by the percentage of the specific T-cell subset detected by flow-cytometric analysis. Stimulation indices used for analysis were calculated by dividing the absolute number of proliferating and cytokine producing T cells for cultures stimulated with antigens by the absolute number of the corresponding T-cell population for medium-rested controls (see Supplementary Text for additional details).

### Assay to measure cytokine concentrations in supernatants

Culture supernatants from the optimized CFSE-based assay were used for measurement of cytokines using a Th1/Th2/Th17 cytokine bead array kit (Becton Dickinson). Stimulation indices used for analysis were calculated by dividing the concentration of cytokines (pg/ml) in supernatants from cultures stimulated with antigen by corresponding concentrations for cultures rested in medium.

### RNA sequencing and data processing

PBMC were stimulated with F1/V vaccine antigen for 24 h before RNA was extracted. A short stimulation time (i.e., 24 h) was selected to avoid missing early gene expression changes. Stimulation for 18–48 h has been shown to identify transcriptomics responses associated with immune responses.^60^ RNA-Seq was carried out using single-end 50 base read sequencing using an Illumina HiSeq3000 sequencer. Forty-one libraries were multiplexed across four sequencing lanes with targeted coverage of 39 million reads per library. Human reference genome assembly (GRCh38), gene models, and associated gene annotation information were obtained from the ENSEMBL database (Version 90). The genomic reference was built by merging all human chromosomes except for X and Y chromosomes (to avoid gender-specific effects). Adapter sequences and low-quality 5′ ending sequences were removed from raw sequencing reads using the Trimmomatic software (Version 0.36). Following adapter removal, sequence reads were aligned to the index of known human rRNAs and tRNAs using Bowtie2 with its local alignment option. Filtered reads were aligned to the reference transcriptome/genome using the latest version of HISAT2 splice-aware sequence aligner (Version 2.1.0). For each sample, the quality of reference alignments was evaluated using the RSeQC software (Version 2.6.4). Gene expression quantification was conducted on the gene level using Subread v1.5.3 counting mapped paired-reads to obtain fragment counts per gene (Supplementary Dataset 1, see Supplementary Text for additional details).

### Statistical analysis

Unless otherwise specified, hypothesis tests were carried out in a two-sided manner using an individual alpha level of 0.05.

### Macrophage-protective antibodies, proliferating and cytokine-producing T-cell analysis

Statistically significant changes for each post-vaccination day compared to pre-vaccination measurements were assessed using a two-sided Wilcoxon signed-rank test. The 95% confidence interval (CI) of the median fold change was obtained using the bootstrap method using 1000 replicates for each visit.

### RNA-seq analyses

Systematic sample differences in read counts were corrected for using TMM normalization.^61^ Negative binomial models as implemented in edgeR (Version 3.18.1)^62^ were used to identify genes that were DE from pre-vaccination adjusting for paired samples (subject effect). Genes with an FDR-adjusted *P*-value ≤ 0.05 (p.adjust R function) and a fold change difference of ≥1.5-fold (in either direction) were deemed to be DE. Enrichment analysis for KEGG (v83)^63^ and MSigDB (Version 6.1)^64^ pathways was carried out using the goseq R package (v1.28.1)^65^ accounting for gene length bias (FDR-adjusted *P*-value ≤ 0.01). TMM-normalized log2 fragment counts per million (LCPM) as implemented in edgeR (Supplementary Dataset 2) were used as input for calculating log2 fold changes used for regularized linear regression analysis (glmnet Version 2.0–13 R package) to identify gene responses that best predicted peak log2 changes in protective levels of antibodies (based on inverse Caspase-3 levels). The input set included genes with an average absolute fold change of 1.5 on day 14 and/or 28 post-second vaccination. Leave-one-out cross-validation was used to select optimal regularization parameters (see Supplementary Text for additional details).

### Ethics statement

The trial was approved by the Saint Louis University institutional review board and is registered on ClinicalTrials.gov (No. NCT01381744). All subjects provided written informed consent prior to initiation of study procedures and future use of clinical samples.

## Supplementary information

Supplementary.pdf

## Data Availability

Data that support the findings of this study have been deposited in GEO (Accession GSE136878).
