# Supplementary material for: Flagellin adjuvanted F1/V subunit plague vaccine induces T cell and functional antibody responses with unique gene signatures
Source: NPJ Vaccines. 2020 Jan 23;5:6. doi: 10.1038/s41541-020-0156-y (PMC6978331; doi:10.1038/s41541-020-0156-y)
Supplement: Supplementary file 1 — Supplementary.pdf [file 41541_2020_156_MOESM1_ESM.pdf]

Supplementary Text "Flagelin adjuvanted F1V subunit plague vaccine induces T cell and functional antibody responses with unique gene signatures"

August 1, 2019

Contents

1 Introduction **9**

2 Supplemental methods **9**

2.1 Analysis Cohort . . . 9

2.2 Antibody, T-cell, and Cytokine Assays . . . 9

2.2.1 Derived Variables . . . 10

2.2.2 Antibody, T-cell, and Cytokine Assays Analysis . . . 11

2.3 RNA-Seq . . . 11

2.3.1 RNA-Seq Experiment, Processing, Data QC and Filtering . . . 11

2.3.2 RNA-Seq Data Analysis . . . 13

2.4 Software . . . 15

3 Supplemental Results **16**

3.1 Antibody, T-cell, and Cytokine Results . . . 16

3.2 Transcriptomics results . . . 16

List of Figures

Supplementary Figure 1 Analysis population consort flow diagram 19
Supplementary Figure 2 Scatterplots that summarize the correlation between log_2 fold change in inverse Caspase-3 levels and log_2 fold change in ELISA IgG for F1 antigen . . 20
Supplementary Figure 3 Scatterplots that summarize the correlation between log_2 fold change in inverse Caspase-3 levels and log_2 fold change in ELISA IgG for V antigen . . . 21
Supplementary Figure 4 Scatterplots that summarize the correlation between log_2 fold change in inverse Caspase-3 levels and log_2 fold change in the stimulation index of proliferating CD4+/CFSElo/IL-6+ T-cells 22
Supplementary Figure 5 Scatterplots that summarize the correlation between log_2 fold change in inverse Caspase-3 levels and log_2 fold change in the stimulation index of proliferating CD4+/CFSElo/IFNγ+ T-cells 23
Supplementary Figure 6 Boxplots of log_2 counts per million before TMM normalization (RNA-Seq). . 24
Supplementary Figure 7 Boxplots of log_2 counts per million after TMM normalization (RNA-Seq). . . 25
Supplementary Figure 8 Empirical cumulative distribution function plots of log_2 counts per million before and after TMM normalization (RNA-Seq). 26
Supplementary Figure 9 Spearman correlation heatmap (RNA-Seq). 27
Supplementary Figure 10 PCA biplots (RNA-Seq). 28
Supplementary Figure 11 Non-metric multidimensional scaling biplots (RNA-Seq) 29
Supplementary Figure 12 Hierarchical clustering plots (RNA-Seq). 30
Supplementary Figure 13 Reverse empirical cumulative distribution function plots to assess lowly-expressed gene cut off (RNA-Seq). 31
Supplementary Figure 14 Reverse empirical cumulative distribution function plots to assess fold change cut off (RNA-Seq). 31
Supplementary Figure 15 Volcano and MA plots (RNA-Seq) 32
Supplementary Figure 16 Pathway map - Amoebiasis (Day 28 post-second vaccination) 33
Supplementary Figure 17 Pathway map - Complement and coagulation cascades (Day 14 post-second vaccination) 34
Supplementary Figure 18 Pathway map - Complement and coagulation cascades (Day 28 post-second vaccination) 35
Supplementary Figure 19 Pathway map - Cytokine-cytokine receptor interaction (Day 14 post-second vaccination) 36
Supplementary Figure 20 Pathway map - Cytokine-cytokine receptor interaction (Day 28 post-second vaccination) 37
Supplementary Figure 21 Pathway map - Fluid shear stress and atherosclerosis (Day 14 post-second vaccination) 38
Supplementary Figure 22 Pathway map - Fluid shear stress and atherosclerosis (Day 28 post-second vaccination) 39
Supplementary Figure 23 Pathway map - Hematopoietic cell lineage (Day 28 post-second vaccination) . 40

Supplementary Figure 24	Pathway map - IL-17 signaling pathway (Day 28 post-second vaccination)	41
Supplementary Figure 25	Pathway map - Jak-STAT signaling pathway (Day 14 post-second vaccination)	42
Supplementary Figure 26	Pathway map - Osteoclast differentiation (Day 14 post-second vaccination)	43
Supplementary Figure 27	Pathway map - Osteoclast differentiation (Day 28 post-second vaccination)	44
Supplementary Figure 28	Pathway map - Phagosome (Day 28 post-second vaccination)	45
Supplementary Figure 29	Pathway map - Proteoglycans in cancer (Day 14 post-second vaccination)	46
Supplementary Figure 30	Pathway map - Proteoglycans in cancer (Day 28 post-second vaccination)	47
Supplementary Figure 31	Pathway map - Rheumatoid arthritis (Day 28 post-second vaccination)	48
Supplementary Figure 32	Pathway map - Staphylococcus aureus infection (Day 14 post-second vaccination)	49
Supplementary Figure 33	Pathway map - Staphylococcus aureus infection (Day 28 post-second vaccination)	50
Supplementary Figure 34	Pathway map - Tuberculosis (Day 14 post-second vaccination)	51
Supplementary Figure 35	Pathway map - Tuberculosis (Day 28 post-second vaccination)	52
Supplementary Figure 36	Pathway enrichment Venn diagrams (RNA-Seq)	53
Supplementary Figure 37	Scatterplots that summarize the correlation between log_2 Inverse Caspase-3 Levels and log_2 fold change response for genes identified using regularized linear regression (RNA-Seq, Day 14 post-second vaccination)	54
Supplementary Figure 38	Canonical correlation plots (RNA-Seq/(RNA-Seq, Day 14 post-second vaccination))	55
Supplementary Figure 39	Canonical correlation plots (RNA-Seq/(RNA-Seq, Day 14 post-second vaccination))	56
Supplementary Figure 40	Canonical correlation plots (RNA-Seq/(RNA-Seq, Day 28 post-second vaccination))	57

List of Tables

Supplementary Table 1 Summary of categorical demographic and baseline characteristics for the immunogenicity analysis population . . . 58
Supplementary Table 2 Summary of age demographic characteristics for the immunogenicity analysis population . . . 58
Supplementary Table 3 Summary of categorical demographic and baseline characteristics for the transcriptomics analysis population . . . 59
Supplementary Table 4 Summary of age demographic characteristics for the transcriptomics analysis population . . . 59
Supplementary Table 5 Summaries of inverse normalized anti-V-antigen antibody caspase-3 levels by study visit day and treatment group . . . 60
Supplementary Table 6 Summaries of the fold change in inverse normalized anti-V-antigen antibody caspase-3 levels by study visit day and treatment group . . . 61
Supplementary Table 7 Spearman correlation between fold change in ELISA IgG and change in protective antibody response . . . 62
Supplementary Table 8 Wilcoxon Signed Rank Test (Proliferating and cytokine producing T cell and cytokine responses, F1/V 10ug/ml) . . . 63
Supplementary Table 9 Spearman correlation between fold change in proliferating and cytokine producing CD4+ T cell stimulation index and change in protective antibody response 64
Supplementary Table 10 Genes differentially expressed at Day 14 post-second vaccination compared to Day 0 (RNA-Seq). . . . 65
Supplementary Table 11 Genes differentially expressed at Day 28 post-second vaccination compared to Day 0 (RNA-Seq). . . . 85
Supplementary Table 12 KEGG Pathways enriched in differentially expressed genes at Day 14 post-second vaccination (RNA-Seq) . . . 103
Supplementary Table 13 MSigDB Reactome Pathways enriched in differentially expressed genes at Day 14 post-second vaccination (RNA-Seq) . . . 103
Supplementary Table 14 MSigDB Immunologic Signature Sets enriched in differentially expressed genes at Day 14 post-second vaccination (RNA-Seq) . . . 103
Supplementary Table 15 GO Biological Processes enriched in differentially expressed genes at Day 14 post-second vaccination (RNA-Seq) . . . 105
Supplementary Table 16 GO Cellular Components enriched in differentially expressed genes at Day 14 post-second vaccination (RNA-Seq) . . . 107
Supplementary Table 17 GO Molecular Functions enriched in differentially expressed genes at Day 14 post-second vaccination (RNA-Seq) . . . 107
Supplementary Table 18 KEGG Pathways enriched in differentially expressed genes at Day 28 post-second vaccination (RNA-Seq) . . . 107
Supplementary Table 19 MSigDB Reactome Pathways enriched in differentially expressed genes at Day 28 post-second vaccination (RNA-Seq) . . . 108

Supplementary Table 20 MSigDB Immunologic Signature Sets enriched in differentially expressed genes at Day 28 post-second vaccination (RNA-Seq) 108
Supplementary Table 21 GO Biological Processes enriched in differentially expressed genes at Day 28 post-second vaccination (RNA-Seq) 110
Supplementary Table 22 GO Cellular Components enriched in differentially expressed genes at Day 28 post-second vaccination (RNA-Seq) 112
Supplementary Table 23 GO Molecular Functions enriched in differentially expressed genes at Day 28 post-second vaccination (RNA-Seq) 112
Supplementary Table 24 Combination of genes correlated with log_2 transformed inverse Caspase-3 levels (RNA-Seq, Day 14 post-second vaccination) 113
Supplementary Table 25 Regularized canonical correlation analysis model summary statistics (RNA-Seq) 114
Supplementary Table 26 Number of excluded genes by gene type (RNA-Seq) 115
Supplementary Table 27 Overview of gene sets used for the enrichment analysis (RNA-Seq). 115
Supplementary Table 28 Summary human reference genome alignment statistics (RNA-Seq) 115
Supplementary Table 29 Summary human reference gene model alignment statistics (RNA-Seq) 115
Supplementary Table 30 Number of genes that passed the low expression cut off (RNA-Seq) 116
Supplementary Table 31 Regularized linear logistic regression model summary statistics (RNA-Seq) . . 117
Supplementary Table 32 List of R packages and versions used for the analyses presented in this report. . 117

Abbreviations

AE	Adverse Event
CDF	Cumulative distribution function
CFR	Code of Federal Regulations
CFSE	Carboxyfluorescein succinimidyl ester
CI	Confidence Interval
CIOMS	Council for International Organizations of Medical Sciences
CMI	Cell-Mediated Immunity
CRF	Case Report Form
DA	Differentially Abundant
DE	Differentially Expressed
DHHS	Department of Health and Human Services
DMID	Division of Microbiology and Infectious Diseases, NIAID, NIH, DHHS
DNA	Deoxyribonucleic Acid
DSMB	Data and Safety Monitoring Board
FDA	Food and Drug Administration
FDR	False Discovery Rate
FWA	Federalwide Assurance
GCP	Good Clinical Practice
GTF	Gene Transfer Format
ICF	Informed Consent Form
ICH	International Council on Harmonisation
IFN	Interferon
IgG	Immunoglobulin G
IGL	Immunoglobulin Lambda
IL	Interleukin
IRB	Institutional Review Board
ISM	Independent Safety Monitor
JAMA	Journal of the American Medical Association
KEGG	Kyoto Encyclopedia of Genes and Genomes
LCPM	Log2 Counts Per Million
LFC	Log2 Fold Change
MEDRA	Medical Dictionary for Regulatory Activities
MOP	Manual of Procedures
MSigDB	Molecular Signatures Database
MOI	Multiplicity of Infection

mt-rRNAs	Mitochondrial rRNAs
mt-tRNAs	Mitochondrial tRNAs
N	Number (typically refers to subjects)
NEJM	New England Journal of Medicine
NIAID	National Institute of Allergy and Infectious Diseases, NIH, DHHS
NIH	National Institutes of Health
NHP	Nonhuman primates
OCRA	Office of Clinical Research Affairs, DMID, NIAID, NIH, DHHS
OHRP	Office for Human Research Protections
ORA	Office of Regulatory Affairs, DMID, NIAID, NIH, DHHS
PBMC	Peripheral Blood Mononuclear Cells
PBMC	Peripheral Blood Mononuclear Cells
PCA	Principal component analysis
PCTC	Proliferating and Cytokine Producing T Cells
PHI	Personal Health Information
PI	Principal Investigator
RCCA	Regularized Canonical Correlation Analysis
RCDF	Reverse cumulative distribution function
rYPt	recombinant Yersinia pseudotuberculosis
RNA	Ribonucleic Acid
SAE	Serious Adverse Event
SDCC	Statistical and Data Coordinating Center
SMC	Safety Monitoring Committee
SAP	Statistical Analysis Plan
SOC	System Organ Class
SOP	Standard Operating Procedure
TMM	Trimmed Mean of M-values
TNF	Tumor Necrosis Factor
US	United States
VTEU	Vaccine and Treatment Evaluation Unit
WHO	World Health Organization
Yp	Yersinia pestis

Copyright Notice:

The KEGG image files and limited text-based data summary files (**collectively**, the "‘KEGG Data Snapshots"’) provided herein are copyright ©Kanehisa Laboratories. All rights reserved. By accepting the KEGG Data Snapshots, following terms shall be automatically accepted without limitation:

– Redistribution of the KEGG Data Snapshots is strictly prohibited.

– KEGG Data Snapshots may not be used outside of their intended purpose as part of a summary of a data analysis. For example, the data may not be stored or assembled in order to create an internal database, even for personal use.

– A limited number of images included in the KEGG Data Snapshots may be published in printed media with appropriate attributes as is standard in academia.

– The rights granted hereunder are non-transferable.

1 Introduction

This supplemental text provides supporting information for the manuscript entitled "Flagelin adjuvanted F1V subunit plague vaccine induces T cell and functional antibody responses with unique gene signatures". Serum and PBMC samples were tested for three time points (pre-vaccination, and days 14 and 28 post-second dose) from participants vaccinated with 6 mcg (n=8) and 10 mcg (n=8) of flagellin-adjuvanted F1/V vaccine (Supplementary Figure 1). Pre- and post-vaccination assessments included protective function of antibodies (caspase-3), proliferating and cytokine-producing T cells, cytokines, and PBMC transcriptional profiles. Transcriptomics analysis included the identification of genes that differed in their response from pre-vaccination, co-expressed gene clusters, and enriched pathways. Transcriptomics response profiles were correlated with each of the three other immunogenicity assays. In addition, correlations between the protective function of antibodies and IgG ELISA results were evaluated.

2 Supplemental methods

2.1 Analysis Cohort

Three subjects in the analysis cohort consented to de-identified future use. Categorical demographic and baseline characteristics for the remaining 13 subjects were summarized for all subjects in the analysis cohort (Supplementary Table 3). Ten subjects (n=10, 77%) were male and three were female (n=3, 23%). All were of non-Hispanic ethnicity (n=13, 100%). Ten subjects (n=10, 77%) classified themselves as white race, one subject classified themself as American Indian or Alaska Native race (n=1, 8%), one as Asian (n=1, 8%), and one as Black or African American (n=1, 8%). Continuous demographic and baseline characteristics for the 13 subjects are summarized in Supplementary Table 4. The mean age for subjects was 30.3 years (range: 21-42) with a median age of 29 years.

2.2 Antibody, T-cell, and Cytokine Assays

Macrophage-protective function of antibody responses Anti-V-antigen antibody caspase-3 assay was used to measure the function of antibodies to protect macrophages from the cytotoxicity of Yersinia. Recombinant Yersinia pseudotuberculosis expressing V antigen was pretreated with serum. The pretreated bacteria was added into a commercially available macrophage cell line in 96 well plates. Caspase-3 in the culture supernatants was quantified 1.5 hours later using a caspase-3 assay kit (EnzChek).

Optimized CFSE-based flow cytometric assay Three concentrations of killed recombinant Y. pseudotuberculosis expressing V antigens (20) and four concentrations of mixed F1 and V *Y. pestis* antigens were used to identify the optimal antigen concentration for stimulation of CMI. At different time points (days 3-10), cells were stained for surface markers such as CD3 and CD4, and intracellular cytokines such

as interferon (IFN)-gamma and interleukin (IL) 4. Flow-cytometric acquisition was performed by using a multi-color BD FACSCanto II instrument, and analyses was done using CellQuest and FlowJo (Tree Star) software. A minimum of 10,000 CD4+ events were to be acquired. Cells with decreased CFSE fluorescence were considered as proliferating cells. The absolute numbers of effector CD4+ T cells were calculated by multiplying the total number of viable cells recovered by the percentage of the specific T cell subset detected by flow-cytometric analysis. Stimulation index was obtained by calculating the ratio of stimulated versus unstimulated (medium rest control) T cells that both proliferated and produced cytokine as measured by flow cytometry. Mann-Whitney U tests were used to analyze differences in the expansions of CD4+ T cells in samples from vaccinated and non-vaccinated participants. Optimal antigen concentration and duration of stimulation was selected based on the highest vaccine-specific CD4+ T cell responses.

The optimized CFSE-based flow cytometric assay was then used to measure CMI in pre-vaccination and post-vaccination PBMC from participants who received 6 to 10 mcg of flagellin-adjuvanted F1/V vaccine. The CFSE-assay was performed using optimal antigen concentrations and the optimal culture duration before staining for surface markers such as CD3, CD4 and CD8 as well as intracellular cytokines such as (IFN-γ, IL-2, TNF-α, IL 4, IL-6, IL-10). Flow cytometry was performed as described above.

Cytokine assay Culture supernatants from the optimized CFSE-based assay were used for measurement of cytokines using a cytokine bead array (Becton Dickinson). This array allowed a detailed characterization of Th1, Th2 and Th17 cytokine levels, and was a powerful supplement to the results from CFSE-based assays.

2.2.1 Derived Variables

Macrophage-protective function of antibodies Caspase-3 levels were assessed as the mean of triplicate measurements. Serum-free infected controls were used for normalization. Infected control normalization was carried out by dividing mean sample caspase-3 levels by serum-free infected control caspase-3 levels. Higher normalized caspase-3 levels values indicated lower protection and vice versa. For a more intuitive interpretation of protective function of antibodies, the inverse of the normalized caspase-3 level variable was analyzed and summarized.

Stimulation index (proliferating and cytokine-producing T cells) Sample CFSE PCTC results (absolute number of T-cells) for a certain cytokine-producing T cell subset following sample antigen stimulation were divided by respective CFSE results (absolute number of T-cells) of the respective medium rested (unstimulated) T cells subset from the same sample.

Stimulation index (Th1, Th2 and Th17 cytokines) Sample cytokine bead array results (pg/mL) for a certain cytokine (IL-17A, IFN-γ, TNF, IL-10, IL-6, IL-4, and IL-2) level following sample antigen stimulation were divided by respective cytokine levels (pg/mL) of unstimulated (medium rested) results from the same sample.

Peak Responses For gene expression correlation analyses, peak protective function of antibodies (inverse of normalized caspase-3 levels) was calculated as the maximum fold change per subject at Day 14 or 28 post-second vaccination compared to Day 0.

2.2.2 Antibody, T-cell, and Cytokine Assays Analysis

The protective function of antibodies (inverse of normalized caspase-3 levels), stimulation index of proliferating and cytokine-producing T cells, and stimulation index (ratio for cytokines) was summarized using minimum, Q1, median, Q3, maximum, and 95% CI of the median. Summaries were provided by visit and study arm, as well as pooled across study arms. The 95% confidence interval (CI) of the median was obtained using the bootstrap method using 1,000 replicates for each visit and study arm combination. Similarly, fold change compared to pre-vaccination was summarized. Time trends of median fold change and associated 95% bootstrap CIs were visualized. Pre-vaccination and post-vaccination results were contrasted using boxplots. Statistically significant changes for each post-vaccination day compared to pre-vaccination measurements were assessed for combined study arms using a two-sided Wilcoxon Signed-Rank test with an individual alpha level of 0.05.

2.3 RNA-Seq

2.3.1 RNA-Seq Experiment, Processing, Data QC and Filtering

RNA-Seq Experiment RNA-Seq was carried out using single-end 50 base read sequencing using an Illumina HiSeq3000 sequencer. Fourty-one libraries were multiplexed across 4 sequencing lanes with targeted coverage of 39 million reads per library.

RNA-Seq Data Processing The latest version of the human reference genome assembly (GRCh38), gene models, and associated gene annotation information in the form of a Gene Transfer Format (GTF) file were obtained from the ENSEMBL database (Version 90). The genomic reference was built by merging all human chromosomes except for X and Y chromosomes (to avoid gender-specific effects). Sequences of known human rRNAs, rRNA pseudogenes, tRNA pseudogenes, mitochondrial rRNAs (mt-rRNAs), mitochondrial tRNAs (mt-tRNAs), and mt-rRNA pseudogenes were obtained from the Ensembl database (Version 90, August 2017) using the *biomaRt* R package (Version 2.32.1). Known human tRNA sequences

were obtained from the Ensembl database (Version 90, August 2017) using the Ensembl Perl API (Version 90). These sequences were then used to build a *Bowtie2* index of contaminant sequences. Adapter sequences and low-quality 5' ending sequences were removed from raw sequencing reads using the *Trimmomatic* software (Version 0.36). The impact of quality filtering was assessed using the *FastQC* software. Following adapter removal, sequence reads were aligned to the index of known human rRNAs and tRNAs using *Bowtie2* with its local alignment option. Reads that mapped to the index were removed, and those that did not were output to a FASTQ file for alignment to the reference genome. Filtered reads were aligned to the reference transcriptome/genome using the latest version of *HISAT2* splice-aware sequence aligner (Version 2.1.0). For each sample, the quality of reference alignments was evaluated using the *RSeQC* software (Version 2.6.4).

Gene Expression Quantification and Normalization Gene expression quality were summarized in tabular form, univariate boxplots, and multivariate starplots. Quantification was carried out on the gene level using the *featureCounts* function of the *Subread* software package (Version 1.5.3). Reads that overlapped with multiple genes or mapped to multiple locations on the reference genome were excluded. Known ribosomal, transfer, and mitochondrial RNA genes were removed from the final read count analysis dataset (Supplementary Table 26). Systematic sample differences in sequencing coverage were corrected for by calculating scaling factors for each sample using the trimmed mean of M-values (TMM) method as implemented in the R package *edgeR* (Version 3.18.1). TMM-normalized $\log_2$ counts per million (LCPM) for each gene were calculated using *edgeR*. A TMM-scaled count of 0.5 reads was added to each gene to avoid taking the $\log_2$ of zero. LCPM distributions were visualized using univariate boxplots and cumulative distribution function (CDF) plots before and after TMM-normalization.

Global Outlier and Batch Effect Detection TMM-normalized LCPM were standardized (z-score: mean=0, variance=1) and LCPM distributions across samples were inspected for outliers (potential sample mislabeling, experimental error, etc.) and systematic effects (sample ordering, batch processing, etc.) using correlation heatmaps, principal component analysis, multidimensional scaling, and hierarchical clustering.

Determination of Cut Offs for Filtering Lowly Expressed Genes To determine a suitable cutoff value for filtering lowly expressed genes, reverse cumulative distribution (RCDF) plots summarizing the percentage of genes that exceeded a certain average LCPM cutoff were plotted on the y-axis. The pre-specified cutoff of 1 LPCM (2 counts per million on the original scale) retained 12,596 genes, which was in the targeted range of 10,000 and 14,000 genes (Supplementary Table 30, Supplementary Figure 13). Genes that passed this cutoff were used as input for all downstream analyses.

2.3.2 RNA-Seq Data Analysis

Identification of Differentially Expressed Genes Negative binomial generalized linear models as implemented in *edgeR* were applied to identify DE genes after exclusion of outlying samples and lowly expressed genes. TMM-adjusted total read counts per sample were included in the models as an offset to account for systematic sample differences. For each post-first vaccination day (Day 14 and Day 28 post-second vaccination), a negative binomial model was fit to pre- and post-first vaccination read counts across subjects. The subject effects for estimating subject-specific pre-first vaccination levels were added to account for paired samples from the same subject. Effects were parameterized in the design matrix using dummy variables with one variable for each subject and an additional variable for the pre- vs. post-first vaccination effect. For vaccination effects, the value of the dummy variable was set to 1 for post-second vaccination (Day 14 or Day 28 post-second vaccination) and 0 for pre-first vaccination (Day 0) observations. For each gene, the statistical significance of the post- vs. pre-vaccination effect was evaluated using a likelihood ratio testing the following hypothesis on the $\log_2$ scale:

$$\mathbf{H_0}: \mu_{\mathbf{post-vaccination}} - \mu_{\mathbf{pre-vaccination}} = \mathbf{0}, \quad \mathbf{H_1}: \mu_{\mathbf{post-vaccination}} - \mu_{\mathbf{pre-vaccination}} \neq \mathbf{0}$$

To control for testing multiple genes, the false-discovery rate (FDR) based on the Benjamini-Hochberg procedure as implemented in the *p.adjust* R function was be applied for each model. Genes with an absolute fold change (effect size) of ≥ 1.2 compared to pre-first vaccination and FDR-adjusted p-value < 0.05 were be considered to be significantly differentially expressed (DE). Treatment effects in terms of overall fold changes and FDR-adjusted p-values were summarized for all genes that passed the low expression cut off using MA plots and Volcano plots. The fold change cutoff of 1.2 was selected based on RCDF plots summarizing the percentage of DE genes that exceeded a certain fold change cut off (Supplementary Figure 14). Overlap in DE genes between post-vaccination days was summarized using Venn diagrams, both in terms of overall numbers and separately for up/down-regulated DE genes.

Heatmap Analysis Per-subject baseline $\log_2$ fold change patterns for DE genes were visualized for each post-vaccination day (Day 14 and Day 28 post-second vaccination) using heatmaps. Genes identified as DE for any post vaccination day (Day 14 or Day 28 post-second vaccination) were included as part of this analysis. Heatmap subject and gene dendrograms were based on uncentered Pearson correlation distance and complete linkage hierarchical clustering.

2.3.2.1 Gene Clustering Analysis Unsupervised multiscale bootstrapping as implemented in the *pvclust* R package (Version 2.0.0) was carried out to identify robust clusters of genes with correlated $\log_2$ fold change responses across Day 14 and Day 28 post-second vaccination. Bootstrap resampling was based on uncentered Pearson correlation distances between $\log_2$ fold change responses. The input list of genes for this analysis included genes identified as DE for any post-vaccination day (Day 14 or Day 28 post-second vaccination). Clusters were obtained using the hierarchical complete linkage clustering

algorithm. To evaluate robustness of gene clusters, multiscale bootstrapping as implemented in the *pvclust* R package (Version 2.0.0) was carried out separately for each post-vaccination day using varying dataset sizes (0.6*N, 0.7*N, 0.8*N, 0.9*N, 1*N, 1.1*N, 1.2*N, 1.3*N, 1.4*N; where N stands for the respective dataset size). For each dataset size bin, 1,000 bootstrap samples were obtained, and ordinary and multiscale bootstrap probabilities were calculated. Clusters with a multiscale bootstrap probability $\geq$ 0.95 and maximum distance between cluster members of 0.5 (equivalent to minimum uncentered Pearson correlation of 0.5 among all members) were considered robust gene clusters.

Pathway Enrichment Analysis Pathway enrichment analysis was carried out separately for each identified DE gene set (Day 14 or Day 28 post-second vaccination vs. Day 0) using published gene set information obtained from the KEGG pathway database (Version 83.0, 09/26/2017), MSigDB (Version 6.1: Reactome Pathways and Immunologic Signatures, GO Biological Processes, GO Molecular Functions, and GO Cellular Components) (Supplementary Table 27). The analysis was conducted using the *goseq* R package (Version 1.28.0) which adjusts for gene length bias when modeling the null distribution. To compensate for testing multiple gene sets, for each of the 6 gene set and post-vaccination day combinations, p-values were adjusted using the Benjamani-Hochberg procedure to control the FDR. Gene sets with an FDR-adjusted p-value < 0.05 were considered to be significantly enriched. For significantly enriched KEGG pathways, color-coded KEGG pathway maps were generated (KEGG KGML pathway layout information Version 86.0, 04/01/2018).

Identification of Transcriptomics Responses That Best Predict Protective Antibody Response Regularized linear regression models were fit to determine gene expression $\log_2$ fold change responses that best predicted peak changes in protective levels of antibodies (based on inverse Caspase-3 levels) using the *glmnet* R package (Version 2.0-13). Separate models were fit for each post vaccination day (Day 14 and Day 28 post-second vaccination). To avoid overfitting, and to facilitate variable selection, an elastic net regularization step (combination of L1 Lasso and L2 ridge penalties) was applied. Leave-one-out cross validation was used to determine the optimum regularization parameters that minimized the model mean squared error. The input gene set included genes with an absolute mean baseline fold change in LCPM of $\geq$ 1.5 at Day 14 or Day 28 post-second vaccination. Prior to modeling, gene variable $\log_2$ fold changes were standardized (z-score: mean=0, variance=1) using the *glmnet* standardize=TRUE option. Genes selected by each model were further characterized using pathway enrichment analysis as described in the previous method section.

Identification of Transcriptomics Responses That Best Correlate With Changes in Proliferating and Cytokine Producing T-cells or Cytokine Responses Regularized canonical correlation analysis (RCCA) as implemented in the *mixOmics* R package (Version 6.1.3) was applied to maximize the inter-set correlation between transcriptomic $\log_2$ fold change gene responses and PCTC or cytokine fold change re-

sponses for the respective post-vaccination day. RCCA was performed for each post-vaccination day (Day 14 and Day 28 post-second vaccination) and assay (PCTC and cytokine measurements). Prior to modeling, PCTC and cytokine fold change variables were $\log_2$ transformed and then standardized (z-score: mean=0, variance=1). A regularization step was included to avoid overfitting and to handle collinearity within variable sets. Regularization parameters (λ_1 and λ_2) were estimated using leave-one-out cross validation (grid-search of 250,000 parameter combinations ranging from 0.000001 to 1 in 500 increments). Correlations between original variables (Pearson correlation) and corresponding canonical variates were calculated (canonical loadings). Canonical loadings were squared to obtain the percent-explained variance for each variable. Tabular and graphical summaries were presented if the model achieved a cross validation score (CV) of ≥ 0.5.

Canonical loadings for each variable set were visualized for the first two canonical variates using 2-dimensional canonical correlation circle plots. Gene and T-cell or cytokine variables for which the first two canonical variates explained less than 25% of their total variance (placed within the inner circle of the correlation circle plots) were grayed out. Variables or groups of variables that are closely positioned in these plots are positively correlated while those diametrically opposed are negatively correlated. The strength of the correlation is encoded by the distance from the center of the circle as well as by the angle between variables when viewed as vectors originating from the center. A sharp angle between variables represents a positive correlation, an obtuse angle indicates a negative correlation while a right angle is observed for zero correlation. Thus, maximum correlation is achieved when variables are closely placed together to the outer circle (or directly opposed on the outer circle). KEGG BRITE pathway hierarchy (second level) information for the top five pathway categories (in terms of number of genes that were correlated) was overlaid on top of the correlation circle plots to highlight known functional organization. Identified robust gene clusters were integrated by drawing lines between correlated genes that formed a robust gene cluster.

Genes that showed a correlation of $r > 0.5$ with the respective first canonical variate were further characterized using pathway enrichment analysis as described in the pathway enrichment method section.

2.4 Software

Data was analyzed using the *R statistical programming language* and *R Bioconductor* packages. A complete listing of R package versions are given in Supplementary Table 32. This report was generated using the *knitr* R package (Version 1.17) and LaTeX typesetting software (Version TeX Live 2012/Debian). The operating system used was *Ubuntu* (Version 16.04.2 LTS).

3 Supplemental Results

3.1 Antibody, T-cell, and Cytokine Results

Summary statistics for the protective function of antibodies against anti-V-antigen (inverse of normalized caspase-3 levels) pre-vaccination (Day 0), for each post-vaccination day (Day 14 and Day 28 post-second vaccination), and treatment group (Flagellin/F1/V 6mcg, Flagellin/F1/V 10mcg) as well as combined treatment groups are tabulated in Supplementary Table 5. Statistically signifcant Wilcoxon Signed-Rank test results to assess change for each post-vaccination day compared to pre-vaccination measurements are described in Supplementary Table 8 for proliferating and cytokine-producing T cells and for cytokines.

Correlation Between Protective Antibody Response and ELISA Results Supplementary Table 7 lists Spearman correlation results to assess correlation between log_2 fold change in protective levels of antibody based on inverse normalized caspase-3 levels and change in IgG ELISA titer as well as IgG ELISA concentration against the F1 and V antigens (this data was obtained for the parent study: DMID 08-0006). Corresponding scatterplots with trend lines are shown in Supplementary Figures 2 and 3

Correlation Between Protective Antibody Response and Proliferating and IL-6 and IFN-γ Producing CD4+ T Cells Supplementary Table 9 lists Spearman correlation results to assess correlation between log_2 fold changes in protective levels of antibody based on inverse normalized caspase-3 levels and change in T-cell stimulation index for CD4+/CFSElo/IL-6+ T-cells and CD4+/CFSElo/IFNg+ T-cells following F1/V stimulation. Corresponding scatterplots with trend lines are shown in Supplementary Figures 4 and 5

3.2 Transcriptomics results

RNA-Seq Reference Alignment Statistics On average, 35 million individual reads were mapped for each sample against the reference genome sequence, of which 79% uniquely mapped to a location on the human reference assembly (Supplementary Table 28). When reads were mapped against known gene models, on average, 20 million reads were uniquely counted in the expression quantification step. The vast majority (on average 81%) of tags (spliced reads) mapped to known[1] exonic regions followed by intronic (17%), and intergenic regions (1%) (Supplementary Table 29).

Impact of TMM Normalization The impact of between-sample normalization using the trimmed mean of M-values (TMM) method is visualized in Supplementary Figures 6 to 8. The plots summarize log_2 counts per million (LCPM) distributions for each sample before and after normalization. The medians and interquartile ranges of LCPM distributions were very similar for most of the 35 samples before and after normalization.

[1] Ensembl gene models (Version 90, August 2017)

Global Gene Expression Patterns, Confounding Effects, Outlying Samples Principal component analysis (PCA), non-metric multidimensional scaling, hierarchical clustering, and heatmap results based on LCPM gene variables are shown in Supplementary Figures 9 to 12. Sample SAM01 showed an outlying profile in the PCA analysis (Supplementary Figure 10). However, as a strong outlying signal was not consistently observed across all methods, it was decided to keep the sample as part of the analysis. No batch effects were observed.

Identification of Differentially Expressed Genes MA and Volcano plots are provided in Supplementary Figure 15. Differentially expressed (DE) genes for each post-vaccination day comparison (Day 14 post-second vaccination vs. Day 0 and Day 28 post-second vaccination vs. Day 0) ordered by treatment effect (absolute $\log_2$ fold change) are listed in Supplementary Tables 10 and 11. Response dynamics and functional context of DE genes over time was evaluated using heatmaps, unsupervised gene clustering, and supervised pathway enrichment analysis.

Pathways Enriched in DE Genes Pathway enrichment analysis was carried out by post-vaccination day using published gene sets outlined in Supplementary Table 27. Significantly enriched gene sets for each of the six pathway resources are given in Supplementary Tables 12 to 23. Enriched KEGG pathway maps color-coded by expression fold change and fold change significance are shown in Supplementary Figures 16 to 35. Venn diagrams that summarize the overlap in enriched pathways are provided in Supplementary Figure 36.

Gene Responses That Best Predict Protective Antibody Response Regularized linear regression analysis was carried out to identify combinations of gene log_2 fold changes that were correlated with maximum change in protective antibody responses at Day 14 or Day 28 post-second vaccination. The input set included 62 genes with a minimum average absolute fold change of 1.5 fold. For Day 14 post-second vaccination the best predictive model (α=0.19 and λ=0.2171) included 16 of these genes, of which 56% were DE expressed from pre-vaccination (Supplementary Table 31) and 56% are known to play a role in *REGULATION OF CELLULAR PROCESS* (Source: UniProt GO). Annotations and regression coefficients are listed in Supplementary Table 24. Scatterplots that summarize correlations between log_2 fold changes in gene expression and maximum protective antibody responses for each gene are provided in Supplementary Figure 37.

Gene Responses That Best Correlate With Changes in Proliferating and Cytokine Producing T Cells or Cytokine Responses Regularized canonical correlation analysis (RCCA) was performed to identify combinations of gene $\log_2$ fold changes that were best correlated with the respective Day 14 or Day 28 post-second vaccination T-cell or cytokine responses. The input set included genes with an average absolute fold change of $\geq$ 1.5 at either Day 14 or Day 28 post-second vaccination. Overall, 62 genes met

this cut off. Model performance was summarized in Supplementary Table 25. For T-cells, only the Day 14 post-second vaccination model met the cross validation score (CV) cut off of 0.5. For cytokines, both models (Day 14 and Day 28 post-second vaccination) met the cut off. Regularized canonical correlation analysis (RCCA) was performed to identify combinations of gene $\log_2$ fold changes that were best correlated with the respective Day 14 or Day 28 post-second vaccination T-cell or cytokine responses. The input set included genes with an average absolute fold change of ≥ 1.5 at either Day 14 or Day 28 post-second vaccination. Overall, 62 genes met this cut off. Model performance was summarized in Supplementary Table 25. For T-cells, only the Day 14 post-second vaccination model met the cross validation score (CV) cut off of 0.5. For cytokines, both models (Day 14 and Day 28 post-second vaccination) met the cut off. Correlation circle plots summarizing associations between variable sets based on the first two canonical variates are shown in Supplementary Figures 38 to 40.

Figures

Supplementary Figure 1: Analysis population consort flow diagram.

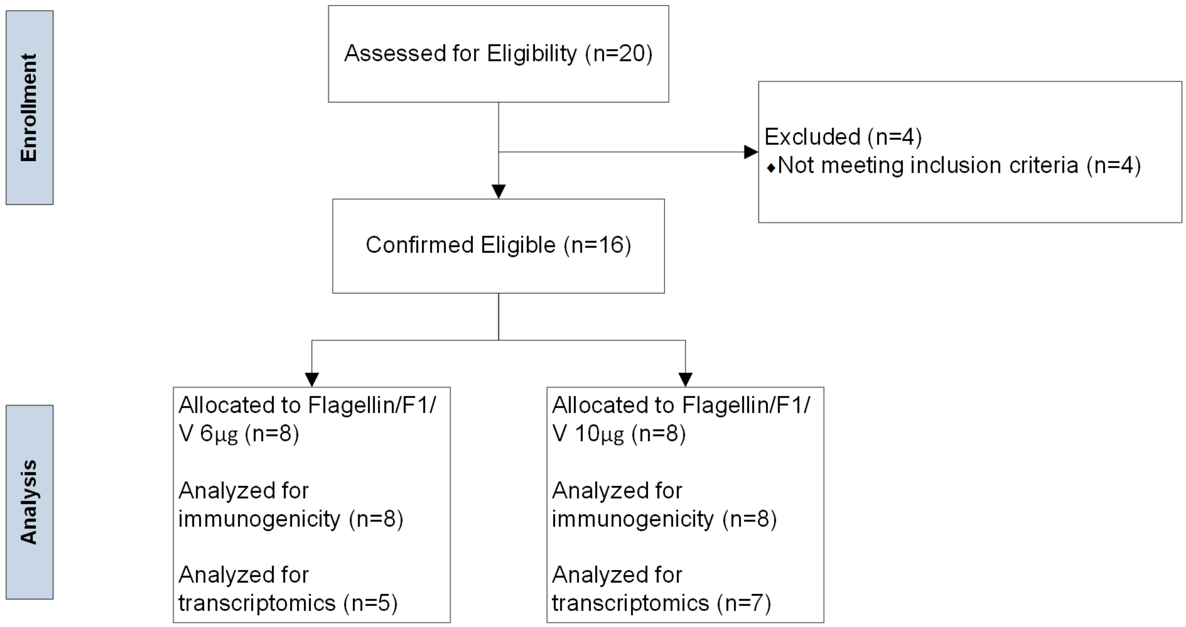

Supplementary Figure 2: Scatterplots that summarize the correlation between log_2 fold change in inverse Caspase-3 levels and log_2 fold change in ELISA IgG concentration and titer for F1 antigen. The green solid trend line represents a locally weighted regression fit.

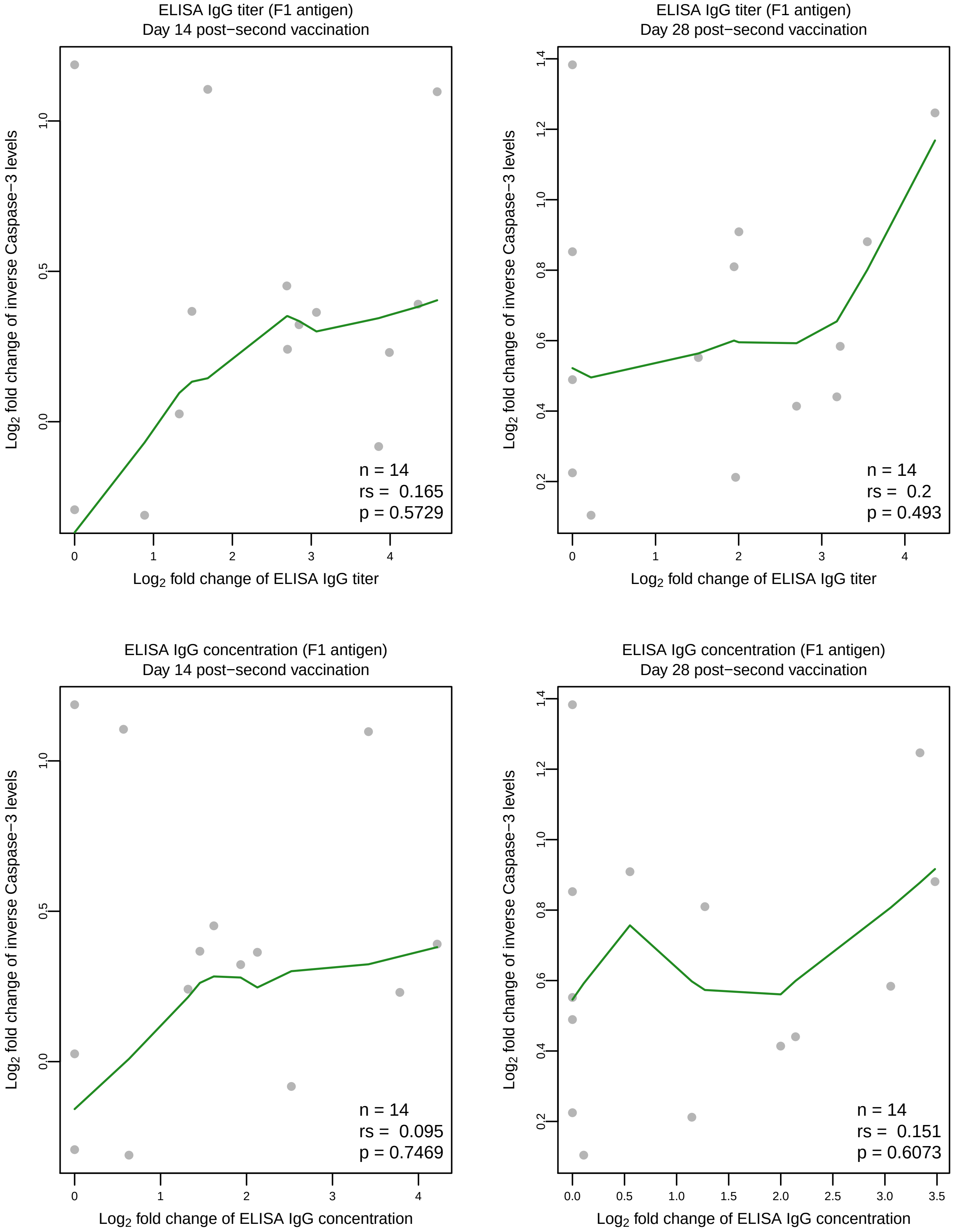

Supplementary Figure 3: Scatterplots that summarize the correlation between log_2 fold change in inverse Caspase-3 levels and log_2 fold change in ELISA IgG concentration and titer for V antigen. The green solid trend line represents a locally weighted regression fit.

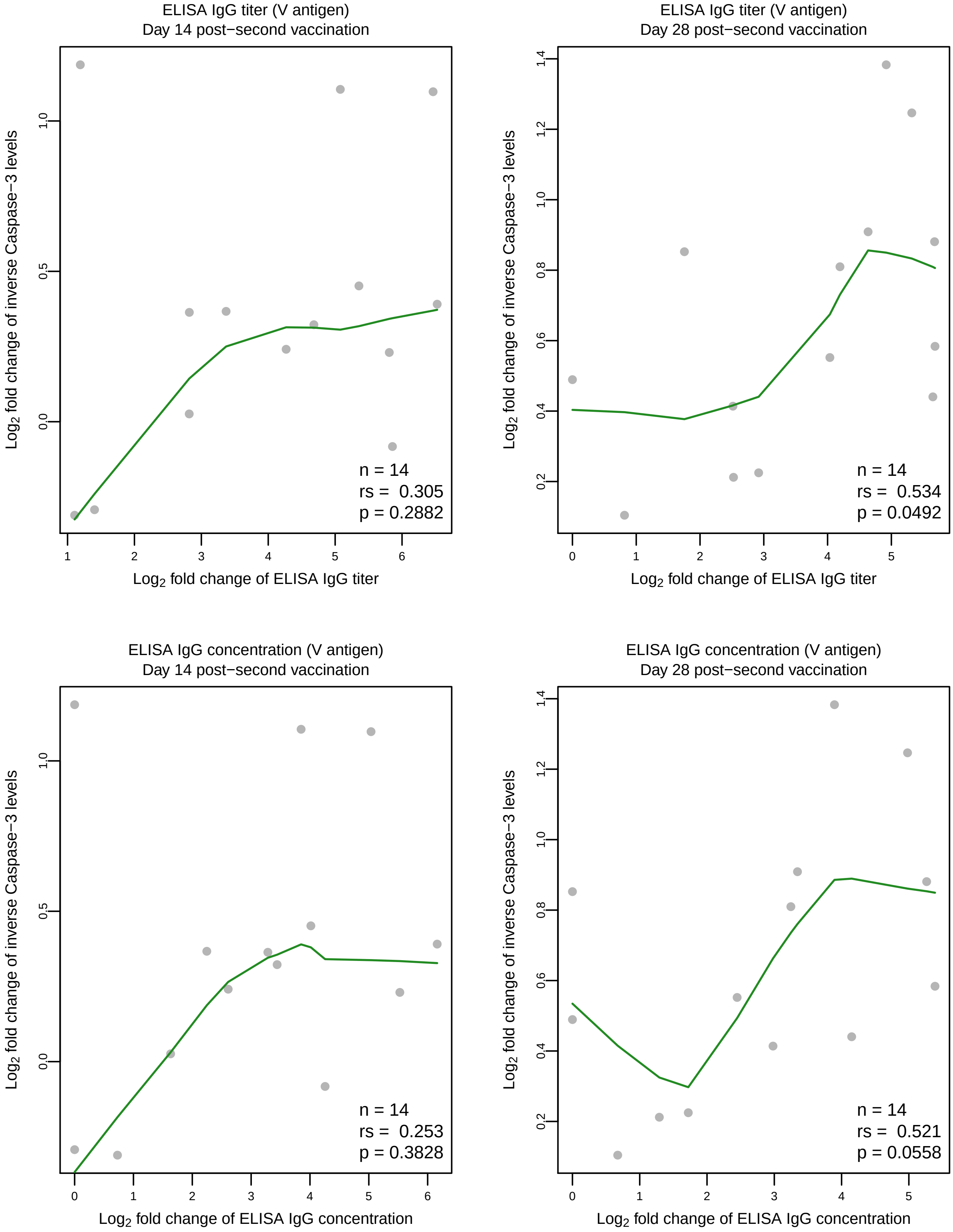

Supplementary Figure 4: Scatterplots that summarize the correlation between log_2 fold change in inverse Caspase-3 levels (y-axis) and log_2 fold change in the stimulation index of CD4+/CFSElo/IL-6+ T-cells (x-axis) by post-vaccination day. The green solid trend line represents a locally weighted regression fit.

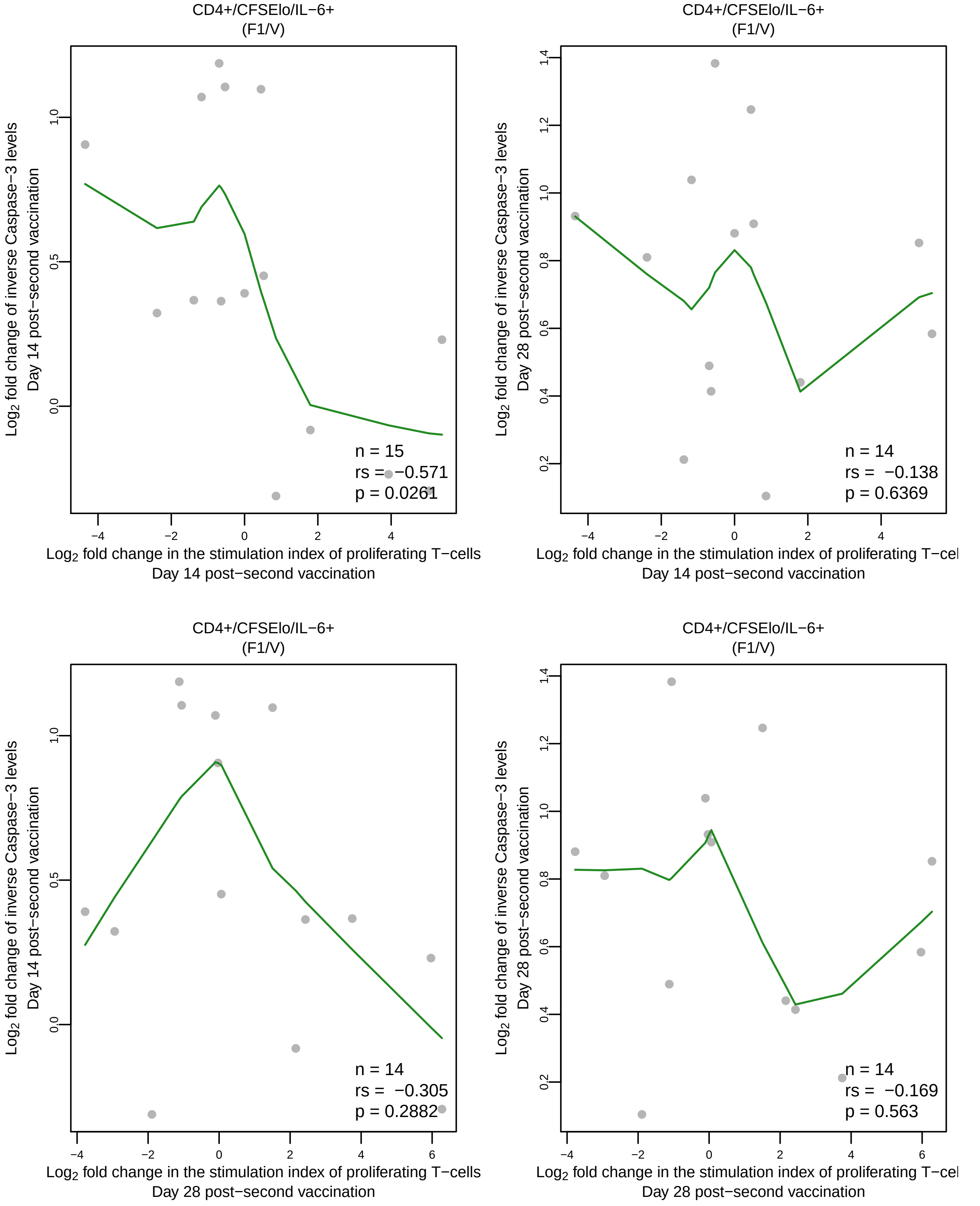

Supplementary Figure 5: Scatterplots that summarize the correlation between log_2 fold change in inverse Caspase-3 levels (y-axis) and log_2 fold change in the stimulation index of CD4+/CFSElo/IFNγ+ T-cells (x-axis) by post-vaccination day. The green solid trend line represents a locally weighted regression fit.

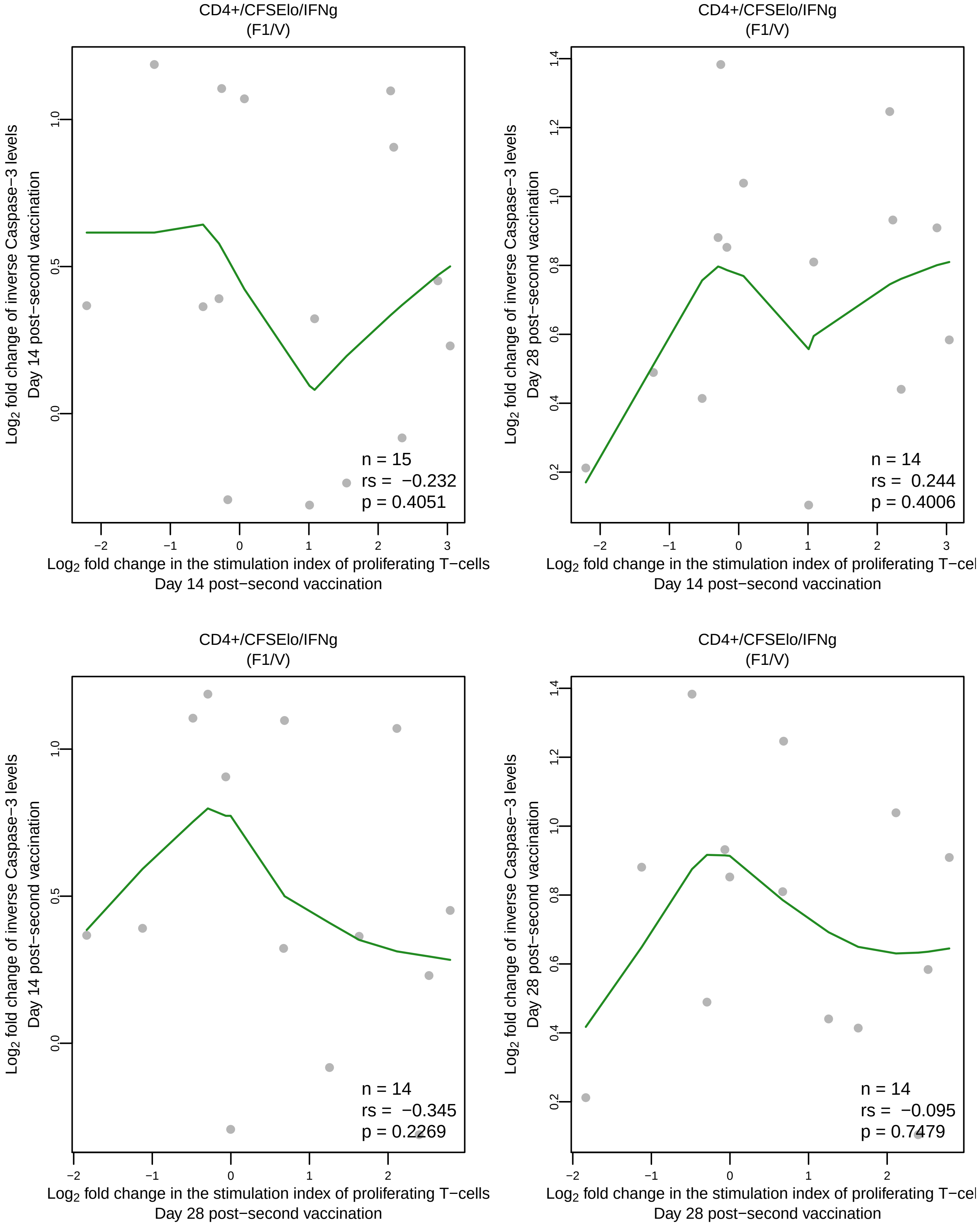

Supplementary Figure 6: Boxplots of log_2 counts per million before TMM normalization (RNA-Seq). Timepoints day 14 and day 28 refer to days 14 and 28 post-second vaccination.

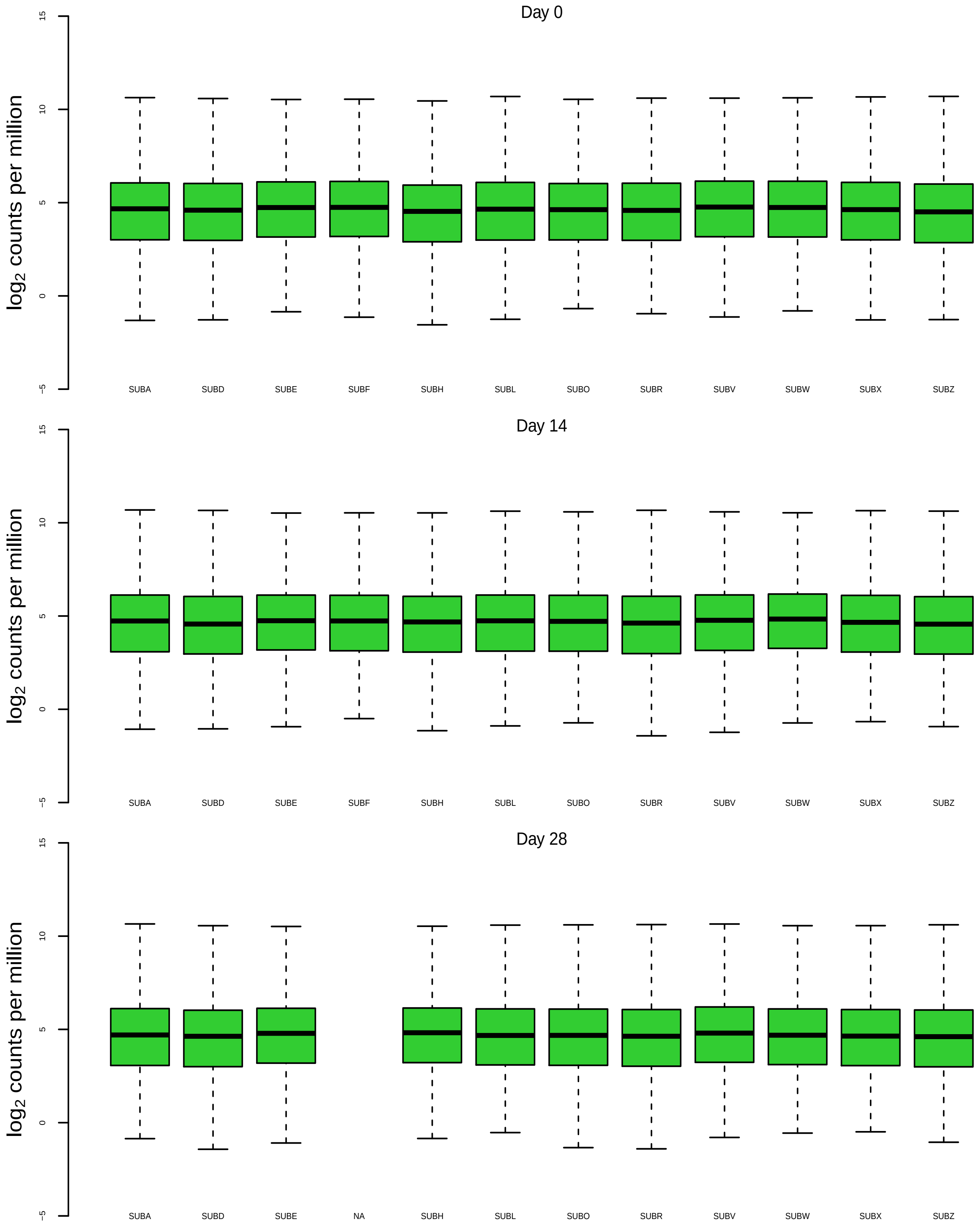

Supplementary Figure 7: Boxplots of log_2 counts per million after TMM normalization (RNA-Seq). Timepoints day 14 and day 28 refer to days 14 and 28 post-second vaccination.

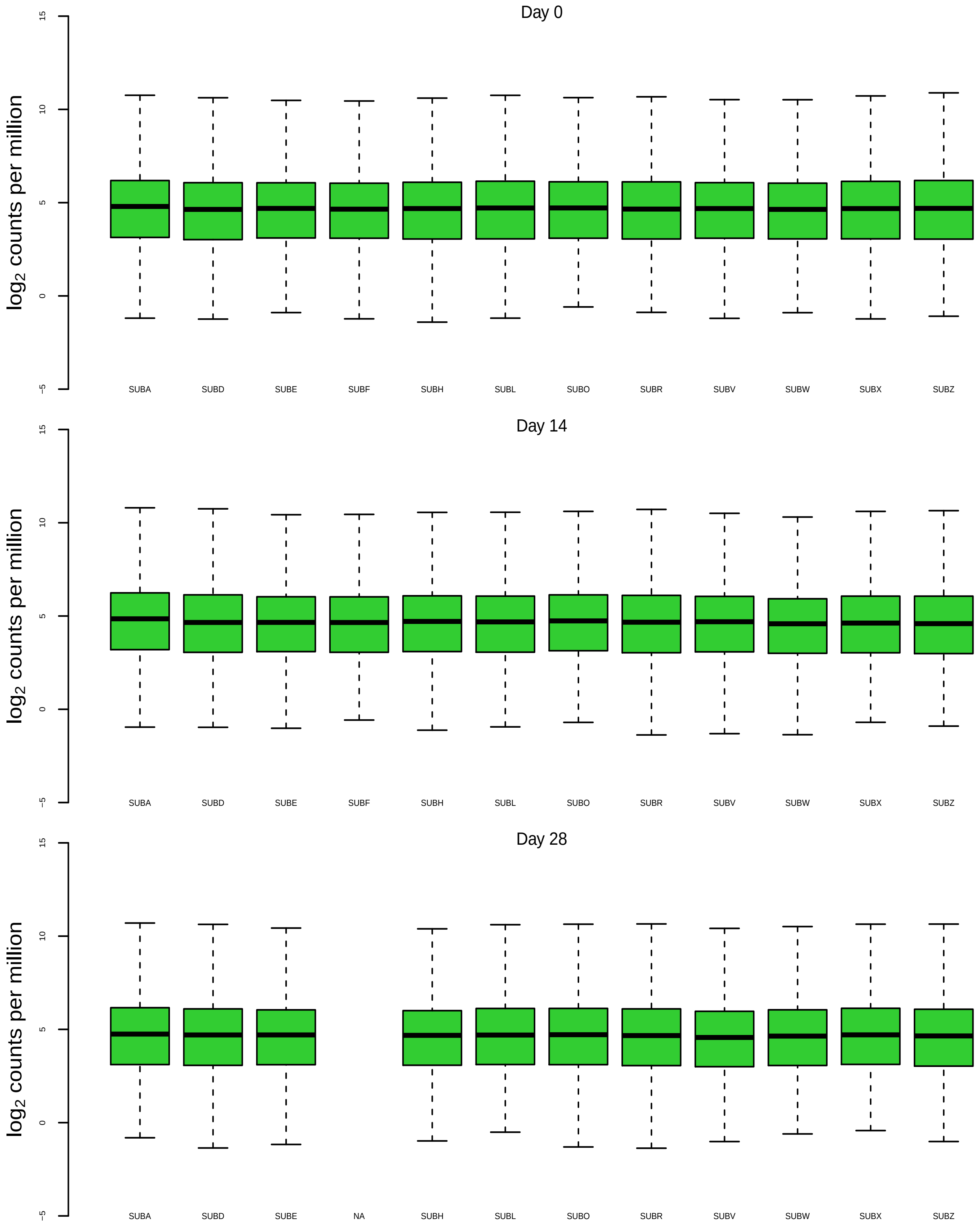

Supplementary Figure 8: Empirical cumulative distribution function plots of log_2 counts per million before and after TMM normalization (RNA-Seq). Timepoints day 14 and day 28 refer to days 14 and 28 post-second vaccination.

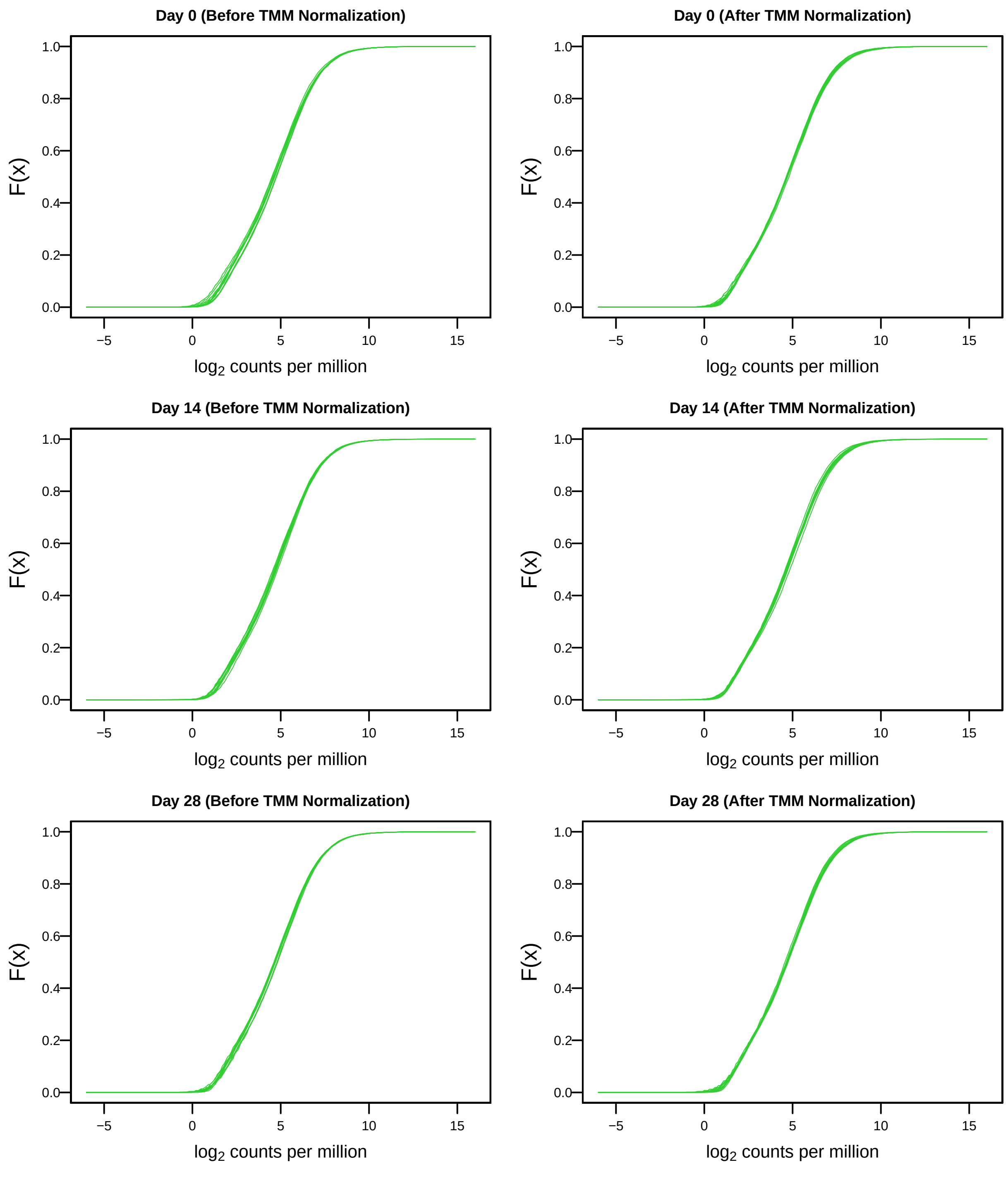

Supplementary Figure 9: Spearman correlation heatmap of TMM-normalized $\log_2$ counts per million values (RNA-Seq). Rows and columns both represent samples. Cells colored red have a higher Spearman correlation coefficient than those colored blue.

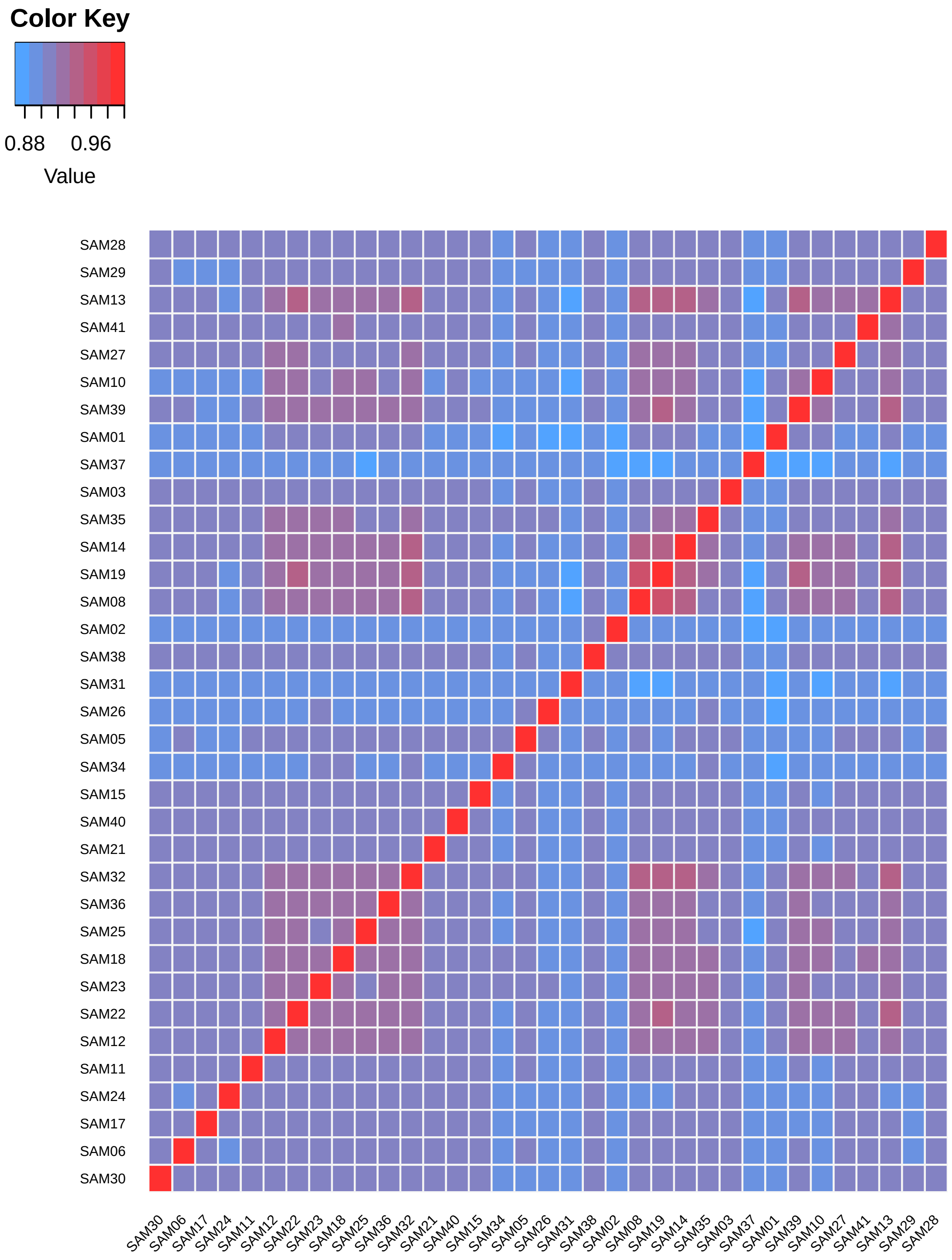

Supplementary Figure 10: PCA biplots (RNA-Seq). 95% confidence ellipses are drawn. The right panel is identical to the left panel except that it shows dataset labels for the four most outlying samples based on maximum Mahalanobis distance.

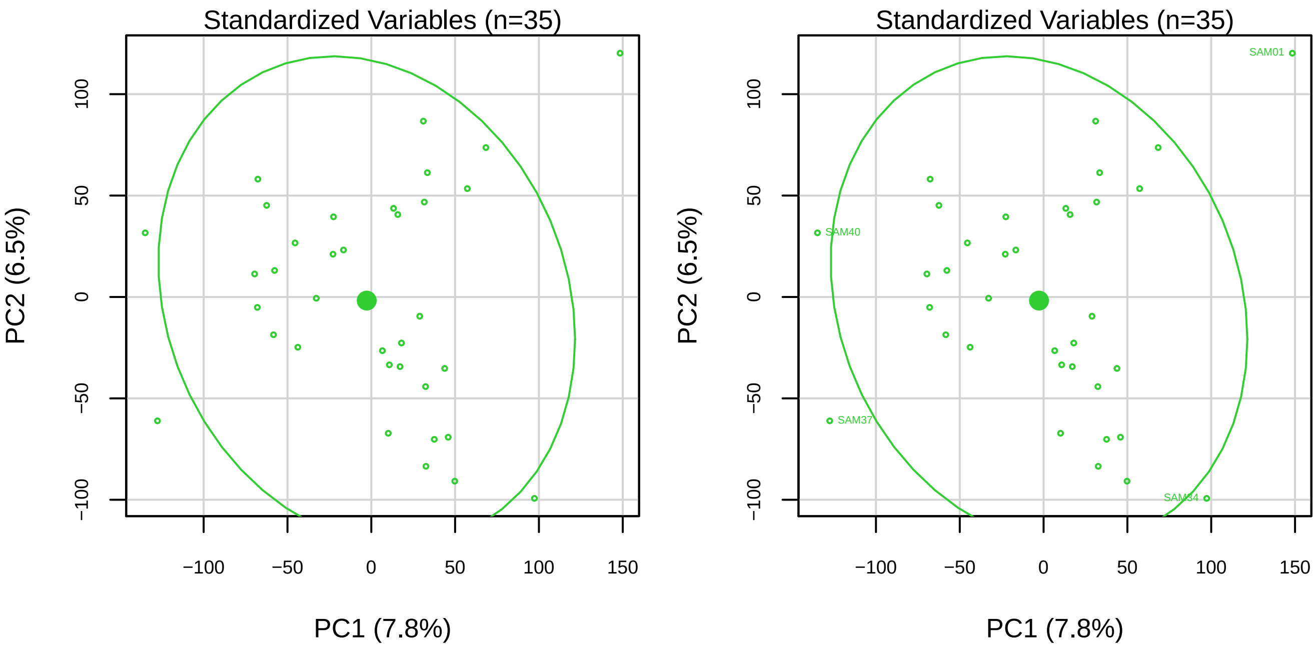

Supplementary Figure 11: Non-metric multidimensional scaling biplots (RNA-Seq). For the top panels, Euclidean distance was used to determine pairwise differences. For the bottom panels, 1-Spearman distance was used to determine pairwise differences. The right panels are identical to the left panels except that they show dataset labels for the four most outlying samples based on maximum Mahalanobis distance.

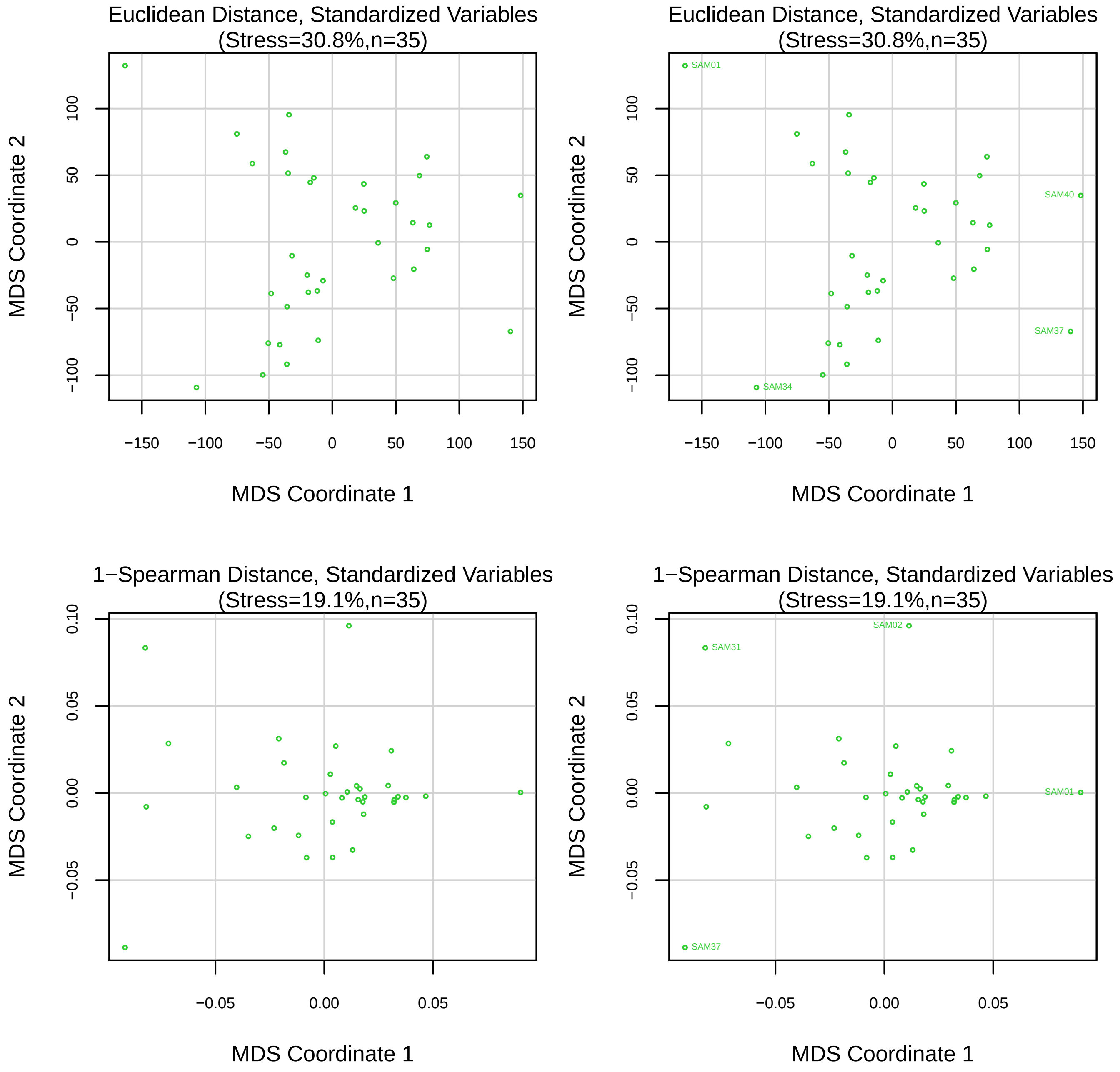

Supplementary Figure 12: Hierarchical Clustering Plots (RNA-Seq). Euclidean and 1-Spearman distances are hierarchically clustered using the complete linkage clustering algorithm.

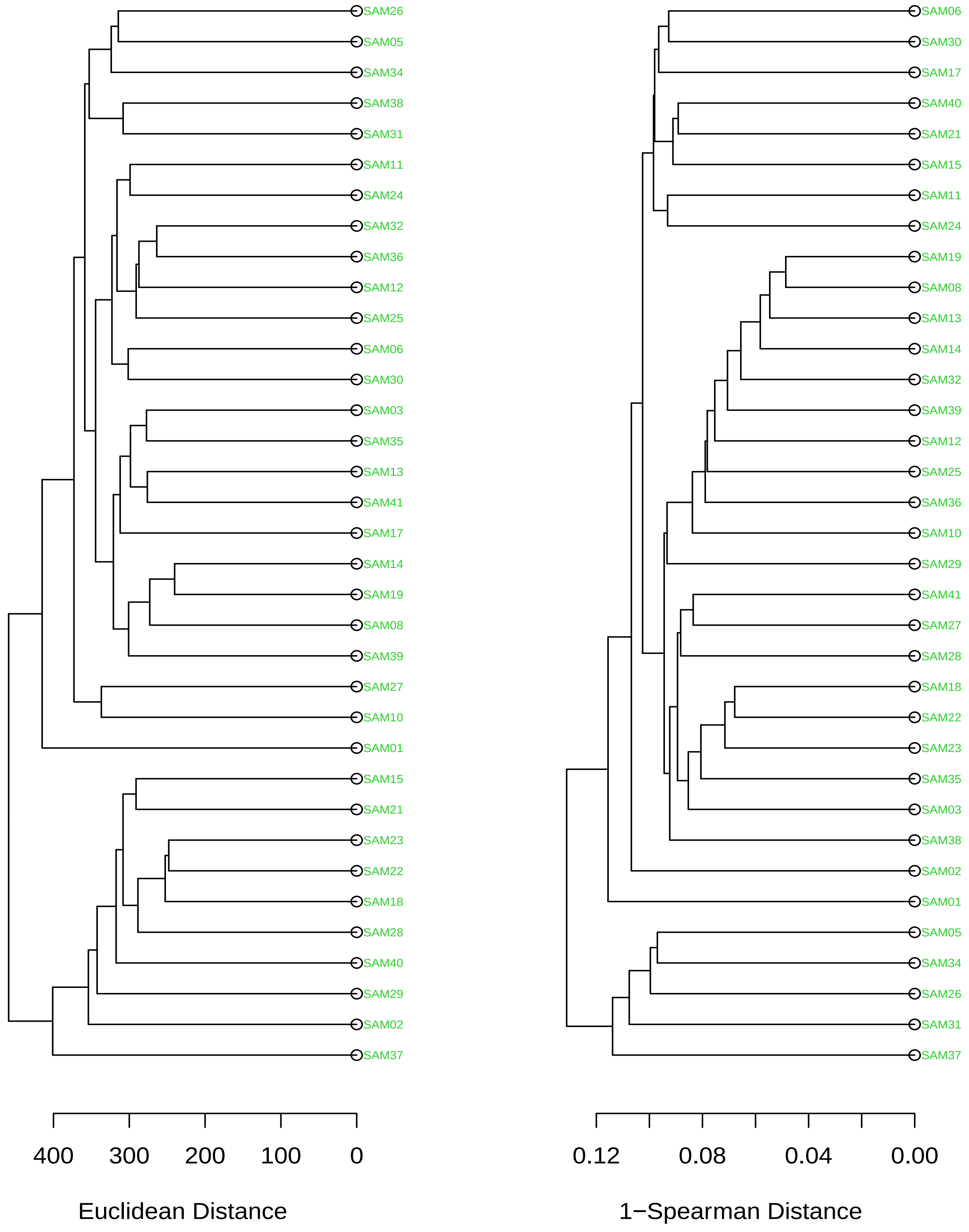

Supplementary Figure 13: Reverse empirical cumulative distribution function plots to assess lowly-expressed gene cut offs (RNA-Seq). The x-axis represents the log_2 count per million cut off for identifying lowly-expressed genes. The y-axis shows the percentage of all genes with an average gene expression level across all study samples that exceeds the respective cut off. The grey box indicates the target range of genes to be selected (between 10,000 and 14,000 genes). The black vertical line represents the low-expression cutoff.

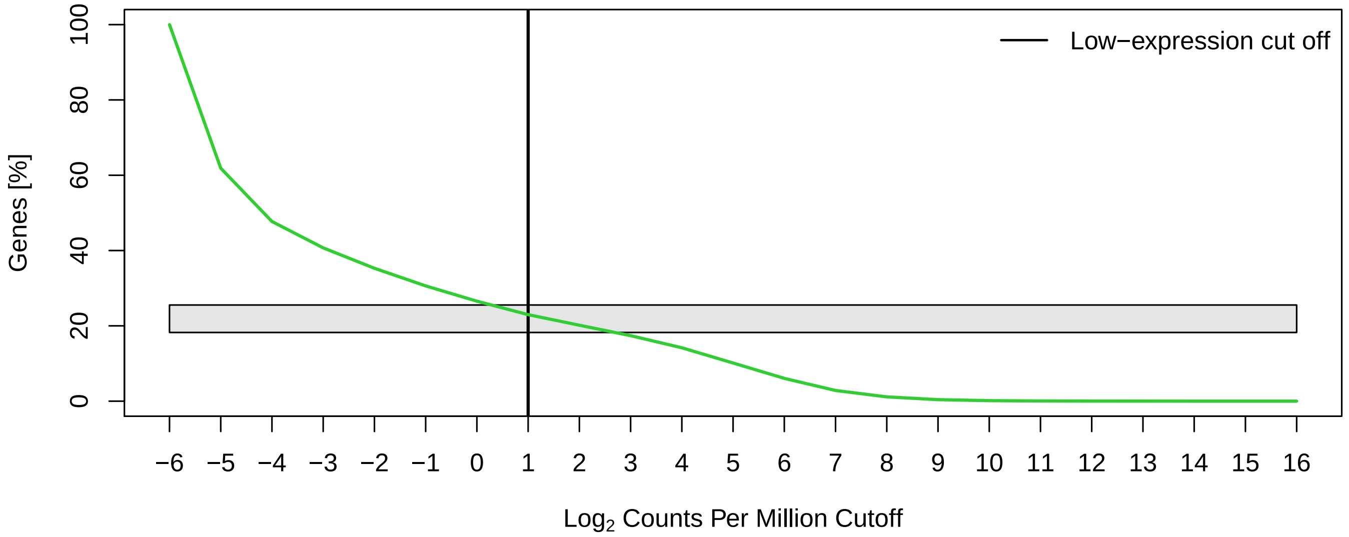

Supplementary Figure 14: Reverse empirical cumulative distribution function plots to assess fold change cut offs (RNA-Seq). The x-axis represents the log_2 fold change cut off for identifying DE genes. The y-axis shows the percentage of genes that meet the FDR-adjusted p-value cut off whose average fold change exceeds the respective cut off. The grey and black vertical lines represent the default (1.5 FC) and the actual (1.2 FC) cutoffs, respectively.

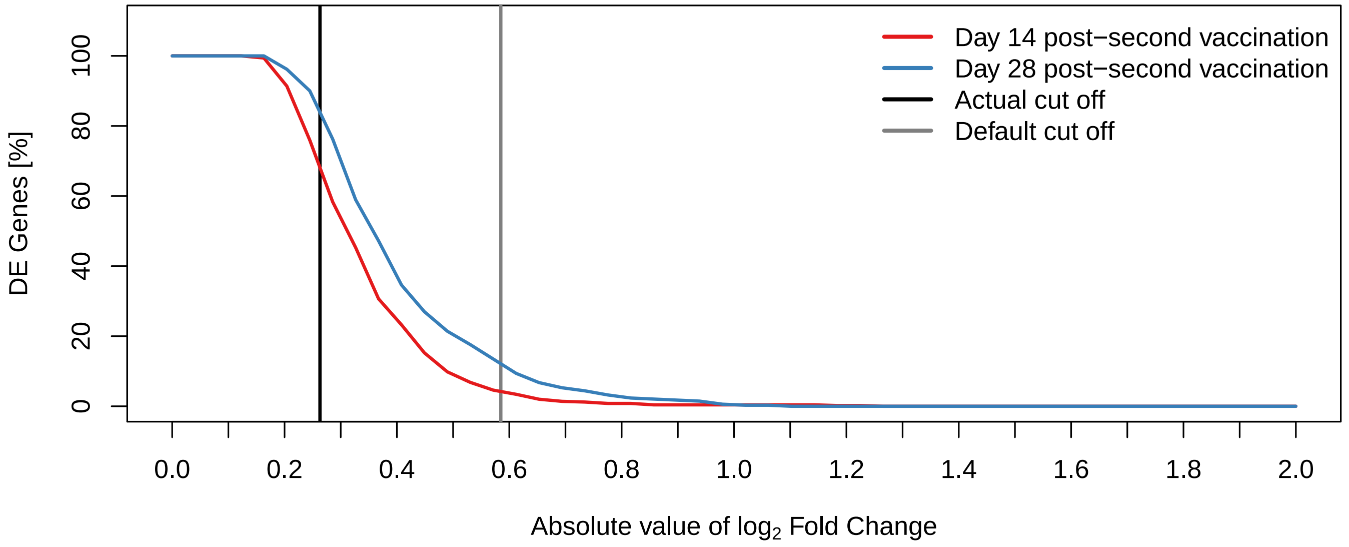

Supplementary Figure 15: Volcano and MA plots by timepoint (RNA-Seq). Blue lines indicate the pre-specified minimum fold change cut off. In red: signifcant genes; in grey: genes that did not pass the fold change cut offs; in black: genes that passed the fold change cut off but were not significant.

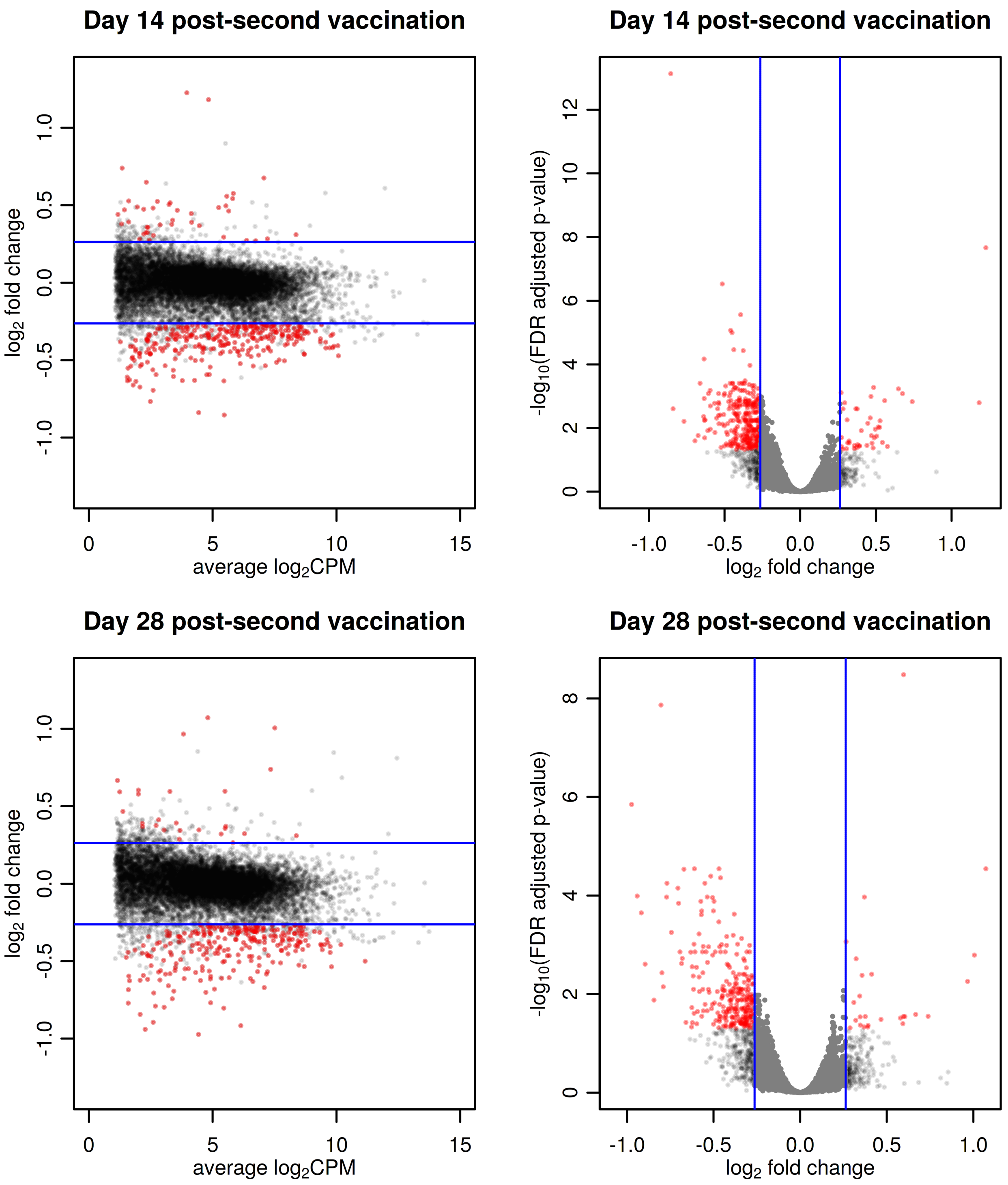

Supplementary Figure 16: KEGG Pathway Map - Amoebiasis (RNA-Seq, Day 28 post-second vaccination). Node color gradient encodes fold change from pre-vaccination (for multi-gene pathway nodes the median fold change is used). In red: up-regulated compared to pre-vaccination, in blue: down-regulated compared to pre-vaccination. In black: fold change close to 1, in dark grey: genes filtered out due to low overall expression, light grey: gene missing database mapping, white: non-human gene. DE genes are highlighted using red (significantly up-regulated) and blue (significantly down-regulated) node label and border colors.

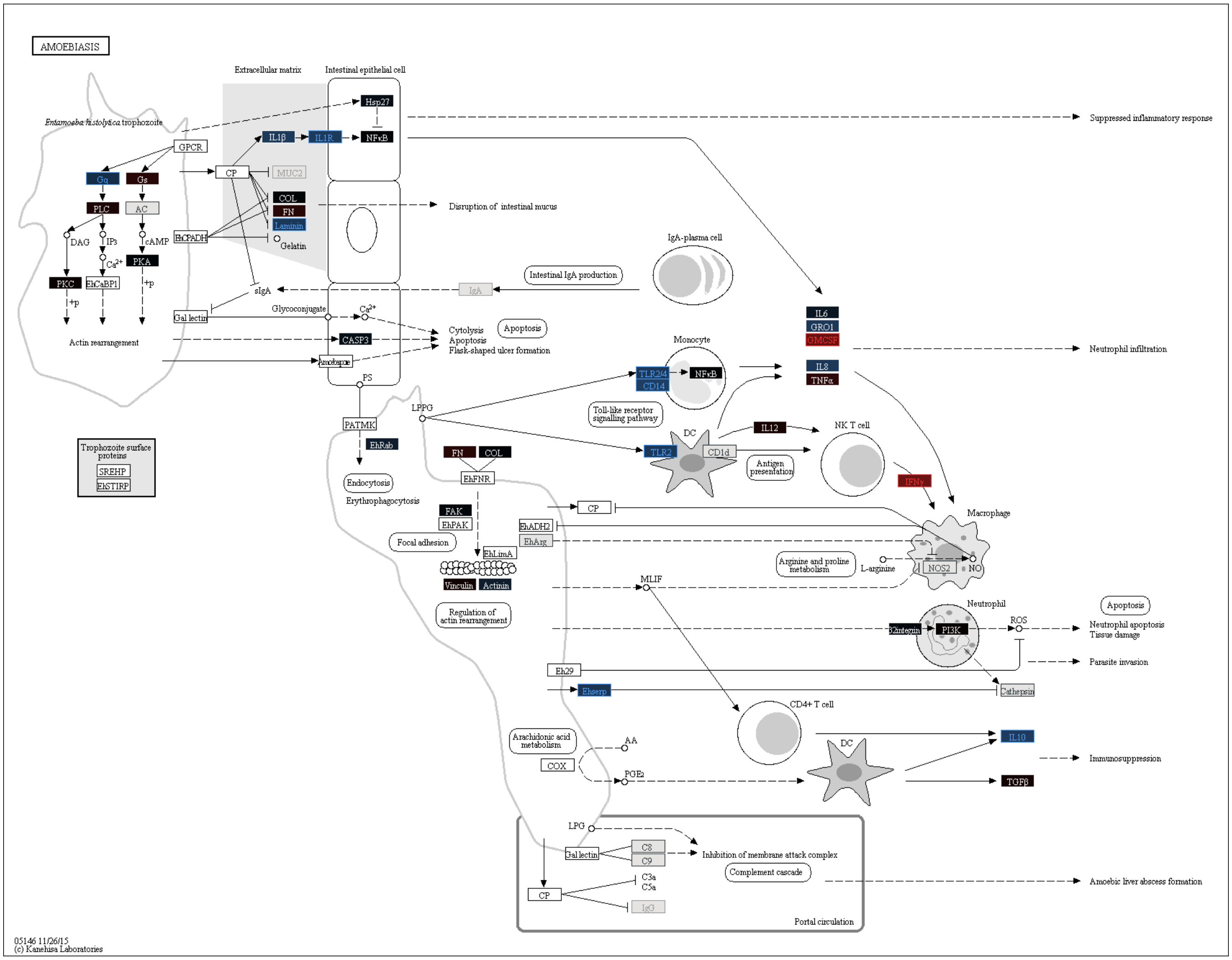

Supplementary Figure 17: KEGG Pathway Map - Complement and coagulation cascades (RNA-Seq, Day 14 post-second vaccination). Node color gradient encodes fold change from pre-vaccination (for multi-gene pathway nodes the median fold change is used). In red: up-regulated compared to pre-vaccination, in blue: down-regulated compared to pre-vaccination. In black: fold change close to 1, in dark grey: genes filtered out due to low overall expression, light grey: gene missing database mapping, white: non-human gene. DE genes are highlighted using red (significantly up-regulated) and blue (significantly down-regulated) node label and border colors.

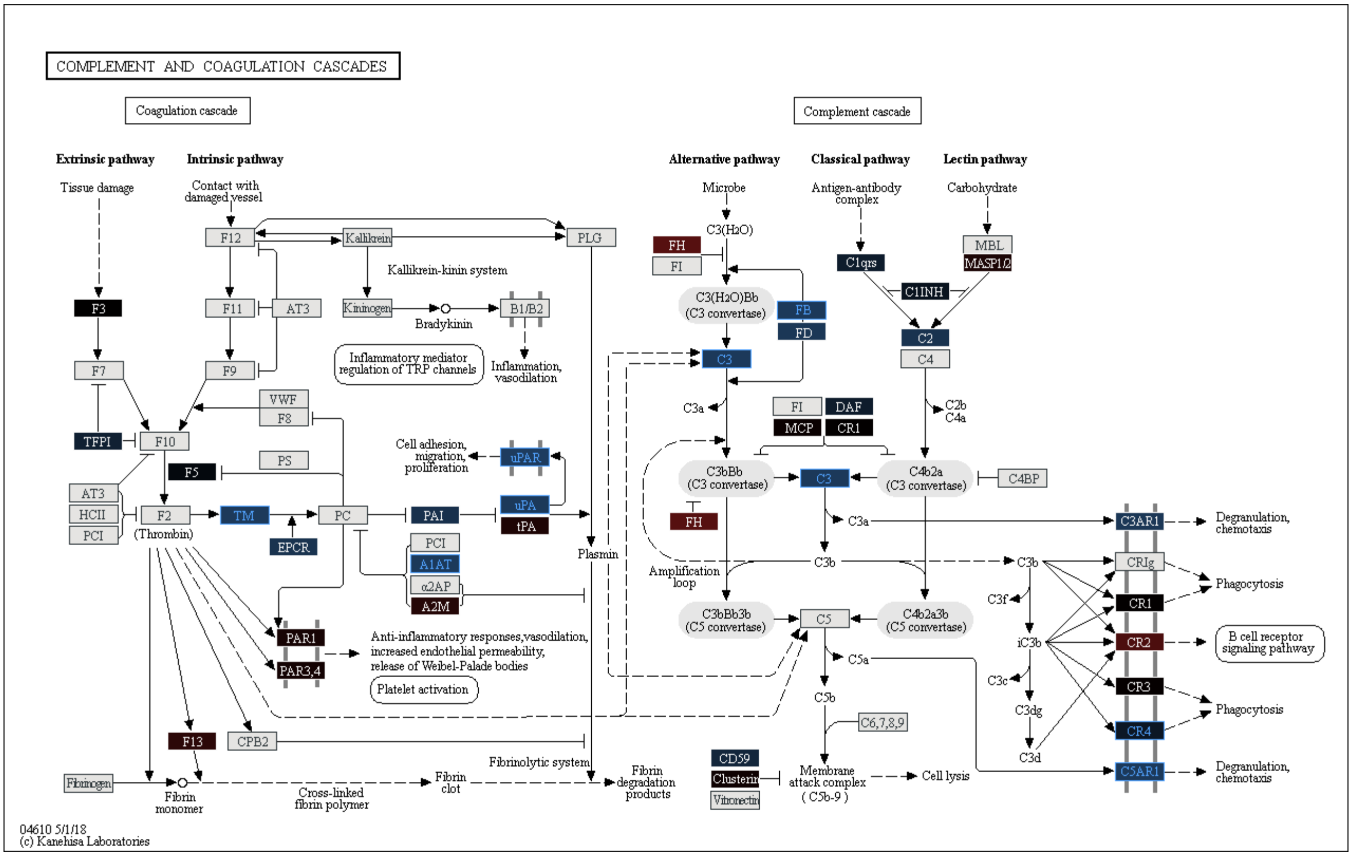

Supplementary Figure 18: KEGG Pathway Map - Complement and coagulation cascades (RNA-Seq, Day 28 post-second vaccination). Node color gradient encodes fold change from pre-vaccination (for multi-gene pathway nodes the median fold change is used). In red: up-regulated compared to pre-vaccination, in blue: down-regulated compared to pre-vaccination. In black: fold change close to 1, in dark grey: genes filtered out due to low overall expression, light grey: gene missing database mapping, white: non-human gene. DE genes are highlighted using red (significantly up-regulated) and blue (significantly down-regulated) node label and border colors.

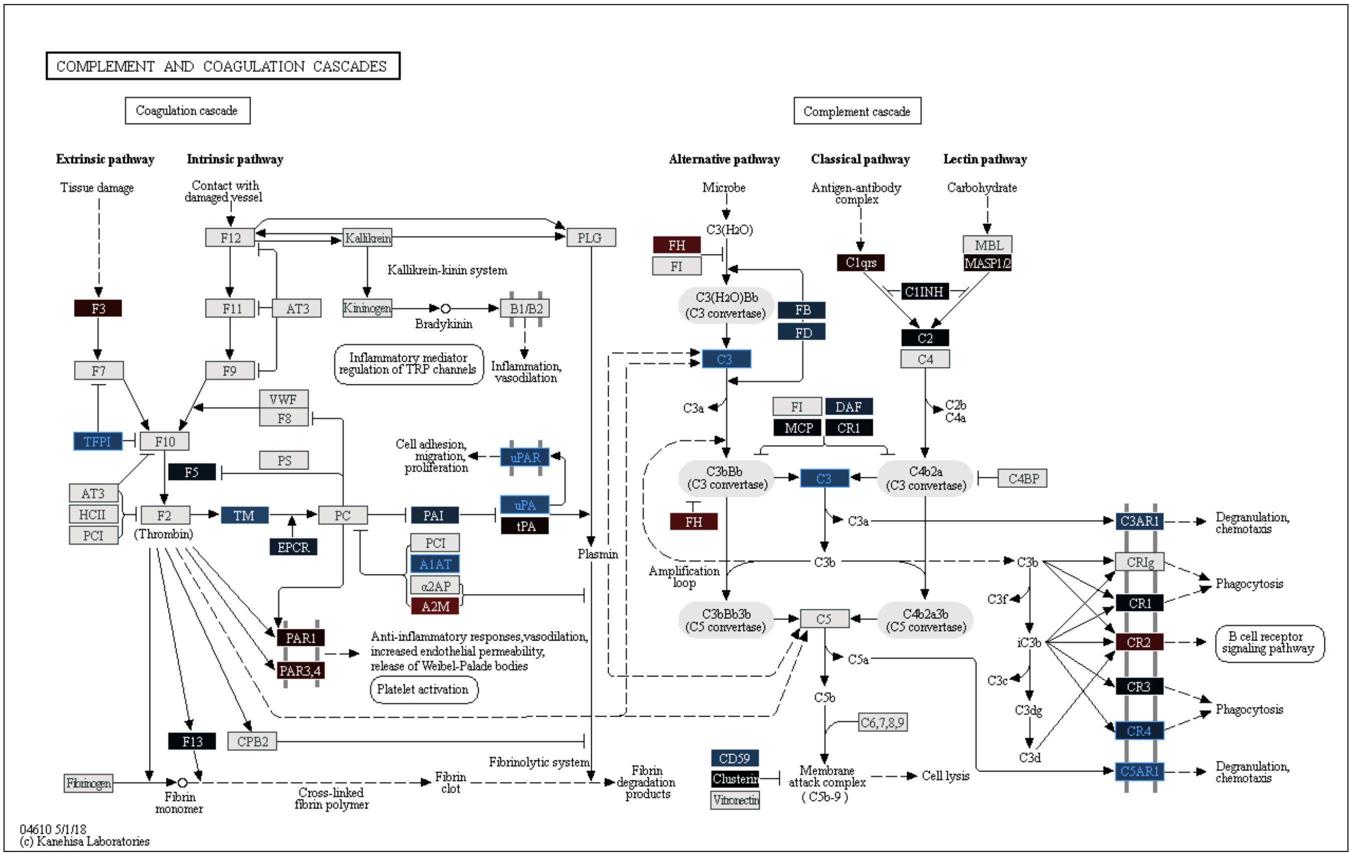

Supplementary Figure 19: KEGG Pathway Map - Cytokine-cytokine receptor interaction (RNA-Seq, Day 14 post-second vaccination). Node color gradient encodes fold change from pre-vaccination (for multi-gene pathway nodes the median fold change is used). In red: up-regulated compared to pre-vaccination, in blue: down-regulated compared to pre-vaccination. In black: fold change close to 1, in dark grey: genes filtered out due to low overall expression, light grey: gene missing database mapping, white: non-human gene. DE genes are highlighted using red (significantly up-regulated) and blue (significantly down-regulated) node label and border colors.

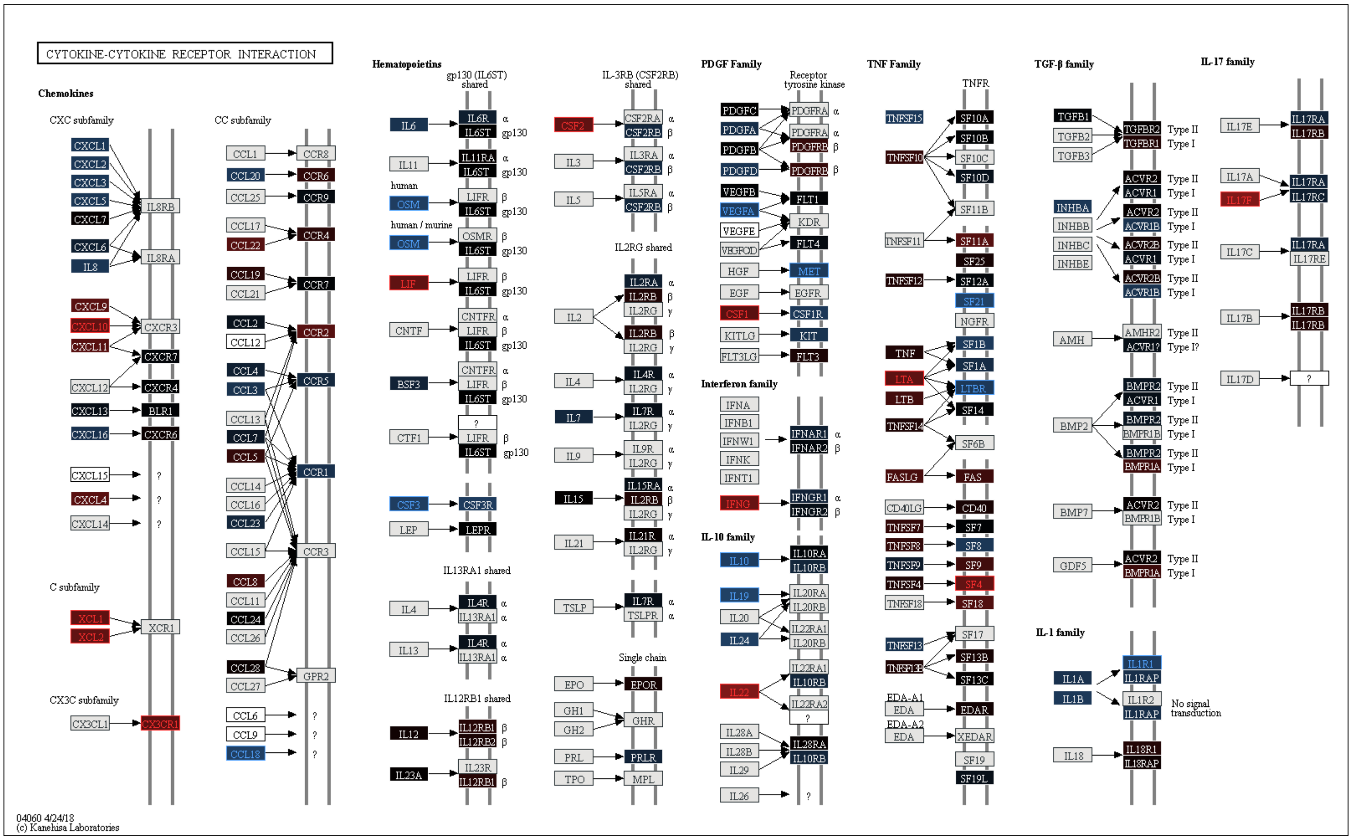

Supplementary Figure 20: KEGG Pathway Map - Cytokine-cytokine receptor interaction (RNA-Seq, Day 28 post-second vaccination). Node color gradient encodes fold change from pre-vaccination (for multi-gene pathway nodes the median fold change is used). In red: up-regulated compared to pre-vaccination, in blue: down-regulated compared to pre-vaccination. In black: fold change close to 1, in dark grey: genes filtered out due to low overall expression, light grey: gene missing database mapping, white: non-human gene. DE genes are highlighted using red (significantly up-regulated) and blue (significantly down-regulated) node label and border colors.

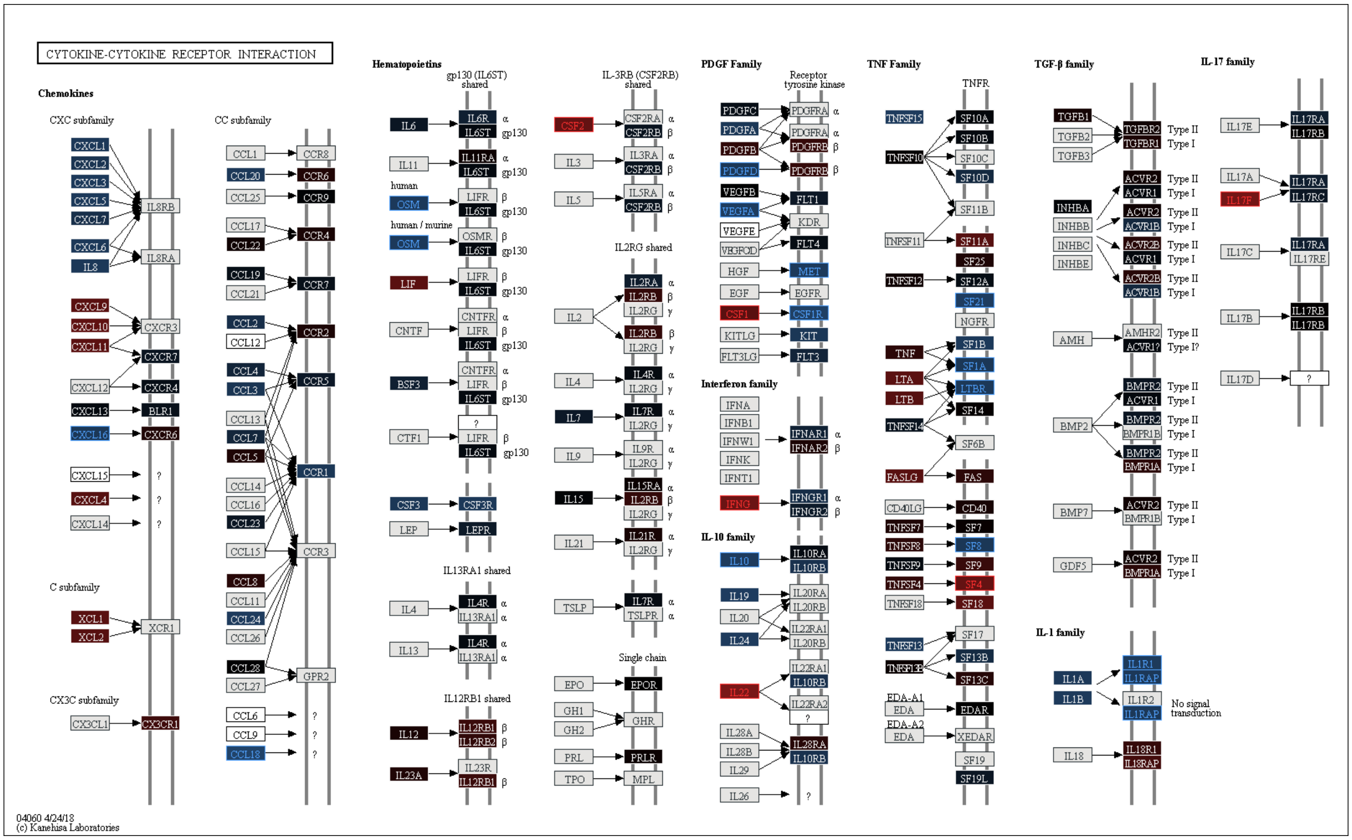

Supplementary Figure 21: KEGG Pathway Map - Fluid shear stress and atherosclerosis (RNA-Seq, Day 14 post-second vaccination). Node color gradient encodes fold change from pre-vaccination (for multi-gene pathway nodes the median fold change is used). In red: up-regulated compared to pre-vaccination, in blue: down-regulated compared to pre-vaccination. In black: fold change close to 1, in dark grey: genes filtered out due to low overall expression, light grey: gene missing database mapping, white: non-human gene. DE genes are highlighted using red (significantly up-regulated) and blue (significantly down-regulated) node label and border colors.

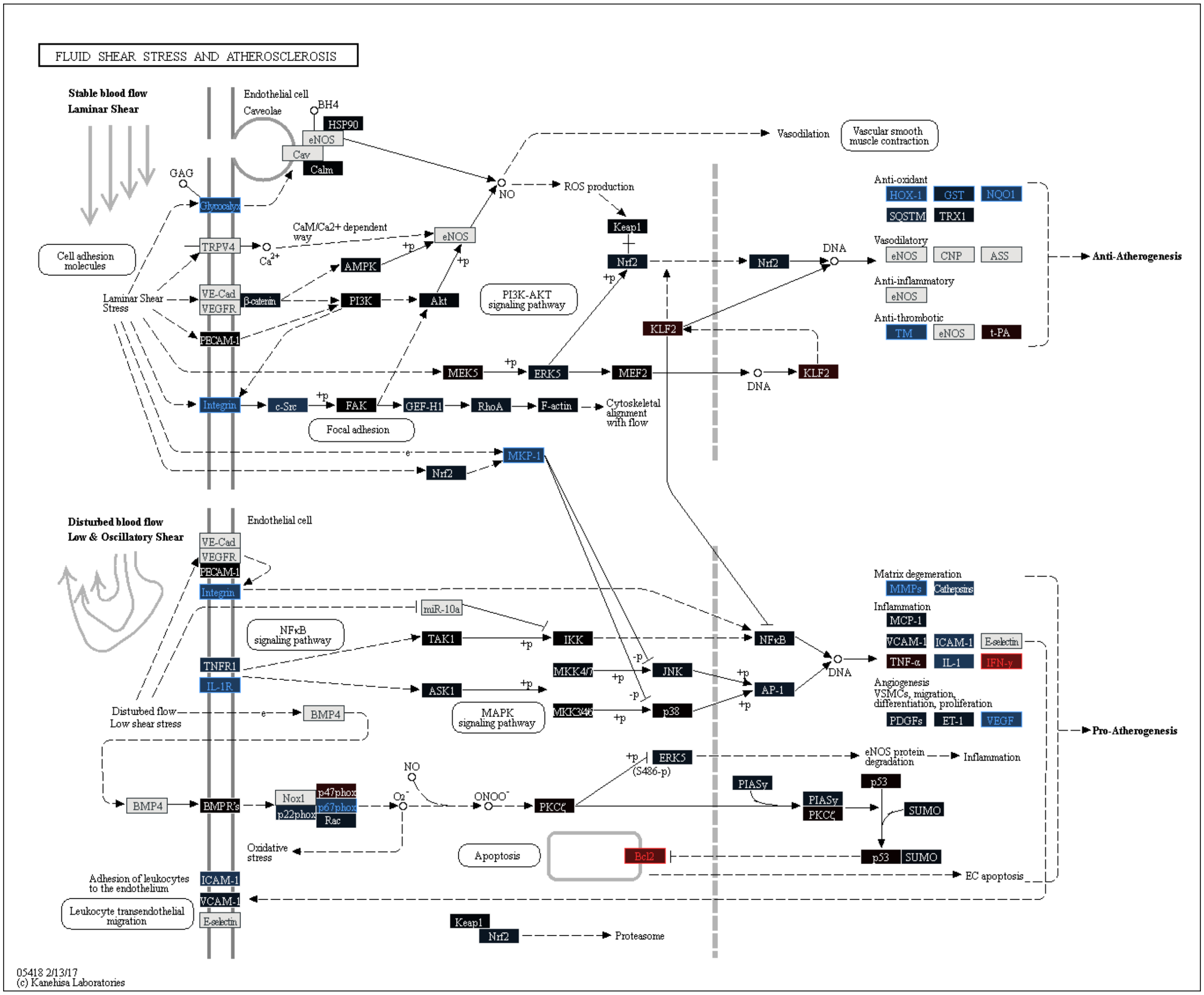

Supplementary Figure 22: KEGG Pathway Map - Fluid shear stress and atherosclerosis (RNA-Seq, Day 28 post-second vaccination). Node color gradient encodes fold change from pre-vaccination (for multi-gene pathway nodes the median fold change is used). In red: up-regulated compared to pre-vaccination, in blue: down-regulated compared to pre-vaccination. In black: fold change close to 1, in dark grey: genes filtered out due to low overall expression, light grey: gene missing database mapping, white: non-human gene. DE genes are highlighted using red (significantly up-regulated) and blue (significantly down-regulated) node label and border colors.

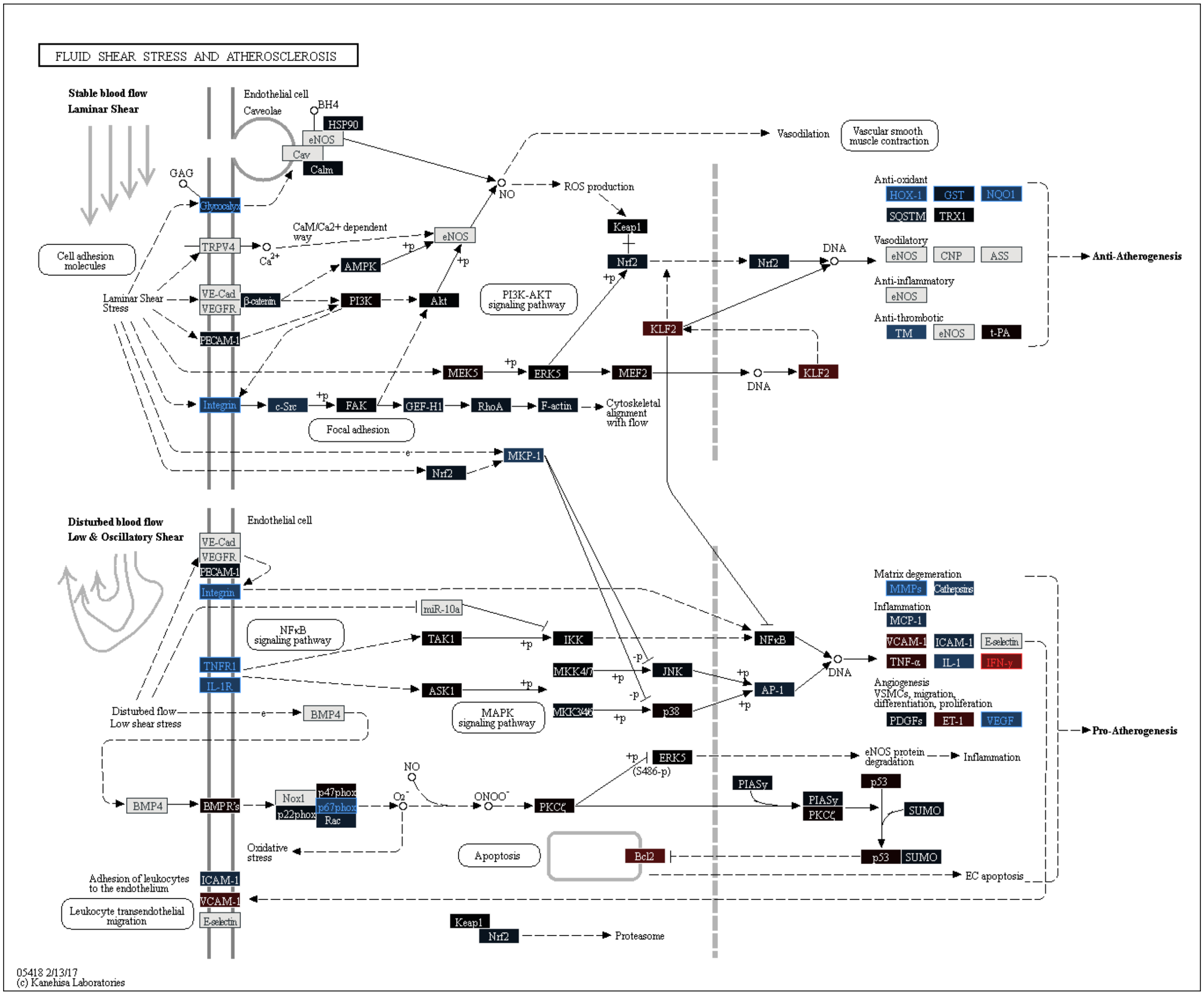

Supplementary Figure 23: KEGG Pathway Map - Hematopoietic cell lineage (RNA-Seq, Day 28 post-second vaccination). Node color gradient encodes fold change from pre-vaccination (for multi-gene pathway nodes the median fold change is used). In red: up-regulated compared to pre-vaccination, in blue: down-regulated compared to pre-vaccination. In black: fold change close to 1, in dark grey: genes filtered out due to low overall expression, light grey: gene missing database mapping, white: non-human gene. DE genes are highlighted using red (significantly up-regulated) and blue (significantly down-regulated) node label and border colors.

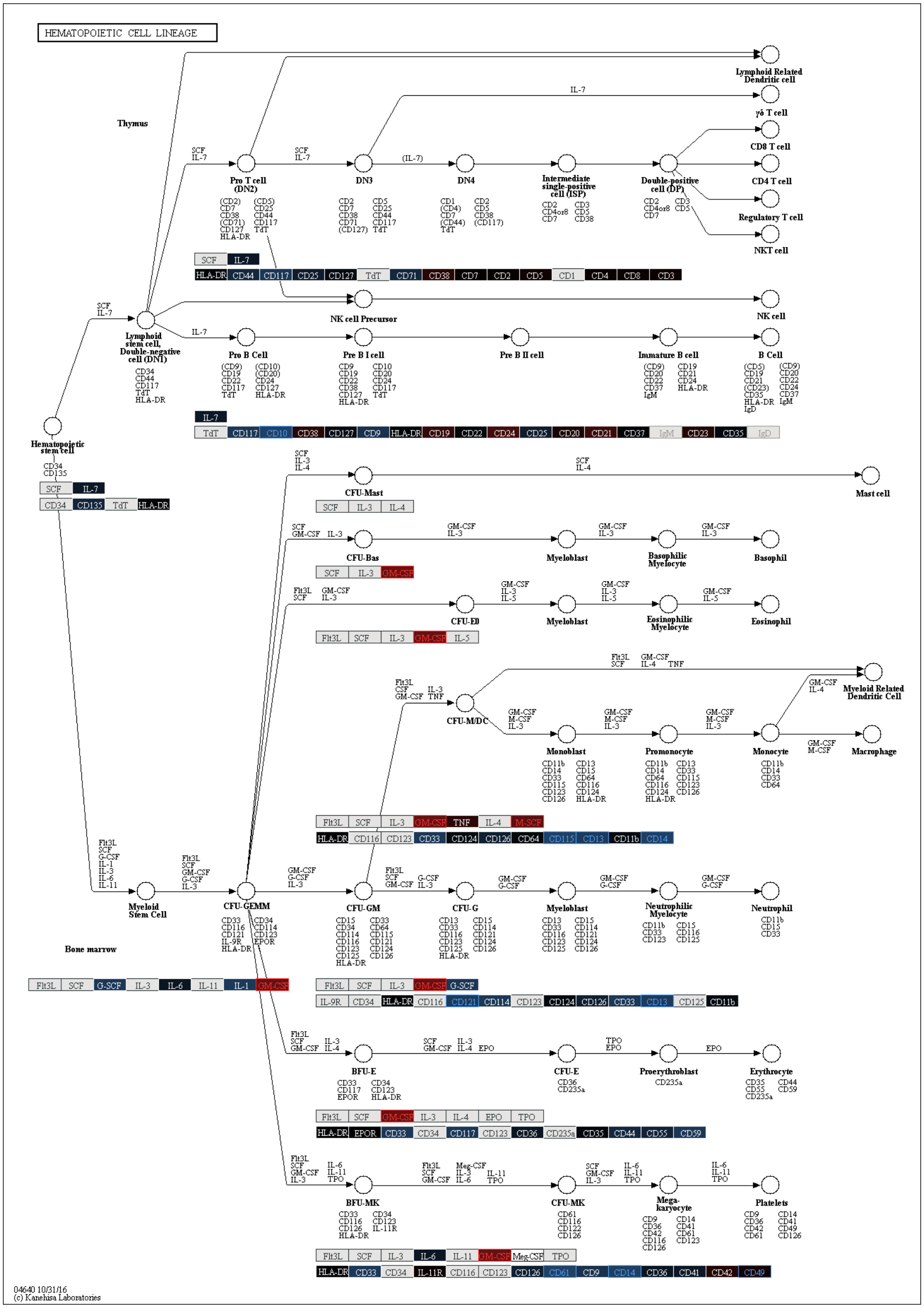

Supplementary Figure 24: KEGG Pathway Map - IL-17 signaling pathway (RNA-Seq, Day 28 post-second vaccination). Node color gradient encodes fold change from pre-vaccination (for multi-gene pathway nodes the median fold change is used). In red: up-regulated compared to pre-vaccination, in blue: down-regulated compared to pre-vaccination. In black: fold change close to 1, in dark grey: genes filtered out due to low overall expression, light grey: gene missing database mapping, white: non-human gene. DE genes are highlighted using red (significantly up-regulated) and blue (significantly down-regulated) node label and border colors.

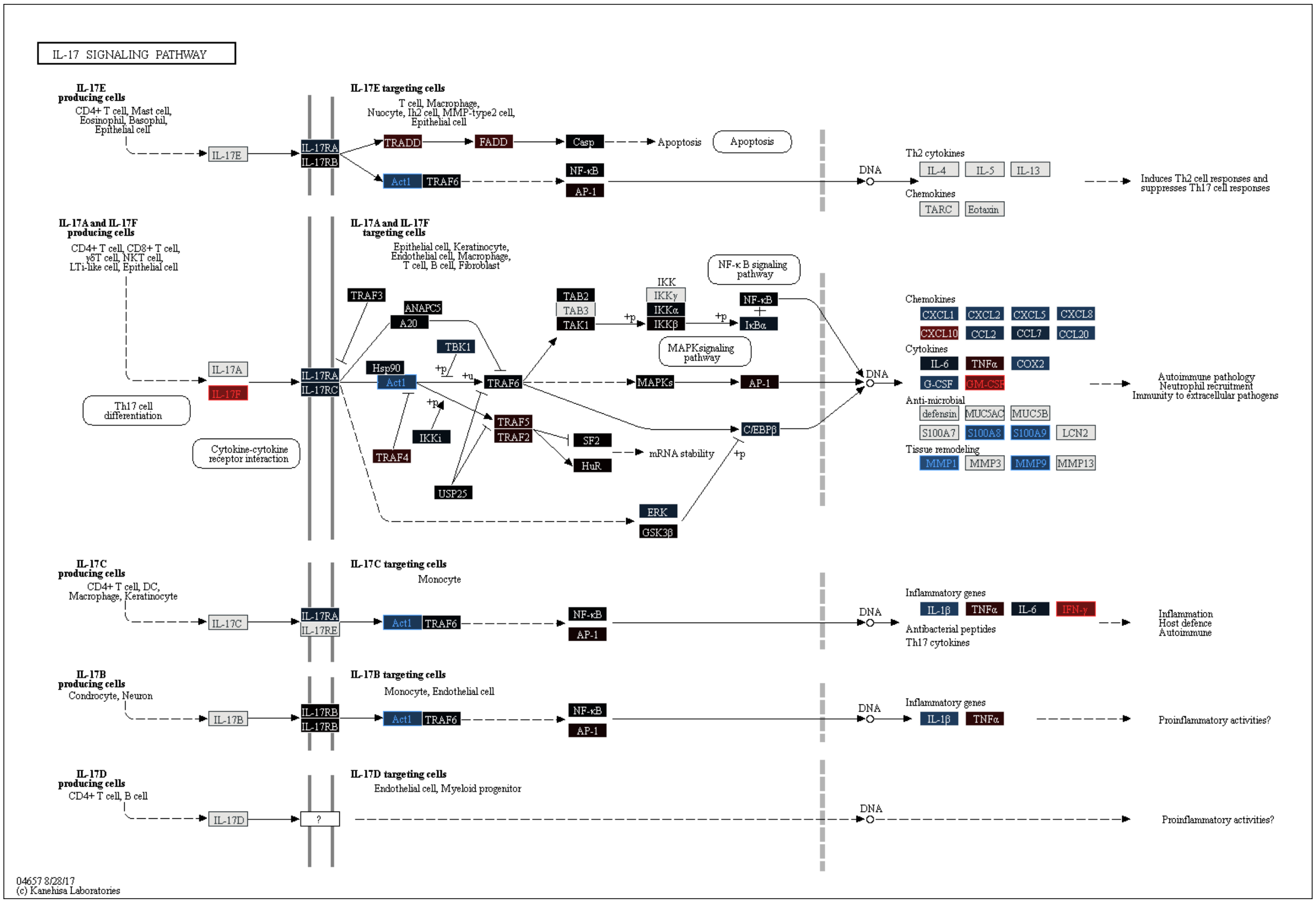

Supplementary Figure 25: KEGG Pathway Map - Jak-STAT signaling pathway (RNA-Seq, Day 14 post-second vaccination). Node color gradient encodes fold change from pre-vaccination (for multi-gene pathway nodes the median fold change is used). In red: up-regulated compared to pre-vaccination, in blue: down-regulated compared to pre-vaccination. In black: fold change close to 1, in dark grey: genes filtered out due to low overall expression, light grey: gene missing database mapping, white: non-human gene. DE genes are highlighted using red (significantly up-regulated) and blue (significantly down-regulated) node label and border colors.

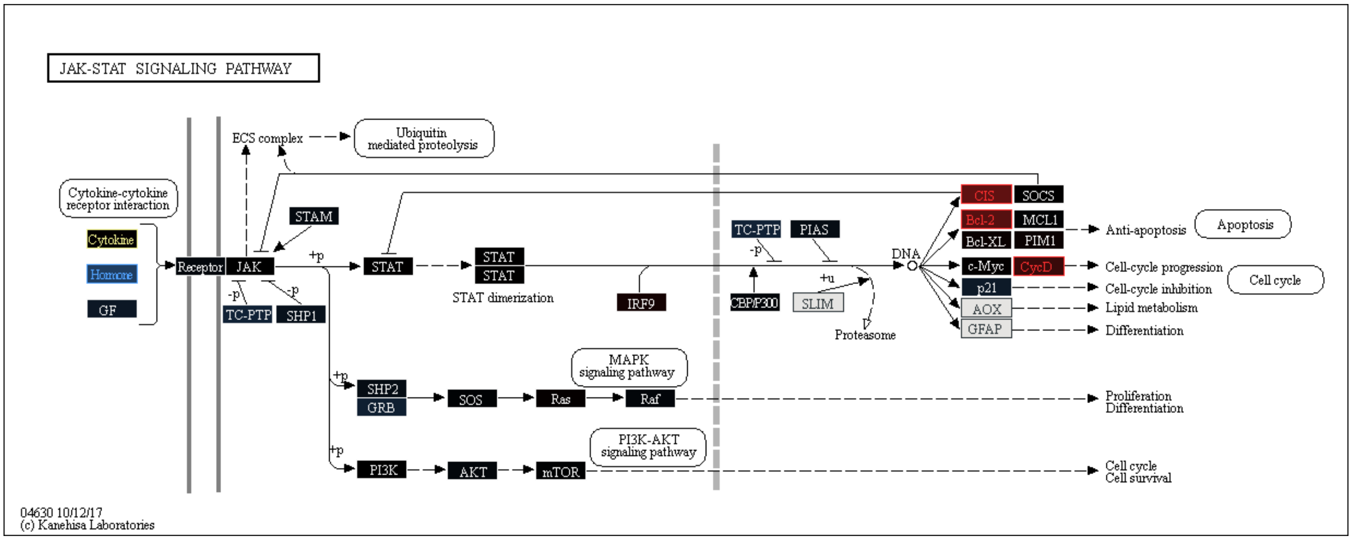

Supplementary Figure 26: KEGG Pathway Map - Osteoclast differentiation (RNA-Seq, Day 14 post-second vaccination). Node color gradient encodes fold change from pre-vaccination (for multi-gene pathway nodes the median fold change is used). In red: up-regulated compared to pre-vaccination, in blue: down-regulated compared to pre-vaccination. In black: fold change close to 1, in dark grey: genes filtered out due to low overall expression, light grey: gene missing database mapping, white: non-human gene. DE genes are highlighted using red (significantly up-regulated) and blue (significantly down-regulated) node label and border colors.

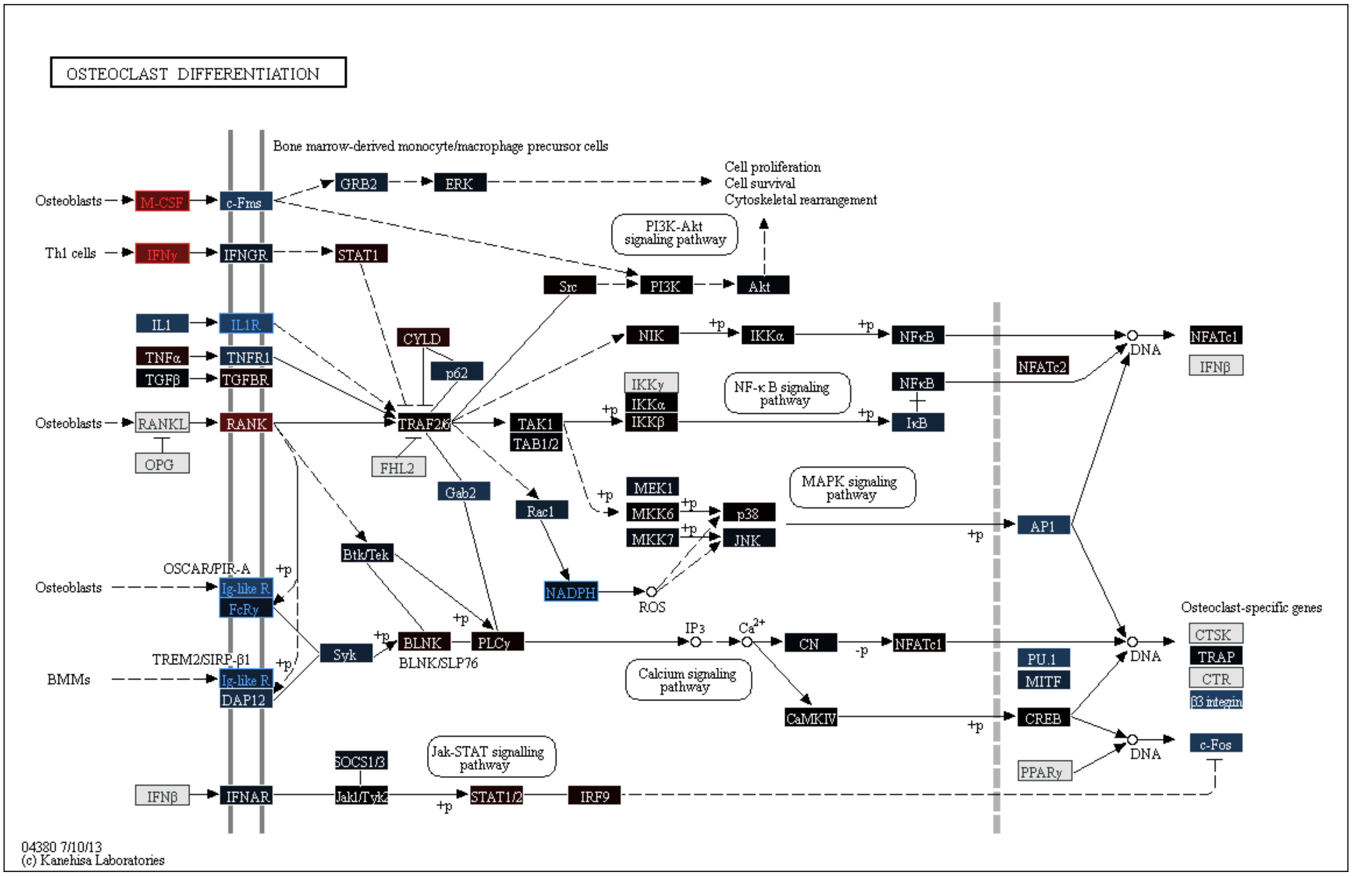

Supplementary Figure 27: KEGG Pathway Map - Osteoclast differentiation (RNA-Seq, Day 28 post-second vaccination). Node color gradient encodes fold change from pre-vaccination (for multi-gene pathway nodes the median fold change is used). In red: up-regulated compared to pre-vaccination, in blue: down-regulated compared to pre-vaccination. In black: fold change close to 1, in dark grey: genes filtered out due to low overall expression, light grey: gene missing database mapping, white: non-human gene. DE genes are highlighted using red (significantly up-regulated) and blue (significantly down-regulated) node label and border colors.

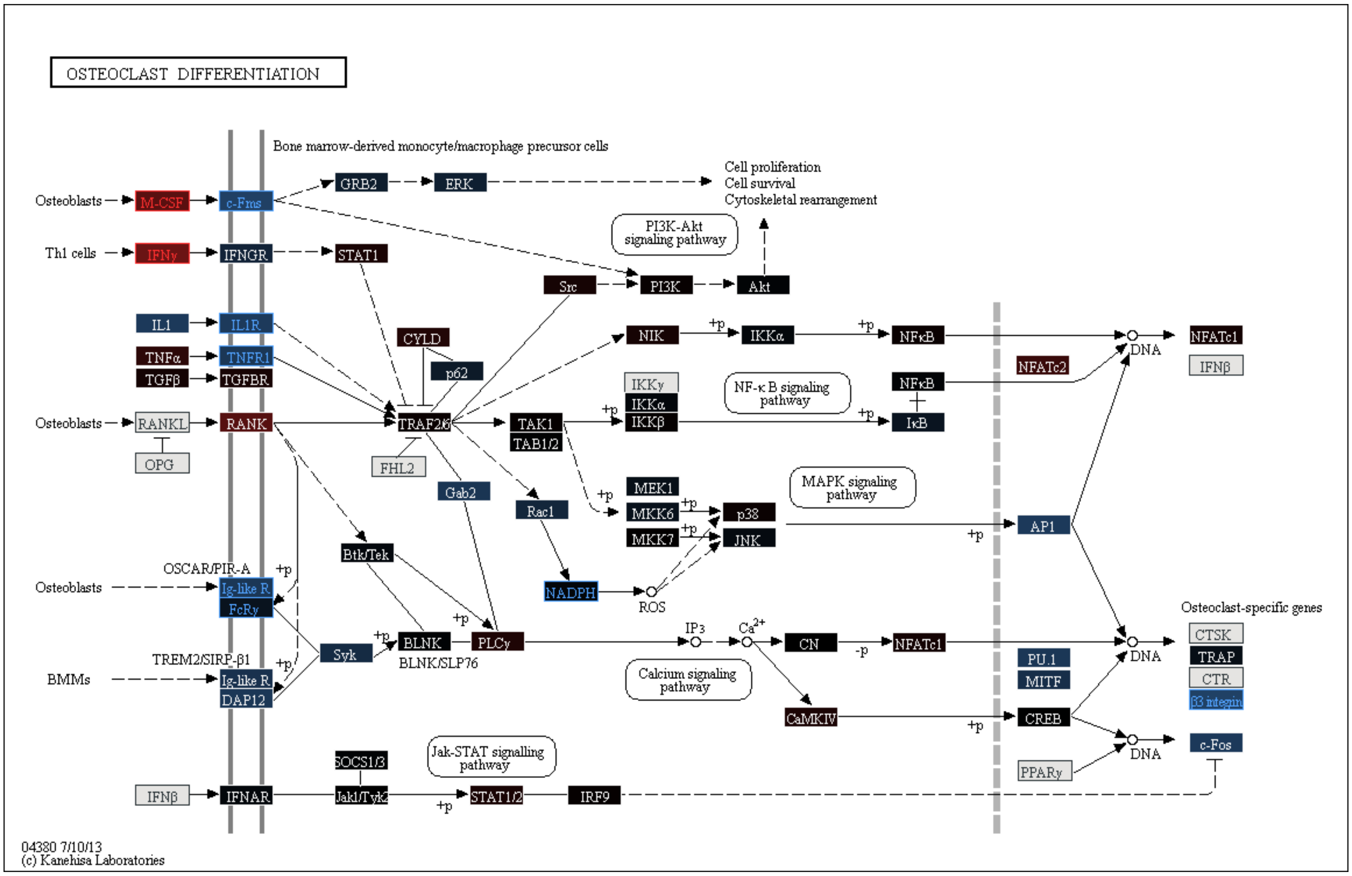

Supplementary Figure 28: KEGG Pathway Map - Phagosome (RNA-Seq, Day 28 post-second vaccination). Node color gradient encodes fold change from pre-vaccination (for multi-gene pathway nodes the median fold change is used). In red: up-regulated compared to pre-vaccination, in blue: down-regulated compared to pre-vaccination. In black: fold change close to 1, in dark grey: genes filtered out due to low overall expression, light grey: gene missing database mapping, white: non-human gene. DE genes are highlighted using red (significantly up-regulated) and blue (significantly down-regulated) node label and border colors.

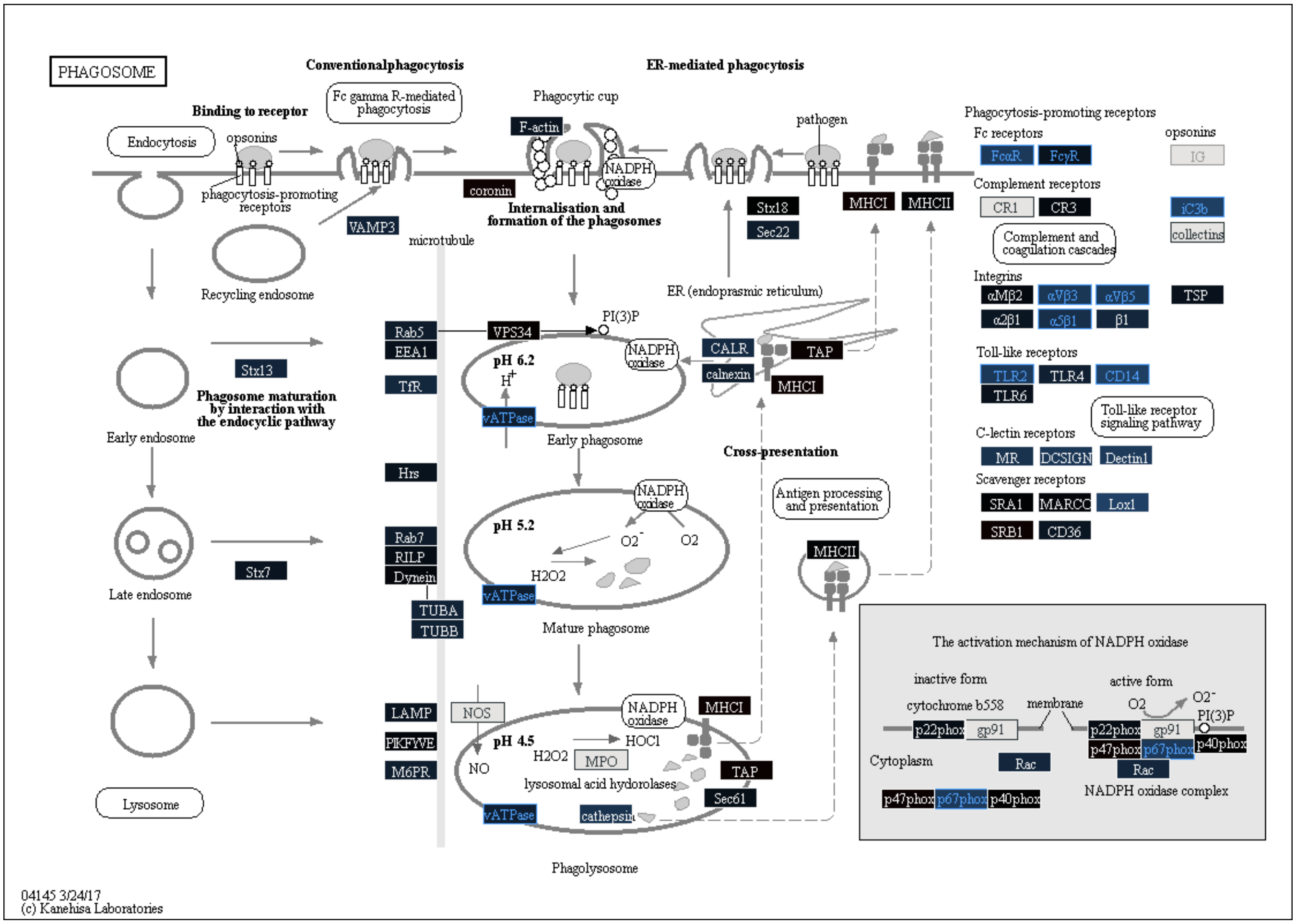

Supplementary Figure 29: KEGG Pathway Map - Proteoglycans in cancer (RNA-Seq, Day 14 post-second vaccination). Node color gradient encodes fold change from pre-vaccination (for multi-gene pathway nodes the median fold change is used). In red: up-regulated compared to pre-vaccination, in blue: down-regulated compared to pre-vaccination. In black: fold change close to 1, in dark grey: genes filtered out due to low overall expression, light grey: gene missing database mapping, white: non-human gene. DE genes are highlighted using red (significantly up-regulated) and blue (significantly down-regulated) node label and border colors.

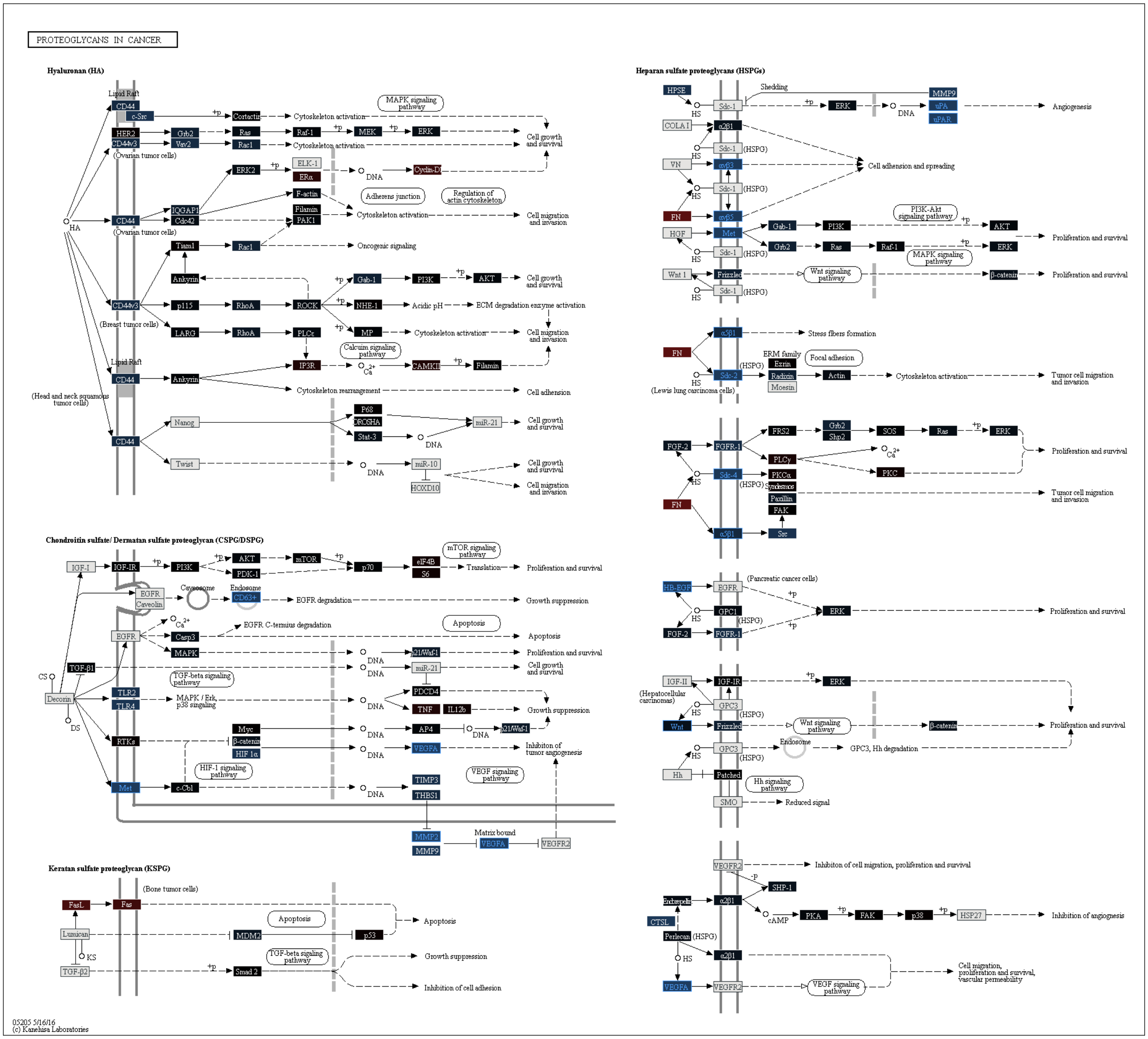

Supplementary Figure 30: KEGG Pathway Map - Proteoglycans in cancer (RNA-Seq, Day 28 post-second vaccination). Node color gradient encodes fold change from pre-vaccination (for multi-gene pathway nodes the median fold change is used). In red: up-regulated compared to pre-vaccination, in blue: down-regulated compared to pre-vaccination. In black: fold change close to 1, in dark grey: genes filtered out due to low overall expression, light grey: gene missing database mapping, white: non-human gene. DE genes are highlighted using red (significantly up-regulated) and blue (significantly down-regulated) node label and border colors.

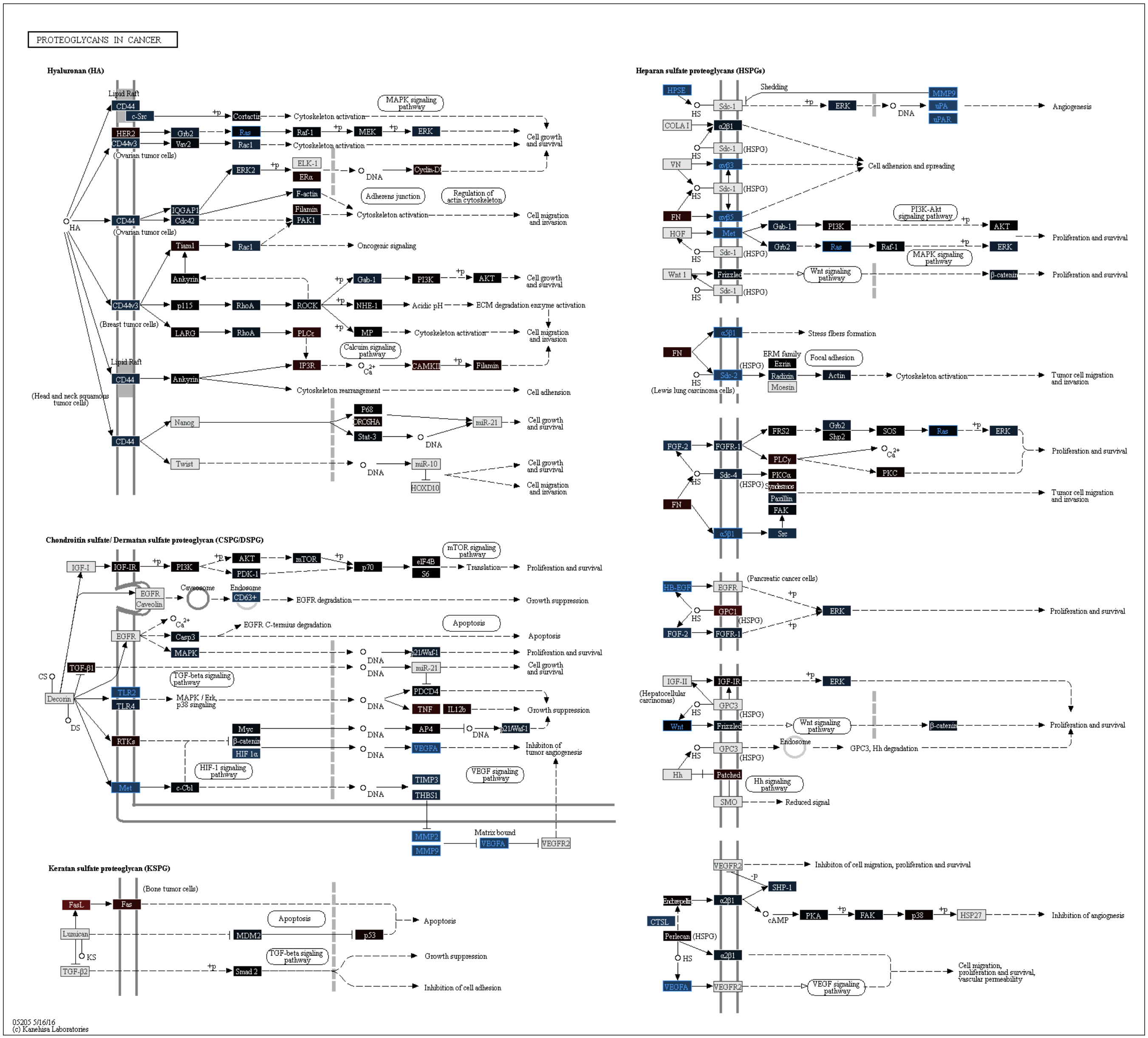

Supplementary Figure 31: KEGG Pathway Map - Rheumatoid arthritis (RNA-Seq, Day 28 post-second vaccination). Node color gradient encodes fold change from pre-vaccination (for multi-gene pathway nodes the median fold change is used). In red: up-regulated compared to pre-vaccination, in blue: down-regulated compared to pre-vaccination. In black: fold change close to 1, in dark grey: genes filtered out due to low overall expression, light grey: gene missing database mapping, white: non-human gene. DE genes are highlighted using red (significantly up-regulated) and blue (significantly down-regulated) node label and border colors.

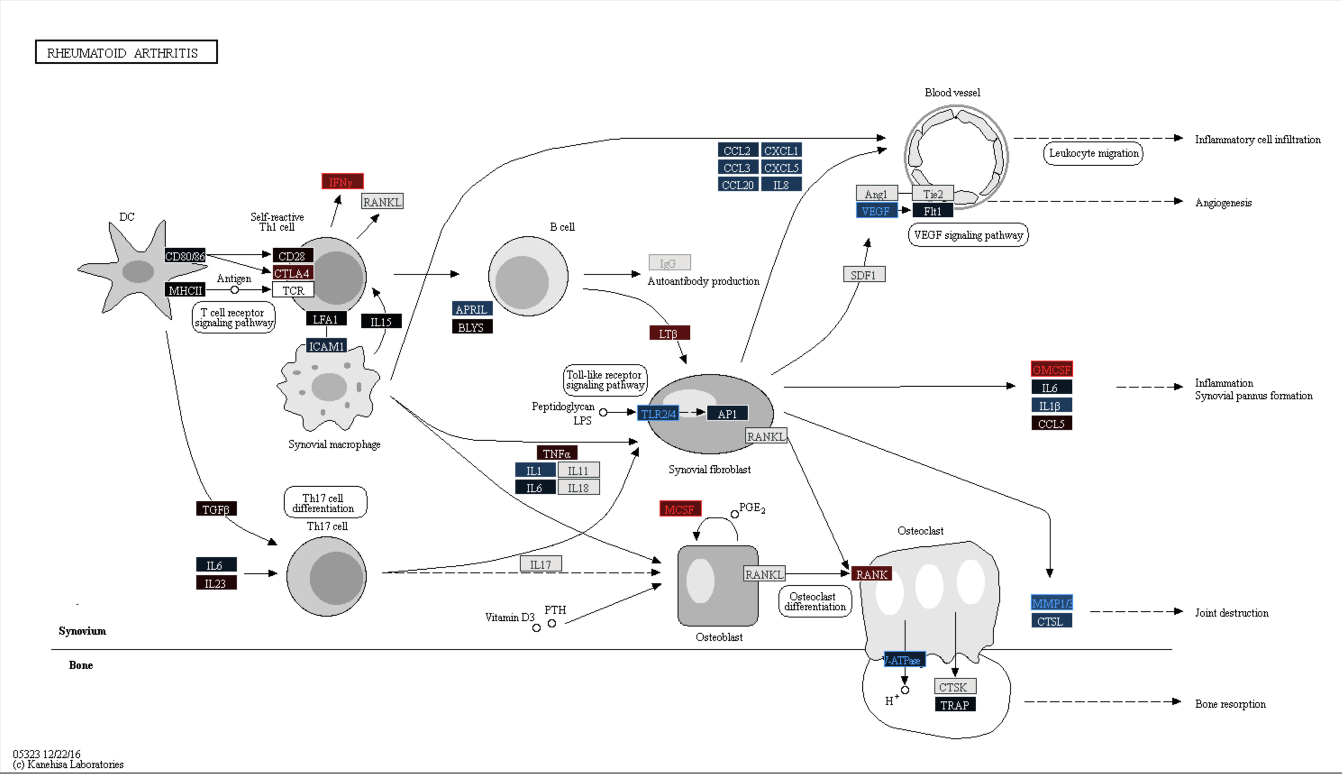

Supplementary Figure 32: KEGG Pathway Map - Staphylococcus aureus infection (RNA-Seq, Day 14 post-second vaccination). Node color gradient encodes fold change from pre-vaccination (for multi-gene pathway nodes the median fold change is used). In red: up-regulated compared to pre-vaccination, in blue: down-regulated compared to pre-vaccination. In black: fold change close to 1, in dark grey: genes filtered out due to low overall expression, light grey: gene missing database mapping, white: non-human gene. DE genes are highlighted using red (significantly up-regulated) and blue (significantly down-regulated) node label and border colors.

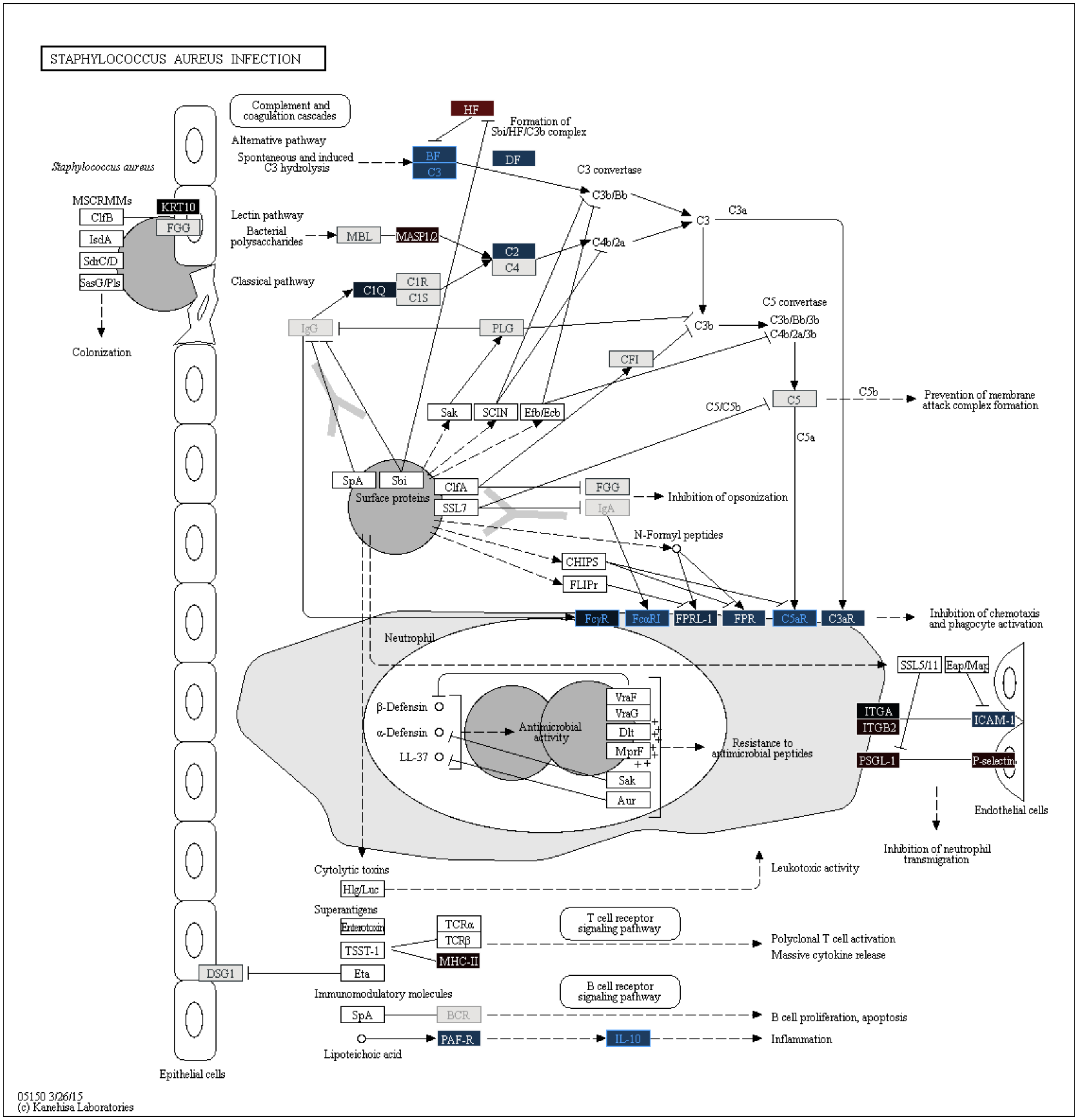

Supplementary Figure 33: KEGG Pathway Map - Staphylococcus aureus infection (RNA-Seq, Day 28 post-second vaccination). Node color gradient encodes fold change from pre-vaccination (for multi-gene pathway nodes the median fold change is used). In red: up-regulated compared to pre-vaccination, in blue: down-regulated compared to pre-vaccination. In black: fold change close to 1, in dark grey: genes filtered out due to low overall expression, light grey: gene missing database mapping, white: non-human gene. DE genes are highlighted using red (significantly up-regulated) and blue (significantly down-regulated) node label and border colors.

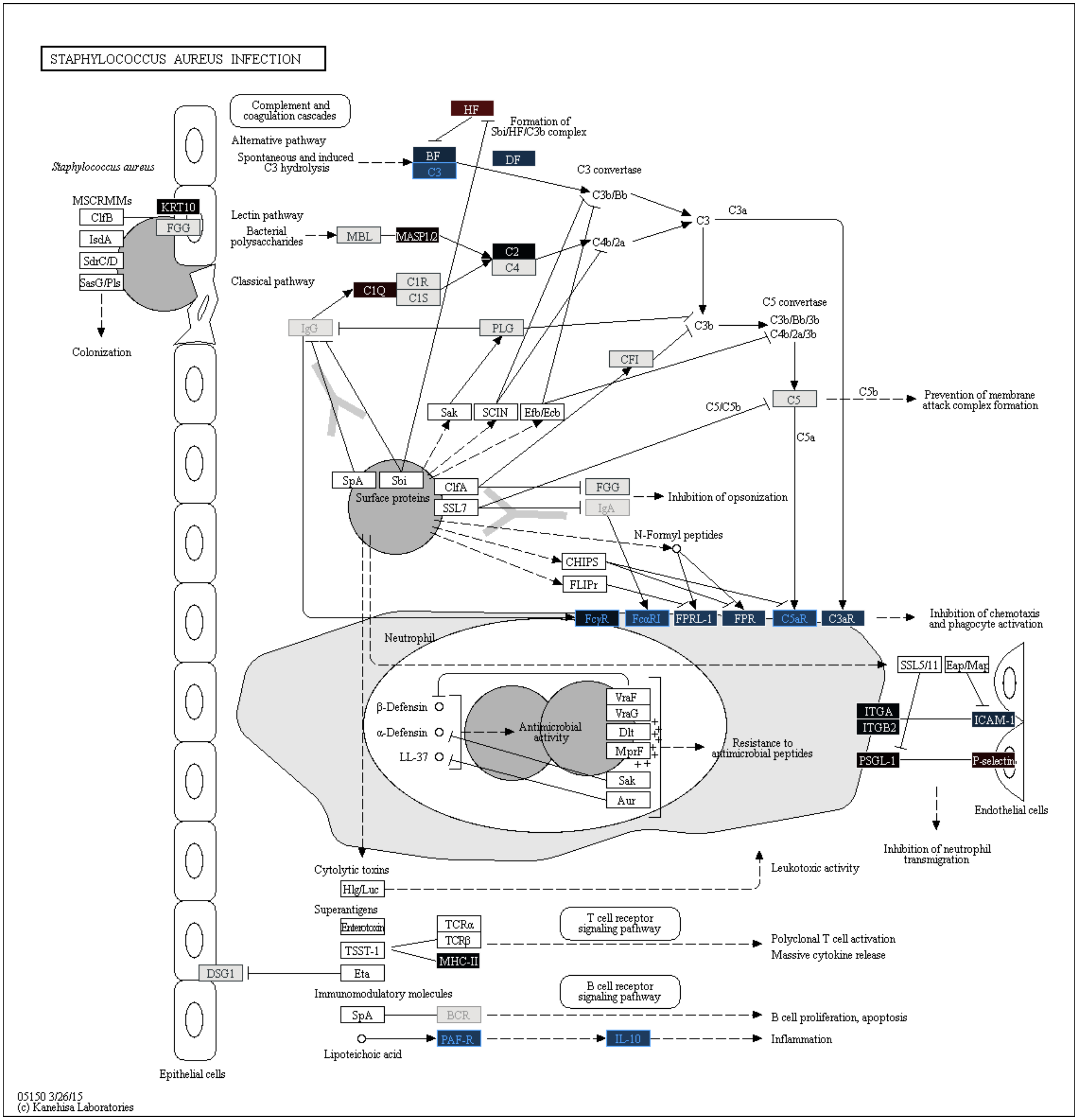

Supplementary Figure 34: KEGG Pathway Map - Tuberculosis (RNA-Seq, Day 14 post-second vaccination). Node color gradient encodes fold change from pre-vaccination (for multi-gene pathway nodes the median fold change is used). In red: up-regulated compared to pre-vaccination, in blue: down-regulated compared to pre-vaccination. In black: fold change close to 1, in dark grey: genes filtered out due to low overall expression, light grey: gene missing database mapping, white: non-human gene. DE genes are highlighted using red (significantly up-regulated) and blue (significantly down-regulated) node label and border colors.

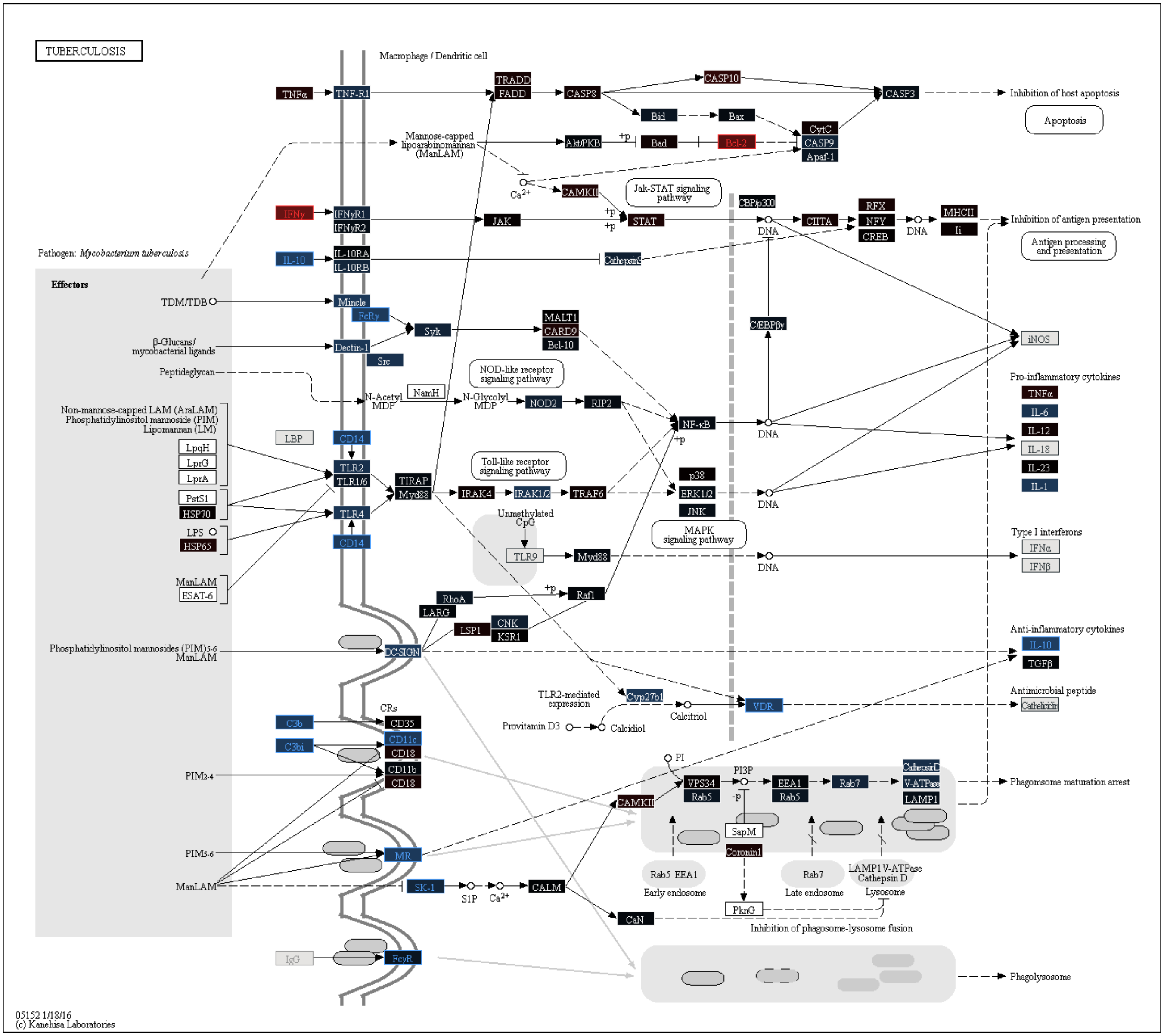

Supplementary Figure 35: KEGG Pathway Map - Tuberculosis (RNA-Seq, Day 28 post-second vaccination). Node color gradient encodes fold change from pre-vaccination (for multi-gene pathway nodes the median fold change is used). In red: up-regulated compared to pre-vaccination, in blue: down-regulated compared to pre-vaccination. In black: fold change close to 1, in dark grey: genes filtered out due to low overall expression, light grey: gene missing database mapping, white: non-human gene. DE genes are highlighted using red (significantly up-regulated) and blue (significantly down-regulated) node label and border colors.

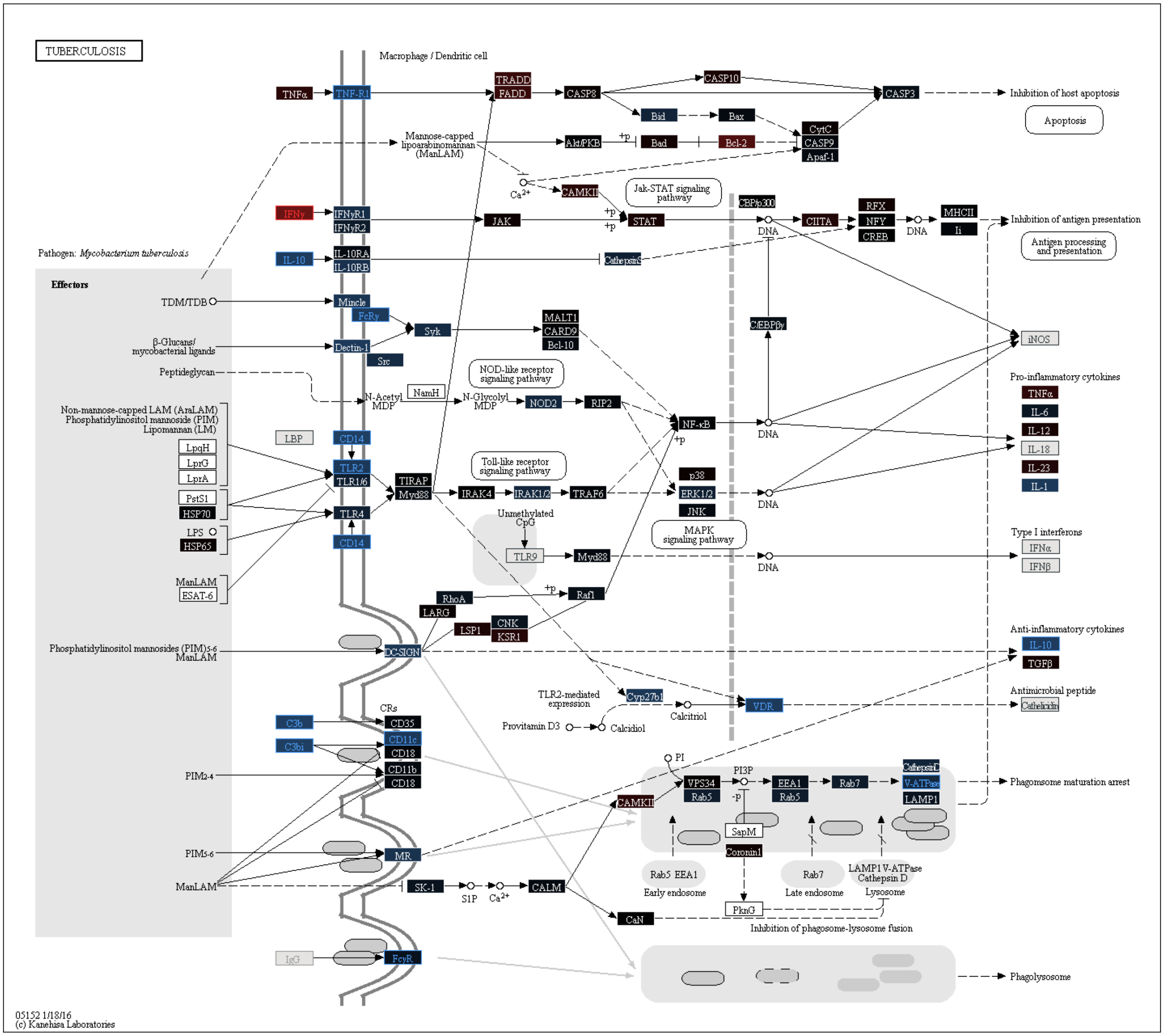

Supplementary Figure 36: Pathway enrichment analysis Venn diagrams summarizing overlap in significantly enriched gene sets between post-vaccination days (RNA-Seq). To determine significantly enriched gene sets, DE genes were used. Timepoints day 14 and day 28 refer to days 14 and 28 post-second vaccination.

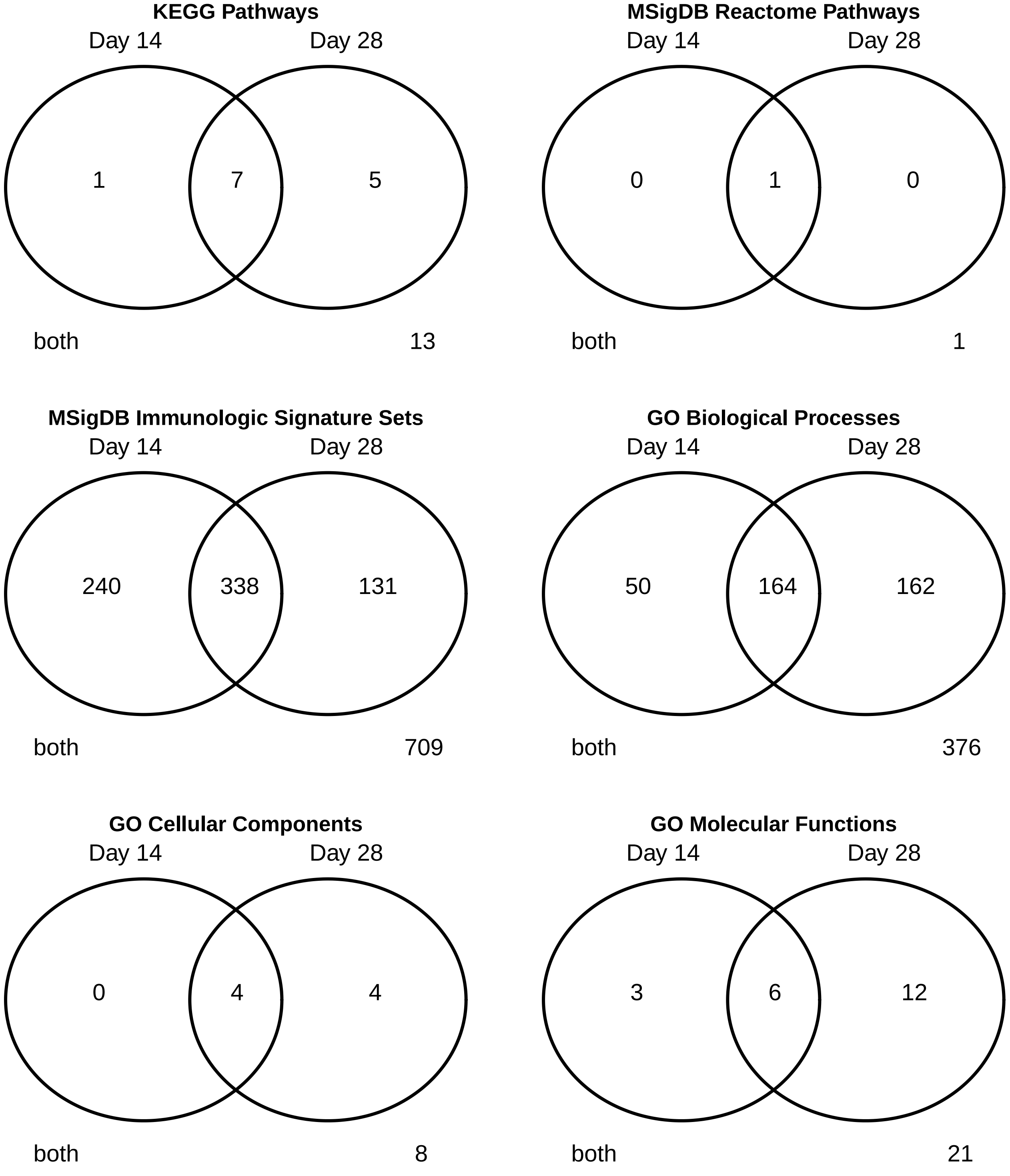

Supplementary Figure 37: Scatterplots that summarize the correlation between peak log_2 Inverse Caspase-3 Levels and log_2 fold change response for genes identified using regularized linear regression (RNA-Seq, Day 14 post-second vaccination). Sorted by absolute descending regularized linear regression coefficient. The green solid trend line represents a locally weighted regression fit.

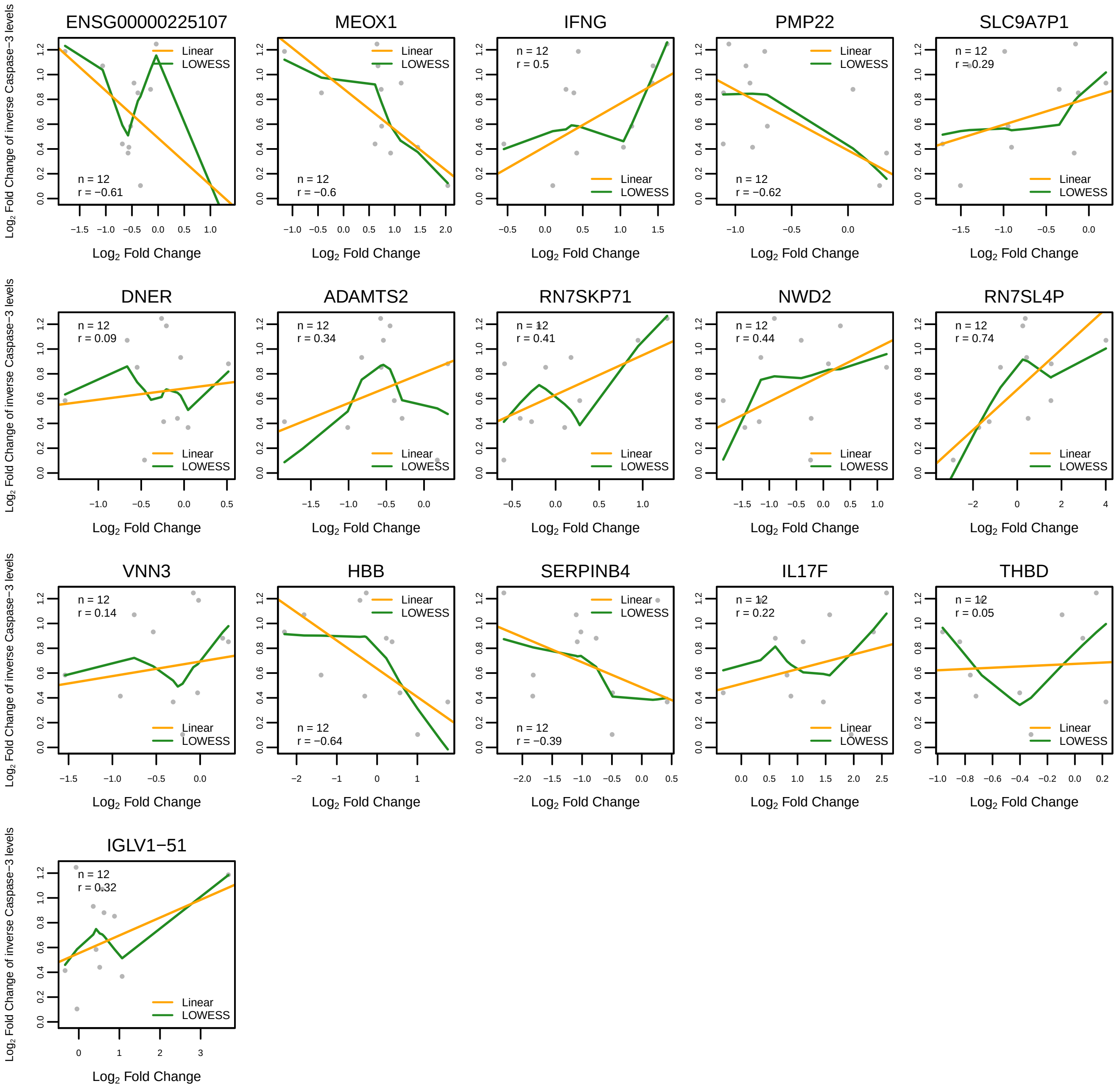

Supplementary Figure 38: Canonical correlation plots summarizing gene responses best correlated with changes in proliferating cytokine producing T-cell responses (RNA-Seq, Day 14 post-second vaccination). In brown: proliferating cytokine producing T-cell variables, red/blue: gene variables color-coded by baseline log fold change (up/down), +/- prefix: SDEG yes/no, colored-dots: pathway class, grey lines: robust clusters.

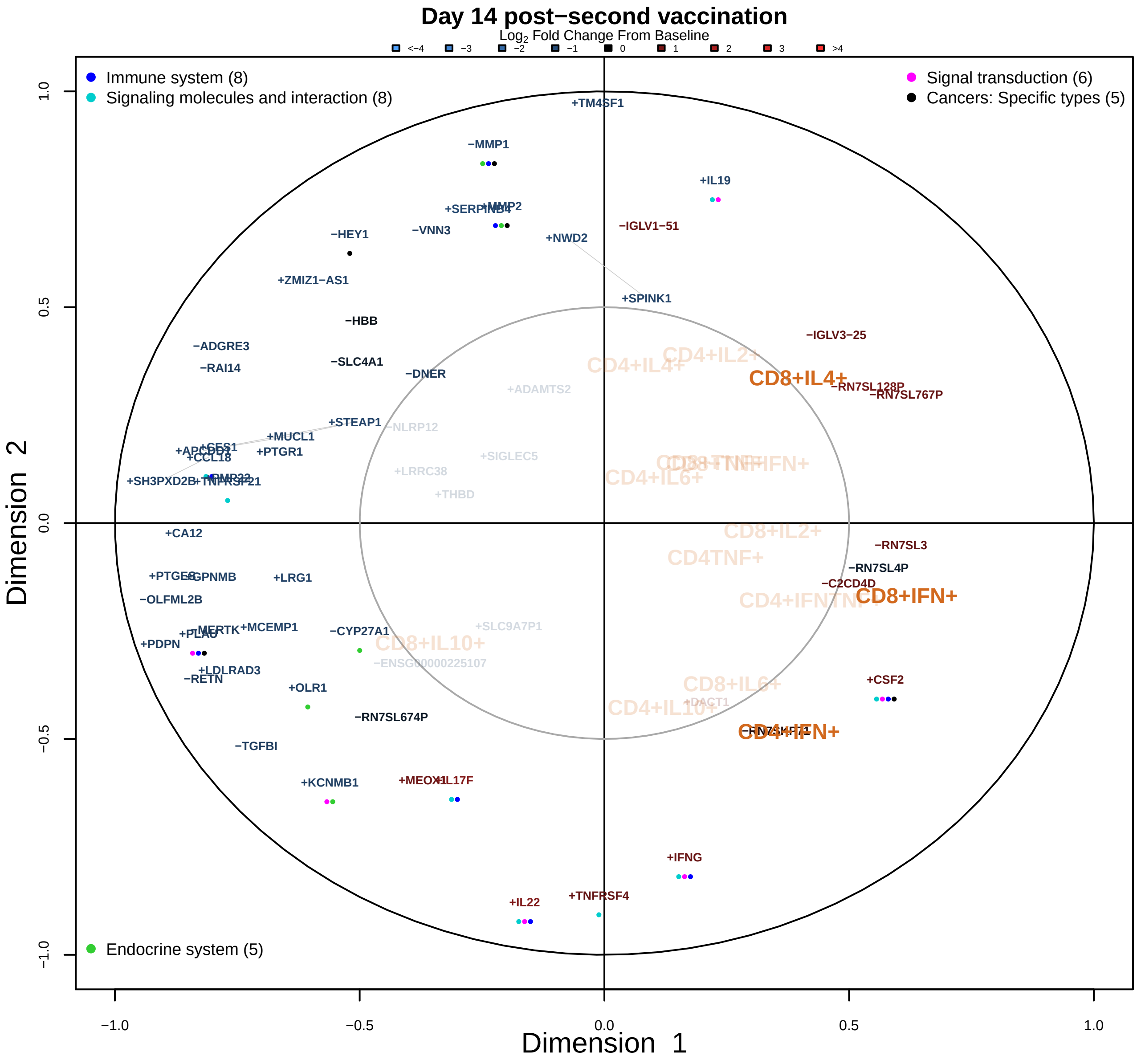

CD4+IL6+ = CD4+/CFSElo/IL−6+
CD4+IL10+ = CD4+/CFSElo/IL−10+
CD4+IL6IL10+ = CD4+/CFSElo/IL−6/IL−10+
CD4+IFN+ = CD4+/CFSElo/IFN+
CD4TNF+ = CD4/CFSElo/TNF+
CD4+IFNTNF+ = CD4+/CFSElo/IFN/TNF+
CD4+IL4+ = CD4+/CFSElo/IL−4+
CD4+IL2+ = CD4+/CFSElo/IL−2+
CD4+IL4IL2+ = CD4+/CFSElo/IL−4/IL−2+
CD8+IL6+ = CD8+/CFSElo/IL−6+
CD8+IL10+ = CD8+/CFSElo/IL−10+
CD8+IL10IL6+ = CD8+/CFSElo/IL−10/IL−6+
CD8+IFN+ = CD8+/CFSElo/IFN+
CD8+TNF+ = CD8+/CFSElo/TNF+
CD8+TNFIFN+ = CD8+/CFSElo/TNF/IFN+
CD8+IL4+ = CD8+/CFSElo/IL−4+
CD8+IL2+ = CD8+/CFSElo/IL−2+
CD8+IL4IL2+ = CD8+/CFSElo/IL−4/IL−2+

Supplementary Figure 39: Canonical correlation plots summarizing gene responses best correlated with changes in proliferating cytokine producing T-cell responses (RNA-Seq, Day 14 post-second vaccination). In brown: proliferating cytokine producing T-cell variables, red/blue: gene variables color-coded by baseline log fold change (up/down), +/- prefix: SDEG yes/no, colored-dots: pathway class, grey lines: robust clusters.

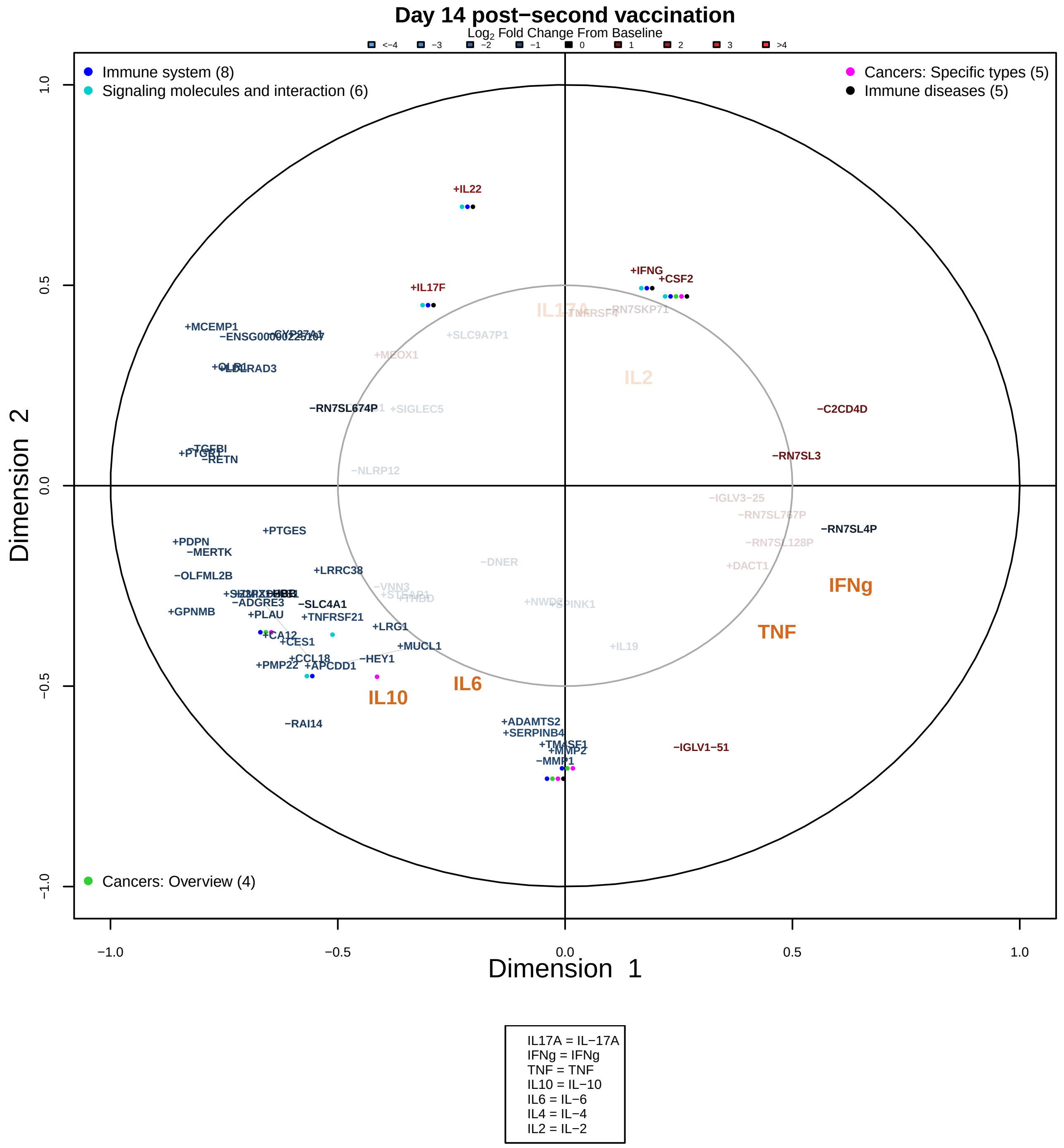

Supplementary Figure 40: Canonical correlation plots summarizing gene responses best correlated with changes in cytokine responses (RNA-Seq, Day 28 post-second vaccination). In brown: cytokine variables, red/blue: gene variables color-coded by baseline log fold change (up/down), +/- prefix: SDEG yes/no, colored-dots: pathway class, grey lines: robust clusters.

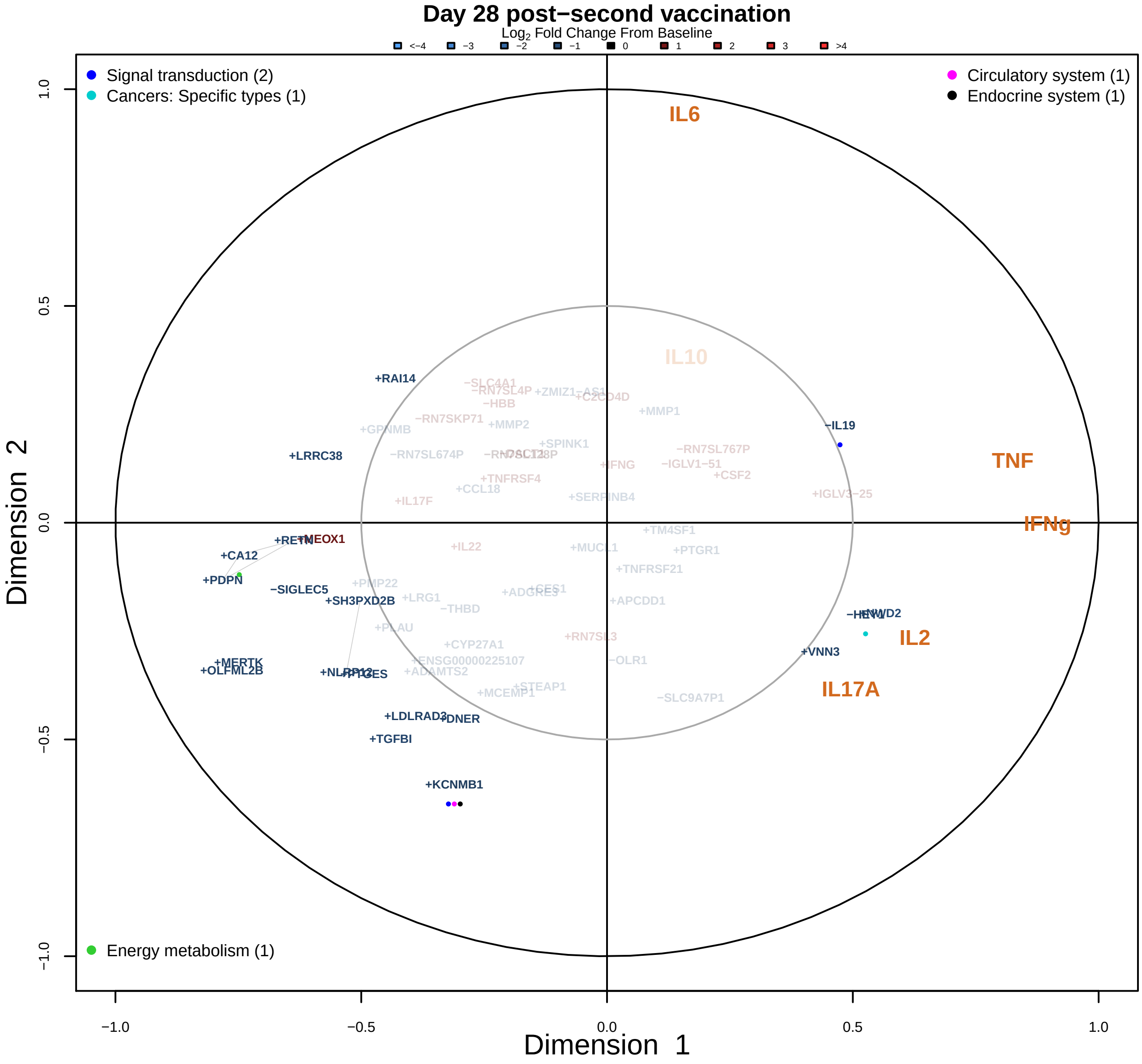

IL17A = IL−17A
IFNg = IFNg
TNF = TNF
IL10 = IL−10
IL6 = IL−6
IL4 = IL−4
IL2 = IL−2

Tables

Supplementary Table 1: Summary of categorical demographic and baseline characteristics for the immunogenicity analysis population. Three of the 16 subjects only consented to deidentified future use. As a consequence, they are missing from this summary.

Variable	Characteristic	N	%
**Sex**	Male	10	77
	Female	3	23
**Race**	American Indian or Native Alaskan	1	8
	Asian	1	8
	Native Hawaiian or Pacific Islander	0	0
	Black or African American	1	8
	White	10	77
	Unknown	0	0
	Multi-Race	0	0
**Ethnicity**	Not Hispanic or Latino	13	100

Supplementary Table 2: Summary of age demographic characteristics for the immunogenicity analysis population. Three of the 16 subjects only consented to deidentified future use. As a consequence, they are missing from this summary.

Statistic	
**Mean**	30.3
**Standard Deviation**	6.8
**Median**	29
**Minimum**	21
**Maximum**	42

Supplementary Table 3: Summary of categorical demographic and baseline characteristics for the transcriptomics analysis population. Two of the 12 subjects only consented to deidentified future use. As a consequence, they are missing from this summary.

Variable	Characteristic	N	%
**Sex**	Male	7	70
	Female	3	30
**Race**	American Indian or Native Alaskan	0	0
	Asian	1	10
	Native Hawaiian or Pacific Islander	0	0
	Black or African American	0	0
	White	9	90
	Unknown	0	0
	Multi-Race	0	0
**Ethnicity**	Not Hispanic or Latino	10	100

Supplementary Table 4: Summary of age demographic characteristics for the transcriptomics analysis population. Two of the 12 subjects only consented to deidentified future use. As a consequence, they are missing from this summary.

Statistic	
**Mean**	30.4
**Standard Deviation**	6.2
**Median**	28
**Minimum**	23
**Maximum**	42

Supplementary Table 5: Summaries of inverse normalized anti-V-antigen antibody caspase-3 levels by study visit day and treatment group

	**Flagellin/F1/V 6μg (N=8)**	**Flagellin/F1/V 10μg (N=8)**	**Combined (N=16)**
**Study Visit Day / Statistic**			
**Day 0 Pre-Vaccination 1**			
n	8	8	16
Minimum	0.935	1.611	0.935
Q1	1.37	2.02	1.76
Median (95% CI)	1.929 (1.027, 2.59)	2.49 (1.851, 3.326)	2.079 (1.731, 2.679)
MAD	0.82	1.06	0.73
Q3	2.2	3.3	2.65
Maximum	2.841	3.38	3.38
**Day 14 Post-Vaccination 2**			
n	8	8	16
Minimum	1.923	1.572	1.572
Q1	2.06	2.54	2.06
Median (95% CI)	2.341 (1.963, 3.182)	3.206 (1.89, 4.479)	2.715 (2.088, 3.424)
MAD	0.52	1.87	1.02
Q3	2.8	4.46	3.34
Maximum	3.663	5.73	5.73
**Day 28 Post-Vaccination 2**			
n	8	7	15
Minimum	1.921	2.414	1.921
Q1	2.56	4.03	2.77
Median (95% CI)	2.898 (1.959, 3.29)	4.575 (3.533, 6.103)	3.29 (2.764, 4.575)
MAD	0.49	1.54	1.3
Q3	3.19	5.52	4.55
Maximum	3.858	6.159	6.159

Supplementary Table 6: Summaries of the fold change in inverse normalized anti-V-antigen antibody caspase-3 levels by study visit day and treatment group

	**Flagellin/F1/V 6μg (N=8)**	**Flagellin/F1/V 10μg (N=8)**	**Combined (N=16)**
**Study Visit Day / Statistic**			
**Day 14 Post-Vaccination 2 vs. Pre-Vaccination 1**			
n	8	8	16
Minimum	0.806	0.816	0.806
Q1	1.23	0.92	1.12
Median (95% CI)	1.288 (1.182, 2.1)	1.242 (0.849, 2.139)	1.288 (1.173, 1.873)
MAD	0.44	0.51	0.58
Q3	1.93	1.56	1.93
Maximum	2.151	2.276	2.276
**Day 28 Post-Vaccination 2 vs. Pre-Vaccination 1**			
n	8	7	15
Minimum	1.075	1.357	1.075
Q1	1.29	1.45	1.38
Median (95% CI)	1.609 (1.158, 2.054)	1.806 (1.404, 1.878)	1.753 (1.357, 1.878)
MAD	0.55	0.45	0.45
Q3	1.94	1.86	1.89
Maximum	2.608	2.373	2.608

Supplementary Table 7: Spearman correlation between fold change in ELISA IgG and change in protective antibody response

ELISA Results		Caspase-3 Results	Spearman Correlation (p-value) [n]
**ELISA IgG titer (F1 antigen)**	Log_2 fold change in ELISA IgG titer (Day 14 post-second vaccination)	Log_2 fold change in normalized anti-V-antigen antibody caspase-3 levels (Day 14 post-second vaccination)	0.165 (0.5729) [14]
	Log_2 fold change in ELISA IgG titer (Day 28 post-second vaccination)	Log_2 fold change in normalized anti-V-antigen antibody caspase-3 levels (Day 28 post-second vaccination)	0.2 (0.493) [14]
**ELISA IgG concentration (F1 antigen)**	Log_2 fold change in ELISA IgG conc (Day 14 post-second vaccination)	Log_2 fold change in normalized anti-V-antigen antibody caspase-3 levels (Day 14 post-second vaccination)	0.095 (0.7469) [14]
	Log_2 fold change in ELISA IgG conc (Day 28 post-second vaccination)	Log_2 fold change in normalized anti-V-antigen antibody caspase-3 levels (Day 28 post-second vaccination)	0.151 (0.6073) [14]
**ELISA IgG titer (V antigen)**	Log_2 fold change in ELISA IgG titer (Day 14 post-second vaccination)	Log_2 fold change in normalized anti-V-antigen antibody caspase-3 levels (Day 14 post-second vaccination)	0.305 (0.2882) [14]
	Log_2 fold change in ELISA IgG titer (Day 28 post-second vaccination)	Log_2 fold change in normalized anti-V-antigen antibody caspase-3 levels (Day 28 post-second vaccination)	0.534 (0.0492) [14]
**ELISA IgG concentration (V antigen)**	Log_2 fold change in ELISA IgG conc (Day 14 post-second vaccination)	Log_2 fold change in normalized anti-V-antigen antibody caspase-3 levels (Day 14 post-second vaccination)	0.253 (0.3828) [14]
	Log_2 fold change in ELISA IgG conc (Day 28 post-second vaccination)	Log_2 fold change in normalized anti-V-antigen antibody caspase-3 levels (Day 28 post-second vaccination)	0.521 (0.0558) [14]

Supplementary Table 8: Wilcoxon Signed Rank Test for comparing proliferating and cytokine producing T cell and cytokine stimulation index levels at Day 14 or Day 28 post-second vaccination with pre-vaccination (F1/V 10ug/ml, all treatment groups)

	**Day 14 Post-Vaccination 2 vs. Pre-Vaccination 1**				**Day 28 Post-Vaccination 2 vs. Pre-Vaccination 1**			
	**n**	**Wilcoxon Statistic**	**P-Value**	**Median Fold Change**	**n**	**Wilcoxon Statistic**	**P-Value**	**Median Fold Change**
**Proliferating Cytokine Producing T-Cells**								
CD4+/CFSElo/IL-6+	15	40	0.4513	1	14	46	0.7148	1.01
CD4+/CFSElo/IL-10+	15	31	0.107	2.12	14	20	0.0419	7.43
CD4+/CFSElo/IL-6/IL-10+	15	11	>0.9999	1	14	12	0.8339	1
CD4+/CFSElo/IFNγ+	15	34	0.1514	2.01	14	33	0.2412	1.6
CD4/CFSElo/TNF+	15	37	0.2078	1.48	14	40	0.4631	1.22
CD4+/CFSElo/IFN/TNFα+	15	36	0.1876	1.54	14	21	0.0494	1.68
CD4+/CFSElo/IL-4+	15	27	0.2084	1	14	8	0.0294	2.02
CD4+/CFSElo/IL-2+	15	32	0.3636	1.09	14	67	0.1422	0.3
CD4+/CFSElo/IL-4/IL-2+	15	1	>0.9999	1	14	0	0.3711	1
CD8+/CFSElo/IL-6+	15	40	0.4513	1.78	14	35	0.7837	1.19
CD8+/CFSElo/IL-10+	15	60	>0.9999	1.51	14	61	0.6257	0.9
CD8+/CFSElo/IL-10/IL-6+	15	0	0.1814	1	14	2	>0.9999	1
CD8+/CFSElo/IFNγ+	15	50	0.5995	1.05	14	38	0.391	1.44
CD8+/CFSElo/TNFα+	15	84	0.1876	0.82	14	59	0.7148	0.61
CD8+/CFSElo/TNF/IFNγ+	15	62	0.9341	1.09	14	49	0.8552	0.85
CD8+/CFSElo/IL-4+	15	62	0.5721	0.77	14	44	0.7241	1.04
CD8+/CFSElo/IL-2+	15	33	0.1354	1.78	14	34	0.442	1.44
CD8+/CFSElo/IL-4/IL-2+	15	12	0.2807	1	14	10	0.5541	1
**Cytokines**								
IL-17A	15	20	0.0806	1.08	14	26	0.3268	1.12
IFNγ	15	18	0.0151	1.34	14	40	0.4631	1
TNFα	15	83	0.2078	0.72	14	73	0.2166	0.5
IL-10	15	68	0.6788	1.01	14	72	0.2412	0.89
IL-6	15	60	>0.9999	0.99	14	47	0.7609	1.18
IL-4	15	0	NA	1	14	0	NA	1
IL-2	15	75	0.1673	0.96	14	52	0.3268	1

Supplementary Table 9: Spearman correlation between fold change in proliferating and cytokine producing CD4+ T cell stimulation index and change in protective antibody response

	**Spearman Correlation (p-value) [n]**			
	**CD4+/CFSElo/IL-6+ T-cells (F1/V)**		**CD4+/CFSElo/IFNγ+ T-cells (F1/V)**	
	**Log_2 fold change in the stimulation index of proliferating cells (Day 14 post-second vaccination)**	**Log_2 fold change in the stimulation index of proliferating cells (Day 28 post-second vaccination)**	**Log_2 fold change in the stimulation index of proliferating cells (Day 14 post-second vaccination)**	**Log_2 fold change in the stimulation index of proliferating cells (Day 28 post-second vaccination)**
**Log_2 fold change in inverse normalized anti-V-antigen antibody Caspase-3 levels (Day 14 post-second vaccination)**	-0.571 (0.0261) [15]	-0.305 (0.2882) [14]	-0.232 (0.4051) [15]	-0.345 (0.2269) [14]
**Log_2 fold change in inverse normalized anti-V-antigen antibody Caspase-3 levels (Day 28 post-second vaccination)**	-0.138 (0.6369) [14]	-0.169 (0.563) [14]	0.244 (0.4006) [14]	-0.095 (0.7479) [14]

Supplementary Table 10: Genes differentially expressed at day 14 post-second vaccination compared to day 0 (RNA-Seq). Sorted by descending absolute log_2 fold change (Day 14 post-second vaccination vs. Day 0). Gene model summaries and annotations are based on Ensembl Version 90 (August 2017).

**Ensembl Gene ID**	**Ensembl Gene Name**	**Ensembl Gene Description**	**Gene Type**	**Log_2 Fold Change (Day 14 vs. Day 0)**	**Average Log_2 CPM**	**Likelihood Ratio Test Statistic**	**P-Value**	**FDR Adjusted P-Value**
ENSG00000112116	IL17F	interleukin 17F [Source:HGNC Symbol;Acc:HGNC:16404]	protein coding	1.23	3.96	48.43	<0.0001	<0.0001
ENSG00000127318	IL22	interleukin 22 [Source:HGNC Symbol;Acc:HGNC:14900]	protein coding	1.18	4.84	19.59	<0.0001	0.0016
ENSG00000198848	CES1	carboxylesterase 1 [Source:HGNC Symbol;Acc:HGNC:1863]	protein coding	-0.86	5.47	74.58	<0.0001	<0.0001
ENSG00000206073	SERPINB4	serpin family B member 4 [Source:HGNC Symbol;Acc:HGNC:10570]	protein coding	-0.84	4.44	17.90	<0.0001	0.0025
ENSG00000174145	NWD2	NACHT and WD repeat domain containing 2 [Source:HGNC Symbol;Acc:HGNC:29229]	protein coding	-0.77	2.49	15.28	<0.0001	0.0062
ENSG00000005102	MEOX1	mesenchyme homeobox 1 [Source:HGNC Symbol;Acc:HGNC:7013]	protein coding	0.74	1.35	19.81	<0.0001	0.0015
ENSG00000087245	MMP2	matrix metallopeptidase 2 [Source:HGNC Symbol;Acc:HGNC:7166]	protein coding	-0.70	2.60	11.50	0.0007	0.0254
ENSG00000111537	IFNG	interferon gamma [Source:HGNC Symbol;Acc:HGNC:5438]	protein coding	0.68	7.07	21.88	<0.0001	0.0008
ENSG00000164647	STEAP1	STEAP family member 1 [Source:HGNC Symbol;Acc:HGNC:11378]	protein coding	-0.67	2.06	12.69	0.0004	0.0172
ENSG00000105501	SIGLEC5	sialic acid binding Ig like lectin 5 [Source:HGNC Symbol;Acc:HGNC:10874]	protein coding	-0.66	1.79	24.93	<0.0001	0.0004
ENSG00000165617	DACT1	dishevelled binding antagonist of beta catenin 1 [Source:HGNC Symbol;Acc:HGNC:17748]	protein coding	0.65	2.32	23.28	<0.0001	0.0006
ENSG00000227825	SLC9A7P1	solute carrier family 9 member 7 pseudogene 1 [Source:HGNC Symbol;Acc:HGNC:32679]	transcribed processed pseudogene	-0.64	1.90	16.38	<0.0001	0.0044
ENSG00000146072	TNFRSF21	TNF receptor superfamily member 21 [Source:HGNC Symbol;Acc:HGNC:13469]	protein coding	-0.64	5.46	29.77	<0.0001	<0.0001
ENSG00000171236	LRG1	leucine rich alpha-2-glycoprotein 1 [Source:HGNC Symbol;Acc:HGNC:29480]	protein coding	-0.63	1.56	20.88	<0.0001	0.0012
ENSG00000136695	IL36RN	interleukin 36 receptor antagonist [Source:HGNC Symbol;Acc:HGNC:15561]	protein coding	-0.63	3.78	15.64	<0.0001	0.0058
ENSG00000142224	IL19	interleukin 19 [Source:HGNC Symbol;Acc:HGNC:5990]	protein coding	-0.63	4.27	12.12	0.0005	0.0204

Supplementary Table 10: Genes differentially expressed at day 14 post-second vaccination compared to day 0 (RNA-Seq). Sorted by descending absolute log_2 fold change (Day 14 post-second vaccination vs. Day 0). Gene model summaries and annotations are based on Ensembl Version 90 (August 2017) (continued).

Ensembl Gene ID	Ensembl Gene Name	Ensembl Gene Description	Gene Type	Log_2 Fold Change (Day 14 vs. Day 0)	Average Log_2 CPM	Likelihood Ratio Test Statistic	P-Value	FDR Adjusted P-Value
ENSG00000087116	ADAMTS2	ADAM metallopeptidase with thrombospondin type 1 motif 2 [Source:HGNC Symbol;Acc:HGNC:218]	protein coding	-0.63	1.64	15.78	<0.0001	0.0056
ENSG00000164266	SPINK1	serine peptidase inhibitor, Kazal type 1 [Source:HGNC Symbol;Acc:HGNC:11244]	protein coding	-0.61	1.55	13.55	0.0002	0.0121
ENSG00000183019	MCEMP1	mast cell expressed membrane protein 1 [Source:HGNC Symbol;Acc:HGNC:27291]	protein coding	-0.61	3.42	21.93	<0.0001	0.0008
ENSG00000179241	LDLRAD3	low density lipoprotein receptor class A domain containing 3 [Source:HGNC Symbol;Acc:HGNC:27046]	protein coding	-0.60	4.76	22.82	<0.0001	0.0007
ENSG00000162897	FCAMR	Fc fragment of IgA and IgM receptor [Source:HGNC Symbol;Acc:HGNC:24692]	protein coding	-0.59	2.71	13.87	0.0002	0.0109
ENSG00000275385	CCL18	C-C motif chemokine ligand 18 [Source:HGNC Symbol;Acc:HGNC:10616]	protein coding	-0.59	4.87	18.52	<0.0001	0.002
ENSG00000211890	IGHA2	immunoglobulin heavy constant alpha 2 (A2m marker) [Source:HGNC Symbol;Acc:HGNC:5479]	IG C gene	0.58	5.84	10.29	0.0013	0.038
ENSG00000172551	MUCL1	mucin like 1 [Source:HGNC Symbol;Acc:HGNC:30588]	protein coding	-0.57	3.41	19.40	<0.0001	0.0017
ENSG00000224596	ZMIZ1-AS1	ZMIZ1 antisense RNA 1 [Source:HGNC Symbol;Acc:HGNC:27433]	antisense RNA	-0.56	2.59	13.21	0.0003	0.0139
ENSG00000186827	TNFRSF4	TNF receptor superfamily member 4 [Source:HGNC Symbol;Acc:HGNC:11918]	protein coding	0.56	5.57	20.27	<0.0001	0.0014
ENSG00000140090	SLC24A4	solute carrier family 24 member 4 [Source:HGNC Symbol;Acc:HGNC:10978]	protein coding	-0.55	1.58	16.10	<0.0001	0.0049
ENSG00000109099	PMP22	peripheral myelin protein 22 [Source:HGNC Symbol;Acc:HGNC:9118]	protein coding	-0.55	4.90	17.90	<0.0001	0.0025
ENSG00000169245	CXCL10	C-X-C motif chemokine ligand 10 [Source:HGNC Symbol;Acc:HGNC:10637]	protein coding	0.54	5.81	11.19	0.0008	0.0285
ENSG00000213694	S1PR3	sphingosine-1-phosphate receptor 3 [Source:HGNC Symbol;Acc:HGNC:3167]	protein coding	-0.54	2.08	16.58	<0.0001	0.004
ENSG00000172322	CLEC12A	C-type lectin domain family 12 member A [Source:HGNC Symbol;Acc:HGNC:31713]	protein coding	-0.54	1.70	21.95	<0.0001	0.0008
ENSG00000074410	CA12	carbonic anhydrase 12 [Source:HGNC Symbol;Acc:HGNC:1371]	protein coding	-0.54	6.64	19.66	<0.0001	0.0016

Supplementary Table 10: Genes differentially expressed at day 14 post-second vaccination compared to day 0 (RNA-Seq). Sorted by descending absolute log_2 fold change (Day 14 post-second vaccination vs. Day 0). Gene model summaries and annotations are based on Ensembl Version 90 (August 2017) (continued).

**Ensembl Gene ID**	**Ensembl Gene Name**	**Ensembl Gene Description**	**Gene Type**	Log_2 **Fold Change (Day 14 vs. Day 0)**	**Average** Log_2 **CPM**	**Likelihood Ratio Test Statistic**	**P-Value**	**FDR Adjusted P-Value**
ENSG00000174705	SH3PXD2B	SH3 and PX domains 2B [Source:HGNC Symbol;Acc:HGNC:29242]	protein coding	-0.54	7.13	19.13	<0.0001	0.0017
ENSG00000136235	GPNMB	glycoprotein nmb [Source:HGNC Symbol;Acc:HGNC:4462]	protein coding	-0.54	3.08	14.84	0.0001	0.0074
ENSG00000145936	KCNMB1	potassium calcium-activated channel subfamily M regulatory beta subunit 1 [Source:HGNC Symbol;Acc:HGNC:6285]	protein coding	-0.53	2.14	16.66	<0.0001	0.0039
ENSG00000211640	IGLV6-57	immunoglobulin lambda variable 6-57 [Source:HGNC Symbol;Acc:HGNC:5927]	IG V gene	0.53	1.61	15.38	<0.0001	0.0059
ENSG00000143185	XCL2	X-C motif chemokine ligand 2 [Source:HGNC Symbol;Acc:HGNC:10646]	protein coding	0.52	2.77	14.21	0.0002	0.0095
ENSG00000173391	OLR1	oxidized low density lipoprotein receptor 1 [Source:HGNC Symbol;Acc:HGNC:8133]	protein coding	-0.52	6.11	10.08	0.0015	0.0408
ENSG00000164400	CSF2	colony stimulating factor 2 [Source:HGNC Symbol;Acc:HGNC:2434]	protein coding	0.52	3.29	14.26	0.0002	0.0093
ENSG00000165474	GJB2	gap junction protein beta 2 [Source:HGNC Symbol;Acc:HGNC:4284]	protein coding	-0.51	7.58	42.49	<0.0001	<0.0001
ENSG00000164125	FAM198B	family with sequence similarity 198 member B [Source:HGNC Symbol;Acc:HGNC:25312]	protein coding	-0.51	2.37	14.76	0.0001	0.0076
ENSG00000108342	CSF3	colony stimulating factor 3 [Source:HGNC Symbol;Acc:HGNC:2438]	protein coding	-0.51	7.90	16.76	<0.0001	0.0038
ENSG00000128342	LIF	LIF, interleukin 6 family cytokine [Source:HGNC Symbol;Acc:HGNC:6596]	protein coding	0.50	3.24	12.55	0.0004	0.018
ENSG00000154856	APCDD1	APC down-regulated 1 [Source:HGNC Symbol;Acc:HGNC:15718]	protein coding	-0.50	4.30	21.63	<0.0001	0.0009
ENSG00000169122	FAM110B	family with sequence similarity 110 member B [Source:HGNC Symbol;Acc:HGNC:28587]	protein coding	-0.50	1.83	14.48	0.0001	0.0086
ENSG00000169908	TM4SF1	transmembrane 4 L six family member 1 [Source:HGNC Symbol;Acc:HGNC:11853]	protein coding	-0.50	4.57	10.39	0.0013	0.0368
ENSG00000099985	OSM	oncostatin M [Source:HGNC Symbol;Acc:HGNC:8506]	protein coding	-0.50	7.69	21.96	<0.0001	0.0008
ENSG00000145649	GZMA	granzyme A [Source:HGNC Symbol;Acc:HGNC:4708]	protein coding	0.50	5.54	13.87	0.0002	0.0109

Supplementary Table 10: Genes differentially expressed at day 14 post-second vaccination compared to day 0 (RNA-Seq). Sorted by descending absolute log_2 fold change (Day 14 post-second vaccination vs. Day 0). Gene model summaries and annotations are based on Ensembl Version 90 (August 2017) (continued).

**Ensembl Gene ID**	**Ensembl Gene Name**	**Ensembl Gene Description**	**Gene Type**	**Log_2 Fold Change (Day 14 vs. Day 0)**	**Average Log_2 CPM**	**Likelihood Ratio Test Statistic**	**P-Value**	**FDR Adjusted P-Value**
ENSG00000128578	STRIP2	striatin interacting protein 2 [Source:HGNC Symbol;Acc:HGNC:22209]	protein coding	-0.49	3.49	24.18	<0.0001	0.0005
ENSG00000270550	IGHV3-30	immunoglobulin heavy variable 3-30 [Source:HGNC Symbol;Acc:HGNC:5591]	IG V gene	0.49	1.96	12.13	0.0005	0.0204
ENSG00000014257	ACPP	acid phosphatase, prostate [Source:HGNC Symbol;Acc:HGNC:125]	protein coding	-0.49	1.74	10.84	0.001	0.0316
ENSG00000179431	FJX1	four jointed box 1 [Source:HGNC Symbol;Acc:HGNC:17166]	protein coding	-0.49	4.12	13.29	0.0003	0.0135
ENSG00000114737	CISH	cytokine inducible SH2 containing protein [Source:HGNC Symbol;Acc:HGNC:1984]	protein coding	0.49	5.25	23.80	<0.0001	0.0005
ENSG00000211653	IGLV1-40	immunoglobulin lambda variable 1-40 [Source:HGNC Symbol;Acc:HGNC:5877]	IG V gene	0.48	2.59	9.99	0.0016	0.0424
ENSG00000260314	MRC1	mannose receptor C-type 1 [Source:HGNC Symbol;Acc:HGNC:7228]	protein coding	-0.48	1.77	12.85	0.0003	0.0161
ENSG00000162494	LRRC38	leucine rich repeat containing 38 [Source:HGNC Symbol;Acc:HGNC:27005]	protein coding	-0.48	2.05	14.85	0.0001	0.0074
ENSG00000148344	PTGES	prostaglandin E synthase [Source:HGNC Symbol;Acc:HGNC:9599]	protein coding	-0.48	6.55	10.60	0.0011	0.0345
ENSG00000143184	XCL1	X-C motif chemokine ligand 1 [Source:HGNC Symbol;Acc:HGNC:10645]	protein coding	0.47	2.22	11.00	0.0009	0.03
ENSG00000011422	PLAUR	plasminogen activator, urokinase receptor [Source:HGNC Symbol;Acc:HGNC:9053]	protein coding	-0.47	10.08	19.88	<0.0001	0.0015
ENSG00000183347	GBP6	guanylate binding protein family member 6 [Source:HGNC Symbol;Acc:HGNC:25395]	protein coding	0.47	1.44	12.64	0.0004	0.0175
ENSG00000136634	IL10	interleukin 10 [Source:HGNC Symbol;Acc:HGNC:5962]	protein coding	-0.47	6.07	17.84	<0.0001	0.0025
ENSG00000112394	SLC16A10	solute carrier family 16 member 10 [Source:HGNC Symbol;Acc:HGNC:17027]	protein coding	-0.47	6.86	21.42	<0.0001	0.001
ENSG00000102471	NDFIP2	Nedd4 family interacting protein 2 [Source:HGNC Symbol;Acc:HGNC:18537]	protein coding	0.47	3.57	21.27	<0.0001	0.001
ENSG00000112715	VEGFA	vascular endothelial growth factor A [Source:HGNC Symbol;Acc:HGNC:12680]	protein coding	-0.46	6.42	25.22	<0.0001	0.0004
ENSG00000018280	SLC11A1	solute carrier family 11 member 1 [Source:HGNC Symbol;Acc:HGNC:10907]	protein coding	-0.46	8.70	18.56	<0.0001	0.002

Supplementary Table 10: Genes differentially expressed at day 14 post-second vaccination compared to day 0 (RNA-Seq). Sorted by descending absolute log_2 fold change (Day 14 post-second vaccination vs. Day 0). Gene model summaries and annotations are based on Ensembl Version 90 (August 2017) (continued).

Ensembl Gene ID	Ensembl Gene Name	Ensembl Gene Description	Gene Type	Log_2 Fold Change (Day 14 vs. Day 0)	Average Log_2 CPM	Likelihood Ratio Test Statistic	P-Value	FDR Adjusted P-Value
ENSG00000252010	SCARNA5	small Cajal body-specific RNA 5 [Source:HGNC Symbol;Acc:HGNC:32561]	scaRNA	0.46	5.65	16.13	<0.0001	0.0048
ENSG00000134830	C5AR2	complement component 5a receptor 2 [Source:HGNC Symbol;Acc:HGNC:4527]	protein coding	-0.46	2.48	11.33	0.0008	0.0269
ENSG00000136689	IL1RN	interleukin 1 receptor antagonist [Source:HGNC Symbol;Acc:HGNC:6000]	protein coding	-0.46	8.69	22.26	<0.0001	0.0008
ENSG00000136379	ABHD17C	abhydrolase domain containing 17C [Source:HGNC Symbol;Acc:HGNC:26925]	protein coding	-0.46	5.32	34.94	<0.0001	<0.0001
ENSG00000014914	MTMR11	myotubularin related protein 11 [Source:HGNC Symbol;Acc:HGNC:24307]	protein coding	-0.46	2.48	15.42	<0.0001	0.0059
ENSG00000139354	GAS2L3	growth arrest specific 2 like 3 [Source:HGNC Symbol;Acc:HGNC:27475]	protein coding	-0.46	2.47	10.90	0.001	0.0311
ENSG00000183307	TMEM121B	transmembrane protein 121B [Source:HGNC Symbol;Acc:HGNC:1844]	protein coding	-0.46	2.03	10.46	0.0012	0.0363
ENSG00000145555	MYO10	myosin X [Source:HGNC Symbol;Acc:HGNC:7593]	protein coding	-0.46	3.64	20.20	<0.0001	0.0014
ENSG00000249173	LINC01093	long intergenic non-protein coding RNA 1093 [Source:HGNC Symbol;Acc:HGNC:49218]	lincRNA	-0.46	2.06	13.51	0.0002	0.0123
ENSG00000243440			protein coding	-0.45	2.34	10.38	0.0013	0.0368
ENSG00000073712	FERMT2	fermitin family member 2 [Source:HGNC Symbol;Acc:HGNC:15767]	protein coding	-0.45	4.60	34.27	<0.0001	<0.0001
ENSG00000153071	DAB2	DAB2, clathrin adaptor protein [Source:HGNC Symbol;Acc:HGNC:2662]	protein coding	-0.45	5.40	25.15	<0.0001	0.0004
ENSG00000162493	PDPN	podoplanin [Source:HGNC Symbol;Acc:HGNC:29602]	protein coding	-0.45	5.71	10.96	0.0009	0.0304
ENSG00000226979	LTA	lymphotoxin alpha [Source:HGNC Symbol;Acc:HGNC:6709]	protein coding	0.45	4.14	14.92	0.0001	0.0071
ENSG00000198682	PAPSS2	3'-phosphoadenosine 5'-phosphosulfate synthase 2 [Source:HGNC Symbol;Acc:HGNC:8604]	protein coding	-0.45	6.01	25.22	<0.0001	0.0004
ENSG00000142185	TRPM2	transient receptor potential cation channel subfamily M member 2 [Source:HGNC Symbol;Acc:HGNC:12339]	protein coding	-0.44	6.04	23.09	<0.0001	0.0006
ENSG00000123689	G0S2	G0/G1 switch 2 [Source:HGNC Symbol;Acc:HGNC:30229]	protein coding	-0.44	7.34	17.98	<0.0001	0.0024

Supplementary Table 10: Genes differentially expressed at day 14 post-second vaccination compared to day 0 (RNA-Seq). Sorted by descending absolute log_2 fold change (Day 14 post-second vaccination vs. Day 0). Gene model summaries and annotations are based on Ensembl Version 90 (August 2017) (continued).

**Ensembl Gene ID**	**Ensembl Gene Name**	**Ensembl Gene Description**	**Gene Type**	Log_2 **Fold Change (Day 14 vs. Day 0)**	**Average** Log_2 **CPM**	**Likelihood Ratio Test Statistic**	**P-Value**	**FDR Adjusted P-Value**
ENSG00000236939	BAALC-AS2	BAALC antisense RNA 2 [Source:HGNC Symbol;Acc:HGNC:28595]	antisense RNA	-0.44	1.89	11.76	0.0006	0.0233
ENSG00000124216	SNAI1	snail family transcriptional repressor 1 [Source:HGNC Symbol;Acc:HGNC:11128]	protein coding	-0.44	2.98	15.58	<0.0001	0.0058
ENSG00000225342			antisense RNA	0.44	1.18	10.54	0.0012	0.0353
ENSG00000106853	PTGR1	prostaglandin reductase 1 [Source:HGNC Symbol;Acc:HGNC:18429]	protein coding	-0.44	3.50	18.94	<0.0001	0.0018
ENSG00000254415	SIGLEC14	sialic acid binding Ig like lectin 14 [Source:HGNC Symbol;Acc:HGNC:32926]	protein coding	-0.44	6.45	31.57	<0.0001	<0.0001
ENSG00000143226	FCGR2A	Fc fragment of IgG receptor IIa [Source:HGNC Symbol;Acc:HGNC:3616]	protein coding	-0.44	7.06	17.08	<0.0001	0.0034
ENSG00000185052	SLC24A3	solute carrier family 24 member 3 [Source:HGNC Symbol;Acc:HGNC:10977]	protein coding	-0.43	3.49	11.03	0.0009	0.0298
ENSG00000250274			lincRNA	-0.43	2.66	13.20	0.0003	0.0139
ENSG00000008394	MGST1	microsomal glutathione S-transferase 1 [Source:HGNC Symbol;Acc:HGNC:7061]	protein coding	-0.43	3.76	18.86	<0.0001	0.0019
ENSG00000115594	IL1R1	interleukin 1 receptor type 1 [Source:HGNC Symbol;Acc:HGNC:5993]	protein coding	-0.43	6.20	23.82	<0.0001	0.0005
ENSG00000250929	LINC01181	long intergenic non-protein coding RNA 1181 [Source:HGNC Symbol;Acc:HGNC:25533]	antisense RNA	-0.43	1.90	13.63	0.0002	0.012
ENSG00000115107	STEAP3	STEAP3 metalloreductase [Source:HGNC Symbol;Acc:HGNC:24592]	protein coding	-0.43	4.94	16.87	<0.0001	0.0036
ENSG00000072840	EVC	EvC ciliary complex subunit 1 [Source:HGNC Symbol;Acc:HGNC:3497]	protein coding	-0.43	2.42	10.90	0.001	0.0311
ENSG00000035862	TIMP2	TIMP metallopeptidase inhibitor 2 [Source:HGNC Symbol;Acc:HGNC:11821]	protein coding	-0.43	4.65	23.75	<0.0001	0.0005
ENSG00000002587	HS3ST1	heparan sulfate-glucosamine 3-sulfotransferase 1 [Source:HGNC Symbol;Acc:HGNC:5194]	protein coding	-0.43	5.04	11.69	0.0006	0.024
ENSG00000166396	SERPINB7	serpin family B member 7 [Source:HGNC Symbol;Acc:HGNC:13902]	protein coding	-0.43	7.25	13.98	0.0002	0.0106
ENSG00000171631	P2RY6	pyrimidinergic receptor P2Y6 [Source:HGNC Symbol;Acc:HGNC:8543]	protein coding	-0.42	4.59	12.37	0.0004	0.0189
ENSG00000111424	VDR	vitamin D receptor [Source:HGNC Symbol;Acc:HGNC:12679]	protein coding	-0.42	5.88	16.73	<0.0001	0.0038

Supplementary Table 10: Genes differentially expressed at day 14 post-second vaccination compared to day 0 (RNA-Seq). Sorted by descending absolute log_2 fold change (Day 14 post-second vaccination vs. Day 0). Gene model summaries and annotations are based on Ensembl Version 90 (August 2017) (continued).

Ensembl Gene ID	Ensembl Gene Name	Ensembl Gene Description	Gene Type	Log_2 Fold Change (Day 14 vs. Day 0)	Average Log_2 CPM	Likelihood Ratio Test Statistic	P-Value	FDR Adjusted P-Value
ENSG00000110079	MS4A4A	membrane spanning 4-domains A4A [Source:HGNC Symbol;Acc:HGNC:13371]	protein coding	-0.42	2.89	12.00	0.0005	0.0215
ENSG00000183762	KREMEN1	kringle containing transmembrane protein 1 [Source:HGNC Symbol;Acc:HGNC:17550]	protein coding	-0.42	6.55	13.02	0.0003	0.015
ENSG00000103569	AQP9	aquaporin 9 [Source:HGNC Symbol;Acc:HGNC:643]	protein coding	-0.42	9.57	16.87	<0.0001	0.0036
ENSG00000125810	CD93	CD93 molecule [Source:HGNC Symbol;Acc:HGNC:15855]	protein coding	-0.42	9.73	19.86	<0.0001	0.0015
ENSG00000051128	HOMER3	homer scaffolding protein 3 [Source:HGNC Symbol;Acc:HGNC:17514]	protein coding	-0.42	3.06	18.31	<0.0001	0.0022
ENSG00000103888	CEMIP	cell migration inducing hyaluronan binding protein [Source:HGNC Symbol;Acc:HGNC:29213]	protein coding	-0.42	9.85	9.66	0.0019	0.0477
ENSG00000100767	PAPLN	papilin, proteoglycan like sulfated glycoprotein [Source:HGNC Symbol;Acc:HGNC:19262]	protein coding	-0.42	4.63	16.32	<0.0001	0.0045
ENSG00000100292	HMOX1	heme oxygenase 1 [Source:HGNC Symbol;Acc:HGNC:5013]	protein coding	-0.42	7.24	18.74	<0.0001	0.0019
ENSG00000167850	CD300C	CD300c molecule [Source:HGNC Symbol;Acc:HGNC:19320]	protein coding	-0.41	3.90	16.40	<0.0001	0.0044
ENSG00000172594	SMPDL3A	sphingomyelin phosphodiesterase acid like 3A [Source:HGNC Symbol;Acc:HGNC:17389]	protein coding	-0.41	6.79	14.34	0.0002	0.0091
ENSG00000116962	NID1	nidogen 1 [Source:HGNC Symbol;Acc:HGNC:7821]	protein coding	-0.41	4.30	23.32	<0.0001	0.0006
ENSG00000101188	NTSR1	neurotensin receptor 1 [Source:HGNC Symbol;Acc:HGNC:8039]	protein coding	-0.41	1.79	10.10	0.0015	0.0406
ENSG00000169860	P2RY1	purinergic receptor P2Y1 [Source:HGNC Symbol;Acc:HGNC:8539]	protein coding	-0.41	2.58	18.09	<0.0001	0.0024
ENSG00000165480	SKA3	spindle and kinetochore associated complex subunit 3 [Source:HGNC Symbol;Acc:HGNC:20262]	protein coding	-0.41	1.67	10.75	0.001	0.0327
ENSG00000224397	SMIM25	small integral membrane protein 25 [Source:HGNC Symbol;Acc:HGNC:50328]	lincRNA	-0.41	4.47	15.61	<0.0001	0.0058
ENSG00000170458	CD14	CD14 molecule [Source:HGNC Symbol;Acc:HGNC:1628]	protein coding	-0.41	9.90	10.34	0.0013	0.0375
ENSG00000137331	IER3	immediate early response 3 [Source:HGNC Symbol;Acc:HGNC:5392]	protein coding	-0.41	10.06	15.45	<0.0001	0.0059

Supplementary Table 10: Genes differentially expressed at day 14 post-second vaccination compared to day 0 (RNA-Seq). Sorted by descending absolute log_2 fold change (Day 14 post-second vaccination vs. Day 0). Gene model summaries and annotations are based on Ensembl Version 90 (August 2017) (continued).

Ensembl Gene ID	Ensembl Gene Name	Ensembl Gene Description	Gene Type	Log_2 Fold Change (Day 14 vs. Day 0)	Average Log_2 CPM	Likelihood Ratio Test Statistic	P-Value	FDR Adjusted P-Value
ENSG00000189060	H1F0	H1 histone family member 0 [Source:HGNC Symbol;Acc:HGNC:4714]	protein coding	-0.41	5.32	20.46	<0.0001	0.0013
ENSG00000100504	PYGL	glycogen phosphorylase L [Source:HGNC Symbol;Acc:HGNC:9725]	protein coding	-0.41	5.47	10.58	0.0011	0.0348
ENSG00000178726	THBD	thrombomodulin [Source:HGNC Symbol;Acc:HGNC:11784]	protein coding	-0.40	5.39	16.98	<0.0001	0.0034
ENSG00000204161	C10orf128	chromosome 10 open reading frame 128 [Source:HGNC Symbol;Acc:HGNC:27274]	protein coding	0.40	3.38	13.56	0.0002	0.0121
ENSG00000167680	SEMA6B	semaphorin 6B [Source:HGNC Symbol;Acc:HGNC:10739]	protein coding	-0.40	7.93	20.27	<0.0001	0.0014
ENSG00000124104	SNX21	sorting nexin family member 21 [Source:HGNC Symbol;Acc:HGNC:16154]	protein coding	-0.40	3.20	25.01	<0.0001	0.0004
ENSG00000129450	SIGLEC9	sialic acid binding Ig like lectin 9 [Source:HGNC Symbol;Acc:HGNC:10878]	protein coding	-0.40	6.22	21.00	<0.0001	0.0011
ENSG00000196878	LAMB3	laminin subunit beta 3 [Source:HGNC Symbol;Acc:HGNC:6490]	protein coding	-0.40	5.51	18.82	<0.0001	0.0019
ENSG00000150510	FAM124A	family with sequence similarity 124 member A [Source:HGNC Symbol;Acc:HGNC:26413]	protein coding	-0.40	4.45	18.49	<0.0001	0.002
ENSG00000105976	MET	MET proto-oncogene, receptor tyrosine kinase [Source:HGNC Symbol;Acc:HGNC:7029]	protein coding	-0.40	8.30	15.16	<0.0001	0.0064
ENSG00000124731	TREM1	triggering receptor expressed on myeloid cells 1 [Source:HGNC Symbol;Acc:HGNC:17760]	protein coding	-0.40	8.02	20.22	<0.0001	0.0014
ENSG00000258376			antisense RNA	-0.40	2.71	12.61	0.0004	0.0176
ENSG00000175352	NRIP3	nuclear receptor interacting protein 3 [Source:HGNC Symbol;Acc:HGNC:1167]	protein coding	-0.39	5.62	22.89	<0.0001	0.0007
ENSG00000095383	TBC1D2	TBC1 domain family member 2 [Source:HGNC Symbol;Acc:HGNC:18026]	protein coding	-0.39	5.65	37.58	<0.0001	<0.0001
ENSG00000204822	MRPL53	mitochondrial ribosomal protein L53 [Source:HGNC Symbol;Acc:HGNC:16684]	protein coding	0.39	1.61	10.41	0.0013	0.0368
ENSG00000181790	ADGRB1	adhesion G protein-coupled receptor B1 [Source:HGNC Symbol;Acc:HGNC:943]	protein coding	-0.39	2.64	10.06	0.0015	0.0411
ENSG00000171476	HOPX	HOP homeobox [Source:HGNC Symbol;Acc:HGNC:24961]	protein coding	0.39	4.15	10.40	0.0013	0.0368

Supplementary Table 10: Genes differentially expressed at day 14 post-second vaccination compared to day 0 (RNA-Seq). Sorted by descending absolute log_2 fold change (Day 14 post-second vaccination vs. Day 0). Gene model summaries and annotations are based on Ensembl Version 90 (August 2017) (continued).

Ensembl Gene ID	Ensembl Gene Name	Ensembl Gene Description	Gene Type	Log_2 Fold Change (Day 14 vs. Day 0)	Average Log_2 CPM	Likelihood Ratio Test Statistic	P-Value	FDR Adjusted P-Value
ENSG00000198853	RUSC2	RUN and SH3 domain containing 2 [Source:HGNC Symbol;Acc:HGNC:23625]	protein coding	-0.39	5.90	25.75	<0.0001	0.0004
ENSG00000165029	ABCA1	ATP binding cassette subfamily A member 1 [Source:HGNC Symbol;Acc:HGNC:29]	protein coding	-0.38	9.57	11.62	0.0007	0.0244
ENSG00000273443			lincRNA	-0.38	1.27	9.98	0.0016	0.0427
ENSG00000121743	GJA3	gap junction protein alpha 3 [Source:HGNC Symbol;Acc:HGNC:4277]	protein coding	-0.38	5.35	22.77	<0.0001	0.0007
ENSG00000158869	FCER1G	Fc fragment of IgE receptor Ig [Source:HGNC Symbol;Acc:HGNC:3611]	protein coding	-0.38	9.35	12.33	0.0004	0.0191
ENSG00000050730	TNIP3	TNFAIP3 interacting protein 3 [Source:HGNC Symbol;Acc:HGNC:19315]	protein coding	-0.38	8.39	18.97	<0.0001	0.0018
ENSG00000153558	FBXL2	F-box and leucine rich repeat protein 2 [Source:HGNC Symbol;Acc:HGNC:13598]	protein coding	0.38	1.32	10.66	0.0011	0.0337
ENSG00000267737			lincRNA	-0.38	2.39	10.94	0.0009	0.0307
ENSG00000123342	MMP19	matrix metallopeptidase 19 [Source:HGNC Symbol;Acc:HGNC:7165]	protein coding	-0.38	7.41	25.24	<0.0001	0.0004
ENSG00000180113	TDRD6	tudor domain containing 6 [Source:HGNC Symbol;Acc:HGNC:21339]	protein coding	-0.38	5.28	16.16	<0.0001	0.0048
ENSG00000104974	LILRA1	leukocyte immunoglobulin like receptor A1 [Source:HGNC Symbol;Acc:HGNC:6602]	protein coding	-0.38	6.43	12.80	0.0003	0.0164
ENSG00000182010	RTKN2	rhotekin 2 [Source:HGNC Symbol;Acc:HGNC:19364]	protein coding	0.38	3.35	17.83	<0.0001	0.0025
ENSG00000116574	RHOU	ras homolog family member U [Source:HGNC Symbol;Acc:HGNC:17794]	protein coding	-0.38	6.80	31.18	<0.0001	<0.0001
ENSG00000171517	LPAR3	lysophosphatidic acid receptor 3 [Source:HGNC Symbol;Acc:HGNC:14298]	protein coding	-0.38	3.94	13.88	0.0002	0.0109
ENSG00000139926	FRMD6	FERM domain containing 6 [Source:HGNC Symbol;Acc:HGNC:19839]	protein coding	-0.37	2.35	11.45	0.0007	0.0256
ENSG00000146674	IGFBP3	insulin like growth factor binding protein 3 [Source:HGNC Symbol;Acc:HGNC:5472]	protein coding	0.37	2.88	10.01	0.0016	0.042
ENSG00000213977	TAX1BP3	Tax1 binding protein 3 [Source:HGNC Symbol;Acc:HGNC:30684]	protein coding	-0.37	1.89	10.24	0.0014	0.0387
ENSG00000184371	CSF1	colony stimulating factor 1 [Source:HGNC Symbol;Acc:HGNC:2432]	protein coding	0.37	4.46	17.92	<0.0001	0.0025

Supplementary Table 10: Genes differentially expressed at day 14 post-second vaccination compared to day 0 (RNA-Seq). Sorted by descending absolute log_2 fold change (Day 14 post-second vaccination vs. Day 0). Gene model summaries and annotations are based on Ensembl Version 90 (August 2017) (continued).

**Ensembl Gene ID**	**Ensembl Gene Name**	**Ensembl Gene Description**	**Gene Type**	**Log_2 Fold Change (Day 14 vs. Day 0)**	**Average Log_2 CPM**	**Likelihood Ratio Test Statistic**	**P-Value**	**FDR Adjusted P-Value**
ENSG00000152213	ARL11	ADP ribosylation factor like GTPase 11 [Source:HGNC Symbol;Acc:HGNC:24046]	protein coding	-0.37	2.36	15.74	<0.0001	0.0056
ENSG00000117090	SLAMF1	signaling lymphocytic activation molecule family member 1 [Source:HGNC Symbol;Acc:HGNC:10903]	protein coding	-0.37	8.05	22.87	<0.0001	0.0007
ENSG00000198719	DLL1	delta like canonical Notch ligand 1 [Source:HGNC Symbol;Acc:HGNC:2908]	protein coding	-0.37	5.04	24.94	<0.0001	0.0004
ENSG00000128274	A4GALT	alpha 1,4-galactosyltransferase (P blood group) [Source:HGNC Symbol;Acc:HGNC:18149]	protein coding	-0.37	3.49	9.70	0.0018	0.0471
ENSG00000075391	RASAL2	RAS protein activator like 2 [Source:HGNC Symbol;Acc:HGNC:9874]	protein coding	-0.37	3.22	11.87	0.0006	0.0224
ENSG00000107249	GLIS3	GLIS family zinc finger 3 [Source:HGNC Symbol;Acc:HGNC:28510]	protein coding	-0.37	5.55	11.79	0.0006	0.023
ENSG00000090674	MCOLN1	mucolipin 1 [Source:HGNC Symbol;Acc:HGNC:13356]	protein coding	-0.36	3.52	26.34	<0.0001	0.0003
ENSG00000070731	ST6GALNAC2	ST6 N-acetylgalactosaminide alpha-2,6-sialyltransferase 2 [Source:HGNC Symbol;Acc:HGNC:10867]	protein coding	-0.36	2.33	16.74	<0.0001	0.0038
ENSG00000138061	CYP1B1	cytochrome P450 family 1 subfamily B member 1 [Source:HGNC Symbol;Acc:HGNC:2597]	protein coding	-0.36	8.63	13.06	0.0003	0.0148
ENSG00000020181	ADGRA2	adhesion G protein-coupled receptor A2 [Source:HGNC Symbol;Acc:HGNC:17849]	protein coding	-0.36	5.06	19.87	<0.0001	0.0015
ENSG00000197249	SERPINA1	serpin family A member 1 [Source:HGNC Symbol;Acc:HGNC:8941]	protein coding	-0.36	9.36	11.84	0.0006	0.0227
ENSG00000120162	MOB3B	MOB kinase activator 3B [Source:HGNC Symbol;Acc:HGNC:23825]	protein coding	-0.36	3.16	20.08	<0.0001	0.0014
ENSG00000167613	LAIR1	leukocyte associated immunoglobulin like receptor 1 [Source:HGNC Symbol;Acc:HGNC:6477]	protein coding	-0.36	8.19	15.86	<0.0001	0.0054
ENSG00000114315	HES1	hes family bHLH transcription factor 1 [Source:HGNC Symbol;Acc:HGNC:5192]	protein coding	-0.36	3.97	15.54	<0.0001	0.0058
ENSG00000125730	C3	complement C3 [Source:HGNC Symbol;Acc:HGNC:1318]	protein coding	-0.36	6.94	20.75	<0.0001	0.0012
ENSG00000198053	SIRPA	signal regulatory protein alpha [Source:HGNC Symbol;Acc:HGNC:9662]	protein coding	-0.36	7.27	19.82	<0.0001	0.0015

Supplementary Table 10: Genes differentially expressed at day 14 post-second vaccination compared to day 0 (RNA-Seq). Sorted by descending absolute log_2 fold change (Day 14 post-second vaccination vs. Day 0). Gene model summaries and annotations are based on Ensembl Version 90 (August 2017) (continued).

Ensembl Gene ID	Ensembl Gene Name	Ensembl Gene Description	Gene Type	Log_2 Fold Change (Day 14 vs. Day 0)	Average Log_2 CPM	Likelihood Ratio Test Statistic	P-Value	FDR Adjusted P-Value
ENSG00000105509	HAS1	hyaluronan synthase 1 [Source:HGNC Symbol;Acc:HGNC:4818]	protein coding	-0.36	7.00	15.19	<0.0001	0.0064
ENSG00000075651	PLD1	phospholipase D1 [Source:HGNC Symbol;Acc:HGNC:9067]	protein coding	-0.36	6.67	17.10	<0.0001	0.0034
ENSG00000168329	CX3CR1	C-X3-C motif chemokine receptor 1 [Source:HGNC Symbol;Acc:HGNC:2558]	protein coding	0.36	2.35	10.52	0.0012	0.0356
ENSG00000127838	PNKD	paroxysmal nonkinesigenic dyskinesia [Source:HGNC Symbol;Acc:HGNC:9153]	protein coding	-0.36	6.63	18.73	<0.0001	0.0019
ENSG00000175489	LRRC25	leucine rich repeat containing 25 [Source:HGNC Symbol;Acc:HGNC:29806]	protein coding	-0.36	8.53	13.88	0.0002	0.0109
ENSG00000143067	ZNF697	zinc finger protein 697 [Source:HGNC Symbol;Acc:HGNC:32034]	protein coding	-0.36	4.88	15.72	<0.0001	0.0056
ENSG00000128218	VPREB3	V-set pre-B cell surrogate light chain 3 [Source:HGNC Symbol;Acc:HGNC:12710]	protein coding	0.36	2.39	9.87	0.0017	0.0441
ENSG00000148926	ADM	adrenomedullin [Source:HGNC Symbol;Acc:HGNC:259]	protein coding	-0.36	7.47	16.98	<0.0001	0.0034
ENSG00000181019	NQO1	NAD(P)H quinone dehydrogenase 1 [Source:HGNC Symbol;Acc:HGNC:2874]	protein coding	-0.36	4.34	16.64	<0.0001	0.0039
ENSG00000073792	IGF2BP2	insulin like growth factor 2 mRNA binding protein 2 [Source:HGNC Symbol;Acc:HGNC:28867]	protein coding	-0.35	2.40	12.49	0.0004	0.0183
ENSG00000135636	DYSF	dysferlin [Source:HGNC Symbol;Acc:HGNC:3097]	protein coding	-0.35	4.83	11.11	0.0009	0.0293
ENSG00000103855	CD276	CD276 molecule [Source:HGNC Symbol;Acc:HGNC:19137]	protein coding	-0.35	5.14	14.48	0.0001	0.0086
ENSG00000239998	LILRA2	leukocyte immunoglobulin like receptor A2 [Source:HGNC Symbol;Acc:HGNC:6603]	protein coding	-0.35	6.61	12.57	0.0004	0.0178
ENSG00000109674	NEIL3	nei like DNA glycosylase 3 [Source:HGNC Symbol;Acc:HGNC:24573]	protein coding	-0.35	3.42	11.43	0.0007	0.0258
ENSG00000127507	ADGRE2	adhesion G protein-coupled receptor E2 [Source:HGNC Symbol;Acc:HGNC:3337]	protein coding	-0.35	7.13	12.95	0.0003	0.0154
ENSG00000122861	PLAU	plasminogen activator, urokinase [Source:HGNC Symbol;Acc:HGNC:9052]	protein coding	-0.35	5.46	19.13	<0.0001	0.0017
ENSG00000165886	UBTD1	ubiquitin domain containing 1 [Source:HGNC Symbol;Acc:HGNC:25683]	protein coding	-0.35	3.87	15.14	<0.0001	0.0065

Supplementary Table 10: Genes differentially expressed at day 14 post-second vaccination compared to day 0 (RNA-Seq). Sorted by descending absolute log_2 fold change (Day 14 post-second vaccination vs. Day 0). Gene model summaries and annotations are based on Ensembl Version 90 (August 2017) (continued).

**Ensembl Gene ID**	**Ensembl Gene Name**	**Ensembl Gene Description**	**Gene Type**	Log_2 **Fold Change (Day 14 vs. Day 0)**	**Average** Log_2 **CPM**	**Likelihood Ratio Test Statistic**	**P-Value**	**FDR Adjusted P-Value**
ENSG00000132669	RIN2	Ras and Rab interactor 2 [Source:HGNC Symbol;Acc:HGNC:18750]	protein coding	-0.35	8.08	18.68	<0.0001	0.0019
ENSG00000168995	SIGLEC7	sialic acid binding Ig like lectin 7 [Source:HGNC Symbol;Acc:HGNC:10876]	protein coding	-0.35	3.68	11.65	0.0006	0.0241
ENSG00000143162	CREG1	cellular repressor of E1A stimulated genes 1 [Source:HGNC Symbol;Acc:HGNC:2351]	protein coding	-0.35	8.43	18.09	<0.0001	0.0024
ENSG00000243649	CFB	complement factor B [Source:HGNC Symbol;Acc:HGNC:1037]	protein coding	-0.35	4.13	12.37	0.0004	0.0189
ENSG00000186818	LILRB4	leukocyte immunoglobulin like receptor B4 [Source:HGNC Symbol;Acc:HGNC:6608]	protein coding	-0.35	8.40	12.25	0.0005	0.0196
ENSG00000163235	TGFA	transforming growth factor alpha [Source:HGNC Symbol;Acc:HGNC:11765]	protein coding	-0.35	6.06	22.37	<0.0001	0.0008
ENSG00000079308	TNS1	tensin 1 [Source:HGNC Symbol;Acc:HGNC:11973]	protein coding	-0.35	4.54	9.92	0.0016	0.0434
ENSG00000153823	PID1	phosphotyrosine interaction domain containing 1 [Source:HGNC Symbol;Acc:HGNC:26084]	protein coding	-0.35	7.21	10.80	0.001	0.0321
ENSG00000143545	RAB13	RAB13, member RAS oncogene family [Source:HGNC Symbol;Acc:HGNC:9762]	protein coding	-0.35	5.22	11.03	0.0009	0.0298
ENSG00000168461	RAB31	RAB31, member RAS oncogene family [Source:HGNC Symbol;Acc:HGNC:9771]	protein coding	-0.35	7.81	18.82	<0.0001	0.0019
ENSG00000138448	ITGAV	integrin subunit alpha V [Source:HGNC Symbol;Acc:HGNC:6150]	protein coding	-0.34	8.06	13.56	0.0002	0.0121
ENSG00000197405	C5AR1	complement C5a receptor 1 [Source:HGNC Symbol;Acc:HGNC:1338]	protein coding	-0.34	8.36	13.61	0.0002	0.012
ENSG00000104043	ATP8B4	ATPase phospholipid transporting 8B4 (putative) [Source:HGNC Symbol;Acc:HGNC:13536]	protein coding	-0.34	4.05	16.98	<0.0001	0.0034
ENSG00000170909	OSCAR	osteoclast associated, immunoglobulin-like receptor [Source:HGNC Symbol;Acc:HGNC:29960]	protein coding	-0.34	5.82	20.19	<0.0001	0.0014
ENSG00000059728	MXD1	MAX dimerization protein 1 [Source:HGNC Symbol;Acc:HGNC:6761]	protein coding	-0.34	8.54	14.76	0.0001	0.0076
ENSG00000167703	SLC43A2	solute carrier family 43 member 2 [Source:HGNC Symbol;Acc:HGNC:23087]	protein coding	-0.34	9.78	9.96	0.0016	0.0429
ENSG00000233058	LINC00884	long intergenic non-protein coding RNA 884 [Source:HGNC Symbol;Acc:HGNC:48570]	antisense RNA	-0.34	3.56	15.62	<0.0001	0.0058

Supplementary Table 10: Genes differentially expressed at day 14 post-second vaccination compared to day 0 (RNA-Seq). Sorted by descending absolute log_2 fold change (Day 14 post-second vaccination vs. Day 0). Gene model summaries and annotations are based on Ensembl Version 90 (August 2017) (continued).

Ensembl Gene ID	Ensembl Gene Name	Ensembl Gene Description	Gene Type	Log_2 Fold Change (Day 14 vs. Day 0)	Average Log_2 CPM	Likelihood Ratio Test Statistic	P-Value	FDR Adjusted P-Value
ENSG00000147650	LRP12	LDL receptor related protein 12 [Source:HGNC Symbol;Acc:HGNC:31708]	protein coding	-0.34	4.82	19.22	<0.0001	0.0017
ENSG00000268734			lincRNA	-0.34	4.71	11.04	0.0009	0.0298
ENSG00000111186	WNT5B	Wnt family member 5B [Source:HGNC Symbol;Acc:HGNC:16265]	protein coding	-0.34	2.47	10.96	0.0009	0.0304
ENSG00000105928	DFNA5	DFNA5, deafness associated tumor suppressor [Source:HGNC Symbol;Acc:HGNC:2810]	protein coding	-0.34	5.94	20.00	<0.0001	0.0015
ENSG00000204577	LILRB3	leukocyte immunoglobulin like receptor B3 [Source:HGNC Symbol;Acc:HGNC:6607]	protein coding	-0.34	6.28	13.25	0.0003	0.0137
ENSG00000215861			unprocessed pseudogene	-0.34	4.14	11.64	0.0006	0.0242
ENSG00000256235	SMIM3	small integral membrane protein 3 [Source:HGNC Symbol;Acc:HGNC:30248]	protein coding	-0.34	6.49	24.65	<0.0001	0.0004
ENSG00000261618			lincRNA	-0.34	4.18	11.01	0.0009	0.03
ENSG00000146070	PLA2G7	phospholipase A2 group VII [Source:HGNC Symbol;Acc:HGNC:9040]	protein coding	-0.34	8.24	14.50	0.0001	0.0086
ENSG00000121316	PLBD1	phospholipase B domain containing 1 [Source:HGNC Symbol;Acc:HGNC:26215]	protein coding	-0.34	2.42	10.17	0.0014	0.0395
ENSG00000173918	C1QTNF1	C1q and TNF related 1 [Source:HGNC Symbol;Acc:HGNC:14324]	protein coding	-0.33	7.83	14.61	0.0001	0.0081
ENSG00000074964	ARHGEF10L	Rho guanine nucleotide exchange factor 10 like [Source:HGNC Symbol;Acc:HGNC:25540]	protein coding	-0.33	7.34	17.11	<0.0001	0.0034
ENSG00000052795	FNIP2	folliculin interacting protein 2 [Source:HGNC Symbol;Acc:HGNC:29280]	protein coding	-0.33	7.80	20.24	<0.0001	0.0014
ENSG00000105520	PLPPR2	phospholipid phosphatase related 2 [Source:HGNC Symbol;Acc:HGNC:29566]	protein coding	-0.33	3.32	10.17	0.0014	0.0395
ENSG00000169439	SDC2	syndecan 2 [Source:HGNC Symbol;Acc:HGNC:10659]	protein coding	-0.33	8.42	13.50	0.0002	0.0123
ENSG00000120129	DUSP1	dual specificity phosphatase 1 [Source:HGNC Symbol;Acc:HGNC:3064]	protein coding	-0.33	9.86	12.03	0.0005	0.0214
ENSG00000196923	PDLIM7	PDZ and LIM domain 7 [Source:HGNC Symbol;Acc:HGNC:22958]	protein coding	-0.33	4.84	28.68	<0.0001	0.0001
ENSG00000088826	SMOX	spermine oxidase [Source:HGNC Symbol;Acc:HGNC:15862]	protein coding	-0.33	5.29	16.31	<0.0001	0.0045

Supplementary Table 10: Genes differentially expressed at day 14 post-second vaccination compared to day 0 (RNA-Seq). Sorted by descending absolute log_2 fold change (Day 14 post-second vaccination vs. Day 0). Gene model summaries and annotations are based on Ensembl Version 90 (August 2017) (continued).

**Ensembl Gene ID**	**Ensembl Gene Name**	**Ensembl Gene Description**	**Gene Type**	Log_2 **Fold Change (Day 14 vs. Day 0)**	**Average** Log_2 **CPM**	**Likelihood Ratio Test Statistic**	**P-Value**	**FDR Adjusted P-Value**
ENSG00000140022	STON2	stonin 2 [Source:HGNC Symbol;Acc:HGNC:30652]	protein coding	-0.33	3.42	10.18	0.0014	0.0395
ENSG00000146094	DOK3	docking protein 3 [Source:HGNC Symbol;Acc:HGNC:24583]	protein coding	-0.33	5.96	21.97	<0.0001	0.0008
ENSG00000123610	TNFAIP6	TNF alpha induced protein 6 [Source:HGNC Symbol;Acc:HGNC:11898]	protein coding	-0.33	8.71	11.90	0.0006	0.0223
ENSG00000164929	BAALC	BAALC, MAP3K1 and KLF4 binding [Source:HGNC Symbol;Acc:HGNC:14333]	protein coding	-0.33	3.56	11.01	0.0009	0.03
ENSG00000108950	FAM20A	FAM20A, golgi associated secretory pathway pseudokinase [Source:HGNC Symbol;Acc:HGNC:23015]	protein coding	-0.33	5.77	10.82	0.001	0.0319
ENSG00000136098	NEK3	NIMA related kinase 3 [Source:HGNC Symbol;Acc:HGNC:7746]	protein coding	-0.33	2.69	10.29	0.0013	0.038
ENSG00000091073	DTX2	deltex E3 ubiquitin ligase 2 [Source:HGNC Symbol;Acc:HGNC:15973]	protein coding	-0.33	4.79	18.97	<0.0001	0.0018
ENSG00000166825	ANPEP	alanyl aminopeptidase, membrane [Source:HGNC Symbol;Acc:HGNC:500]	protein coding	-0.33	8.86	14.36	0.0002	0.0091
ENSG00000176170	SPHK1	sphingosine kinase 1 [Source:HGNC Symbol;Acc:HGNC:11240]	protein coding	-0.33	6.93	14.27	0.0002	0.0093
ENSG00000186431	FCAR	Fc fragment of IgA receptor [Source:HGNC Symbol;Acc:HGNC:3608]	protein coding	-0.32	6.63	15.53	<0.0001	0.0058
ENSG00000139572	GPR84	G protein-coupled receptor 84 [Source:HGNC Symbol;Acc:HGNC:4535]	protein coding	-0.32	6.85	11.90	0.0006	0.0223
ENSG00000189233	NUGGC	nuclear GTPase, germinal center associated [Source:HGNC Symbol;Acc:HGNC:33550]	protein coding	0.32	2.36	11.12	0.0009	0.0293
ENSG00000099250	NRP1	neuropilin 1 [Source:HGNC Symbol;Acc:HGNC:8004]	protein coding	-0.32	6.61	15.24	<0.0001	0.0063
ENSG00000130202	NECTIN2	nectin cell adhesion molecule 2 [Source:HGNC Symbol;Acc:HGNC:9707]	protein coding	-0.32	7.56	9.90	0.0017	0.0438
ENSG00000163069	SGCB	sarcoglycan beta [Source:HGNC Symbol;Acc:HGNC:10806]	protein coding	0.32	2.30	11.45	0.0007	0.0256
ENSG00000131042	LILRB2	leukocyte immunoglobulin like receptor B2 [Source:HGNC Symbol;Acc:HGNC:6606]	protein coding	-0.32	8.33	9.87	0.0017	0.0441
ENSG00000244482	LILRA6	leukocyte immunoglobulin like receptor A6 [Source:HGNC Symbol;Acc:HGNC:15495]	protein coding	-0.32	5.57	15.54	<0.0001	0.0058

Supplementary Table 10: Genes differentially expressed at day 14 post-second vaccination compared to day 0 (RNA-Seq). Sorted by descending absolute log_2 fold change (Day 14 post-second vaccination vs. Day 0). Gene model summaries and annotations are based on Ensembl Version 90 (August 2017) (continued).

Ensembl Gene ID	Ensembl Gene Name	Ensembl Gene Description	Gene Type	Log_2 Fold Change (Day 14 vs. Day 0)	Average Log_2 CPM	Likelihood Ratio Test Statistic	P-Value	FDR Adjusted P-Value
ENSG00000119900	OGFRL1	opioid growth factor receptor like 1 [Source:HGNC Symbol;Acc:HGNC:21378]	protein coding	-0.32	7.58	18.46	<0.0001	0.0021
ENSG00000101187	SLCO4A1	solute carrier organic anion transporter family member 4A1 [Source:HGNC Symbol;Acc:HGNC:10953]	protein coding	-0.32	7.48	24.35	<0.0001	0.0005
ENSG00000177706	FAM20C	FAM20C, golgi associated secretory pathway kinase [Source:HGNC Symbol;Acc:HGNC:22140]	protein coding	-0.32	3.96	12.70	0.0004	0.0172
ENSG00000135678	CPM	carboxypeptidase M [Source:HGNC Symbol;Acc:HGNC:2311]	protein coding	-0.32	5.66	13.91	0.0002	0.0109
ENSG00000132205	EMILIN2	elastin microfibril interfacer 2 [Source:HGNC Symbol;Acc:HGNC:19881]	protein coding	-0.32	8.81	13.63	0.0002	0.012
ENSG00000111321	LTBR	lymphotoxin beta receptor [Source:HGNC Symbol;Acc:HGNC:6718]	protein coding	-0.32	5.98	19.17	<0.0001	0.0017
ENSG00000229644	NAMPTP1	nicotinamide phosphoribosyltransferase pseudogene 1 [Source:HGNC Symbol;Acc:HGNC:17633]	processed pseudogene	-0.31	8.43	11.46	0.0007	0.0256
ENSG00000123411	IKZF4	IKAROS family zinc finger 4 [Source:HGNC Symbol;Acc:HGNC:13179]	protein coding	0.31	2.22	11.11	0.0009	0.0293
ENSG00000242048			processed transcript	-0.31	3.80	10.22	0.0014	0.039
ENSG00000087074	PPP1R15A	protein phosphatase 1 regulatory subunit 15A [Source:HGNC Symbol;Acc:HGNC:14375]	protein coding	-0.31	8.71	18.04	<0.0001	0.0024
ENSG00000135404	CD63	CD63 molecule [Source:HGNC Symbol;Acc:HGNC:1692]	protein coding	-0.31	9.16	10.46	0.0012	0.0363
ENSG00000114251	WNT5A	Wnt family member 5A [Source:HGNC Symbol;Acc:HGNC:12784]	protein coding	-0.31	5.57	12.57	0.0004	0.0178
ENSG00000157557	ETS2	ETS proto-oncogene 2, transcription factor [Source:HGNC Symbol;Acc:HGNC:3489]	protein coding	-0.31	8.46	19.03	<0.0001	0.0018
ENSG00000118971	CCND2	cyclin D2 [Source:HGNC Symbol;Acc:HGNC:1583]	protein coding	0.31	8.37	15.77	<0.0001	0.0056
ENSG00000131981	LGALS3	galectin 3 [Source:HGNC Symbol;Acc:HGNC:6563]	protein coding	-0.31	7.94	17.25	<0.0001	0.0032
ENSG00000140678	ITGAX	integrin subunit alpha X [Source:HGNC Symbol;Acc:HGNC:6152]	protein coding	-0.31	7.02	23.46	<0.0001	0.0006
ENSG00000085514	PILRA	paired immunoglobin like type 2 receptor alpha [Source:HGNC Symbol;Acc:HGNC:20396]	protein coding	-0.31	8.08	15.62	<0.0001	0.0058
ENSG00000136720	HS6ST1	heparan sulfate 6-O-sulfotransferase 1 [Source:HGNC Symbol;Acc:HGNC:5201]	protein coding	-0.31	5.80	19.51	<0.0001	0.0016

Supplementary Table 10: Genes differentially expressed at day 14 post-second vaccination compared to day 0 (RNA-Seq). Sorted by descending absolute log_2 fold change (Day 14 post-second vaccination vs. Day 0). Gene model summaries and annotations are based on Ensembl Version 90 (August 2017) (continued).

**Ensembl Gene ID**	**Ensembl Gene Name**	**Ensembl Gene Description**	**Gene Type**	Log_2 **Fold Change (Day 14 vs. Day 0)**	**Average** Log_2 **CPM**	**Likelihood Ratio Test Statistic**	**P-Value**	**FDR Adjusted P-Value**
ENSG00000160746	ANO10	anoctamin 10 [Source:HGNC Symbol;Acc:HGNC:25519]	protein coding	-0.31	4.27	18.78	<0.0001	0.0019
ENSG00000113070	HBEGF	heparin binding EGF like growth factor [Source:HGNC Symbol;Acc:HGNC:3059]	protein coding	-0.31	6.70	13.21	0.0003	0.0139
ENSG00000140859	KIFC3	kinesin family member C3 [Source:HGNC Symbol;Acc:HGNC:6326]	protein coding	-0.31	6.35	10.70	0.0011	0.0332
ENSG00000198018	ENTPD7	ectonucleoside triphosphate diphosphohydrolase 7 [Source:HGNC Symbol;Acc:HGNC:19745]	protein coding	-0.31	4.97	22.45	<0.0001	0.0008
ENSG00000100284	TOM1	target of myb1 membrane trafficking protein [Source:HGNC Symbol;Acc:HGNC:11982]	protein coding	-0.31	7.75	21.31	<0.0001	0.001
ENSG00000075131	TIPIN	TIMELESS interacting protein [Source:HGNC Symbol;Acc:HGNC:30750]	protein coding	0.31	2.60	9.82	0.0017	0.0451
ENSG00000185340	GAS2L1	growth arrest specific 2 like 1 [Source:HGNC Symbol;Acc:HGNC:16955]	protein coding	-0.31	3.39	13.77	0.0002	0.0113
ENSG00000155465	SLC7A7	solute carrier family 7 member 7 [Source:HGNC Symbol;Acc:HGNC:11065]	protein coding	-0.30	8.55	13.47	0.0002	0.0125
ENSG00000133805	AMPD3	adenosine monophosphate deaminase 3 [Source:HGNC Symbol;Acc:HGNC:470]	protein coding	-0.30	7.38	24.33	<0.0001	0.0005
ENSG00000090376	IRAK3	interleukin 1 receptor associated kinase 3 [Source:HGNC Symbol;Acc:HGNC:17020]	protein coding	-0.30	7.54	13.84	0.0002	0.011
ENSG00000177628	GBA	glucosylceramidase beta [Source:HGNC Symbol;Acc:HGNC:4177]	protein coding	-0.30	6.00	13.54	0.0002	0.0122
ENSG00000116991	SIPA1L2	signal induced proliferation associated 1 like 2 [Source:HGNC Symbol;Acc:HGNC:23800]	protein coding	-0.30	5.31	19.51	<0.0001	0.0016
ENSG00000198715	GLMP	glycosylated lysosomal membrane protein [Source:HGNC Symbol;Acc:HGNC:29436]	protein coding	-0.30	5.44	18.20	<0.0001	0.0023
ENSG00000100600	LGMN	legumain [Source:HGNC Symbol;Acc:HGNC:9472]	protein coding	-0.30	5.29	10.51	0.0012	0.0356
ENSG00000267519			lincRNA	-0.30	2.77	10.39	0.0013	0.0368
ENSG00000105355	PLIN3	perilipin 3 [Source:HGNC Symbol;Acc:HGNC:16893]	protein coding	-0.30	6.33	17.25	<0.0001	0.0032
ENSG00000145014	TMEM44	transmembrane protein 44 [Source:HGNC Symbol;Acc:HGNC:25120]	protein coding	-0.30	2.88	9.92	0.0016	0.0434
ENSG00000042286	AIFM2	apoptosis inducing factor, mitochondria associated 2 [Source:HGNC Symbol;Acc:HGNC:21411]	protein coding	-0.30	4.01	15.71	<0.0001	0.0056

Supplementary Table 10: Genes differentially expressed at day 14 post-second vaccination compared to day 0 (RNA-Seq). Sorted by descending absolute log_2 fold change (Day 14 post-second vaccination vs. Day 0). Gene model summaries and annotations are based on Ensembl Version 90 (August 2017) (continued).

Ensembl Gene ID	Ensembl Gene Name	Ensembl Gene Description	Gene Type	Log_2 Fold Change (Day 14 vs. Day 0)	Average Log_2 CPM	Likelihood Ratio Test Statistic	P-Value	FDR Adjusted P-Value
ENSG00000091436	MAP3K20	mitogen-activated protein kinase kinase kinase 20 [Source:HGNC Symbol;Acc:HGNC:17797]	protein coding	-0.30	6.64	15.39	<0.0001	0.0059
ENSG00000000938	FGR	FGR proto-oncogene, Src family tyrosine kinase [Source:HGNC Symbol;Acc:HGNC:3697]	protein coding	-0.30	7.15	11.98	0.0005	0.0216
ENSG00000140450	ARRDC4	arrestin domain containing 4 [Source:HGNC Symbol;Acc:HGNC:28087]	protein coding	-0.30	5.77	18.91	<0.0001	0.0018
ENSG00000163191	S100A11	S100 calcium binding protein A11 [Source:HGNC Symbol;Acc:HGNC:10488]	protein coding	-0.30	6.81	10.72	0.0011	0.0329
ENSG00000163393	SLC22A15	solute carrier family 22 member 15 [Source:HGNC Symbol;Acc:HGNC:20301]	protein coding	-0.30	3.10	12.71	0.0004	0.0171
ENSG00000185697	MYBL1	MYB proto-oncogene like 1 [Source:HGNC Symbol;Acc:HGNC:7547]	protein coding	0.29	5.45	19.53	<0.0001	0.0016
ENSG00000163874	ZC3H12A	zinc finger CCCH-type containing 12A [Source:HGNC Symbol;Acc:HGNC:26259]	protein coding	-0.29	8.30	17.30	<0.0001	0.0032
ENSG00000149798	CDC42EP2	CDC42 effector protein 2 [Source:HGNC Symbol;Acc:HGNC:16263]	protein coding	-0.29	6.25	18.33	<0.0001	0.0022
ENSG00000133612	AGAP3	ArfGAP with GTPase domain, ankyrin repeat and PH domain 3 [Source:HGNC Symbol;Acc:HGNC:16923]	protein coding	-0.29	6.82	19.48	<0.0001	0.0016
ENSG00000186522	SEPT10	septin 10 [Source:HGNC Symbol;Acc:HGNC:14349]	protein coding	-0.29	3.83	10.68	0.0011	0.0334
ENSG00000056972	TRAF3IP2	TRAF3 interacting protein 2 [Source:HGNC Symbol;Acc:HGNC:1343]	protein coding	-0.29	6.40	20.35	<0.0001	0.0014
ENSG00000162817	C1orf115	chromosome 1 open reading frame 115 [Source:HGNC Symbol;Acc:HGNC:25873]	protein coding	-0.29	3.71	12.20	0.0005	0.02
ENSG00000142694	EVA1B	eva-1 homolog B [Source:HGNC Symbol;Acc:HGNC:25558]	protein coding	-0.29	3.95	10.43	0.0012	0.0366
ENSG00000147454	SLC25A37	solute carrier family 25 member 37 [Source:HGNC Symbol;Acc:HGNC:29786]	protein coding	-0.29	8.04	12.43	0.0004	0.0186
ENSG00000124145	SDC4	syndecan 4 [Source:HGNC Symbol;Acc:HGNC:10661]	protein coding	-0.29	6.05	15.40	<0.0001	0.0059
ENSG00000161638	ITGA5	integrin subunit alpha 5 [Source:HGNC Symbol;Acc:HGNC:6141]	protein coding	-0.29	8.53	13.96	0.0002	0.0107

Supplementary Table 10: Genes differentially expressed at day 14 post-second vaccination compared to day 0 (RNA-Seq). Sorted by descending absolute log_2 fold change (Day 14 post-second vaccination vs. Day 0). Gene model summaries and annotations are based on Ensembl Version 90 (August 2017) (continued).

Ensembl Gene ID	Ensembl Gene Name	Ensembl Gene Description	Gene Type	Log_2 Fold Change (Day 14 vs. Day 0)	Average Log_2 CPM	Likelihood Ratio Test Statistic	P-Value	FDR Adjusted P-Value
ENSG00000183621	ZNF438	zinc finger protein 438 [Source:HGNC Symbol;Acc:HGNC:21029]	protein coding	-0.29	4.39	17.27	<0.0001	0.0032
ENSG00000127946	HIP1	huntingtin interacting protein 1 [Source:HGNC Symbol;Acc:HGNC:4913]	protein coding	-0.29	5.85	11.53	0.0007	0.025
ENSG00000145685	LHFPL2	LHFPL tetraspan subfamily member 2 [Source:HGNC Symbol;Acc:HGNC:6588]	protein coding	-0.29	8.60	11.53	0.0007	0.025
ENSG00000005238	FAM214B	family with sequence similarity 214 member B [Source:HGNC Symbol;Acc:HGNC:25666]	protein coding	-0.29	6.06	15.66	<0.0001	0.0058
ENSG00000186594	MIR22HG	MIR22 host gene [Source:HGNC Symbol;Acc:HGNC:28219]	lincRNA	-0.29	4.72	20.61	<0.0001	0.0013
ENSG00000138111	MFSD13A	major facilitator superfamily domain containing 13A [Source:HGNC Symbol;Acc:HGNC:26196]	protein coding	-0.28	3.61	11.27	0.0008	0.0274
ENSG00000070614	NDST1	N-deacetylase and N-sulfotransferase 1 [Source:HGNC Symbol;Acc:HGNC:7680]	protein coding	-0.28	6.02	11.28	0.0008	0.0274
ENSG00000136026	CKAP4	cytoskeleton associated protein 4 [Source:HGNC Symbol;Acc:HGNC:16991]	protein coding	-0.28	6.99	13.83	0.0002	0.0111
ENSG00000217801			transcribed unprocessed pseudogene	-0.28	3.97	10.20	0.0014	0.0392
ENSG00000125505	MBOAT7	membrane bound O-acyltransferase domain containing 7 [Source:HGNC Symbol;Acc:HGNC:15505]	protein coding	-0.28	7.37	13.24	0.0003	0.0137
ENSG00000090104	RGS1	regulator of G protein signaling 1 [Source:HGNC Symbol;Acc:HGNC:9991]	protein coding	0.28	7.22	17.85	<0.0001	0.0025
ENSG00000197208	SLC22A4	solute carrier family 22 member 4 [Source:HGNC Symbol;Acc:HGNC:10968]	protein coding	-0.28	3.09	11.65	0.0006	0.0241
ENSG00000110880	CORO1C	coronin 1C [Source:HGNC Symbol;Acc:HGNC:2254]	protein coding	-0.28	6.75	15.54	<0.0001	0.0058
ENSG00000075223	SEMA3C	semaphorin 3C [Source:HGNC Symbol;Acc:HGNC:10725]	protein coding	-0.28	6.53	11.88	0.0006	0.0224
ENSG00000197965	MPZL1	myelin protein zero like 1 [Source:HGNC Symbol;Acc:HGNC:7226]	protein coding	-0.28	6.32	15.58	<0.0001	0.0058
ENSG00000163694	RBM47	RNA binding motif protein 47 [Source:HGNC Symbol;Acc:HGNC:30358]	protein coding	-0.28	7.78	12.49	0.0004	0.0183

Supplementary Table 10: Genes differentially expressed at day 14 post-second vaccination compared to day 0 (RNA-Seq). Sorted by descending absolute log_2 fold change (Day 14 post-second vaccination vs. Day 0). Gene model summaries and annotations are based on Ensembl Version 90 (August 2017) (continued).

Ensembl Gene ID	Ensembl Gene Name	Ensembl Gene Description	Gene Type	Log_2 Fold Change (Day 14 vs. Day 0)	Average Log_2 CPM	Likelihood Ratio Test Statistic	P-Value	FDR Adjusted P-Value
ENSG00000176125	UFSP1	UFM1 specific peptidase 1 (inactive) [Source:HGNC Symbol;Acc:HGNC:33821]	protein coding	0.28	2.05	9.75	0.0018	0.0462
ENSG00000116017	ARID3A	AT-rich interaction domain 3A [Source:HGNC Symbol;Acc:HGNC:3031]	protein coding	-0.28	6.70	18.35	<0.0001	0.0022
ENSG00000030582	GRN	granulin precursor [Source:HGNC Symbol;Acc:HGNC:4601]	protein coding	-0.28	8.49	11.15	0.0008	0.029
ENSG00000159399	HK2	hexokinase 2 [Source:HGNC Symbol;Acc:HGNC:4923]	protein coding	-0.28	7.24	23.52	<0.0001	0.0006
ENSG00000148834	GSTO1	glutathione S-transferase omega 1 [Source:HGNC Symbol;Acc:HGNC:13312]	protein coding	-0.28	7.18	17.54	<0.0001	0.0029
ENSG00000182022	CHST15	carbohydrate sulfotransferase 15 [Source:HGNC Symbol;Acc:HGNC:18137]	protein coding	-0.28	7.48	14.67	0.0001	0.0079
ENSG00000136630	HLX	H2.0 like homeobox [Source:HGNC Symbol;Acc:HGNC:4978]	protein coding	-0.28	6.54	20.71	<0.0001	0.0012
ENSG00000138463	DIRC2	disrupted in renal carcinoma 2 [Source:HGNC Symbol;Acc:HGNC:16628]	protein coding	-0.28	4.64	14.20	0.0002	0.0095
ENSG00000134802	SLC43A3	solute carrier family 43 member 3 [Source:HGNC Symbol;Acc:HGNC:17466]	protein coding	-0.28	6.28	21.53	<0.0001	0.001
ENSG00000121797	CCRL2	C-C motif chemokine receptor like 2 [Source:HGNC Symbol;Acc:HGNC:1612]	protein coding	-0.28	6.58	20.78	<0.0001	0.0012
ENSG00000116260	QSOX1	quiescin sulfhydryl oxidase 1 [Source:HGNC Symbol;Acc:HGNC:9756]	protein coding	-0.28	7.43	13.59	0.0002	0.012
ENSG00000156642	NPTN	neuroplastin [Source:HGNC Symbol;Acc:HGNC:17867]	protein coding	-0.28	8.04	12.24	0.0005	0.0196
ENSG00000139117	CPNE8	copine 8 [Source:HGNC Symbol;Acc:HGNC:23498]	protein coding	-0.27	4.18	11.80	0.0006	0.0229
ENSG00000221883	ARIH2OS	ariadne RBR E3 ubiquitin protein ligase 2 opposite strand [Source:HGNC Symbol;Acc:HGNC:34425]	protein coding	0.27	2.44	10.21	0.0014	0.039
ENSG00000130766	SESN2	sestrin 2 [Source:HGNC Symbol;Acc:HGNC:20746]	protein coding	-0.27	5.65	16.05	<0.0001	0.005
ENSG00000160213	CSTB	cystatin B [Source:HGNC Symbol;Acc:HGNC:2482]	protein coding	-0.27	7.47	10.29	0.0013	0.038
ENSG00000110848	CD69	CD69 molecule [Source:HGNC Symbol;Acc:HGNC:1694]	protein coding	0.27	6.37	12.18	0.0005	0.02
ENSG00000134531	EMP1	epithelial membrane protein 1 [Source:HGNC Symbol;Acc:HGNC:3333]	protein coding	-0.27	7.08	13.74	0.0002	0.0114

Supplementary Table 10: Genes differentially expressed at day 14 post-second vaccination compared to day 0 (RNA-Seq). Sorted by descending absolute log_2 fold change (Day 14 post-second vaccination vs. Day 0). Gene model summaries and annotations are based on Ensembl Version 90 (August 2017) (continued).

**Ensembl Gene ID**	**Ensembl Gene Name**	**Ensembl Gene Description**	**Gene Type**	Log_2 **Fold Change (Day 14 vs. Day 0)**	**Average** Log_2 **CPM**	**Likelihood Ratio Test Statistic**	**P-Value**	**FDR Adjusted P-Value**
ENSG00000131669	NINJ1	ninjurin 1 [Source:HGNC Symbol;Acc:HGNC:7824]	protein coding	-0.27	9.40	9.83	0.0017	0.0448
ENSG00000166920	C15orf48	chromosome 15 open reading frame 48 [Source:HGNC Symbol;Acc:HGNC:29898]	protein coding	-0.27	7.10	14.32	0.0002	0.0091
ENSG00000167995	BEST1	bestrophin 1 [Source:HGNC Symbol;Acc:HGNC:12703]	protein coding	-0.27	5.35	19.37	<0.0001	0.0017
ENSG00000171791	BCL2	BCL2, apoptosis regulator [Source:HGNC Symbol;Acc:HGNC:990]	protein coding	0.27	6.74	22.24	<0.0001	0.0008
ENSG00000006534	ALDH3B1	aldehyde dehydrogenase 3 family member B1 [Source:HGNC Symbol;Acc:HGNC:410]	protein coding	-0.27	5.17	12.87	0.0003	0.016
ENSG00000175066	GK5	glycerol kinase 5 (putative) [Source:HGNC Symbol;Acc:HGNC:28635]	protein coding	-0.27	5.86	11.41	0.0007	0.026
ENSG00000116701	NCF2	neutrophil cytosolic factor 2 [Source:HGNC Symbol;Acc:HGNC:7661]	protein coding	-0.27	8.12	12.65	0.0004	0.0175
ENSG00000213903	LTB4R	leukotriene B4 receptor [Source:HGNC Symbol;Acc:HGNC:6713]	protein coding	-0.27	4.38	13.31	0.0003	0.0135
ENSG00000185033	SEMA4B	semaphorin 4B [Source:HGNC Symbol;Acc:HGNC:10730]	protein coding	-0.27	6.20	11.58	0.0007	0.0248
ENSG00000106089	STX1A	syntaxin 1A [Source:HGNC Symbol;Acc:HGNC:11433]	protein coding	-0.27	2.84	10.24	0.0014	0.0387
ENSG00000104763	ASAH1	N-acylsphingosine amidohydrolase 1 [Source:HGNC Symbol;Acc:HGNC:735]	protein coding	-0.27	7.93	10.56	0.0012	0.0351
ENSG00000110911	SLC11A2	solute carrier family 11 member 2 [Source:HGNC Symbol;Acc:HGNC:10908]	protein coding	-0.27	6.97	19.26	<0.0001	0.0017
ENSG00000251230	MIR3945HG	MIR3945 host gene [Source:HGNC Symbol;Acc:HGNC:52002]	lincRNA	-0.26	6.52	15.17	<0.0001	0.0064
ENSG00000039319	ZFYVE16	zinc finger FYVE-type containing 16 [Source:HGNC Symbol;Acc:HGNC:20756]	protein coding	-0.26	6.69	12.65	0.0004	0.0175

Supplementary Table 11: Genes differentially expressed at day 28 post-second vaccination compared to day 0 (RNA-Seq). Sorted by descending absolute log_2 fold change (Day 28 post-second vaccination vs. Day 0). Gene model summaries and annotations are based on Ensembl Version 90 (August 2017).

Ensembl Gene ID	Ensembl Gene Name	Ensembl Gene Description	Gene Type	Log_2 Fold Change (Day 28 vs. Day 0)	Average Log_2 CPM	Likelihood Ratio Test Statistic	P-Value	FDR Adjusted P-Value
ENSG00000127318	IL22	interleukin 22 [Source:HGNC Symbol;Acc:HGNC:14900]	protein coding	1.07	4.81	32.24	<0.0001	<0.0001
ENSG00000278771	RN7SL3	RNA, 7SL, cytoplasmic 3 [Source:HGNC Symbol;Acc:HGNC:23135]	misc RNA	1.01	7.51	20.25	<0.0001	0.0016
ENSG00000206073	SERPINB4	serpin family B member 4 [Source:HGNC Symbol;Acc:HGNC:10570]	protein coding	-0.97	4.43	39.45	<0.0001	<0.0001
ENSG00000112116	IL17F	interleukin 17F [Source:HGNC Symbol;Acc:HGNC:16404]	protein coding	0.97	3.82	17.01	<0.0001	0.0055
ENSG00000174145	NWD2	NACHT and WD repeat domain containing 2 [Source:HGNC Symbol;Acc:HGNC:29229]	protein coding	-0.94	2.28	28.27	<0.0001	0.0001
ENSG00000196611	MMP1	matrix metallopeptidase 1 [Source:HGNC Symbol;Acc:HGNC:7155]	protein coding	-0.92	6.14	25.65	<0.0001	0.0002
ENSG00000131355	ADGRE3	adhesion G protein-coupled receptor E3 [Source:HGNC Symbol;Acc:HGNC:23647]	protein coding	-0.90	2.60	19.17	<0.0001	0.0025
ENSG00000164647	STEAP1	STEAP family member 1 [Source:HGNC Symbol;Acc:HGNC:11378]	protein coding	-0.84	2.07	14.23	0.0002	0.0132
ENSG00000198848	CES1	carboxylesterase 1 [Source:HGNC Symbol;Acc:HGNC:1863]	protein coding	-0.80	5.45	49.33	<0.0001	<0.0001
ENSG00000136235	GPNMB	glycoprotein nmb [Source:HGNC Symbol;Acc:HGNC:4462]	protein coding	-0.80	3.04	18.17	<0.0001	0.0037
ENSG00000087245	MMP2	matrix metallopeptidase 2 [Source:HGNC Symbol;Acc:HGNC:7166]	protein coding	-0.79	2.69	16.34	<0.0001	0.0071
ENSG00000171236	LRG1	leucine rich alpha-2-glycoprotein 1 [Source:HGNC Symbol;Acc:HGNC:29480]	protein coding	-0.77	1.59	27.82	<0.0001	0.0001
ENSG00000109099	PMP22	peripheral myelin protein 22 [Source:HGNC Symbol;Acc:HGNC:9118]	protein coding	-0.77	4.92	29.75	<0.0001	<0.0001
ENSG00000172551	MUCL1	mucin like 1 [Source:HGNC Symbol;Acc:HGNC:30588]	protein coding	-0.74	3.37	23.58	<0.0001	0.0006
ENSG00000111537	IFNG	interferon gamma [Source:HGNC Symbol;Acc:HGNC:5438]	protein coding	0.74	7.35	12.08	0.0005	0.0282
ENSG00000224596	ZMIZ1-AS1	ZMIZ1 antisense RNA 1 [Source:HGNC Symbol;Acc:HGNC:27433]	antisense RNA	-0.71	2.62	29.14	<0.0001	<0.0001
ENSG00000275385	CCL18	C-C motif chemokine ligand 18 [Source:HGNC Symbol;Acc:HGNC:10616]	protein coding	-0.70	4.73	26.78	<0.0001	0.0001

Supplementary Table 11: Genes differentially expressed at day 28 post-second vaccination compared to day 0 (RNA-Seq). Sorted by descending absolute log_2 fold change (Day 28 post-second vaccination vs. Day 0). Gene model summaries and annotations are based on Ensembl Version 90 (August 2017) (continued).

**Ensembl Gene ID**	**Ensembl Gene Name**	**Ensembl Gene Description**	**Gene Type**	**Log_2 Fold Change (Day 28 vs. Day 0)**	**Average Log_2 CPM**	**Likelihood Ratio Test Statistic**	**P-Value**	**FDR Adjusted P-Value**
ENSG00000183019	MCEMP1	mast cell expressed membrane protein 1 [Source:HGNC Symbol;Acc:HGNC:27291]	protein coding	-0.69	3.43	20.82	<0.0001	0.0014
ENSG00000087116	ADAMTS2	ADAM metallopeptidase with thrombospondin type 1 motif 2 [Source:HGNC Symbol;Acc:HGNC:218]	protein coding	-0.69	1.60	19.29	<0.0001	0.0024
ENSG00000120708	TGFBI	transforming growth factor beta induced [Source:HGNC Symbol;Acc:HGNC:11771]	protein coding	-0.68	5.07	19.92	<0.0001	0.0019
ENSG00000174705	SH3PXD2B	SH3 and PX domains 2B [Source:HGNC Symbol;Acc:HGNC:29242]	protein coding	-0.67	7.09	31.90	<0.0001	<0.0001
ENSG00000005102	MEOX1	mesenchyme homeobox 1 [Source:HGNC Symbol;Acc:HGNC:7013]	protein coding	0.67	1.16	12.33	0.0004	0.0258
ENSG00000104918	RETN	resistin [Source:HGNC Symbol;Acc:HGNC:20389]	protein coding	-0.66	3.22	11.19	0.0008	0.0376
ENSG00000074410	CA12	carbonic anhydrase 12 [Source:HGNC Symbol;Acc:HGNC:1371]	protein coding	-0.63	6.63	20.62	<0.0001	0.0014
ENSG00000164266	SPINK1	serine peptidase inhibitor, Kazal type 1 [Source:HGNC Symbol;Acc:HGNC:11244]	protein coding	-0.63	1.64	10.48	0.0012	0.0465
ENSG00000169908	TM4SF1	transmembrane 4 L six family member 1 [Source:HGNC Symbol;Acc:HGNC:11853]	protein coding	-0.63	4.51	13.30	0.0003	0.0188
ENSG00000162494	LRRC38	leucine rich repeat containing 38 [Source:HGNC Symbol;Acc:HGNC:27005]	protein coding	-0.62	2.07	18.87	<0.0001	0.0028
ENSG00000187957	DNER	delta/notch like EGF repeat containing [Source:HGNC Symbol;Acc:HGNC:24456]	protein coding	-0.62	2.87	11.12	0.0009	0.038
ENSG00000142405	NLRP12	NLR family pyrin domain containing 12 [Source:HGNC Symbol;Acc:HGNC:22938]	protein coding	-0.62	2.38	17.70	<0.0001	0.0043
ENSG00000153208	MERTK	MER proto-oncogene, tyrosine kinase [Source:HGNC Symbol;Acc:HGNC:7027]	protein coding	-0.62	4.91	15.94	<0.0001	0.008
ENSG00000259207	ITGB3	integrin subunit beta 3 [Source:HGNC Symbol;Acc:HGNC:6156]	protein coding	-0.61	3.85	22.12	<0.0001	0.001
ENSG00000148344	PTGES	prostaglandin E synthase [Source:HGNC Symbol;Acc:HGNC:9599]	protein coding	-0.61	6.53	12.44	0.0004	0.0246
ENSG00000122861	PLAU	plasminogen activator, urokinase [Source:HGNC Symbol;Acc:HGNC:9052]	protein coding	-0.61	5.37	32.57	<0.0001	<0.0001
ENSG00000162493	PDPN	podoplanin [Source:HGNC Symbol;Acc:HGNC:29602]	protein coding	-0.61	5.67	20.73	<0.0001	0.0014

Supplementary Table 11: Genes differentially expressed at day 28 post-second vaccination compared to day 0 (RNA-Seq). Sorted by descending absolute log_2 fold change (Day 28 post-second vaccination vs. Day 0). Gene model summaries and annotations are based on Ensembl Version 90 (August 2017) (continued).

**Ensembl Gene ID**	**Ensembl Gene Name**	**Ensembl Gene Description**	**Gene Type**	Log_2 **Fold Change (Day 28 vs. Day 0)**	**Average** Log_2 **CPM**	**Likelihood Ratio Test Statistic**	**P-Value**	**FDR Adjusted P-Value**
ENSG00000100336	APOL4	apolipoprotein L4 [Source:HGNC Symbol;Acc:HGNC:14867]	protein coding	0.60	2.00	12.02	0.0005	0.0285
ENSG00000162745	OLFML2B	olfactomedin like 2B [Source:HGNC Symbol;Acc:HGNC:24558]	protein coding	-0.60	4.92	18.79	<0.0001	0.0029
ENSG00000168874	ATOH8	atonal bHLH transcription factor 8 [Source:HGNC Symbol;Acc:HGNC:24126]	protein coding	-0.60	1.61	10.93	0.0009	0.0402
ENSG00000186827	TNFRSF4	TNF receptor superfamily member 4 [Source:HGNC Symbol;Acc:HGNC:11918]	protein coding	0.60	5.50	53.48	<0.0001	<0.0001
ENSG00000164400	CSF2	colony stimulating factor 2 [Source:HGNC Symbol;Acc:HGNC:2434]	protein coding	0.60	3.28	11.99	0.0005	0.0285
ENSG00000225556	C2CD4D	C2 calcium dependent domain containing 4D [Source:HGNC Symbol;Acc:HGNC:37210]	protein coding	0.59	1.25	10.95	0.0009	0.0399
ENSG00000225107			lincRNA	-0.59	2.20	12.72	0.0004	0.0226
ENSG00000196549	MME	membrane metalloendopeptidase [Source:HGNC Symbol;Acc:HGNC:7154]	protein coding	-0.58	3.10	23.37	<0.0001	0.0006
ENSG00000135929	CYP27A1	cytochrome P450 family 27 subfamily A member 1 [Source:HGNC Symbol;Acc:HGNC:2605]	protein coding	-0.58	7.17	17.53	<0.0001	0.0045
ENSG00000196209	SIRPB2	signal regulatory protein beta 2 [Source:HGNC Symbol;Acc:HGNC:16247]	protein coding	-0.58	3.28	15.55	<0.0001	0.0089
ENSG00000211659	IGLV3-25	immunoglobulin lambda variable 3-25 [Source:HGNC Symbol;Acc:HGNC:5908]	IG V gene	0.58	2.00	11.75	0.0006	0.0306
ENSG00000154856	APCDD1	APC down-regulated 1 [Source:HGNC Symbol;Acc:HGNC:15718]	protein coding	-0.57	4.31	27.10	<0.0001	0.0001
ENSG00000039560	RAI14	retinoic acid induced 14 [Source:HGNC Symbol;Acc:HGNC:14873]	protein coding	-0.57	2.82	25.32	<0.0001	0.0002
ENSG00000166396	SERPINB7	serpin family B member 7 [Source:HGNC Symbol;Acc:HGNC:13902]	protein coding	-0.57	7.22	25.91	<0.0001	0.0002
ENSG00000182578	CSF1R	colony stimulating factor 1 receptor [Source:HGNC Symbol;Acc:HGNC:2433]	protein coding	-0.57	6.82	12.11	0.0005	0.0282
ENSG00000179241	LDLRAD3	low density lipoprotein receptor class A domain containing 3 [Source:HGNC Symbol;Acc:HGNC:27046]	protein coding	-0.56	4.87	20.93	<0.0001	0.0014
ENSG00000093134	VNN3	vanin 3 [Source:HGNC Symbol;Acc:HGNC:16431]	protein coding	-0.56	2.90	15.91	<0.0001	0.008

Supplementary Table 11: Genes differentially expressed at day 28 post-second vaccination compared to day 0 (RNA-Seq). Sorted by descending absolute log_2 fold change (Day 28 post-second vaccination vs. Day 0). Gene model summaries and annotations are based on Ensembl Version 90 (August 2017) (continued).

Ensembl Gene ID	Ensembl Gene Name	Ensembl Gene Description	Gene Type	Log_2 Fold Change (Day 28 vs. Day 0)	Average Log_2 CPM	Likelihood Ratio Test Statistic	P-Value	FDR Adjusted P-Value
ENSG00000106853	PTGR1	prostaglandin reductase 1 [Source:HGNC Symbol;Acc:HGNC:18429]	protein coding	-0.56	3.48	18.19	<0.0001	0.0037
ENSG00000171631	P2RY6	pyrimidinergic receptor P2Y6 [Source:HGNC Symbol;Acc:HGNC:8543]	protein coding	-0.56	4.59	21.66	<0.0001	0.0011
ENSG00000163221	S100A12	S100 calcium binding protein A12 [Source:HGNC Symbol;Acc:HGNC:10489]	protein coding	-0.56	5.85	12.80	0.0003	0.0219
ENSG00000167850	CD300C	CD300c molecule [Source:HGNC Symbol;Acc:HGNC:19320]	protein coding	-0.55	3.88	29.88	<0.0001	<0.0001
ENSG00000113749	HRH2	histamine receptor H2 [Source:HGNC Symbol;Acc:HGNC:5183]	protein coding	-0.55	4.02	13.67	0.0002	0.0161
ENSG00000185052	SLC24A3	solute carrier family 24 member 3 [Source:HGNC Symbol;Acc:HGNC:10977]	protein coding	-0.54	3.49	20.98	<0.0001	0.0014
ENSG00000170458	CD14	CD14 molecule [Source:HGNC Symbol;Acc:HGNC:1628]	protein coding	-0.54	9.80	12.83	0.0003	0.0219
ENSG00000035862	TIMP2	TIMP metallopeptidase inhibitor 2 [Source:HGNC Symbol;Acc:HGNC:11821]	protein coding	-0.54	4.58	28.04	<0.0001	0.0001
ENSG00000018280	SLC11A1	solute carrier family 11 member 1 [Source:HGNC Symbol;Acc:HGNC:10907]	protein coding	-0.54	8.70	19.43	<0.0001	0.0023
ENSG00000139354	GAS2L3	growth arrest specific 2 like 3 [Source:HGNC Symbol;Acc:HGNC:27475]	protein coding	-0.53	2.52	15.87	<0.0001	0.008
ENSG00000125730	C3	complement C3 [Source:HGNC Symbol;Acc:HGNC:1318]	protein coding	-0.53	6.88	21.55	<0.0001	0.0011
ENSG00000100985	MMP9	matrix metallopeptidase 9 [Source:HGNC Symbol;Acc:HGNC:7176]	protein coding	-0.53	8.49	15.40	<0.0001	0.0092
ENSG00000128578	STRIP2	striatin interacting protein 2 [Source:HGNC Symbol;Acc:HGNC:22209]	protein coding	-0.52	3.53	31.01	<0.0001	<0.0001
ENSG00000143546	S100A8	S100 calcium binding protein A8 [Source:HGNC Symbol;Acc:HGNC:10498]	protein coding	-0.51	7.75	13.08	0.0003	0.0197
ENSG00000134028	ADAMDEC1	ADAM like decysin 1 [Source:HGNC Symbol;Acc:HGNC:16299]	protein coding	-0.51	7.43	13.67	0.0002	0.0161
ENSG00000153071	DAB2	DAB2, clathrin adaptor protein [Source:HGNC Symbol;Acc:HGNC:2662]	protein coding	-0.51	5.42	27.61	<0.0001	0.0001
ENSG00000198682	PAPSS2	3'-phosphoadenosine 5'-phosphosulfate synthase 2 [Source:HGNC Symbol;Acc:HGNC:8604]	protein coding	-0.51	6.07	20.28	<0.0001	0.0016

Supplementary Table 11: Genes differentially expressed at day 28 post-second vaccination compared to day 0 (RNA-Seq). Sorted by descending absolute log_2 fold change (Day 28 post-second vaccination vs. Day 0). Gene model summaries and annotations are based on Ensembl Version 90 (August 2017) (continued).

Ensembl Gene ID	Ensembl Gene Name	Ensembl Gene Description	Gene Type	Log_2 Fold Change (Day 28 vs. Day 0)	Average Log_2 CPM	Likelihood Ratio Test Statistic	P-Value	FDR Adjusted P-Value
ENSG00000107249	GLIS3	GLIS family zinc finger 3 [Source:HGNC Symbol;Acc:HGNC:28510]	protein coding	-0.50	5.48	25.96	<0.0001	0.0002
ENSG00000163220	S100A9	S100 calcium binding protein A9 [Source:HGNC Symbol;Acc:HGNC:10499]	protein coding	-0.50	11.16	10.87	0.001	0.0408
ENSG00000099250	NRP1	neuropilin 1 [Source:HGNC Symbol;Acc:HGNC:8004]	protein coding	-0.50	6.56	27.05	<0.0001	0.0001
ENSG00000124216	SNAI1	snail family transcriptional repressor 1 [Source:HGNC Symbol;Acc:HGNC:11128]	protein coding	-0.49	3.02	13.84	0.0002	0.0151
ENSG00000145555	MYO10	myosin X [Source:HGNC Symbol;Acc:HGNC:7593]	protein coding	-0.49	3.71	20.82	<0.0001	0.0014
ENSG00000091106	NLRC4	NLR family CARD domain containing 4 [Source:HGNC Symbol;Acc:HGNC:16412]	protein coding	-0.49	4.77	11.44	0.0007	0.0345
ENSG00000112394	SLC16A10	solute carrier family 16 member 10 [Source:HGNC Symbol;Acc:HGNC:17027]	protein coding	-0.48	6.88	15.57	<0.0001	0.0089
ENSG00000284486			processed transcript	-0.48	1.51	12.51	0.0004	0.0241
ENSG00000134830	C5AR2	complement component 5a receptor 2 [Source:HGNC Symbol;Acc:HGNC:4527]	protein coding	-0.47	2.52	10.37	0.0013	0.0482
ENSG00000112715	VEGFA	vascular endothelial growth factor A [Source:HGNC Symbol;Acc:HGNC:12680]	protein coding	-0.47	6.48	24.60	<0.0001	0.0003
ENSG00000127838	PNKD	paroxysmal nonkinesigenic dyskinesia [Source:HGNC Symbol;Acc:HGNC:9153]	protein coding	-0.47	6.59	32.63	<0.0001	<0.0001
ENSG00000265100			antisense RNA	0.47	1.38	11.60	0.0007	0.0326
ENSG00000073792	IGF2BP2	insulin like growth factor 2 mRNA binding protein 2 [Source:HGNC Symbol;Acc:HGNC:28867]	protein coding	-0.47	2.38	11.55	0.0007	0.033
ENSG00000114251	WNT5A	Wnt family member 5A [Source:HGNC Symbol;Acc:HGNC:12784]	protein coding	-0.47	5.59	12.00	0.0005	0.0285
ENSG00000146072	TNFRSF21	TNF receptor superfamily member 21 [Source:HGNC Symbol;Acc:HGNC:13469]	protein coding	-0.47	5.67	11.33	0.0008	0.0357
ENSG00000150760	DOCK1	dedicator of cytokinesis 1 [Source:HGNC Symbol;Acc:HGNC:2987]	protein coding	-0.47	2.42	13.65	0.0002	0.0161
ENSG00000100292	HMOX1	heme oxygenase 1 [Source:HGNC Symbol;Acc:HGNC:5013]	protein coding	-0.46	7.25	21.53	<0.0001	0.0011
ENSG00000135373	EHF	ETS homologous factor [Source:HGNC Symbol;Acc:HGNC:3246]	protein coding	-0.46	3.34	12.10	0.0005	0.0282

Supplementary Table 11: Genes differentially expressed at day 28 post-second vaccination compared to day 0 (RNA-Seq). Sorted by descending absolute log_2 fold change (Day 28 post-second vaccination vs. Day 0). Gene model summaries and annotations are based on Ensembl Version 90 (August 2017) (continued).

Ensembl Gene ID	Ensembl Gene Name	Ensembl Gene Description	Gene Type	Log_2 Fold Change (Day 28 vs. Day 0)	Average Log_2 CPM	Likelihood Ratio Test Statistic	P-Value	FDR Adjusted P-Value
ENSG00000142185	TRPM2	transient receptor potential cation channel subfamily M member 2 [Source:HGNC Symbol;Acc:HGNC:12339]	protein coding	-0.46	6.03	30.63	<0.0001	<0.0001
ENSG00000003436	TFPI	tissue factor pathway inhibitor [Source:HGNC Symbol;Acc:HGNC:11760]	protein coding	-0.46	4.63	20.68	<0.0001	0.0014
ENSG00000124731	TREM1	triggering receptor expressed on myeloid cells 1 [Source:HGNC Symbol;Acc:HGNC:17760]	protein coding	-0.45	8.05	21.99	<0.0001	0.001
ENSG00000280254			TEC	-0.45	2.34	14.56	0.0001	0.0121
ENSG00000143545	RAB13	RAB13, member RAS oncogene family [Source:HGNC Symbol;Acc:HGNC:9762]	protein coding	-0.45	5.22	14.73	0.0001	0.0114
ENSG00000002587	HS3ST1	heparan sulfate-glucosamine 3-sulfotransferase 1 [Source:HGNC Symbol;Acc:HGNC:5194]	protein coding	-0.45	5.10	15.96	<0.0001	0.008
ENSG00000213694	S1PR3	sphingosine-1-phosphate receptor 3 [Source:HGNC Symbol;Acc:HGNC:3167]	protein coding	-0.45	2.16	14.96	0.0001	0.0106
ENSG00000268734			lincRNA	-0.45	4.76	16.41	<0.0001	0.0069
ENSG00000125810	CD93	CD93 molecule [Source:HGNC Symbol;Acc:HGNC:15855]	protein coding	-0.44	9.75	13.40	0.0003	0.018
ENSG00000008394	MGST1	microsomal glutathione S-transferase 1 [Source:HGNC Symbol;Acc:HGNC:7061]	protein coding	-0.44	3.83	15.74	<0.0001	0.0085
ENSG00000225774	SIRPAP1	signal regulatory protein alpha pseudogene 1 [Source:HGNC Symbol;Acc:HGNC:9663]	processed pseudogene	-0.44	1.58	10.60	0.0011	0.0446
ENSG00000105520	PLPPR2	phospholipid phosphatase related 2 [Source:HGNC Symbol;Acc:HGNC:29566]	protein coding	-0.44	3.29	12.68	0.0004	0.0226
ENSG00000142512	SIGLEC10	sialic acid binding Ig like lectin 10 [Source:HGNC Symbol;Acc:HGNC:15620]	protein coding	-0.44	4.14	11.76	0.0006	0.0306
ENSG00000197405	C5AR1	complement C5a receptor 1 [Source:HGNC Symbol;Acc:HGNC:1338]	protein coding	-0.43	8.36	15.70	<0.0001	0.0085
ENSG00000168461	RAB31	RAB31, member RAS oncogene family [Source:HGNC Symbol;Acc:HGNC:9771]	protein coding	-0.43	7.79	20.69	<0.0001	0.0014
ENSG00000100504	PYGL	glycogen phosphorylase L [Source:HGNC Symbol;Acc:HGNC:9725]	protein coding	-0.43	5.46	12.24	0.0005	0.0268
ENSG00000124882	EREG	epiregulin [Source:HGNC Symbol;Acc:HGNC:3443]	protein coding	-0.43	7.77	10.70	0.0011	0.0431

Supplementary Table 11: Genes differentially expressed at day 28 post-second vaccination compared to day 0 (RNA-Seq). Sorted by descending absolute log_2 fold change (Day 28 post-second vaccination vs. Day 0). Gene model summaries and annotations are based on Ensembl Version 90 (August 2017) (continued).

Ensembl Gene ID	Ensembl Gene Name	Ensembl Gene Description	Gene Type	Log_2 Fold Change (Day 28 vs. Day 0)	Average Log_2 CPM	Likelihood Ratio Test Statistic	P-Value	FDR Adjusted P-Value
ENSG00000169122	FAM110B	family with sequence similarity 110 member B [Source:HGNC Symbol;Acc:HGNC:28587]	protein coding	-0.43	1.87	10.48	0.0012	0.0465
ENSG00000189060	H1F0	H1 histone family member 0 [Source:HGNC Symbol;Acc:HGNC:4714]	protein coding	-0.43	5.19	11.87	0.0006	0.0296
ENSG00000186407	CD300E	CD300e molecule [Source:HGNC Symbol;Acc:HGNC:28874]	protein coding	-0.43	9.43	11.38	0.0007	0.0351
ENSG00000224397	SMIM25	small integral membrane protein 25 [Source:HGNC Symbol;Acc:HGNC:50328]	lincRNA	-0.43	4.48	17.05	<0.0001	0.0055
ENSG00000138061	CYP1B1	cytochrome P450 family 1 subfamily B member 1 [Source:HGNC Symbol;Acc:HGNC:2597]	protein coding	-0.43	8.64	12.68	0.0004	0.0226
ENSG00000143226	FCGR2A	Fc fragment of IgG receptor IIa [Source:HGNC Symbol;Acc:HGNC:3616]	protein coding	-0.42	7.11	21.86	<0.0001	0.001
ENSG00000175352	NRIP3	nuclear receptor interacting protein 3 [Source:HGNC Symbol;Acc:HGNC:1167]	protein coding	-0.42	5.63	23.01	<0.0001	0.0007
ENSG00000169860	P2RY1	purinergic receptor P2Y1 [Source:HGNC Symbol;Acc:HGNC:8539]	protein coding	-0.42	2.62	12.55	0.0004	0.0237
ENSG00000136634	IL10	interleukin 10 [Source:HGNC Symbol;Acc:HGNC:5962]	protein coding	-0.42	6.18	19.17	<0.0001	0.0025
ENSG00000144959	NCEH1	neutral cholesterol ester hydrolase 1 [Source:HGNC Symbol;Acc:HGNC:29260]	protein coding	-0.42	4.76	15.04	0.0001	0.0105
ENSG00000167680	SEMA6B	semaphorin 6B [Source:HGNC Symbol;Acc:HGNC:10739]	protein coding	-0.42	7.96	12.97	0.0003	0.0206
ENSG00000088826	SMOX	spermine oxidase [Source:HGNC Symbol;Acc:HGNC:15862]	protein coding	-0.42	5.28	19.06	<0.0001	0.0026
ENSG00000254415	SIGLEC14	sialic acid binding Ig like lectin 14 [Source:HGNC Symbol;Acc:HGNC:32926]	protein coding	-0.41	6.50	13.08	0.0003	0.0197
ENSG00000088543	C3orf18	chromosome 3 open reading frame 18 [Source:HGNC Symbol;Acc:HGNC:24837]	protein coding	0.41	2.82	17.94	<0.0001	0.0039
ENSG00000115107	STEAP3	STEAP3 metalloreductase [Source:HGNC Symbol;Acc:HGNC:24592]	protein coding	-0.41	4.94	16.63	<0.0001	0.0063
ENSG00000169439	SDC2	syndecan 2 [Source:HGNC Symbol;Acc:HGNC:10659]	protein coding	-0.41	8.44	12.67	0.0004	0.0226
ENSG00000180113	TDRD6	tudor domain containing 6 [Source:HGNC Symbol;Acc:HGNC:21339]	protein coding	-0.41	5.30	18.77	<0.0001	0.0029

Supplementary Table 11: Genes differentially expressed at day 28 post-second vaccination compared to day 0 (RNA-Seq). Sorted by descending absolute log_2 fold change (Day 28 post-second vaccination vs. Day 0). Gene model summaries and annotations are based on Ensembl Version 90 (August 2017) (continued).

Ensembl Gene ID	Ensembl Gene Name	Ensembl Gene Description	Gene Type	Log_2 Fold Change (Day 28 vs. Day 0)	Average Log_2 CPM	Likelihood Ratio Test Statistic	P-Value	FDR Adjusted P-Value
ENSG00000167703	SLC43A2	solute carrier family 43 member 2 [Source:HGNC Symbol;Acc:HGNC:23087]	protein coding	-0.41	9.75	14.14	0.0002	0.0135
ENSG00000051128	HOMER3	homer scaffolding protein 3 [Source:HGNC Symbol;Acc:HGNC:17514]	protein coding	-0.40	3.09	10.52	0.0012	0.0461
ENSG00000100767	PAPLN	papilin, proteoglycan like sulfated glycoprotein [Source:HGNC Symbol;Acc:HGNC:19262]	protein coding	-0.40	4.60	15.66	<0.0001	0.0086
ENSG00000105976	MET	MET proto-oncogene, receptor tyrosine kinase [Source:HGNC Symbol;Acc:HGNC:7029]	protein coding	-0.40	8.36	15.49	<0.0001	0.009
ENSG00000115594	IL1R1	interleukin 1 receptor type 1 [Source:HGNC Symbol;Acc:HGNC:5993]	protein coding	-0.40	6.35	14.23	0.0002	0.0132
ENSG00000145936	KCNMB1	potassium calcium-activated channel subfamily M regulatory beta subunit 1 [Source:HGNC Symbol;Acc:HGNC:6285]	protein coding	-0.40	2.20	13.71	0.0002	0.0161
ENSG00000165801	ARHGEF40	Rho guanine nucleotide exchange factor 40 [Source:HGNC Symbol;Acc:HGNC:25516]	protein coding	-0.40	4.19	10.66	0.0011	0.0437
ENSG00000166825	ANPEP	alanyl aminopeptidase, membrane [Source:HGNC Symbol;Acc:HGNC:500]	protein coding	-0.40	8.86	15.15	<0.0001	0.0101
ENSG00000197249	SERPINA1	serpin family A member 1 [Source:HGNC Symbol;Acc:HGNC:8941]	protein coding	-0.40	9.39	14.41	0.0001	0.0129
ENSG00000138448	ITGAV	integrin subunit alpha V [Source:HGNC Symbol;Acc:HGNC:6150]	protein coding	-0.40	8.09	10.82	0.001	0.0416
ENSG00000168615	ADAM9	ADAM metallopeptidase domain 9 [Source:HGNC Symbol;Acc:HGNC:216]	protein coding	-0.40	7.63	13.21	0.0003	0.0192
ENSG00000151012	SLC7A11	solute carrier family 7 member 11 [Source:HGNC Symbol;Acc:HGNC:11059]	protein coding	-0.39	9.51	11.93	0.0006	0.029
ENSG00000011422	PLAUR	plasminogen activator, urokinase receptor [Source:HGNC Symbol;Acc:HGNC:9053]	protein coding	-0.39	10.18	11.10	0.0009	0.0382
ENSG00000102471	NDFIP2	Nedd4 family interacting protein 2 [Source:HGNC Symbol;Acc:HGNC:18537]	protein coding	0.39	3.53	10.69	0.0011	0.0431
ENSG00000158869	FCER1G	Fc fragment of IgE receptor Ig [Source:HGNC Symbol;Acc:HGNC:3611]	protein coding	-0.39	9.37	11.80	0.0006	0.0302
ENSG00000050730	TNIP3	TNFAIP3 interacting protein 3 [Source:HGNC Symbol;Acc:HGNC:19315]	protein coding	-0.39	8.44	15.40	<0.0001	0.0092
ENSG00000225963			antisense RNA	0.39	2.16	10.48	0.0012	0.0465

Supplementary Table 11: Genes differentially expressed at day 28 post-second vaccination compared to day 0 (RNA-Seq). Sorted by descending absolute log_2 fold change (Day 28 post-second vaccination vs. Day 0). Gene model summaries and annotations are based on Ensembl Version 90 (August 2017) (continued).

Ensembl Gene ID	Ensembl Gene Name	Ensembl Gene Description	Gene Type	Log_2 Fold Change (Day 28 vs. Day 0)	Average Log_2 CPM	Likelihood Ratio Test Statistic	P-Value	FDR Adjusted P-Value
ENSG00000165474	GJB2	gap junction protein beta 2 [Source:HGNC Symbol;Acc:HGNC:4284]	protein coding	-0.39	7.76	13.16	0.0003	0.0193
ENSG00000250274			lincRNA	-0.39	2.75	10.86	0.001	0.0408
ENSG00000181019	NQO1	NAD(P)H quinone dehydrogenase 1 [Source:HGNC Symbol;Acc:HGNC:2874]	protein coding	-0.39	4.36	16.12	<0.0001	0.0077
ENSG00000150510	FAM124A	family with sequence similarity 124 member A [Source:HGNC Symbol;Acc:HGNC:26413]	protein coding	-0.39	4.45	18.23	<0.0001	0.0037
ENSG00000140848	CPNE2	copine 2 [Source:HGNC Symbol;Acc:HGNC:2315]	protein coding	-0.39	4.87	23.18	<0.0001	0.0006
ENSG00000169902	TPST1	tyrosylprotein sulfotransferase 1 [Source:HGNC Symbol;Acc:HGNC:12020]	protein coding	-0.39	3.29	10.72	0.0011	0.0429
ENSG00000103569	AQP9	aquaporin 9 [Source:HGNC Symbol;Acc:HGNC:643]	protein coding	-0.38	9.66	11.88	0.0006	0.0296
ENSG00000129450	SIGLEC9	sialic acid binding Ig like lectin 9 [Source:HGNC Symbol;Acc:HGNC:10878]	protein coding	-0.38	6.25	25.45	<0.0001	0.0002
ENSG00000166927	MS4A7	membrane spanning 4-domains A7 [Source:HGNC Symbol;Acc:HGNC:13378]	protein coding	-0.38	7.10	11.02	0.0009	0.0392
ENSG00000175489	LRRC25	leucine rich repeat containing 25 [Source:HGNC Symbol;Acc:HGNC:29806]	protein coding	-0.38	8.52	14.73	0.0001	0.0114
ENSG00000145014	TMEM44	transmembrane protein 44 [Source:HGNC Symbol;Acc:HGNC:25120]	protein coding	-0.38	2.86	15.50	<0.0001	0.009
ENSG00000020181	ADGRA2	adhesion G protein-coupled receptor A2 [Source:HGNC Symbol;Acc:HGNC:17849]	protein coding	-0.38	5.08	12.65	0.0004	0.0227
ENSG00000155465	SLC7A7	solute carrier family 7 member 7 [Source:HGNC Symbol;Acc:HGNC:11065]	protein coding	-0.38	8.55	16.27	<0.0001	0.0073
ENSG00000163191	S100A11	S100 calcium binding protein A11 [Source:HGNC Symbol;Acc:HGNC:10488]	protein coding	-0.38	6.82	16.72	<0.0001	0.0062
ENSG00000153162	BMP6	bone morphogenetic protein 6 [Source:HGNC Symbol;Acc:HGNC:1073]	protein coding	-0.38	3.64	13.08	0.0003	0.0197
ENSG00000239653	PSMD6-AS2	PSMD6 antisense RNA 2 [Source:HGNC Symbol;Acc:HGNC:44125]	antisense RNA	0.38	2.72	12.01	0.0005	0.0285
ENSG00000139572	GPR84	G protein-coupled receptor 84 [Source:HGNC Symbol;Acc:HGNC:4535]	protein coding	-0.37	6.91	13.01	0.0003	0.0202
ENSG00000196923	PDLIM7	PDZ and LIM domain 7 [Source:HGNC Symbol;Acc:HGNC:22958]	protein coding	-0.37	4.85	17.62	<0.0001	0.0044

Supplementary Table 11: Genes differentially expressed at day 28 post-second vaccination compared to day 0 (RNA-Seq). Sorted by descending absolute log_2 fold change (Day 28 post-second vaccination vs. Day 0). Gene model summaries and annotations are based on Ensembl Version 90 (August 2017) (continued).

Ensembl Gene ID	Ensembl Gene Name	Ensembl Gene Description	Gene Type	Log_2 Fold Change (Day 28 vs. Day 0)	Average Log_2 CPM	Likelihood Ratio Test Statistic	P-Value	FDR Adjusted P-Value
ENSG00000185697	MYBL1	MYB proto-oncogene like 1 [Source:HGNC Symbol;Acc:HGNC:7547]	protein coding	0.37	5.53	27.77	<0.0001	0.0001
ENSG00000260549	MT1L	metallothionein 1L, pseudogene [Source:HGNC Symbol;Acc:HGNC:7404]	transcribed unitary pseudogene	-0.37	6.58	10.28	0.0013	0.0496
ENSG00000242048			processed transcript	-0.37	3.82	11.43	0.0007	0.0346
ENSG00000173918	C1QTNF1	C1q and TNF related 1 [Source:HGNC Symbol;Acc:HGNC:14324]	protein coding	-0.37	7.84	17.31	<0.0001	0.0049
ENSG00000215424	MCM3AP-AS1	MCM3AP antisense RNA 1 [Source:HGNC Symbol;Acc:HGNC:16417]	antisense RNA	0.37	2.17	10.48	0.0012	0.0465
ENSG00000229644	NAMPTP1	nicotinamide phosphoribosyltransferase pseudogene 1 [Source:HGNC Symbol;Acc:HGNC:17633]	processed pseudogene	-0.37	8.45	12.32	0.0004	0.0259
ENSG00000123342	MMP19	matrix metallopeptidase 19 [Source:HGNC Symbol;Acc:HGNC:7165]	protein coding	-0.37	7.45	16.71	<0.0001	0.0062
ENSG00000136689	IL1RN	interleukin 1 receptor antagonist [Source:HGNC Symbol;Acc:HGNC:6000]	protein coding	-0.37	8.75	13.64	0.0002	0.0162
ENSG00000075651	PLD1	phospholipase D1 [Source:HGNC Symbol;Acc:HGNC:9067]	protein coding	-0.37	6.71	20.41	<0.0001	0.0015
ENSG00000085514	PILRA	paired immunoglobin like type 2 receptor alpha [Source:HGNC Symbol;Acc:HGNC:20396]	protein coding	-0.37	8.11	13.20	0.0003	0.0192
ENSG00000101336	HCK	HCK proto-oncogene, Src family tyrosine kinase [Source:HGNC Symbol;Acc:HGNC:4840]	protein coding	-0.36	9.62	12.05	0.0005	0.0283
ENSG00000111424	VDR	vitamin D receptor [Source:HGNC Symbol;Acc:HGNC:12679]	protein coding	-0.36	5.93	22.08	<0.0001	0.001
ENSG00000099985	OSM	oncostatin M [Source:HGNC Symbol;Acc:HGNC:8506]	protein coding	-0.36	7.82	14.16	0.0002	0.0135
ENSG00000113070	HBEGF	heparin binding EGF like growth factor [Source:HGNC Symbol;Acc:HGNC:3059]	protein coding	-0.36	6.70	12.70	0.0004	0.0226
ENSG00000198853	RUSC2	RUN and SH3 domain containing 2 [Source:HGNC Symbol;Acc:HGNC:23625]	protein coding	-0.36	5.95	14.26	0.0002	0.0132
ENSG00000167613	LAIR1	leukocyte associated immunoglobulin like receptor 1 [Source:HGNC Symbol;Acc:HGNC:6477]	protein coding	-0.36	8.22	15.30	<0.0001	0.0096

Supplementary Table 11: Genes differentially expressed at day 28 post-second vaccination compared to day 0 (RNA-Seq). Sorted by descending absolute log_2 fold change (Day 28 post-second vaccination vs. Day 0). Gene model summaries and annotations are based on Ensembl Version 90 (August 2017) (continued).

**Ensembl Gene ID**	**Ensembl Gene Name**	**Ensembl Gene Description**	**Gene Type**	Log_2 **Fold Change (Day 28 vs. Day 0)**	**Average** Log_2 **CPM**	**Likelihood Ratio Test Statistic**	**P-Value**	**FDR Adjusted P-Value**
ENSG00000145649	GZMA	granzyme A [Source:HGNC Symbol;Acc:HGNC:4708]	protein coding	0.36	5.50	17.81	<0.0001	0.0042
ENSG00000173083	HPSE	heparanase [Source:HGNC Symbol;Acc:HGNC:5164]	protein coding	-0.36	6.52	15.89	<0.0001	0.008
ENSG00000121743	GJA3	gap junction protein alpha 3 [Source:HGNC Symbol;Acc:HGNC:4277]	protein coding	-0.36	5.50	15.31	<0.0001	0.0096
ENSG00000116701	NCF2	neutrophil cytosolic factor 2 [Source:HGNC Symbol;Acc:HGNC:7661]	protein coding	-0.35	8.08	16.17	<0.0001	0.0076
ENSG00000075223	SEMA3C	semaphorin 3C [Source:HGNC Symbol;Acc:HGNC:10725]	protein coding	-0.35	6.58	11.40	0.0007	0.035
ENSG00000134531	EMP1	epithelial membrane protein 1 [Source:HGNC Symbol;Acc:HGNC:3333]	protein coding	-0.35	7.10	17.95	<0.0001	0.0039
ENSG00000184371	CSF1	colony stimulating factor 1 [Source:HGNC Symbol;Acc:HGNC:2432]	protein coding	0.35	4.45	10.75	0.001	0.0425
ENSG00000103855	CD276	CD276 molecule [Source:HGNC Symbol;Acc:HGNC:19137]	protein coding	-0.35	5.22	11.21	0.0008	0.0375
ENSG00000088256	GNA11	G protein subunit alpha 11 [Source:HGNC Symbol;Acc:HGNC:4379]	protein coding	-0.35	3.71	15.56	<0.0001	0.0089
ENSG00000221866	PLXNA4	plexin A4 [Source:HGNC Symbol;Acc:HGNC:9102]	protein coding	0.35	3.04	11.94	0.0006	0.029
ENSG00000186431	FCAR	Fc fragment of IgA receptor [Source:HGNC Symbol;Acc:HGNC:3608]	protein coding	-0.35	6.66	11.00	0.0009	0.0392
ENSG00000283787			protein coding	0.34	3.68	14.89	0.0001	0.011
ENSG00000267737			lincRNA	-0.34	2.41	11.65	0.0006	0.0321
ENSG00000090376	IRAK3	interleukin 1 receptor associated kinase 3 [Source:HGNC Symbol;Acc:HGNC:17020]	protein coding	-0.34	7.55	16.03	<0.0001	0.008
ENSG00000127507	ADGRE2	adhesion G protein-coupled receptor E2 [Source:HGNC Symbol;Acc:HGNC:3337]	protein coding	-0.34	7.08	15.91	<0.0001	0.008
ENSG00000138111	MFSD13A	major facilitator superfamily domain containing 13A [Source:HGNC Symbol;Acc:HGNC:26196]	protein coding	-0.34	3.60	11.13	0.0008	0.038
ENSG00000171105	INSR	insulin receptor [Source:HGNC Symbol;Acc:HGNC:6091]	protein coding	-0.34	3.66	11.01	0.0009	0.0392
ENSG00000059728	MXD1	MAX dimerization protein 1 [Source:HGNC Symbol;Acc:HGNC:6761]	protein coding	-0.34	8.59	14.25	0.0002	0.0132

Supplementary Table 11: Genes differentially expressed at day 28 post-second vaccination compared to day 0 (RNA-Seq). Sorted by descending absolute log_2 fold change (Day 28 post-second vaccination vs. Day 0). Gene model summaries and annotations are based on Ensembl Version 90 (August 2017) (continued).

Ensembl Gene ID	Ensembl Gene Name	Ensembl Gene Description	Gene Type	Log_2 Fold Change (Day 28 vs. Day 0)	Average Log_2 CPM	Likelihood Ratio Test Statistic	P-Value	FDR Adjusted P-Value
ENSG00000100284	TOM1	target of myb1 membrane trafficking protein [Source:HGNC Symbol;Acc:HGNC:11982]	protein coding	-0.34	7.77	22.77	<0.0001	0.0007
ENSG00000125505	MBOAT7	membrane bound O-acyltransferase domain containing 7 [Source:HGNC Symbol;Acc:HGNC:15505]	protein coding	-0.34	7.36	14.43	0.0001	0.0128
ENSG00000164023	SGMS2	sphingomyelin synthase 2 [Source:HGNC Symbol;Acc:HGNC:28395]	protein coding	-0.34	4.12	13.15	0.0003	0.0193
ENSG00000197712	FAM114A1	family with sequence similarity 114 member A1 [Source:HGNC Symbol;Acc:HGNC:25087]	protein coding	-0.34	3.25	11.03	0.0009	0.0391
ENSG00000196083	IL1RAP	interleukin 1 receptor accessory protein [Source:HGNC Symbol;Acc:HGNC:5995]	protein coding	-0.34	4.65	13.02	0.0003	0.0202
ENSG00000182022	CHST15	carbohydrate sulfotransferase 15 [Source:HGNC Symbol;Acc:HGNC:18137]	protein coding	-0.34	7.48	15.18	<0.0001	0.0101
ENSG00000104763	ASAH1	N-acylsphingosine amidohydrolase 1 [Source:HGNC Symbol;Acc:HGNC:735]	protein coding	-0.33	7.92	10.88	0.001	0.0408
ENSG00000155099	TMEM55A	transmembrane protein 55A [Source:HGNC Symbol;Acc:HGNC:25452]	protein coding	-0.33	3.16	11.59	0.0007	0.0327
ENSG00000104974	LILRA1	leukocyte immunoglobulin like receptor A1 [Source:HGNC Symbol;Acc:HGNC:6602]	protein coding	-0.33	6.44	15.90	<0.0001	0.008
ENSG00000111321	LTBR	lymphotoxin beta receptor [Source:HGNC Symbol;Acc:HGNC:6718]	protein coding	-0.33	5.99	17.93	<0.0001	0.0039
ENSG00000169403	PTAFR	platelet activating factor receptor [Source:HGNC Symbol;Acc:HGNC:9582]	protein coding	-0.33	8.77	12.00	0.0005	0.0285
ENSG00000006118	TMEM132A	transmembrane protein 132A [Source:HGNC Symbol;Acc:HGNC:31092]	protein coding	-0.33	4.67	19.82	<0.0001	0.0019
ENSG00000143067	ZNF697	zinc finger protein 697 [Source:HGNC Symbol;Acc:HGNC:32034]	protein coding	-0.33	4.93	13.08	0.0003	0.0197
ENSG00000116962	NID1	nidogen 1 [Source:HGNC Symbol;Acc:HGNC:7821]	protein coding	-0.32	4.36	11.51	0.0007	0.0335
ENSG00000113719	ERGIC1	endoplasmic reticulum-golgi intermediate compartment 1 [Source:HGNC Symbol;Acc:HGNC:29205]	protein coding	-0.32	8.31	16.91	<0.0001	0.0057
ENSG00000105810	CDK6	cyclin dependent kinase 6 [Source:HGNC Symbol;Acc:HGNC:1777]	protein coding	0.32	6.29	19.85	<0.0001	0.0019
ENSG00000084234	APLP2	amyloid beta precursor like protein 2 [Source:HGNC Symbol;Acc:HGNC:598]	protein coding	-0.32	8.61	11.00	0.0009	0.0392

Supplementary Table 11: Genes differentially expressed at day 28 post-second vaccination compared to day 0 (RNA-Seq). Sorted by descending absolute log_2 fold change (Day 28 post-second vaccination vs. Day 0). Gene model summaries and annotations are based on Ensembl Version 90 (August 2017) (continued).

Ensembl Gene ID	Ensembl Gene Name	Ensembl Gene Description	Gene Type	Log_2 Fold Change (Day 28 vs. Day 0)	Average Log_2 CPM	Likelihood Ratio Test Statistic	P-Value	FDR Adjusted P-Value
ENSG00000140859	KIFC3	kinesin family member C3 [Source:HGNC Symbol;Acc:HGNC:6326]	protein coding	-0.32	6.35	13.25	0.0003	0.0189
ENSG00000095383	TBC1D2	TBC1 domain family member 2 [Source:HGNC Symbol;Acc:HGNC:18026]	protein coding	-0.32	5.68	19.60	<0.0001	0.0021
ENSG00000174837	ADGRE1	adhesion G protein-coupled receptor E1 [Source:HGNC Symbol;Acc:HGNC:3336]	protein coding	-0.32	8.31	10.96	0.0009	0.0398
ENSG00000100450	GZMH	granzyme H [Source:HGNC Symbol;Acc:HGNC:4710]	protein coding	0.32	5.34	11.48	0.0007	0.034
ENSG00000168389	MFSD2A	major facilitator superfamily domain containing 2A [Source:HGNC Symbol;Acc:HGNC:25897]	protein coding	-0.32	4.42	10.56	0.0012	0.0454
ENSG00000135636	DYSF	dysferlin [Source:HGNC Symbol;Acc:HGNC:3097]	protein coding	-0.32	4.90	18.01	<0.0001	0.0039
ENSG00000070614	NDST1	N-deacetylase and N-sulfotransferase 1 [Source:HGNC Symbol;Acc:HGNC:7680]	protein coding	-0.32	6.01	14.85	0.0001	0.0111
ENSG00000165886	UBTD1	ubiquitin domain containing 1 [Source:HGNC Symbol;Acc:HGNC:25683]	protein coding	-0.32	3.87	10.88	0.001	0.0408
ENSG00000137462	TLR2	toll like receptor 2 [Source:HGNC Symbol;Acc:HGNC:11848]	protein coding	-0.32	8.49	14.20	0.0002	0.0133
ENSG00000145632	PLK2	polo like kinase 2 [Source:HGNC Symbol;Acc:HGNC:19699]	protein coding	-0.32	3.27	12.69	0.0004	0.0226
ENSG00000060982	BCAT1	branched chain amino acid transaminase 1 [Source:HGNC Symbol;Acc:HGNC:976]	protein coding	-0.32	7.85	11.79	0.0006	0.0303
ENSG00000156642	NPTN	neuroplastin [Source:HGNC Symbol;Acc:HGNC:17867]	protein coding	-0.32	8.07	10.68	0.0011	0.0433
ENSG00000169252	ADRB2	adrenoceptor beta 2 [Source:HGNC Symbol;Acc:HGNC:286]	protein coding	-0.32	4.20	11.08	0.0009	0.0383
ENSG00000179630	LACC1	laccase domain containing 1 [Source:HGNC Symbol;Acc:HGNC:26789]	protein coding	-0.32	5.81	10.98	0.0009	0.0394
ENSG00000170542	SERPINB9	serpin family B member 9 [Source:HGNC Symbol;Acc:HGNC:8955]	protein coding	-0.31	9.33	12.07	0.0005	0.0282
ENSG00000135838	NPL	N-acetylneuraminate pyruvate lyase [Source:HGNC Symbol;Acc:HGNC:16781]	protein coding	-0.31	6.03	12.50	0.0004	0.0241
ENSG00000177628	GBA	glucosylceramidase beta [Source:HGNC Symbol;Acc:HGNC:4177]	protein coding	-0.31	6.04	16.01	<0.0001	0.008

Supplementary Table 11: Genes differentially expressed at day 28 post-second vaccination compared to day 0 (RNA-Seq). Sorted by descending absolute log_2 fold change (Day 28 post-second vaccination vs. Day 0). Gene model summaries and annotations are based on Ensembl Version 90 (August 2017) (continued).

**Ensembl Gene ID**	**Ensembl Gene Name**	**Ensembl Gene Description**	**Gene Type**	Log_2 **Fold Change (Day 28 vs. Day 0)**	**Average** Log_2 **CPM**	**Likelihood Ratio Test Statistic**	**P-Value**	**FDR Adjusted P-Value**
ENSG00000134532	SOX5	SRY-box 5 [Source:HGNC Symbol;Acc:HGNC:11201]	protein coding	-0.31	4.64	12.69	0.0004	0.0226
ENSG00000107104	KANK1	KN motif and ankyrin repeat domains 1 [Source:HGNC Symbol;Acc:HGNC:19309]	protein coding	-0.31	6.78	11.82	0.0006	0.0301
ENSG00000059377	TBXAS1	thromboxane A synthase 1 [Source:HGNC Symbol;Acc:HGNC:11609]	protein coding	-0.31	4.86	11.55	0.0007	0.033
ENSG00000039319	ZFYVE16	zinc finger FYVE-type containing 16 [Source:HGNC Symbol;Acc:HGNC:20756]	protein coding	-0.31	6.71	18.70	<0.0001	0.0029
ENSG00000163235	TGFA	transforming growth factor alpha [Source:HGNC Symbol;Acc:HGNC:11765]	protein coding	-0.31	6.12	14.44	0.0001	0.0128
ENSG00000118971	CCND2	cyclin D2 [Source:HGNC Symbol;Acc:HGNC:1583]	protein coding	0.31	8.38	13.90	0.0002	0.0148
ENSG00000142657	PGD	phosphogluconate dehydrogenase [Source:HGNC Symbol;Acc:HGNC:8891]	protein coding	-0.31	9.11	11.16	0.0008	0.0379
ENSG00000198363	ASPH	aspartate beta-hydroxylase [Source:HGNC Symbol;Acc:HGNC:757]	protein coding	-0.31	6.73	11.38	0.0007	0.0351
ENSG00000091436	MAP3K20	mitogen-activated protein kinase kinase kinase 20 [Source:HGNC Symbol;Acc:HGNC:17797]	protein coding	-0.31	6.69	12.79	0.0003	0.022
ENSG00000133961	NUMB	NUMB, endocytic adaptor protein [Source:HGNC Symbol;Acc:HGNC:8060]	protein coding	-0.31	6.65	20.99	<0.0001	0.0014
ENSG00000120162	MOB3B	MOB kinase activator 3B [Source:HGNC Symbol;Acc:HGNC:23825]	protein coding	-0.31	3.15	13.66	0.0002	0.0161
ENSG00000160746	ANO10	anoctamin 10 [Source:HGNC Symbol;Acc:HGNC:25519]	protein coding	-0.31	4.31	17.61	<0.0001	0.0044
ENSG00000104972	LILRB1	leukocyte immunoglobulin like receptor B1 [Source:HGNC Symbol;Acc:HGNC:6605]	protein coding	-0.31	8.63	10.79	0.001	0.0419
ENSG00000197965	MPZL1	myelin protein zero like 1 [Source:HGNC Symbol;Acc:HGNC:7226]	protein coding	-0.31	6.37	14.31	0.0002	0.0132
ENSG00000148926	ADM	adrenomedullin [Source:HGNC Symbol;Acc:HGNC:259]	protein coding	-0.30	7.49	11.56	0.0007	0.033
ENSG00000107551	RASSF4	Ras association domain family member 4 [Source:HGNC Symbol;Acc:HGNC:20793]	protein coding	-0.30	6.55	13.28	0.0003	0.0188
ENSG00000167995	BEST1	bestrophin 1 [Source:HGNC Symbol;Acc:HGNC:12703]	protein coding	-0.30	5.37	15.41	<0.0001	0.0092

Supplementary Table 11: Genes differentially expressed at day 28 post-second vaccination compared to day 0 (RNA-Seq). Sorted by descending absolute log_2 fold change (Day 28 post-second vaccination vs. Day 0). Gene model summaries and annotations are based on Ensembl Version 90 (August 2017) (continued).

Ensembl Gene ID	Ensembl Gene Name	Ensembl Gene Description	Gene Type	Log_2 Fold Change (Day 28 vs. Day 0)	Average Log_2 CPM	Likelihood Ratio Test Statistic	P-Value	FDR Adjusted P-Value
ENSG00000175066	GK5	glycerol kinase 5 (putative) [Source:HGNC Symbol;Acc:HGNC:28635]	protein coding	-0.30	5.92	14.80	0.0001	0.0112
ENSG00000140678	ITGAX	integrin subunit alpha X [Source:HGNC Symbol;Acc:HGNC:6152]	protein coding	-0.30	7.03	16.49	<0.0001	0.0067
ENSG00000186818	LILRB4	leukocyte immunoglobulin like receptor B4 [Source:HGNC Symbol;Acc:HGNC:6608]	protein coding	-0.30	8.46	10.63	0.0011	0.0441
ENSG00000113269	RNF130	ring finger protein 130 [Source:HGNC Symbol;Acc:HGNC:18280]	protein coding	-0.30	6.84	13.28	0.0003	0.0188
ENSG00000133805	AMPD3	adenosine monophosphate deaminase 3 [Source:HGNC Symbol;Acc:HGNC:470]	protein coding	-0.30	7.40	17.18	<0.0001	0.0052
ENSG00000120949	TNFRSF8	TNF receptor superfamily member 8 [Source:HGNC Symbol;Acc:HGNC:11923]	protein coding	-0.30	5.36	12.18	0.0005	0.0275
ENSG00000119900	OGFRL1	opioid growth factor receptor like 1 [Source:HGNC Symbol;Acc:HGNC:21378]	protein coding	-0.30	7.63	15.99	<0.0001	0.008
ENSG00000149798	CDC42EP2	CDC42 effector protein 2 [Source:HGNC Symbol;Acc:HGNC:16263]	protein coding	-0.30	6.27	17.16	<0.0001	0.0052
ENSG00000136630	HLX	H2.0 like homeobox [Source:HGNC Symbol;Acc:HGNC:4978]	protein coding	-0.30	6.56	14.70	0.0001	0.0114
ENSG00000143570	SLC39A1	solute carrier family 39 member 1 [Source:HGNC Symbol;Acc:HGNC:12876]	protein coding	-0.29	8.14	12.81	0.0003	0.0219
ENSG00000119408	NEK6	NIMA related kinase 6 [Source:HGNC Symbol;Acc:HGNC:7749]	protein coding	-0.29	4.62	14.62	0.0001	0.0118
ENSG00000198719	DLL1	delta like canonical Notch ligand 1 [Source:HGNC Symbol;Acc:HGNC:2908]	protein coding	-0.29	5.08	10.72	0.0011	0.0429
ENSG00000056972	TRAF3IP2	TRAF3 interacting protein 2 [Source:HGNC Symbol;Acc:HGNC:1343]	protein coding	-0.29	6.43	21.81	<0.0001	0.001
ENSG00000005238	FAM214B	family with sequence similarity 214 member B [Source:HGNC Symbol;Acc:HGNC:25666]	protein coding	-0.29	6.07	20.80	<0.0001	0.0014
ENSG00000105355	PLIN3	perilipin 3 [Source:HGNC Symbol;Acc:HGNC:16893]	protein coding	-0.29	6.36	13.62	0.0002	0.0162
ENSG00000135678	CPM	carboxypeptidase M [Source:HGNC Symbol;Acc:HGNC:2311]	protein coding	-0.29	5.71	10.36	0.0013	0.0485

Supplementary Table 11: Genes differentially expressed at day 28 post-second vaccination compared to day 0 (RNA-Seq). Sorted by descending absolute log_2 fold change (Day 28 post-second vaccination vs. Day 0). Gene model summaries and annotations are based on Ensembl Version 90 (August 2017) (continued).

Ensembl Gene ID	Ensembl Gene Name	Ensembl Gene Description	Gene Type	Log_2 Fold Change (Day 28 vs. Day 0)	Average Log_2 CPM	Likelihood Ratio Test Statistic	P-Value	FDR Adjusted P-Value
ENSG00000117090	SLAMF1	signaling lymphocytic activation molecule family member 1 [Source:HGNC Symbol;Acc:HGNC:10903]	protein coding	-0.29	8.13	11.93	0.0006	0.029
ENSG00000142192	APP	amyloid beta precursor protein [Source:HGNC Symbol;Acc:HGNC:620]	protein coding	-0.29	7.03	11.83	0.0006	0.0301
ENSG00000006432	MAP3K9	mitogen-activated protein kinase kinase kinase 9 [Source:HGNC Symbol;Acc:HGNC:6861]	protein coding	0.29	3.65	10.35	0.0013	0.0485
ENSG00000158186	MRAS	muscle RAS oncogene homolog [Source:HGNC Symbol;Acc:HGNC:7227]	protein coding	-0.29	4.89	12.10	0.0005	0.0282
ENSG00000136052	SLC41A2	solute carrier family 41 member 2 [Source:HGNC Symbol;Acc:HGNC:31045]	protein coding	-0.28	6.70	12.88	0.0003	0.0213
ENSG00000273749	CYFIP1	cytoplasmic FMR1 interacting protein 1 [Source:HGNC Symbol;Acc:HGNC:13759]	protein coding	-0.28	7.06	12.47	0.0004	0.0243
ENSG00000204577	LILRB3	leukocyte immunoglobulin like receptor B3 [Source:HGNC Symbol;Acc:HGNC:6607]	protein coding	-0.28	6.33	12.02	0.0005	0.0285
ENSG00000110880	CORO1C	coronin 1C [Source:HGNC Symbol;Acc:HGNC:2254]	protein coding	-0.28	6.76	15.16	<0.0001	0.0101
ENSG00000139629	GALNT6	polypeptide N-acetylgalactosaminyltransferase 6 [Source:HGNC Symbol;Acc:HGNC:4128]	protein coding	-0.28	5.88	11.20	0.0008	0.0375
ENSG00000161638	ITGA5	integrin subunit alpha 5 [Source:HGNC Symbol;Acc:HGNC:6141]	protein coding	-0.28	8.56	13.88	0.0002	0.0149
ENSG00000066697	MSANTD3	Myb/SANT DNA binding domain containing 3 [Source:HGNC Symbol;Acc:HGNC:23370]	protein coding	-0.28	5.40	14.27	0.0002	0.0132
ENSG00000006534	ALDH3B1	aldehyde dehydrogenase 3 family member B1 [Source:HGNC Symbol;Acc:HGNC:410]	protein coding	-0.28	5.16	14.34	0.0002	0.0132
ENSG00000133612	AGAP3	ArfGAP with GTPase domain, ankyrin repeat and PH domain 3 [Source:HGNC Symbol;Acc:HGNC:16923]	protein coding	-0.28	6.81	17.77	<0.0001	0.0042
ENSG00000145685	LHFPL2	LHFPL tetraspan subfamily member 2 [Source:HGNC Symbol;Acc:HGNC:6588]	protein coding	-0.28	8.67	11.62	0.0007	0.0326
ENSG00000146094	DOK3	docking protein 3 [Source:HGNC Symbol;Acc:HGNC:24583]	protein coding	-0.28	5.97	17.47	<0.0001	0.0046

Supplementary Table 11: Genes differentially expressed at day 28 post-second vaccination compared to day 0 (RNA-Seq). Sorted by descending absolute log_2 fold change (Day 28 post-second vaccination vs. Day 0). Gene model summaries and annotations are based on Ensembl Version 90 (August 2017) (continued).

**Ensembl Gene ID**	**Ensembl Gene Name**	**Ensembl Gene Description**	**Gene Type**	Log_2 **Fold Change (Day 28 vs. Day 0)**	**Average** Log_2 **CPM**	**Likelihood Ratio Test Statistic**	**P-Value**	**FDR Adjusted P-Value**
ENSG00000148288	GBGT1	globoside alpha-1,3-N-acetylgalactosaminyltransferase 1 (FORS blood group) [Source:HGNC Symbol;Acc:HGNC:20460]	protein coding	-0.28	4.91	16.63	<0.0001	0.0063
ENSG00000100600	LGMN	legumain [Source:HGNC Symbol;Acc:HGNC:9472]	protein coding	-0.28	5.29	13.18	0.0003	0.0193
ENSG00000180061	TMEM150B	transmembrane protein 150B [Source:HGNC Symbol;Acc:HGNC:34415]	protein coding	-0.28	5.56	14.80	0.0001	0.0112
ENSG00000033627	ATP6V0A1	ATPase H+ transporting V0 subunit a1 [Source:HGNC Symbol;Acc:HGNC:865]	protein coding	-0.28	4.81	14.10	0.0002	0.0137
ENSG00000135912	TTLL4	tubulin tyrosine ligase like 4 [Source:HGNC Symbol;Acc:HGNC:28976]	protein coding	-0.27	4.99	14.84	0.0001	0.0111
ENSG00000241839	PLEKHO2	pleckstrin homology domain containing O2 [Source:HGNC Symbol;Acc:HGNC:30026]	protein coding	-0.27	8.30	12.21	0.0005	0.0271
ENSG00000161921	CXCL16	C-X-C motif chemokine ligand 16 [Source:HGNC Symbol;Acc:HGNC:16642]	protein coding	-0.27	6.74	10.41	0.0013	0.0479
ENSG00000256235	SMIM3	small integral membrane protein 3 [Source:HGNC Symbol;Acc:HGNC:30248]	protein coding	-0.27	6.54	13.68	0.0002	0.0161
ENSG00000135862	LAMC1	laminin subunit gamma 1 [Source:HGNC Symbol;Acc:HGNC:6492]	protein coding	-0.27	4.53	16.71	<0.0001	0.0062
ENSG00000157557	ETS2	ETS proto-oncogene 2, transcription factor [Source:HGNC Symbol;Acc:HGNC:3489]	protein coding	-0.27	8.50	10.47	0.0012	0.0465
ENSG00000052795	FNIP2	folliculin interacting protein 2 [Source:HGNC Symbol;Acc:HGNC:29280]	protein coding	-0.27	7.87	12.64	0.0004	0.0227
ENSG00000186350	RXRA	retinoid X receptor alpha [Source:HGNC Symbol;Acc:HGNC:10477]	protein coding	-0.27	6.89	14.22	0.0002	0.0132
ENSG00000067182	TNFRSF1A	TNF receptor superfamily member 1A [Source:HGNC Symbol;Acc:HGNC:11916]	protein coding	-0.27	7.40	14.06	0.0002	0.0138
ENSG00000135046	ANXA1	annexin A1 [Source:HGNC Symbol;Acc:HGNC:533]	protein coding	-0.27	8.34	11.25	0.0008	0.037
ENSG00000030419	IKZF2	IKAROS family zinc finger 2 [Source:HGNC Symbol;Acc:HGNC:13177]	protein coding	0.26	5.82	22.42	<0.0001	0.0009
ENSG00000135318	NT5E	5'-nucleotidase ecto [Source:HGNC Symbol;Acc:HGNC:8021]	protein coding	-0.26	4.49	11.96	0.0005	0.0289
ENSG00000213625	LEPROT	leptin receptor overlapping transcript [Source:HGNC Symbol;Acc:HGNC:29477]	protein coding	-0.26	6.31	12.26	0.0005	0.0266

Supplementary Table 11: Genes differentially expressed at day 28 post-second vaccination compared to day 0 (RNA-Seq). Sorted by descending absolute log_2 fold change (Day 28 post-second vaccination vs. Day 0). Gene model summaries and annotations are based on Ensembl Version 90 (August 2017) (continued).

**Ensembl Gene ID**	**Ensembl Gene Name**	**Ensembl Gene Description**	**Gene Type**	Log_2 **Fold Change (Day 28 vs. Day 0)**	**Average** Log_2 **CPM**	**Likelihood Ratio Test Statistic**	**P-Value**	**FDR Adjusted P-Value**
ENSG00000091073	DTX2	deltex E3 ubiquitin ligase 2 [Source:HGNC Symbol;Acc:HGNC:15973]	protein coding	-0.26	4.82	10.88	0.001	0.0408

Supplementary Table 12: KEGG Pathways enriched in differentially expressed genes at Day 14 post-second vaccination compared to pre-first vaccination (RNA-Seq). Results are sorted by FDR adjusted p-value and Jaccard similarity index. To determine significantly enriched gene sets, DE genes were used.

Gene Set Name	Gene Set Genes #	DE Genes N (%)	Up-reg. DE Genes N (%)	Down-reg. DE Genes N (%)	Jaccard Index	P-Value	FDR Adjusted P-Value
Cytokine-cytokine receptor interaction	257	22 (8.6)	12 (4.7)	10 (3.9)	0.038	<0.0001	0.0016
Fluid shear stress and atherosclerosis	137	15 (10.9)	2 (1.5)	13 (9.5)	0.033	<0.0001	0.0016
Osteoclast differentiation	124	13 (10.5)	2 (1.6)	11 (8.9)	0.029	<0.0001	0.0021
Jak-STAT signaling pathway	155	11 (7.1)	7 (4.5)	4 (2.6)	0.023	0.0003	0.018
Complement and coagulation cascades	76	8 (10.5)	0 (0)	8 (10.5)	0.020	0.0003	0.018
Staphylococcus aureus infection	52	6 (11.5)	0 (0)	6 (11.5)	0.016	0.0008	0.0412
Proteoglycans in cancer	200	13 (6.5)	0 (0)	13 (6.5)	0.025	0.0011	0.0437
Tuberculosis	172	11 (6.4)	2 (1.2)	9 (5.2)	0.022	0.001	0.0437

Supplementary Table 13: MSigDB Reactome Pathways enriched in differentially expressed genes at Day 14 post-second vaccination compared to pre-first vaccination (RNA-Seq). Results are sorted by FDR adjusted p-value and Jaccard similarity index. To determine significantly enriched gene sets, DE genes were used.

Gene Set Name	Gene Set Genes #	DE Genes N (%)	Up-reg. DE Genes N (%)	Down-reg. DE Genes N (%)	Jaccard Index	P-Value	FDR Adjusted P-Value
REACTOME CELL SURFACE INTERACTIONS AT THE VASCULAR WALL	83	9 (10.8)	0 (0)	9 (10.8)	0.022	<0.0001	0.0404

Supplementary Table 14: MSigDB Immunologic Signature Sets enriched in differentially expressed genes at Day 14 post-second vaccination compared to pre-first vaccination (RNA-Seq). Results are sorted by FDR adjusted p-value and Jaccard similarity index. To determine significantly enriched gene sets, DE genes were used.Top 50 results are listed.

Gene Set Name	Gene Set Genes #	DE Genes N (%)	Up-reg. DE Genes N (%)	Down-reg. DE Genes N (%)	Jaccard Index	P-Value	FDR Adjusted P-Value
GSE34156 UNTREATED VS 6H TLR1 TLR2 LIGAND TREATED MONOCYTE UP	178	42 (23.6)	0 (0)	42 (23.6)	0.089	<0.0001	0.0003
GSE30971 CTRL VS LPS STIM MACROPHAGE WBP7 KO 4H UP	171	40 (23.4)	1 (0.6)	39 (22.8)	0.086	<0.0001	0.0003
GSE36888 UNTREATED VS IL2 TREATED TCELL 6H UP	178	40 (22.5)	1 (0.6)	39 (21.9)	0.084	<0.0001	0.0003
GSE34156 UNTREATED VS 24H NOD2 LIGAND TREATED MONOCYTE DN	182	40 (22)	0 (0)	40 (22)	0.084	<0.0001	0.0003
GSE34156 NOD2 LIGAND VS TLR1 TLR2 LIGAND 6H TREATED MONOCYTE DN	175	39 (22.3)	0 (0)	39 (22.3)	0.083	<0.0001	0.0003
GSE30971 WBP7 HET VS KO MACROPHAGE DN	180	38 (21.1)	1 (0.6)	37 (20.6)	0.079	<0.0001	0.0003
GSE36888 UNTREATED VS IL2 TREATED TCELL 17H DN	180	38 (21.1)	1 (0.6)	37 (20.6)	0.079	<0.0001	0.0003
GSE10325 LUPUS BCELL VS LUPUS MYELOID DN	191	38 (19.9)	0 (0)	38 (19.9)	0.078	<0.0001	0.0003

Supplementary Table 14: MSigDB Immunologic Signature Sets enriched in differentially expressed genes at Day 14 post-second vaccination compared to pre-first vaccination (RNA-Seq). Results are sorted by FDR adjusted p-value and Jaccard similarity index. To determine significantly enriched gene sets, DE genes were used.Top 50 results are listed. (continued)

Gene Set Name	Gene Set Genes #	DE Genes N (%)	Up-reg. DE Genes N (%)	Down-reg. DE Genes N (%)	Jaccard Index	P-Value	FDR Adjusted P-Value
GSE30971 WBP7 HET VS KO MACROPHAGE 2H LPS STIM DN	182	37 (20.3)	1 (0.5)	36 (19.8)	0.077	<0.0001	0.0003
GSE30971 CTRL VS LPS STIM MACROPHAGE WBP7 KO 2H UP	170	36 (21.2)	1 (0.6)	35 (20.6)	0.076	<0.0001	0.0003
GSE29618 MONOCYTE VS PDC UP	187	37 (19.8)	0 (0)	37 (19.8)	0.076	<0.0001	0.0003
GSE10325 LUPUS CD4 TCELL VS LUPUS MYELOID DN	188	37 (19.7)	0 (0)	37 (19.7)	0.076	<0.0001	0.0003
GSE29618 MONOCYTE VS MDC DAY7 FLU VACCINE UP	188	37 (19.7)	0 (0)	37 (19.7)	0.076	<0.0001	0.0003
GSE29618 MONOCYTE VS MDC UP	189	37 (19.6)	0 (0)	37 (19.6)	0.076	<0.0001	0.0003
GSE22886 NAIVE CD8 TCELL VS MONOCYTE DN	191	37 (19.4)	0 (0)	37 (19.4)	0.075	<0.0001	0.0003
GSE36888 UNTREATED VS IL2 TREATED STAT5 AB KNOCKIN TCELL 2H UP	177	35 (19.8)	0 (0)	35 (19.8)	0.073	<0.0001	0.0003
GSE24634 TREG VS TCONV POST DAY10 IL4 CONVERSION DN	192	35 (18.2)	0 (0)	35 (18.2)	0.071	<0.0001	0.0003
GSE34156 TLR1 TLR2 LIGAND VS NOD2 AND TLR1 TLR2 LIGAND 24H TREATED MONOCYTE...	179	34 (19)	0 (0)	34 (19)	0.070	<0.0001	0.0003
GSE36888 UNTREATED VS IL2 TREATED STAT5 AB KNOCKIN TCELL 17H UP	172	32 (18.6)	0 (0)	32 (18.6)	0.067	<0.0001	0.0003
GSE36888 UNTREATED VS IL2 TREATED STAT5 AB KNOCKIN TCELL 6H UP	174	31 (17.8)	1 (0.6)	30 (17.2)	0.065	<0.0001	0.0003
GSE22886 NAIVE CD4 TCELL VS MONOCYTE DN	192	32 (16.7)	0 (0)	32 (16.7)	0.064	<0.0001	0.0003
GSE30971 CTRL VS LPS STIM MACROPHAGE WBP7 HET 2H UP	166	30 (18.1)	1 (0.6)	29 (17.5)	0.063	<0.0001	0.0003
GSE22886 DAY0 VS DAY1 MONOCYTE IN CULTURE DN	189	31 (16.4)	1 (0.5)	30 (15.9)	0.063	<0.0001	0.0003
GSE29618 MONOCYTE VS PDC DAY7 FLU VACCINE UP	190	31 (16.3)	0 (0)	31 (16.3)	0.062	<0.0001	0.0003
GSE22886 NAIVE TCELL VS MONOCYTE DN	191	31 (16.2)	0 (0)	31 (16.2)	0.062	<0.0001	0.0003
GSE6269 FLU VS STAPH AUREUS INF PBMC DN	158	29 (18.4)	0 (0)	29 (18.4)	0.062	<0.0001	0.0003
GSE6269 HEALTHY VS STAPH PNEUMO INF PBMC DN	160	26 (16.2)	0 (0)	26 (16.2)	0.055	<0.0001	0.0003
GSE25123 CTRL VS ROSIGLITAZONE STIM PPARG KO MACROPHAGE UP	181	27 (14.9)	0 (0)	27 (14.9)	0.055	<0.0001	0.0003
GSE10325 BCELL VS MYELOID DN	183	27 (14.8)	0 (0)	27 (14.8)	0.055	<0.0001	0.0003
GSE24634 TEFF VS TCONV DAY7 IN CULTURE DN	184	27 (14.7)	0 (0)	27 (14.7)	0.055	<0.0001	0.0003
GSE29618 BCELL VS MONOCYTE DAY7 FLU VACCINE DN	189	27 (14.3)	0 (0)	27 (14.3)	0.054	<0.0001	0.0003
GSE41176 UNSTIM VS ANTI IGM STIM BCELL 1H UP	183	26 (14.2)	0 (0)	26 (14.2)	0.053	<0.0001	0.0003
GSE11057 PBMC VS MEM CD4 TCELL UP	185	26 (14.1)	0 (0)	26 (14.1)	0.052	<0.0001	0.0003
GSE24634 TEFF VS TCONV DAY10 IN CULTURE DN	185	26 (14.1)	0 (0)	26 (14.1)	0.052	<0.0001	0.0003
GSE29618 BCELL VS MONOCYTE DN	187	26 (13.9)	0 (0)	26 (13.9)	0.052	<0.0001	0.0003
GSE25123 WT VS PPARG KO MACROPHAGE UP	173	25 (14.5)	0 (0)	25 (14.5)	0.051	<0.0001	0.0003
GSE22886 NAIVE BCELL VS MONOCYTE DN	195	26 (13.3)	0 (0)	26 (13.3)	0.051	<0.0001	0.0003
GSE24634 TEFF VS TCONV DAY3 IN CULTURE DN	177	25 (14.1)	0 (0)	25 (14.1)	0.051	<0.0001	0.0003
GSE9988 ANTI TREM1 AND LPS VS VEHICLE TREATED MONOCYTES UP	163	24 (14.7)	1 (0.6)	23 (14.1)	0.050	<0.0001	0.0003
GSE24634 TREG VS TCONV POST DAY7 IL4 CONVERSION DN	188	25 (13.3)	0 (0)	25 (13.3)	0.050	<0.0001	0.0003
GSE36888 STAT5 AB KNOCKIN VS WT TCELL IL2 TREATED 17H DN	179	24 (13.4)	0 (0)	24 (13.4)	0.049	<0.0001	0.0003
GSE41176 UNSTIM VS ANTI IGM STIM TAK1 KO BCELL 24H DN	179	24 (13.4)	1 (0.6)	23 (12.8)	0.049	<0.0001	0.0003

Supplementary Table 14: MSigDB Immunologic Signature Sets enriched in differentially expressed genes at Day 14 post-second vaccination compared to pre-first vaccination (RNA-Seq). Results are sorted by FDR adjusted p-value and Jaccard similarity index. To determine significantly enriched gene sets, DE genes were used.Top 50 results are listed. (continued)

Gene Set Name	Gene Set Genes #	DE Genes N (%)	Up-reg. DE Genes N (%)	Down-reg. DE Genes N (%)	Jaccard Index	P-Value	FDR Adjusted P-Value
GSE10325 CD4 TCELL VS MYELOID DN	185	24 (13)	0 (0)	24 (13)	0.048	<0.0001	0.0003
GSE22886 DAY1 VS DAY7 MONOCYTE IN CULTURE UP	185	24 (13)	1 (0.5)	23 (12.4)	0.048	<0.0001	0.0003
GSE25123 IL4 VS IL4 AND ROSIGLITAZONE STIM PPARG KO MACROPHAGE DAY10 DN	174	23 (13.2)	1 (0.6)	22 (12.6)	0.047	<0.0001	0.0003
GSE41176 UNSTIM VS ANTI IGM STIM BCELL 3H UP	184	23 (12.5)	0 (0)	23 (12.5)	0.046	<0.0001	0.0003
GSE41176 WT VS TAK1 KO ANTI IGM STIM BCELL 3H DN	184	23 (12.5)	1 (0.5)	22 (12)	0.046	<0.0001	0.0003
GSE9988 LPS VS CTRL TREATED MONOCYTE UP	165	22 (13.3)	1 (0.6)	21 (12.7)	0.046	<0.0001	0.0003
GSE9988 LOW LPS VS VEHICLE TREATED MONOCYTE UP	166	22 (13.3)	0 (0)	22 (13.3)	0.046	<0.0001	0.0003
GSE45365 NK CELL VS CD11B DC DN	191	23 (12)	2 (1)	21 (11)	0.045	<0.0001	0.0003

Supplementary Table 15: GO Biological Processes enriched in differentially expressed genes at Day 14 post-second vaccination compared to pre-first vaccination (RNA-Seq). Results are sorted by FDR adjusted p-value and Jaccard similarity index. To determine significantly enriched gene sets, DE genes were used.Top 50 results are listed.

Gene Set Name	Gene Set Genes #	DE Genes N (%)	Up-reg. DE Genes N (%)	Down-reg. DE Genes N (%)	Jaccard Index	P-Value	FDR Adjusted P-Value
GO IMMUNE RESPONSE	1028	57 (5.5)	17 (1.7)	40 (3.9)	0.044	<0.0001	0.0009
GO RESPONSE TO MOLECULE OF BACTERIAL ORIGIN	309	25 (8.1)	6 (1.9)	19 (6.1)	0.040	<0.0001	0.0009
GO IMMUNE SYSTEM PROCESS	1860	83 (4.5)	19 (1)	64 (3.4)	0.039	<0.0001	0.0009
GO INFLAMMATORY RESPONSE	430	29 (6.7)	6 (1.4)	23 (5.3)	0.039	<0.0001	0.0009
GO CELL MOTILITY	782	41 (5.2)	5 (0.6)	36 (4.6)	0.038	<0.0001	0.0009
GO RESPONSE TO BACTERIUM	506	30 (5.9)	8 (1.6)	22 (4.3)	0.037	<0.0001	0.0009
GO POSITIVE REGULATION OF LOCOMOTION	393	26 (6.6)	5 (1.3)	21 (5.3)	0.037	<0.0001	0.0009
GO DEFENSE RESPONSE	1158	53 (4.6)	13 (1.1)	40 (3.5)	0.037	<0.0001	0.0009
GO LOCOMOTION	1037	48 (4.6)	6 (0.6)	42 (4.1)	0.036	<0.0001	0.0009
GO RESPONSE TO LIPID	844	41 (4.9)	8 (0.9)	33 (3.9)	0.036	<0.0001	0.0009
GO POSITIVE REGULATION OF RESPONSE TO STIMULUS	1802	74 (4.1)	17 (0.9)	57 (3.2)	0.036	<0.0001	0.0009
GO POSITIVE REGULATION OF INTRACELLULAR SIGNAL TRANSDUCTION	824	40 (4.9)	12 (1.5)	28 (3.4)	0.036	<0.0001	0.0009
GO REGULATION OF CELLULAR COMPONENT MOVEMENT	716	36 (5)	7 (1)	29 (4.1)	0.035	<0.0001	0.0009
GO REGULATION OF RESPONSE TO WOUNDING	395	25 (6.3)	6 (1.5)	19 (4.8)	0.035	<0.0001	0.0009
GO POSITIVE REGULATION OF PHOSPHORUS METABOLIC PROCESS	968	44 (4.5)	11 (1.1)	33 (3.4)	0.035	<0.0001	0.0009
GO REGULATION OF CYTOKINE PRODUCTION	529	29 (5.5)	5 (0.9)	24 (4.5)	0.035	<0.0001	0.0009
GO REGULATION OF ANATOMICAL STRUCTURE MORPHOGENESIS	953	42 (4.4)	8 (0.8)	34 (3.6)	0.034	<0.0001	0.0009
GO REGULATION OF VASCULATURE DEVELOPMENT	218	18 (8.3)	4 (1.8)	14 (6.4)	0.034	<0.0001	0.0009

Supplementary Table 15: GO Biological Processes enriched in differentially expressed genes at Day 14 post-second vaccination compared to pre-first vaccination (RNA-Seq). Results are sorted by FDR adjusted p-value and Jaccard similarity index. To determine significantly enriched gene sets, DE genes were used.Top 50 results are listed. (continued)

Gene Set Name	Gene Set Genes #	DE Genes N (%)	Up-reg. DE Genes N (%)	Down-reg. DE Genes N (%)	Jaccard Index	P-Value	FDR Adjusted P-Value
GO TAXIS	436	25 (5.7)	5 (1.1)	20 (4.6)	0.033	<0.0001	0.0009
GO LEUKOCYTE MIGRATION	252	19 (7.5)	5 (2)	14 (5.6)	0.033	<0.0001	0.0009
GO POSITIVE REGULATION OF TRANSPORT	877	39 (4.4)	8 (0.9)	31 (3.5)	0.033	<0.0001	0.0009
GO NEGATIVE REGULATION OF MULTICELLULAR ORGANISMAL PROCESS	918	40 (4.4)	7 (0.8)	33 (3.6)	0.033	<0.0001	0.0009
GO POSITIVE REGULATION OF MULTICELLULAR ORGANISMAL PROCESS	1304	52 (4)	9 (0.7)	43 (3.3)	0.033	<0.0001	0.0009
GO REGULATION OF INFLAMMATORY RESPONSE	280	19 (6.8)	5 (1.8)	14 (5)	0.032	<0.0001	0.0009
GO REGULATION OF IMMUNE RESPONSE	803	35 (4.4)	5 (0.6)	30 (3.7)	0.032	<0.0001	0.0009
GO POSITIVE REGULATION OF CELL PROLIFERATION	769	34 (4.4)	11 (1.4)	23 (3)	0.032	<0.0001	0.0009
GO CELL CHEMOTAXIS	156	15 (9.6)	5 (3.2)	10 (6.4)	0.031	<0.0001	0.0009
GO POSITIVE REGULATION OF CELL COMMUNICATION	1444	54 (3.7)	13 (0.9)	41 (2.8)	0.031	<0.0001	0.0009
GO REGULATION OF IMMUNE SYSTEM PROCESS	1321	50 (3.8)	9 (0.7)	41 (3.1)	0.031	<0.0001	0.0009
GO POSITIVE REGULATION OF DEVELOPMENTAL PROCESS	1055	42 (4)	9 (0.9)	33 (3.1)	0.031	<0.0001	0.0009
GO REGULATION OF ENDOCYTOSIS	194	16 (8.2)	0 (0)	16 (8.2)	0.031	<0.0001	0.0009
GO POSITIVE REGULATION OF VASCULATURE DEVELOPMENT	128	14 (10.9)	1 (0.8)	13 (10.2)	0.031	<0.0001	0.0009
GO REGULATION OF INTRACELLULAR SIGNAL TRANSDUCTION	1546	56 (3.6)	15 (1)	41 (2.7)	0.031	<0.0001	0.0009
GO RESPONSE TO OXYGEN CONTAINING COMPOUND	1311	49 (3.7)	7 (0.5)	42 (3.2)	0.031	<0.0001	0.0009
GO RESPONSE TO BIOTIC STIMULUS	846	35 (4.1)	10 (1.2)	25 (3)	0.030	<0.0001	0.0009
GO REGULATION OF RESPONSE TO EXTERNAL STIMULUS	883	36 (4.1)	8 (0.9)	28 (3.2)	0.030	<0.0001	0.0009
GO POSITIVE REGULATION OF IMMUNE SYSTEM PROCESS	818	34 (4.2)	9 (1.1)	25 (3.1)	0.030	<0.0001	0.0009
GO RESPONSE TO EXTERNAL STIMULUS	1723	60 (3.5)	11 (0.6)	49 (2.8)	0.030	<0.0001	0.0009
GO POSITIVE REGULATION OF RESPONSE TO EXTERNAL STIMULUS	288	18 (6.2)	6 (2.1)	12 (4.2)	0.030	<0.0001	0.0009
GO REGULATION OF CELL PROLIFERATION	1400	50 (3.6)	12 (0.9)	38 (2.7)	0.030	<0.0001	0.0009
GO CELLULAR RESPONSE TO BIOTIC STIMULUS	157	14 (8.9)	4 (2.5)	10 (6.4)	0.029	<0.0001	0.0009
GO REGULATION OF DEFENSE RESPONSE	723	30 (4.1)	6 (0.8)	24 (3.3)	0.029	<0.0001	0.0009
GO REGULATION OF PHOSPHORUS METABOLIC PROCESS	1511	52 (3.4)	13 (0.9)	39 (2.6)	0.029	<0.0001	0.0009
GO LEUKOCYTE CHEMOTAXIS	112	12 (10.7)	5 (4.5)	7 (6.2)	0.028	<0.0001	0.0009
GO POSITIVE REGULATION OF RESPONSE TO WOUNDING	157	13 (8.3)	5 (3.2)	8 (5.1)	0.027	<0.0001	0.0009
GO POSITIVE REGULATION OF STAT CASCADE	72	10 (13.9)	4 (5.6)	6 (8.3)	0.025	<0.0001	0.0009
GO MYELOID LEUKOCYTE MIGRATION	95	10 (10.5)	4 (4.2)	6 (6.3)	0.024	<0.0001	0.0009
GO POSITIVE REGULATION OF INFLAMMATORY RESPONSE	110	10 (9.1)	4 (3.6)	6 (5.5)	0.023	<0.0001	0.0009
GO GRANULOCYTE MIGRATION	71	9 (12.7)	3 (4.2)	6 (8.5)	0.023	<0.0001	0.0009
GO NEGATIVE REGULATION OF INTERFERON GAMMA PRODUCTION	32	6 (18.8)	1 (3.1)	5 (15.6)	0.017	<0.0001	0.0009

Supplementary Table 16: GO Cellular Components enriched in differentially expressed genes at Day 14 post-second vaccination compared to pre-first vaccination (RNA-Seq). Results are sorted by FDR adjusted p-value and Jaccard similarity index. To determine significantly enriched gene sets, DE genes were used.

Gene Set Name	Gene Set Genes #	DE Genes N (%)	Up-reg. DE Genes N (%)	Down-reg. DE Genes N (%)	Jaccard Index	P-Value	FDR Adjusted P-Value
GO EXTRACELLULAR SPACE	1290	64 (5)	13 (1)	51 (4)	0.041	<0.0001	0.0019
GO CELL SURFACE	711	41 (5.8)	5 (0.7)	36 (5.1)	0.041	<0.0001	0.0019
GO INTRINSIC COMPONENT OF PLASMA MEMBRANE	1526	70 (4.6)	4 (0.3)	66 (4.3)	0.039	<0.0001	0.0019
GO EXTERNAL SIDE OF PLASMA MEMBRANE	223	13 (5.8)	4 (1.8)	9 (4)	0.024	0.0002	0.0333

Supplementary Table 17: GO Molecular Functions enriched in differentially expressed genes at Day 14 post-second vaccination compared to pre-first vaccination (RNA-Seq). Results are sorted by FDR adjusted p-value and Jaccard similarity index. To determine significantly enriched gene sets, DE genes were used.

Gene Set Name	Gene Set Genes #	DE Genes N (%)	Up-reg. DE Genes N (%)	Down-reg. DE Genes N (%)	Jaccard Index	P-Value	FDR Adjusted P-Value
GO CYTOKINE ACTIVITY	210	20 (9.5)	10 (4.8)	10 (4.8)	0.038	<0.0001	0.003
GO CYTOKINE RECEPTOR BINDING	261	20 (7.7)	10 (3.8)	10 (3.8)	0.035	<0.0001	0.003
GO RECEPTOR BINDING	1391	53 (3.8)	11 (0.8)	42 (3)	0.032	<0.0001	0.003
GO GROWTH FACTOR RECEPTOR BINDING	127	11 (8.7)	1 (0.8)	10 (7.9)	0.024	<0.0001	0.0045
GO RECEPTOR ACTIVITY	1510	46 (3)	3 (0.2)	43 (2.8)	0.025	<0.0001	0.0054
GO GROWTH FACTOR ACTIVITY	150	10 (6.7)	3 (2)	7 (4.7)	0.021	0.0001	0.0195
GO IMMUNOGLOBULIN BINDING	21	4 (19)	0 (0)	4 (19)	0.011	0.0003	0.0373
GO CHEMOKINE RECEPTOR BINDING	55	5 (9.1)	3 (5.5)	2 (3.6)	0.013	0.0003	0.0394
GO CCR CHEMOKINE RECEPTOR BINDING	34	4 (11.8)	2 (5.9)	2 (5.9)	0.011	0.0004	0.042

Supplementary Table 18: KEGG Pathways enriched in differentially expressed genes at Day 28 post-second vaccination compared to pre-first vaccination (RNA-Seq). Results are sorted by FDR adjusted p-value and Jaccard similarity index. To determine significantly enriched gene sets, DE genes were used.

Gene Set Name	Gene Set Genes #	DE Genes N (%)	Up-reg. DE Genes N (%)	Down-reg. DE Genes N (%)	Jaccard Index	P-Value	FDR Adjusted P-Value
Cytokine-cytokine receptor interaction	257	20 (7.8)	6 (2.3)	14 (5.4)	0.038	<0.0001	0.0016
Fluid shear stress and atherosclerosis	137	13 (9.5)	1 (0.7)	12 (8.8)	0.032	<0.0001	0.0016
Osteoclast differentiation	124	12 (9.7)	2 (1.6)	10 (8.1)	0.030	<0.0001	0.0024
Hematopoietic cell lineage	91	9 (9.9)	2 (2.2)	7 (7.7)	0.024	<0.0001	0.0024

Supplementary Table 18: KEGG Pathways enriched in differentially expressed genes at Day 28 post-second vaccination compared to pre-first vaccination (RNA-Seq). Results are sorted by FDR adjusted p-value and Jaccard similarity index. To determine significantly enriched gene sets, DE genes were used. (continued)

Gene Set Name	Gene Set Genes #	DE Genes N (%)	Up-reg. DE Genes N (%)	Down-reg. DE Genes N (%)	Jaccard Index	P-Value	FDR Adjusted P-Value
Amoebiasis	93	10 (10.8)	2 (2.2)	8 (8.6)	0.027	<0.0001	0.0026
Proteoglycans in cancer	200	15 (7.5)	0 (0)	15 (7.5)	0.032	<0.0001	0.0043
IL-17 signaling pathway	92	8 (8.7)	3 (3.3)	5 (5.4)	0.021	0.0002	0.0083
Tuberculosis	172	11 (6.4)	1 (0.6)	10 (5.8)	0.024	0.0007	0.0201
Phagosome	145	10 (6.9)	0 (0)	10 (6.9)	0.024	0.0007	0.0201
Rheumatoid arthritis	85	7 (8.2)	3 (3.5)	4 (4.7)	0.019	0.0006	0.0201
Staphylococcus aureus infection	52	6 (11.5)	0 (0)	6 (11.5)	0.018	0.0007	0.0201
Complement and coagulation cascades	76	7 (9.2)	0 (0)	7 (9.2)	0.020	0.0009	0.0246

Supplementary Table 19: MSigDB Reactome Pathways enriched in differentially expressed genes at Day 28 post-second vaccination compared to pre-first vaccination (RNA-Seq). Results are sorted by FDR adjusted p-value and Jaccard similarity index. To determine significantly enriched gene sets, DE genes were used.

Gene Set Name	Gene Set Genes #	DE Genes N (%)	Up-reg. DE Genes N (%)	Down-reg. DE Genes N (%)	Jaccard Index	P-Value	FDR Adjusted P-Value
REACTOME CELL SURFACE INTERACTIONS AT THE VASCULAR WALL	83	10 (12)	0 (0)	10 (12)	0.028	<0.0001	0.0135

Supplementary Table 20: MSigDB Immunologic Signature Sets enriched in differentially expressed genes at Day 28 post-second vaccination compared to pre-first vaccination (RNA-Seq). Results are sorted by FDR adjusted p-value and Jaccard similarity index. To determine significantly enriched gene sets, DE genes were used.Top 50 results are listed.

Gene Set Name	Gene Set Genes #	DE Genes N (%)	Up-reg. DE Genes N (%)	Down-reg. DE Genes N (%)	Jaccard Index	P-Value	FDR Adjusted P-Value
GSE10325 LUPUS CD4 TCELL VS LUPUS MYELOID DN	188	41 (21.8)	0 (0)	41 (21.8)	0.094	<0.0001	0.0003
GSE10325 LUPUS BCELL VS LUPUS MYELOID DN	191	40 (20.9)	0 (0)	40 (20.9)	0.091	<0.0001	0.0003
GSE22886 NAIVE CD8 TCELL VS MONOCYTE DN	191	40 (20.9)	0 (0)	40 (20.9)	0.091	<0.0001	0.0003
GSE34156 UNTREATED VS 24H NOD2 LIGAND TREATED MONOCYTE DN	182	39 (21.4)	0 (0)	39 (21.4)	0.090	<0.0001	0.0003
GSE36888 UNTREATED VS IL2 TREATED TCELL 6H UP	178	38 (21.3)	0 (0)	38 (21.3)	0.089	<0.0001	0.0003
GSE34156 TLR1 TLR2 LIGAND VS NOD2 AND TLR1 TLR2 LIGAND 24H TREATED MONOCYTE...	179	37 (20.7)	0 (0)	37 (20.7)	0.086	<0.0001	0.0003
GSE34156 UNTREATED VS 6H TLR1 TLR2 LIGAND TREATED MONOCYTE UP	178	36 (20.2)	0 (0)	36 (20.2)	0.084	<0.0001	0.0003

Supplementary Table 20: MSigDB Immunologic Signature Sets enriched in differentially expressed genes at Day 28 post-second vaccination compared to pre-first vaccination (RNA-Seq). Results are sorted by FDR adjusted p-value and Jaccard similarity index. To determine significantly enriched gene sets, DE genes were used.Top 50 results are listed. (continued)

Gene Set Name	Gene Set Genes #	DE Genes N (%)	Up-reg. DE Genes N (%)	Down-reg. DE Genes N (%)	Jaccard Index	P-Value	FDR Adjusted P-Value
GSE34156 NOD2 LIGAND VS TLR1 TLR2 LIGAND 6H TREATED MONOCYTE DN	175	35 (20)	0 (0)	35 (20)	0.082	<0.0001	0.0003
GSE24634 TREG VS TCONV POST DAY10 IL4 CONVERSION DN	192	36 (18.8)	0 (0)	36 (18.8)	0.081	<0.0001	0.0003
GSE29618 MONOCYTE VS PDC UP	187	34 (18.2)	0 (0)	34 (18.2)	0.077	<0.0001	0.0003
GSE36888 UNTREATED VS IL2 TREATED STAT5 AB KNOCKIN TCELL 2H UP	177	33 (18.6)	0 (0)	33 (18.6)	0.076	<0.0001	0.0003
GSE36888 UNTREATED VS IL2 TREATED TCELL 17H DN	180	33 (18.3)	0 (0)	33 (18.3)	0.076	<0.0001	0.0003
GSE36888 UNTREATED VS IL2 TREATED STAT5 AB KNOCKIN TCELL 6H UP	174	31 (17.8)	0 (0)	31 (17.8)	0.072	<0.0001	0.0003
GSE29618 MONOCYTE VS MDC UP	189	32 (16.9)	0 (0)	32 (16.9)	0.072	<0.0001	0.0003
GSE6269 FLU VS STAPH AUREUS INF PBMC DN	158	29 (18.4)	0 (0)	29 (18.4)	0.069	<0.0001	0.0003
GSE29618 MONOCYTE VS PDC DAY7 FLU VACCINE UP	190	31 (16.3)	0 (0)	31 (16.3)	0.069	<0.0001	0.0003
GSE22886 NAIVE CD4 TCELL VS MONOCYTE DN	192	30 (15.6)	0 (0)	30 (15.6)	0.067	<0.0001	0.0003
GSE24634 TEFF VS TCONV DAY3 IN CULTURE DN	177	29 (16.4)	0 (0)	29 (16.4)	0.066	<0.0001	0.0003
GSE29618 MONOCYTE VS MDC DAY7 FLU VACCINE UP	188	29 (15.4)	0 (0)	29 (15.4)	0.065	<0.0001	0.0003
GSE22886 NAIVE TCELL VS MONOCYTE DN	191	29 (15.2)	0 (0)	29 (15.2)	0.064	<0.0001	0.0003
GSE22886 NAIVE BCELL VS MONOCYTE DN	195	29 (14.9)	0 (0)	29 (14.9)	0.064	<0.0001	0.0003
GSE30971 CTRL VS LPS STIM MACROPHAGE WBP7 KO 4H UP	171	27 (15.8)	0 (0)	27 (15.8)	0.062	<0.0001	0.0003
GSE29618 BCELL VS MONOCYTE DAY7 FLU VACCINE DN	189	28 (14.8)	0 (0)	28 (14.8)	0.062	<0.0001	0.0003
GSE10325 BCELL VS MYELOID DN	183	27 (14.8)	0 (0)	27 (14.8)	0.061	<0.0001	0.0003
GSE11057 PBMC VS MEM CD4 TCELL UP	185	27 (14.6)	0 (0)	27 (14.6)	0.060	<0.0001	0.0003
GSE41176 UNSTIM VS ANTI IGM STIM TAK1 KO BCELL 24H DN	179	26 (14.5)	1 (0.6)	25 (14)	0.059	<0.0001	0.0003
GSE30971 WBP7 HET VS KO MACROPHAGE DN	180	26 (14.4)	1 (0.6)	25 (13.9)	0.059	<0.0001	0.0003
GSE30971 CTRL VS LPS STIM MACROPHAGE WBP7 HET 2H UP	166	25 (15.1)	0 (0)	25 (15.1)	0.058	<0.0001	0.0003
GSE30971 CTRL VS LPS STIM MACROPHAGE WBP7 KO 2H UP	170	25 (14.7)	0 (0)	25 (14.7)	0.058	<0.0001	0.0003
GSE24634 TEFF VS TCONV DAY10 IN CULTURE DN	185	25 (13.5)	0 (0)	25 (13.5)	0.056	<0.0001	0.0003
GSE25123 WT VS PPARG KO MACROPHAGE UP	173	24 (13.9)	0 (0)	24 (13.9)	0.055	<0.0001	0.0003
GSE6269 HEALTHY VS STAPH PNEUMO INF PBMC DN	160	23 (14.4)	0 (0)	23 (14.4)	0.054	<0.0001	0.0003
GSE30971 WBP7 HET VS KO MACROPHAGE 2H LPS STIM DN	182	24 (13.2)	1 (0.5)	23 (12.6)	0.054	<0.0001	0.0003
GSE10325 CD4 TCELL VS MYELOID DN	185	24 (13)	0 (0)	24 (13)	0.053	<0.0001	0.0003
GSE22886 NAIVE BCELL VS NEUTROPHIL DN	185	24 (13)	0 (0)	24 (13)	0.053	<0.0001	0.0003
GSE11057 CD4 EFF MEM VS PBMC DN	186	24 (12.9)	0 (0)	24 (12.9)	0.053	<0.0001	0.0003
GSE29618 BCELL VS MONOCYTE DN	187	24 (12.8)	0 (0)	24 (12.8)	0.053	<0.0001	0.0003
GSE24634 TREG VS TCONV POST DAY7 IL4 CONVERSION DN	188	23 (12.2)	0 (0)	23 (12.2)	0.051	<0.0001	0.0003
GSE25123 CTRL VS ROSIGLITAZONE STIM PPARG KO MACROPHAGE UP	181	22 (12.2)	0 (0)	22 (12.2)	0.049	<0.0001	0.0003
GSE36888 UNTREATED VS IL2 TREATED STAT5 AB KNOCKIN TCELL 17H UP	172	21 (12.2)	0 (0)	21 (12.2)	0.048	<0.0001	0.0003
GSE36888 STAT5 AB KNOCKIN VS WT TCELL IL2 TREATED 17H DN	179	21 (11.7)	0 (0)	21 (11.7)	0.047	<0.0001	0.0003

Supplementary Table 20: MSigDB Immunologic Signature Sets enriched in differentially expressed genes at Day 28 post-second vaccination compared to pre-first vaccination (RNA-Seq). Results are sorted by FDR adjusted p-value and Jaccard similarity index. To determine significantly enriched gene sets, DE genes were used.Top 50 results are listed. (continued)

Gene Set Name	Gene Set Genes #	DE Genes N (%)	Up-reg. DE Genes N (%)	Down-reg. DE Genes N (%)	Jaccard Index	P-Value	FDR Adjusted P-Value
GSE24634 TEFF VS TCONV DAY7 IN CULTURE DN	184	21 (11.4)	0 (0)	21 (11.4)	0.046	<0.0001	0.0003
GSE9988 ANTI TREM1 AND LPS VS VEHICLE TREATED MONOCYTES UP	163	20 (12.3)	1 (0.6)	19 (11.7)	0.046	<0.0001	0.0003
GSE22140 GERMFREE VS SPF MOUSE CD4 TCELL DN	187	21 (11.2)	0 (0)	21 (11.2)	0.046	<0.0001	0.0003
GSE3982 MAC VS NKCELL UP	183	20 (10.9)	1 (0.5)	19 (10.4)	0.044	<0.0001	0.0003
GSE24634 IL4 VS CTRL TREATED NAIVE CD4 TCELL DAY5 UP	186	20 (10.8)	2 (1.1)	18 (9.7)	0.044	<0.0001	0.0003
GSE22886 DAY0 VS DAY1 MONOCYTE IN CULTURE DN	189	20 (10.6)	1 (0.5)	19 (10.1)	0.044	<0.0001	0.0003
GSE25123 IL4 VS IL4 AND ROSIGLITAZONE STIM PPARG KO MACROPHAGE DAY10 DN	174	19 (10.9)	1 (0.6)	18 (10.3)	0.043	<0.0001	0.0003
GSE2128 CTRL VS MIMETOPE NEGATIVE SELECTION DP THYMOCYTE NOD UP	177	19 (10.7)	0 (0)	19 (10.7)	0.043	<0.0001	0.0003
GSE19198 CTRL VS IL21 TREATED TCELL 1H DN	178	19 (10.7)	0 (0)	19 (10.7)	0.042	<0.0001	0.0003

Supplementary Table 21: GO Biological Processes enriched in differentially expressed genes at Day 28 post-second vaccination compared to pre-first vaccination (RNA-Seq). Results are sorted by FDR adjusted p-value and Jaccard similarity index. To determine significantly enriched gene sets, DE genes were used.Top 50 results are listed.

Gene Set Name	Gene Set Genes #	DE Genes N (%)	Up-reg. DE Genes N (%)	Down-reg. DE Genes N (%)	Jaccard Index	P-Value	FDR Adjusted P-Value
GO INFLAMMATORY RESPONSE	430	35 (8.1)	4 (0.9)	31 (7.2)	0.051	<0.0001	0.0006
GO LEUKOCYTE MIGRATION	252	23 (9.1)	1 (0.4)	22 (8.7)	0.044	<0.0001	0.0006
GO IMMUNE RESPONSE	1028	55 (5.4)	7 (0.7)	48 (4.7)	0.044	<0.0001	0.0006
GO RESPONSE TO MOLECULE OF BACTERIAL ORIGIN	309	25 (8.1)	3 (1)	22 (7.1)	0.044	<0.0001	0.0006
GO RESPONSE TO BACTERIUM	506	32 (6.3)	3 (0.6)	29 (5.7)	0.042	<0.0001	0.0006
GO REGULATION OF RESPONSE TO WOUNDING	395	27 (6.8)	1 (0.3)	26 (6.6)	0.041	<0.0001	0.0006
GO CELL MOTILITY	782	42 (5.4)	1 (0.1)	41 (5.2)	0.041	<0.0001	0.0006
GO TAXIS	436	28 (6.4)	2 (0.5)	26 (6)	0.040	<0.0001	0.0006
GO LOCOMOTION	1037	51 (4.9)	3 (0.3)	48 (4.6)	0.040	<0.0001	0.0006
GO POSITIVE REGULATION OF LOCOMOTION	393	26 (6.6)	2 (0.5)	24 (6.1)	0.040	<0.0001	0.0006
GO REGULATION OF CYTOKINE PRODUCTION	529	31 (5.9)	3 (0.6)	28 (5.3)	0.039	<0.0001	0.0006
GO IMMUNE SYSTEM PROCESS	1860	80 (4.3)	9 (0.5)	71 (3.8)	0.039	<0.0001	0.0006
GO REGULATION OF INFLAMMATORY RESPONSE	280	21 (7.5)	1 (0.4)	20 (7.1)	0.038	<0.0001	0.0006
GO POSITIVE REGULATION OF INTRACELLULAR SIGNAL TRANSDUCTION	824	40 (4.9)	7 (0.8)	33 (4)	0.037	<0.0001	0.0006
GO REGULATION OF CELLULAR COMPONENT MOVEMENT	716	36 (5)	4 (0.6)	32 (4.5)	0.037	<0.0001	0.0006
GO POSITIVE REGULATION OF CYTOKINE PRODUCTION	352	23 (6.5)	3 (0.9)	20 (5.7)	0.037	<0.0001	0.0006
GO POSITIVE REGULATION OF RESPONSE TO WOUNDING	157	16 (10.2)	1 (0.6)	15 (9.6)	0.037	<0.0001	0.0006

Supplementary Table 21: GO Biological Processes enriched in differentially expressed genes at Day 28 post-second vaccination compared to pre-first vaccination (RNA-Seq). Results are sorted by FDR adjusted p-value and Jaccard similarity index. To determine significantly enriched gene sets, DE genes were used.Top 50 results are listed. (continued)

Gene Set Name	Gene Set Genes #	DE Genes N (%)	Up-reg. DE Genes N (%)	Down-reg. DE Genes N (%)	Jaccard Index	P-Value	FDR Adjusted P-Value
GO WOUND HEALING	446	26 (5.8)	0 (0)	26 (5.8)	0.037	<0.0001	0.0006
GO RESPONSE TO LIPID	844	40 (4.7)	4 (0.5)	36 (4.3)	0.037	<0.0001	0.0006
GO RESPONSE TO WOUNDING	538	29 (5.4)	0 (0)	29 (5.4)	0.036	<0.0001	0.0006
GO POSITIVE REGULATION OF RESPONSE TO EXTERNAL STIMULUS	288	20 (6.9)	2 (0.7)	18 (6.2)	0.036	<0.0001	0.0006
GO DEFENSE RESPONSE	1158	50 (4.3)	5 (0.4)	45 (3.9)	0.036	<0.0001	0.0006
GO REGULATION OF DEFENSE RESPONSE	723	35 (4.8)	2 (0.3)	33 (4.6)	0.036	<0.0001	0.0006
GO POSITIVE REGULATION OF RESPONSE TO STIMULUS	1802	72 (4)	8 (0.4)	64 (3.6)	0.036	<0.0001	0.0006
GO POSITIVE REGULATION OF MULTICELLULAR ORGANISMAL PROCESS	1304	54 (4.1)	4 (0.3)	50 (3.8)	0.035	<0.0001	0.0006
GO CELL CHEMOTAXIS	156	15 (9.6)	1 (0.6)	14 (9)	0.035	<0.0001	0.0006
GO ENDOCYTOSIS	485	26 (5.4)	0 (0)	26 (5.4)	0.035	<0.0001	0.0006
GO POSITIVE REGULATION OF CELL PROLIFERATION	769	35 (4.6)	5 (0.7)	30 (3.9)	0.034	<0.0001	0.0006
GO REGULATION OF IMMUNE RESPONSE	803	36 (4.5)	1 (0.1)	35 (4.4)	0.034	<0.0001	0.0006
GO REGULATION OF RESPONSE TO EXTERNAL STIMULUS	883	38 (4.3)	3 (0.3)	35 (4)	0.034	<0.0001	0.0006
GO RESPONSE TO OXYGEN CONTAINING COMPOUND	1311	51 (3.9)	3 (0.2)	48 (3.7)	0.033	<0.0001	0.0006
GO REGULATION OF CELL ADHESION	589	28 (4.8)	3 (0.5)	25 (4.2)	0.033	<0.0001	0.0006
GO SINGLE ORGANISM CELL ADHESION	434	23 (5.3)	3 (0.7)	20 (4.6)	0.033	<0.0001	0.0006
GO REGULATION OF SECRETION	657	30 (4.6)	4 (0.6)	26 (4)	0.033	<0.0001	0.0006
GO MOVEMENT OF CELL OR SUBCELLULAR COMPONENT	1196	47 (3.9)	2 (0.2)	45 (3.8)	0.033	<0.0001	0.0006
GO REGULATION OF VESICLE MEDIATED TRANSPORT	438	23 (5.3)	2 (0.5)	21 (4.8)	0.033	<0.0001	0.0006
GO REGULATION OF CYTOKINE BIOSYNTHETIC PROCESS	90	12 (13.3)	2 (2.2)	10 (11.1)	0.033	<0.0001	0.0006
GO POSITIVE REGULATION OF DEFENSE RESPONSE	348	20 (5.7)	1 (0.3)	19 (5.5)	0.032	<0.0001	0.0006
GO RESPONSE TO BIOTIC STIMULUS	846	35 (4.1)	4 (0.5)	31 (3.7)	0.032	<0.0001	0.0006
GO REGULATION OF INTERLEUKIN 6 PRODUCTION	100	12 (12)	2 (2)	10 (10)	0.032	<0.0001	0.0006
GO POSITIVE REGULATION OF CELL COMMUNICATION	1444	53 (3.7)	7 (0.5)	46 (3.2)	0.032	<0.0001	0.0006
GO REGULATION OF RESPONSE TO STRESS	1383	51 (3.7)	4 (0.3)	47 (3.4)	0.032	<0.0001	0.0006
GO RESPONSE TO CORTICOSTEROID	170	14 (8.2)	1 (0.6)	13 (7.6)	0.032	<0.0001	0.0006
GO RESPONSE TO EXTERNAL STIMULUS	1723	61 (3.5)	5 (0.3)	56 (3.3)	0.031	<0.0001	0.0006
GO POSITIVE REGULATION OF PHOSPHORUS METABOLIC PROCESS	968	38 (3.9)	6 (0.6)	32 (3.3)	0.031	<0.0001	0.0006
GO REGULATION OF CYTOKINE SECRETION	140	13 (9.3)	2 (1.4)	11 (7.9)	0.031	<0.0001	0.0006
GO REGULATION OF CELL PROLIFERATION	1400	51 (3.6)	5 (0.4)	46 (3.3)	0.031	<0.0001	0.0006
GO POSITIVE REGULATION OF INFLAMMATORY RESPONSE	110	12 (10.9)	1 (0.9)	11 (10)	0.031	<0.0001	0.0006
GO LEUKOCYTE CHEMOTAXIS	112	12 (10.7)	1 (0.9)	11 (9.8)	0.031	<0.0001	0.0006
GO REGULATION OF ANATOMICAL STRUCTURE MORPHOGENESIS	953	37 (3.9)	4 (0.4)	33 (3.5)	0.031	<0.0001	0.0006

Supplementary Table 22: GO Cellular Components enriched in differentially expressed genes at Day 28 post-second vaccination compared to pre-first vaccination (RNA-Seq). Results are sorted by FDR adjusted p-value and Jaccard similarity index. To determine significantly enriched gene sets, DE genes were used.

Gene Set Name	Gene Set Genes #	DE Genes N (%)	Up-reg. DE Genes N (%)	Down-reg. DE Genes N (%)	Jaccard Index	P-Value	FDR Adjusted P-Value
GO CELL SURFACE	711	38 (5.3)	2 (0.3)	36 (5.1)	0.040	<0.0001	0.0019
GO INTRINSIC COMPONENT OF PLASMA MEMBRANE	1526	65 (4.3)	2 (0.1)	63 (4.1)	0.037	<0.0001	0.0019
GO EXTRACELLULAR SPACE	1290	51 (4)	6 (0.5)	45 (3.5)	0.033	<0.0001	0.0019
GO EXTERNAL SIDE OF PLASMA MEMBRANE	223	12 (5.4)	1 (0.4)	11 (4.9)	0.024	0.0003	0.0383
GO FILOPODIUM MEMBRANE	17	4 (23.5)	0 (0)	4 (23.5)	0.013	0.0003	0.0383
GO RECEPTOR COMPLEX	311	16 (5.1)	1 (0.3)	15 (4.8)	0.027	0.0004	0.0398
GO PHAGOCYTIC VESICLE	78	7 (9)	0 (0)	7 (9)	0.019	0.0005	0.0398
GO RUFFLE	147	10 (6.8)	1 (0.7)	9 (6.1)	0.024	0.0007	0.0478

Supplementary Table 23: GO Molecular Functions enriched in differentially expressed genes at Day 28 post-second vaccination compared to pre-first vaccination (RNA-Seq). Results are sorted by FDR adjusted p-value and Jaccard similarity index. To determine significantly enriched gene sets, DE genes were used.

Gene Set Name	Gene Set Genes #	DE Genes N (%)	Up-reg. DE Genes N (%)	Down-reg. DE Genes N (%)	Jaccard Index	P-Value	FDR Adjusted P-Value
GO RECEPTOR BINDING	1391	59 (4.2)	5 (0.4)	54 (3.9)	0.036	<0.0001	0.0015
GO GROWTH FACTOR RECEPTOR BINDING	127	13 (10.2)	1 (0.8)	12 (9.4)	0.032	<0.0001	0.0015
GO CYTOKINE RECEPTOR BINDING	261	16 (6.1)	5 (1.9)	11 (4.2)	0.030	<0.0001	0.0015
GO PEPTIDASE REGULATOR ACTIVITY	199	13 (6.5)	0 (0)	13 (6.5)	0.027	<0.0001	0.0015
GO CYTOKINE ACTIVITY	210	13 (6.2)	5 (2.4)	8 (3.8)	0.027	<0.0001	0.0015
GO RECEPTOR ACTIVITY	1510	45 (3)	2 (0.1)	43 (2.8)	0.026	<0.0001	0.0015
GO PEPTIDASE INHIBITOR ACTIVITY	164	12 (7.3)	0 (0)	12 (7.3)	0.027	<0.0001	0.0023
GO SERINE TYPE ENDOPEPTIDASE INHIBITOR ACTIVITY	88	9 (10.2)	0 (0)	9 (10.2)	0.025	<0.0001	0.0023
GO SIGNAL TRANSDUCER ACTIVITY	1602	45 (2.8)	4 (0.2)	41 (2.6)	0.024	<0.0001	0.0049
GO DEATH RECEPTOR ACTIVITY	23	5 (21.7)	1 (4.3)	4 (17.4)	0.016	<0.0001	0.0049
GO RAGE RECEPTOR BINDING	11	3 (27.3)	0 (0)	3 (27.3)	0.010	<0.0001	0.0049
GO PLATELET DERIVED GROWTH FACTOR RECEPTOR BINDING	14	4 (28.6)	0 (0)	4 (28.6)	0.013	0.0001	0.0105
GO SIGNALING RECEPTOR ACTIVITY	1283	36 (2.8)	2 (0.2)	34 (2.7)	0.023	0.0002	0.0114
GO METALLOENDOPEPTIDASE ACTIVITY	106	9 (8.5)	0 (0)	9 (8.5)	0.023	0.0002	0.0114
GO EXTRACELLULAR MATRIX BINDING	48	6 (12.5)	0 (0)	6 (12.5)	0.018	0.0002	0.0114
GO GROWTH FACTOR ACTIVITY	150	9 (6)	2 (1.3)	7 (4.7)	0.021	0.0002	0.013
GO METALLOPEPTIDASE ACTIVITY	174	11 (6.3)	0 (0)	11 (6.3)	0.024	0.0005	0.026
GO CYTOKINE BINDING	83	7 (8.4)	1 (1.2)	6 (7.2)	0.019	0.0007	0.033

Supplementary Table 24: Combination of genes correlated with log_2 transformed inverse Caspase-3 levels (RNA-Seq, Day 14 post-second vaccination). The predictor variables included standardized gene variables with $\geq$1.5 absolute fold change for the respective post-vaccination day in either treatment group. Selected genes: 16, mean squared error: 0.17, R-squared: 0.88). Sorted by absolute descending regression coefficient. Gene model summaries and annotations are based on Ensembl Version 90 (August 2017).

Gene ID	Gene Name	Gene Description	Gene Type	Transcripts	Max Exons	Coefficient
ENSG00000225107			lincRNA	1	4	-0.0969
ENSG00000005102	MEOX1	mesenchyme homeobox 1 [Source:HGNC Symbol;Acc:HGNC:7013]	protein coding	4	4	-0.0934
ENSG00000111537	IFNG	interferon gamma [Source:HGNC Symbol;Acc:HGNC:5438]	protein coding	1	4	0.0906
ENSG00000109099	PMP22	peripheral myelin protein 22 [Source:HGNC Symbol;Acc:HGNC:9118]	protein coding	9	6	-0.0722
ENSG00000227825	SLC9A7P1	solute carrier family 9 member 7 pseudogene 1 [Source:HGNC Symbol;Acc:HGNC:32679]	transcribed processed pseudogene	2	1	0.0704
ENSG00000187957	DNER	delta/notch like EGF repeat containing [Source:HGNC Symbol;Acc:HGNC:24456]	protein coding	2	13	0.0541
ENSG00000087116	ADAMTS2	ADAM metallopeptidase with thrombospondin type 1 motif 2 [Source:HGNC Symbol;Acc:HGNC:218]	protein coding	5	22	0.0521
ENSG00000201428	RN7SKP71	RNA, 7SK small nuclear pseudogene 71 [Source:HGNC Symbol;Acc:HGNC:45795]	misc RNA	1	1	0.0505
ENSG00000174145	NWD2	NACHT and WD repeat domain containing 2 [Source:HGNC Symbol;Acc:HGNC:29229]	protein coding	1	7	0.0463
ENSG00000263740	RN7SL4P	RNA, 7SL, cytoplasmic 4, pseudogene [Source:HGNC Symbol;Acc:HGNC:10039]	misc RNA	1	1	0.0425
ENSG00000093134	VNN3	vanin 3 [Source:HGNC Symbol;Acc:HGNC:16431]	protein coding	14	8	0.0359
ENSG00000244734	HBB	hemoglobin subunit beta [Source:HGNC Symbol;Acc:HGNC:4827]	protein coding	5	4	-0.0344
ENSG00000206073	SERPINB4	serpin family B member 4 [Source:HGNC Symbol;Acc:HGNC:10570]	protein coding	4	8	-0.0235
ENSG00000112116	IL17F	interleukin 17F [Source:HGNC Symbol;Acc:HGNC:16404]	protein coding	2	3	0.0209
ENSG00000178726	THBD	thrombomodulin [Source:HGNC Symbol;Acc:HGNC:11784]	protein coding	1	1	0.0073
ENSG00000211644	IGLV1-51	immunoglobulin lambda variable 1-51 [Source:HGNC Symbol;Acc:HGNC:5882]	IG V gene	1	2	0.0064

Supplementary Table 25: Regularized canonical correlation analysis model summary statistics (RNA-Seq)

**Study Visit**	**Variable Set 1**	**Variable Set 2**	**Input Gene Variables**	**Input PCTC or Cytokine Variables**	**Cross Validation Score**	**Canonical Correlation (C1)**	**Canonical Correlation (C2)**	**Explained Variance Gene Variables (C1+C2)**	**Explained Variance PCTC or Cytokine Variables (C1+C2)**
Day 14 Post-Vaccination 2	Gene Responses	Proliferating cytokine producing T-cells	62	14	0.56	1.00	1.00	0.53	0.18
Day 28 Post-Vaccination 2	Gene Responses	Proliferating cytokine producing T-cells	62	15	0.21	0.95	0.95	0.29	0.43
Day 14 Post-Vaccination 2	Gene Responses	Cytokine response	62	6	0.55	0.96	0.94	0.41	0.30
Day 28 Post-Vaccination 2	Gene Responses	Cytokine response	62	6	0.67	0.95	0.93	0.21	0.58

Supplementary Table 26: Number of excluded genes by gene type (RNA-Seq)

Category/Chromosome	Genes
mitochondrial ribosomal RNA (Mt_rRNA)	2
mitochondrial transfer RNA (Mt_tRNA)	22
ribosomal RNA (rRNA)	514
located on X chromosome	2367
located on Y chromosome	517
Total	3422

Supplementary Table 27: Overview of gene sets used for the enrichment analysis (RNA-Seq).

Category Type	Categories	Distinct #Genes In Set	Median #Genes Per Set
GO Biological Processes	4436	16612	37
GO Cellular Components	580	13053	47
GO Molecular Functions	901	15567	32
KEGG Pathways	321	7225	70
MSigDB Immunologic Signature Sets	4872	19756	203
MSigDB Reactome Pathways	674	6559	28

Supplementary Table 28: Summary human reference genome alignment statistics (RNA-Seq)

	Min	Q1	Median	Mean	Q3	Max	SD	MAD	N
Unmapped Reads [10^6]	0.36	0.64	0.89	1.49	1.67	6.74	1.47	0.44	35
Total Mapped Reads [10^6]	7.95	13.69	21.00	34.56	39.05	162.14	34.78	11.18	35
Uniquely Mapped Reads [10^6]	5.59	11.23	16.21	27.75	31.91	130.55	28.37	8.58	35
Uniquely Mapped Reads [%]	63.00	78.75	80.30	79.41	81.45	88.60	4.32	2.08	35
Counted Fragments [10^6]	3.62	8.06	11.67	19.95	23.02	97.39	20.66	6.48	35
Uniquely Mapped Reads + Strand [10^6]	2.84	5.67	8.17	13.99	16.00	65.77	14.29	4.34	35
Uniquely Mapped Reads - Strand [10^6]	2.75	5.55	8.05	13.76	15.91	64.78	14.08	4.26	35
Median GC [%]	48.00	52.00	52.00	51.49	52.00	52.00	1.01	0.00	35
Mean GC [%]	49.01	51.03	51.45	51.32	51.68	52.26	0.66	0.47	35

Supplementary Table 29: Summary human reference gene model alignment statistics (RNA-Seq)

	Min	Q1	Median	Mean	Q3	Max	SD	MAD	N
Exon Tags [%]	72.83	80.17	81.35	81.28	82.68	86.65	2.78	1.87	35
Intron Tags [%]	12.43	16.19	17.35	17.48	18.55	25.87	2.72	1.78	35
Intergenic Tags [%]	0.91	1.18	1.24	1.24	1.32	1.40	0.12	0.11	35
CDS Exons Tags Per Kb	54.26	110.10	163.57	276.39	300.93	1381.28	287.33	91.17	35
Intron Tags Per Kb	1.28	1.79	2.58	4.36	5.21	17.85	4.19	1.53	35
3’ UTR Tags Per Kb	26.76	57.16	79.10	139.21	160.93	670.63	144.46	44.80	35
5’ UTR Tags Per Kb	19.90	46.88	67.67	116.02	137.99	548.17	118.61	34.77	35
TSS upstream 10Kb Tags Per Kb	0.13	0.24	0.36	0.59	0.66	2.85	0.62	0.22	35
TES downstream 10Kb Tags Per Kb	0.34	0.53	0.84	1.39	1.57	6.35	1.42	0.50	35
Splicing Events [10^5]	8.24	17.49	26.81	44.97	48.03	229.53	47.75	15.39	35
Splicing Junctions [10^5]	1.01	1.24	1.40	1.53	1.71	2.70	0.39	0.25	35

Supplementary Table 30: Number of genes that passed the low expression cut off (RNA-Seq)

	**Genes**
**All Data**	12596

Supplementary Table 31: Regularized linear logistic regression model summary statistics (RNA-Seq). Log_2 transformed inverse Caspase-3 levels were used as the response variable. The predictor variables included standardized gene variables with ≥1.5 absolute fold change for the respective post-vaccination day in either treatment group. SDEG [%]: percent overlap between selected genes and differentially expressed genes.

**Study Visit**	**Input Gene Variables**	**Selected Gene Variables**	**Mean Squared Error**	**R-Squared**	**SDEG [%]**	**Opt. α**	**Opt. λ**	**Intercept**
Day 14 post-second vaccination	62	16	0.17	0.88	56	0.19	0.2171	0.6729

Supplementary Table 32: List of R packages and versions used for the analyses presented in this report. R version 3.4.1 (2017-06-30) 'Single Candle' run on Ubuntu (release 16.04.2 LTS, x86-64-pc-linux-gnu (64-bit) platform).

Package Name	Version	Package Name	Version	Package Name	Version
minqa	1.2.4	parallel	3.4.1	tidyr	0.7.1
corpcor	1.6.9	SummarizedExperiment	1.6.3	httpuv	1.3.5
XVector	0.16.0	SparseM	1.77	stats4	3.4.1
GenomicRanges	1.28.5	memoise	1.1.0	munsell	0.4.3
MatrixModels	0.4-1	gridExtra	2.3	colorspace	1.3-2
bit64	0.9-7	stringi	1.1.5	igraph	1.1.2
AnnotationDbi	1.38.2	S4Vectors	0.14.4	png	0.1-7
RSpectra	0.13-1	GenomicFeatures	1.28.4	Cairo	1.5-9
codetools	0.2-15	caTools	1.17.1	openxlsx	4.0.17
splines	3.4.1	BiocGenerics	0.22.0	stringr	1.2.0
jsonlite	1.5	BiocParallel	1.10.1	glmnet	2.0-13
nloptr	1.0.4	chron	2.3-50	foreach	1.4.4
Rsamtools	1.28.0	GenomeInfoDb	1.12.2	Matrix	1.2-10
pbkrtest	0.4-7	rlang	0.1.2	plyr	1.8.4
GO.db	3.4.1	pkgconfig	2.0.1	mixOmics	6.3.2
shiny	1.0.5	matrixStats	0.52.2	ggplot2	2.2.1
compiler	3.4.1	bitops	1.0-6	lattice	0.20-35
assertthat	0.2.0	rgl	0.98.1	RColorBrewer	1.1-2
lazyeval	0.2.0	evaluate	0.10.1	car	2.1-5
htmltools	0.3.6	purrr	0.2.3	gtools	3.5.0
quantreg	5.33	bindr	0.1	R.utils	2.5.0
tools	3.4.1	GenomicAlignments	1.12.2	R.oo	1.21.0
bindrcpp	0.2	htmlwidgets	0.9	R.methodsS3	1.7.1
gtable	0.2.0	bit	1.1-12	pvclust	2.0-0
glue	1.1.1	magrittr	1.5	sqldf	0.4-11
GenomeInfoDbData	0.99.0	R6	2.2.2	RSQLite	2.0
reshape2	1.4.2	IRanges	2.10.3	gsubfn	0.6-6
dplyr	0.7.3	DelayedArray	0.2.7	proto	1.0.0
Rcpp	0.12.12	DBI	0.7	gplots	3.0.1
Biobase	2.36.2	mgcv	1.8-17	MASS	7.3-47
Biostrings	2.44.2	RCurl	1.95-4.8	goseq	1.28.0
gdata	2.18.0	nnet	7.3-12	geneLenDataBase	1.12.0
nlme	3.1-131	tibble	1.3.4	BiasedUrn	1.07
rtracklayer	1.36.4	rARPACK	0.11-0	biomaRt	2.32.1
iterators	1.0.9	KernSmooth	2.23-15	edgeR	3.18.1
lme4	1.1-13	ellipse	0.3-8	limma	3.32.6
mime	0.5	locfit	1.5-9.1	xtable	1.8-2
XML	3.98-1.9	grid	3.4.1	knitr	1.20
zlibbioc	1.22.0	blob	1.1.0		
scales	0.5.0	digest	0.6.12		
